# Life-Long Radar Tracking of Bumblebees

**DOI:** 10.1371/journal.pone.0160333

**Published:** 2016-08-04

**Authors:** Joseph L. Woodgate, James C. Makinson, Ka S. Lim, Andrew M. Reynolds, Lars Chittka

**Affiliations:** 1 Department of Biological and Experimental Psychology, School of Biological and Chemical Sciences, Queen Mary University of London, London, United Kingdom; 2 Department of Agroecology, Rothamsted Research, Harpenden, Hertfordshire, United Kingdom; Arizona State University, UNITED STATES

## Abstract

Insect pollinators such as bumblebees play a vital role in many ecosystems, so it is important to understand their foraging movements on a landscape scale. We used harmonic radar to record the natural foraging behaviour of *Bombus terrestris audax* workers over their entire foraging career. Every flight ever made outside the nest by four foragers was recorded. Our data reveal where the bees flew and how their behaviour changed with experience, at an unprecedented level of detail. We identified how each bee’s flights fit into two categories—which we named exploration and exploitation flights—examining the differences between the two types of flight and how their occurrence changed over the course of the bees’ foraging careers. Exploitation of learned resources takes place during efficient, straight trips, usually to a single foraging location, and is seldom combined with exploration of other areas. Exploration of the landscape typically occurs in the first few flights made by each bee, but our data show that further exploration flights can be made throughout the bee’s foraging career. Bees showed striking levels of variation in how they explored their environment, their fidelity to particular patches, ratio of exploration to exploitation, duration and frequency of their foraging bouts. One bee developed a straight route to a forage patch within four flights and followed this route exclusively for six days before abandoning it entirely for a closer location; this second location had not been visited since her first exploratory flight nine days prior. Another bee made only rare exploitation flights and continued to explore widely throughout its life; two other bees showed more frequent switches between exploration and exploitation. Our data shed light on the way bumblebees balance exploration of the environment with exploitation of resources and reveal extreme levels of variation between individuals.

## Introduction

Recent advances in animal-tracking technology have brought within reach the goal of tracking every movement of individual animals over their entire lifetimes [1]. The potential of such life-long tracks to advance our understanding of animal behaviour has been compared to that of the advent of DNA sequencing [2], but the field is still in its infancy. Several ambitious studies have begun to reveal the ways in which long range migratory movements of birds develop throughout an individual’s life [3,4], and the effects of migration on mortality [5]. We present here the first study to track the complete flight history of large earth bumblebee (*Bombus terrestris audax*) foragers, from their first orientation flight until the permanent disappearance of the bees from the colony and from the radar. In doing so, we are able to tell the ‘life stories’ of our focal bees, providing fresh insights into the way they explore their environment and exploit the floral resources they find there, and answering a number of questions that can only be addressed by continuous, life-long monitoring: How do the flight characteristics of individual bees change over a lifetime? Do flights transform gradually from exploration of the environment to focussed foraging trips or switch suddenly? Does exploration occur only at the start of a foraging career or does it continue throughout a bee’s life? Do individuals switch fluidly between multiple foraging sites or specialise on a single one? And do individual bees show similar patterns over their lives or do different bees employ different foraging strategies? Globally, pollinator species are in decline [6–8], so a deeper understanding of their movements and foraging behaviour is needed for understanding population dynamics and informing conservation efforts [9].

Foraging behaviour in bees has long been studied [10,11], but investigations of space use in bees have historically been limited to training bees to a single artificial forage source in the field [12], or to lab studies, modelling and field studies in spatially restricted locations [13]. Honeybee researchers have had the added benefit of being able to interpret spatial information encoded in the waggle dance of honeybees in order to determine the profitable locations visited by honeybee foragers [12,14–15]. Waggle dance analysis has allowed honeybee researchers to study a wealth of information about honeybee space use, from how foraging effort changes throughout the season [16] to how honeybees use the waggle dance throughout their lives [17]. While waggle dance analysis has revealed much about the locations dancing foragers visit, it cannot demonstrate how these foragers explore their environment or travel to these locations.

Only in the last 20 years has harmonic radar technology made it possible to observe and record the flight paths of insect pollinators at ecologically relevant scales [18]. Capaldi et al. tracked the first few flights of naïve honeybees (*Apis mellifera*), showing that they learn about their local environment in a series of orientation flights of gradually increasing range, covering increasing areas [19]. Each orientation flight covers a different, narrow sector of the landscape, so that over a number of flights the entire area around the hive is learned [19]. Osborne et al. tracked the initial flights of naïve *B*. *terrestris*, finding that these flights were more complex in shape than those of honeybees, involving explorations in all directions during the course of a single flight [20]. Again the range and area covered increased on subsequent flights [20]. Gaps in the radar tracks occurring over patches of forage provided some evidence that bumblebee foragers may start to feed as early as their first flights, suggesting a dual purpose to these early flights: learning the position of home and exploring for available resources [20]. In the same study, Osborne et al. also investigated the transition from early exploration to more focussed foraging behaviour by tracking later flights of bumblebee workers: their second or third flights, or flights taking place after at least six bouts. They demonstrated that bumblebees spent longer than just their first flight exploring the surrounding environment, but that by the time of their sixth bout or later, the structure of flights was significantly different, resembling the flights of experienced foragers [20]. In an earlier study, Osborne et al. tracked experienced bumblebee foragers for a mean of three flights each [21]. These tracks were longer and straighter than the early flights of inexperienced bees and frequently ended at known patches of forage. Several bees returning with pollen provided further evidence that these were foraging flights [21]. The bees tracked by Osborne et al. appeared to focus their foraging activity on a single patch per foraging trip and showed directional constancy between flights, suggesting fidelity to a single patch [21]. In the 65 outward and 35 return flights from 21 bees monitored, Osborne et al. only observed one instance of a bee switching foraging locations between bouts [21].

The studies described above revealed a great deal about the structure of exploratory and foraging flights, but opened up a number of key questions that are unanswered as yet. Does the change in flight structure from inexperienced to experienced bees occur gradually or as a sudden transition? When and how do bees discover the forage sources they go on to exploit? No prior study has been able to track the activity of individual insects throughout their entire life history, or even a significant portion of their life, making it impossible to address these questions. Studies of foraging behaviour have tracked a few flights from each bee but had no knowledge of their previous or subsequent experience. Lihoreau et al. were the first to track individual bumblebees over many consecutive foraging bouts, using a combination of harmonic radar and motion video capture to detail the formation of efficient and repeatable foraging routes between artificial forage sources over time [22].

Harmonic radar allows us to combine finer-scale temporal data than is usually obtained from satellite tracks with the ability to track every movement of our focal bees over the several week timescale of their lives as foragers. This continuous monitoring of flight patterns allows us to pinpoint when and how particular sites were first discovered; to identify the exact point at which switches in behaviour take place; to tell which parts of the environment are being utilised by individuals at any one time; and to address a number of questions that would be beyond reach without detailed knowledge of an individual’s history and development. Through the analysis of this dataset on the lifelong tracking of four individual forager bees, detailing 31 days of flight activity and 244 flights, we provide the most detailed study to date of the dynamics of resource exploitation versus exploration in a foraging insect pollinator in a field setting. To the best of our knowledge, this dataset represents the first lifetime track of any individual animal in sufficiently high temporal resolution to examine foraging routes.

## Materials and Methods

### Study area

**Fig 1:**
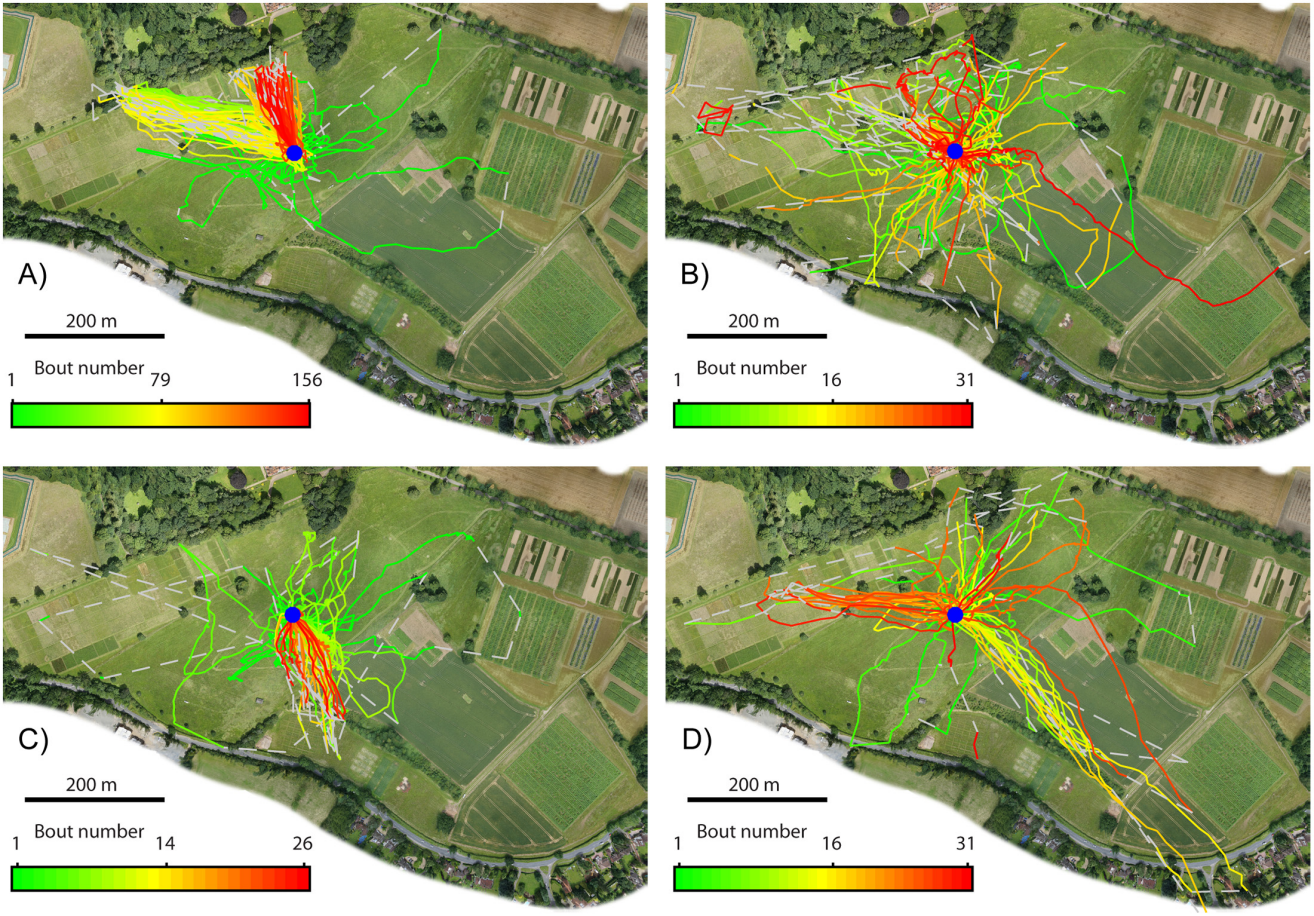
Flight paths of every flight made by four bees. Each panel represents the lifetime activity of a single bee. The position of the nest is marked by a blue circle. Each individual flight is shown in a different colour, the earliest flights undertaken by each bee in green, changing smoothly through yellow until the last flights in each bee’s life are shown in red. Grey dashed lines are used to join radar observations made more than 30 s apart, when the bee’s location was uncertain. A-D): flights of Bee 1–4 respectively.

Field work took place from June to September 2015 on arable farm land at Rothamsted Research (Hertfordshire, UK, 51’48”13N 0’22”8W, Fig 1), under approval from the Rothamsted field experiment committee. The main study site was an unmown field of approximately 700 x 300 m, containing grass and various seasonal wildflowers, mainly thistle (mostly *Cirsium arvense*, with some *Cirsium vulgare*), but also including dandelion (*Taraxacum officinale*), ragwort (*Senecio jacobaea*), daisy (*Bellis perennis*), wild carrot (*Daucus carota*) and hogweed (*Heracleum sphondylium*). On the Northwest our site was bordered by Park Grass, the world’s longest running ecological experimental wildflower and hay meadow [23], but this was mown shortly before the start of our study and provided little forage. To the North, the field was bordered by woodland, whose border was thickly grown with bramble (*Rubus fruticosus*) and by Rothamsted Manor, whose formal gardens contain a variety of cultivated flowers. The Southwestern and Southern limit of the site was a hedge, impervious to radar, bordering a road (B487) on the other side of which are playing fields and residential areas. Arable fields with few sources of forage bordered the site on all other sides.

### Colony preparation and focal bee selection

We used commercially sourced colonies of *Bombus terrestris audax* (Biobest NV, Westerlo, Belgium) that had never previously had access to the outside world. Colonies were kept in wooden nest boxes (30 cm x 21 cm x 16 cm high). A Perspex tunnel (26 cm x 4 cm x 4 cm high) allowed access to the outside world and could be blocked with a Perspex divider when necessary. Our nest boxes and tunnels were designed to give enough room that the bees could enter and leave, and move freely within the nest, with the transponder still attached. The transponders could get stuck in the wax of the nest but the bees usually managed to pull themselves free. We regularly checked the nest throughout the day to ensure we could free the focal bee if she became stuck.

Before transferring colonies to the field we allowed them free access to a small flight cage (45 cm x 45 cm x 45 cm high) containing a gravity feeder filled with 30% sucrose solution. Bees that fed from the feeder were marked using numbered tags (Opalith Zeichenplättchen Leuchtfarben, Bienen-Voigt & Warnholz, Ellerau, Germany). We monitored these bees for 1–2 days, choosing one bee that fed often and regularly as our focal bee.

The entire colony was then transferred to the field where it was placed on a wooden frame (98 cm high) and covered with the lid to a honeybee hive. Once in the field, the tunnel was opened and the entire colony was allowed to forage freely. This allowed us to track the behaviour of the focal bees while the colony operated as naturally as possible, with all foragers able to forage *ad libitum* and no need for supplementary feeding by us. Only one bee (the focal bee) can be tracked at a time using the radar. Foragers from the same colony could not be tracked sequentially since, once the colony was opened in the field, we could not be certain of any non-focal bee’s foraging history. Consequently, each focal bee came from a different colony.

### Radar tracking

Movements of the focal bees outside the nest were tracked using 32 mm harmonic radar (previously described in [18]). The radar was located at the Southeast edge of the experimental field and returned distance and direction coordinates of the bees’ position every 3 s while the bees remained in line-of-sight within a radius of about 800 m (accuracy ≈ ±2 m). The bees’ position often cannot be determined when they land on the ground or when they are in the radar-shadow cast by large objects such as trees or buildings. Flight data were visualised by converting the radar coordinates of the bees’ positions to GPS coordinates and superimposing the tracks onto a high-resolution geolocated orthomosaic image of the field site, created from a large number of aerial photographs taken from a UAV. Regulations prevented us from flying the UAV above the road which forms the Southern border of the field site, so the aerial image cannot show the area beyond the road. There was only one instance of a bee flying beyond the area shown on the image (Bee 4, flight 19, see S233 Fig). In this case the bee flew approximately 20 m further than can be seen on the figure.

A radar transponder consisting of a 16 mm vertical dipole was attached to the numbered tag on the thorax of each focal bee using superglue (Loctite Power Flex Gel, Henkel Ltd., Hemel Hempstead, UK). Transponders weigh around 15 mg. The transponder represents only 8–10% of a typical worker’s mass of 175–200 mg and bumblebees are known to carry nectar loads of up to 90% of their body mass [24]. Transponders have not been found to affect bees’ flight behaviour (although they may increase the duration of foraging bouts [21]).

We monitored the movements of the focal bee for 7–14 hours per day, depending on the weather. Tracking of the focal bee was terminated only if the bee did not return to the nest for 48 hours after which it was presumed to be dead or lost to the colony. The longest absence from which a bee ever returned to the nest was 20h12m. Overnight and on days when it was too rainy or windy to operate the radar, we blocked the tunnel entrance using a divider with an 8mm hole in it. Non-focal bees quickly learned to pass through this hole and foraged normally, but the length of the transponder prevented the focal bee from passing through and ensured that no flight could occur when the radar was not running. On five occasions the focal bee did not return to the nest before sunset so her movements could not be accounted for overnight. On these occasions we returned with the radar soon after sunrise the next morning and it is unlikely that we missed any flight, since bumblebees do not fly in the dark. No bee that had stayed out overnight was ever found to be already flying or attempting to re-enter the nest when we began recording the following day, and the directions from which they were first sighted were consistent with our last sightings the previous evening. On one occasion Bee 2 was observed in the field after sunset, sitting on the stalk of a thistle, just below the flower, and she was still in place when we set up the radar the following morning, resuming foraging at 8:15 am, 1h30m after we had begun recording.

### Field observations

When a bee remained static for a period, an experimenter watching the radar screen could use a custom-written Matlab script to convert the radar coordinates of the last known position of the bee to GPS coordinates. This was communicated to a second observer in the field who used a handheld GPS device (Garmin (Europe) Ltd., Southampton, UK) to search the area around the last sighting of the bee. Using this method, it was sometimes possible to get visual observations of the focal bee in the field. When observations were possible we recorded whether or not the bee was feeding on a flower and used individually numbered stakes to mark the locations of plants on which the bee had foraged. Visual observations were difficult to obtain since the radar only gives us the bee’s position accurate to approximately 2 m, and bees can sometimes make short flights between flowers that do not show up on radar due to obstructions by other plants or the landscape topography, meaning that the observer had to search within a radius of at least 5 m, in a complex landscape of tall stems and flowers where a single bee is difficult to spot. Observations could not be made systematically since they were only successful when the bee remained in the same location long enough for the observer to arrive and search, and because it was often not possible to locate the bee even if it had not moved.

### Data analysis

We extracted a number of variables from the flight tracks. The *flight duration* was the total time elapsed from leaving to re-entering the nest. *Time in flight* was the time during which the tracks showed the bee to be in motion. This excluded time that the bee was known to be stopped but also necessarily excludes time in which the bee was not detected by the radar but may have been in motion. The *flight distance* was the sum of the lengths of straight lines connecting every pair of radar coordinates, so will slightly underestimate the true distance travelled by the bee as bees do not move in perfectly straight lines between observations, and also necessarily excludes any movement made by the bee when out of range of the radar. *Maximum distance* for each flight was the straight-line distance from the nest to the furthest point recorded by the radar. *Digressiveness* was a measure of the shape of each flight and was defined as $\frac{Flight\;distance}{2*Maximum\;distance}$, such that the most efficient flight a bee could make (straight to the furthest point and back again) would have a digressiveness score of 1, while higher values indicate greater levels of digression from this efficient path.

Flights were categorised as either *exploitation* or *exploration flights*. *Exploitation flights* were defined as consisting of a single loop (i.e. the bee did not return within 15 m of the nest and then fly out again in a different direction) and including at least one stop in a location the bee had stopped at in the past. These exploitation flights, then, are flights that allow the bees to forage on familiar resources. All other flights have been categorised as *exploration*. These flights will be seen to differ systematically from the exploitation flights, being more digressive and covering more ground, but may not be solely exploratory in function. One of our focal bees was observed on four occasions at the end-point of exploitation flights, where she was foraging for nectar on thistles. In the case of the other bees we have no direct observations to confirm that they were foraging during flights that we classified, from the movement structure, as exploitation flights, although this seems most likely.

We analysed differences between flights in the two categories using four GLMMs (using the *glme* function of Matlab (Mathworks Inc., Natick, USA)), with flight duration, time in flight, flight distance and digressiveness as the dependent variables, all log transformed. Flight category (exploitation or exploration) was the predictor and bee ID was included as a random factor.

Two bees switched the destinations of the flights we categorised as exploitation flights during their foraging careers. To investigate whether doing so conferred benefits in foraging performance, we reanalysed the data for just the exploitation flights of these two bees. GLMMs were performed on the same four dependent variables as above, with the flight destination (first or second) as predictor and bee ID as a random factor.

## Results

We tracked four bees for periods of 6–15 days each (see Table 1). Our recordings represent every flight made by these bees over their entire life, within the range of the radar (Fig 1). Cumulatively, we recorded 244 flights, comprising of 15142 minutes of flight and covering a distance of 180 km. The paths of every individual flight are available as supplementary figures (S1–S7 Files; S2-S245 Figs). Each bee came from a different colony and the other foragers in each colony were allowed to forage naturally during the period of recording.

**Table 1:** Descriptive statistics for the lifetime activity of four bees.

	Bee 1	Bee 2	Bee 3	Bee 4
**Dates**	25/06/15–06/07/15	11/07/15–16/07/15	23/07/15–06/08/15	21/08/15–03/09/15
**Total days**	12	6	15	14
**Track days**	10	6	8	7
**Total flights**	156	31	26	31
**Explorations preceding 1st exploitation flight**	3	5	6	8
**Total number of exploitation flights**	142	7	17	16
**Proportion of flights that were exploitation**	0.91	0.23	0.65	0.52
**Flights/day (Mean ± sd)**	15.6 ± 8.8	5.2 ± 1.9	3.3 ± 0.9	4.4 ± 3.2
**Flight duration (min:sec, Mean ± sd)**	35:56 ± 119:41	133:10 ± 255:26	169:53 ± 205:33	31:59 ± 20:42
**Time in flight (min:sec, Mean ± sd)**	1:27 ± 2:15	3:43 ± 3:35	7:09 ± 10:52	2:32 ± 1:35
**Time between flights (min:sec, Mean ± sd)**	1:01 ± 1:14	25:44 ± 59:41	5:35 ± 14:46	26:14 ± 40:15
**Flight distance (m, Mean ± sd)**	442 ± 493	1362 ± 1366	1661 ± 2478	825 ± 485
**Max distance from nest (m, Mean ± sd)**	179 ± 71	217 ± 120	168 ± 72	257 ± 125
**Number of nights bee did not return to nest**	2	2	1	0
**Number of flights during which we made observations**	0	7	4	2
**Number of thistle patches bee observed in**	0	15	12	2
**Number of marked thistles**	0	50	34	4

Tracking terminated for two bees (Bee 1 and 3) when they failed to return from an otherwise typical foraging trip, suggesting they may have died during the foraging bout, perhaps as a result of predation by crab spiders or birds. Bee 2’s last recorded flight was a fast, straight flight in a direction that she had not previously exploited for forage and which took her beyond the range of the radar, never to return. Bee 4’s final flight was likewise made in a novel direction.

### Characteristics of flights by individual bees

#### Bee 1

**Fig 2:**
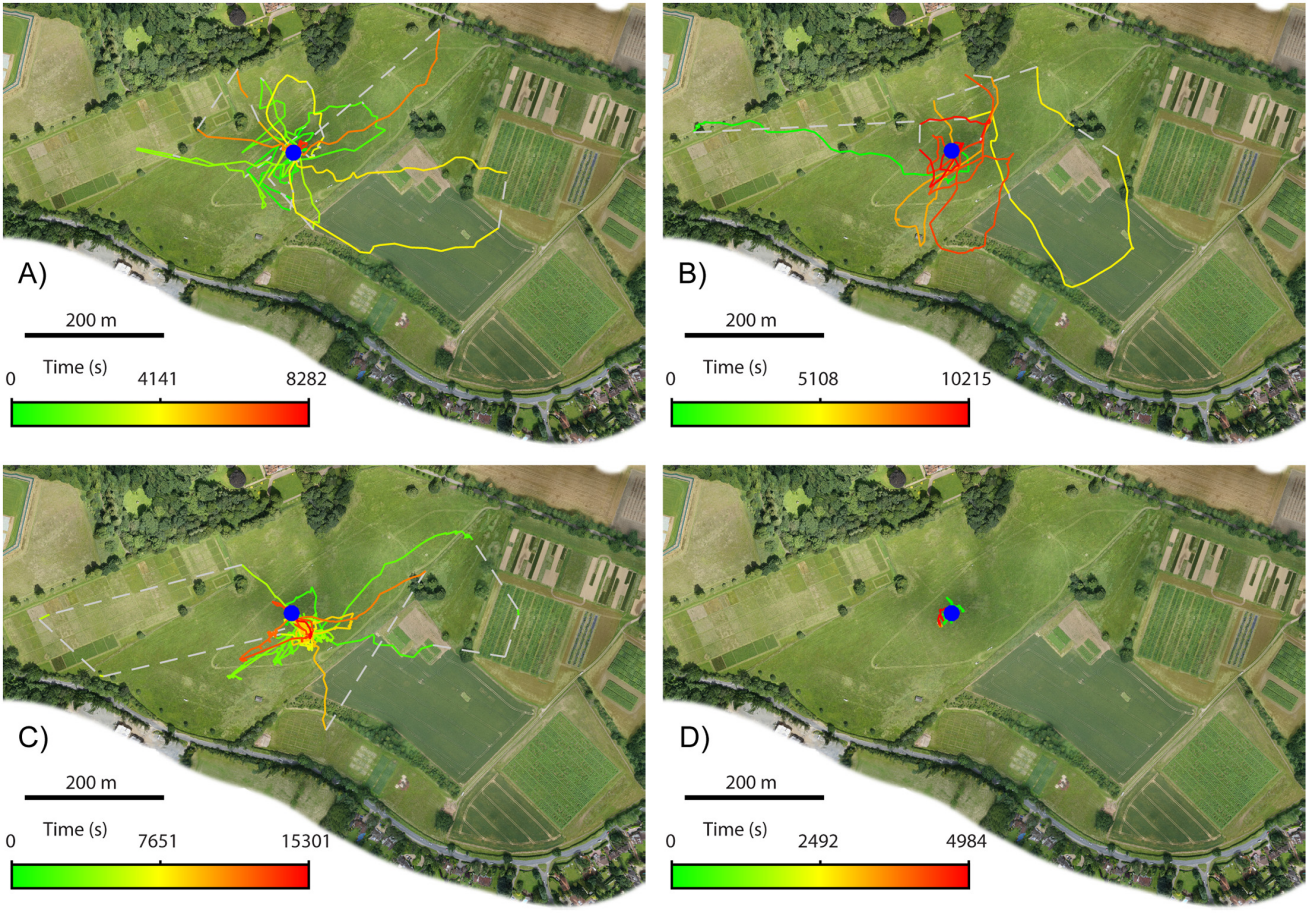
Flight paths of initial exploratory flights of four bees. The position of the nest is marked by a blue circle. Colours represent the time from the start of each flight: initial portion of each flight is in green, changing smoothly through yellow until the end of the flight is shown in red. Grey dashed lines are used to join radar observations made more than 30 s apart, when the bee’s location was uncertain. A-D): flights of Bee 1–4 respectively.

The initial flight of Bee 1 (Fig 2A) lasted 138 minutes and described a pattern typical of the first flights of bumblebees [20], but lasting much longer. Her flight described a number of loops roughly centred on the nest and each in a different direction so that she covered a large area in almost all directions from the nest. She made only one further short flight (13 minutes) on the first day. The following morning she made a 77 minute flight consisting of several loops, mostly in a single direction, toward the corner of the woodland to the West-northwest of the field site. At this time, the main potential sources of forage were brambles and flowering lime trees (*Tilia platyphyllos*) along the edge of the woodland and flowers in the Manor gardens.

Beginning with her next flight (#4, see S5 Fig), almost every flight she made for the remainder of her life fit our definition of exploitation flight: The 11 flights that were not considered exploitation flights all appeared similar to the exploitation flights, with a visit to a known foraging site, but the bee made a shorter second loop before returning to the nest. In these flights she repeatedly flew in the direction of the woodland corner, approximately 270m from the nest, making fast, straight trajectories that were extremely similar both between the outward and return portions of each flight as well as between flights. The portions of her flight furthest from the nest were not detectable by the radar but the flights appear to head toward a clearing at the corner of the woodland. This clearing was thickly overgrown with brambles which were in flower at that time and which attracted large numbers of bees and other pollinating insects. The position of the radar would have allowed us to detect her if her path had continued on the same trajectory into the next field, so it is likely that the clearing was her destination.

Bee 1 continued to visit the same location with remarkably little variation in her flight paths for 78 consecutive flights over the course of five days. After six days of tracking we had to confine her to the nest for two days due to bad weather. On the 9^th^ day of tracking she made only a single flight, although her access to the field was unrestricted, which did not obviously differ from her other flights fitting the definition of exploitation flight.

On the 10^th^ day she made one further flight, similar to her previous exploitation flights, in which she stopped at the corner of the woodland and returned to the nest. Her next flight, however, took an entirely new path, disappearing from radar behind a tree at the boundary between the experimental field and an adjacent field, Park Grass, to the Northwest of the nest, but reappearing from the edge of the woodland to the North-northwest of the nest. From this point on she never repeated her original trajectory but flew toward the edge of the woodland near to Rothamsted Manor, varying between North-northwest and North. The radar tracks typically lost the bee a few meters in front of the woodland, approximately 140 m from the nest, but it is likely that she was feeding on brambles growing at the woodland edge or on lime trees which were flowering along the treeline at that time. We cannot rule out the possibility that she flew over the trees and into the Manor gardens, but on three occasions we detected her with the radar at the woodland edge for 81–319 s, supporting the hypothesis that that was her destination. She continued to visit this new location for a further 69 flights over three days, finally failing to return from an otherwise ordinary looking outbound flight. Of particular note is that a single loop of her initial exploratory flight appears to have taken her to both the locations she later visited in exploitation flights (see Fig 2), and that she never revisited the second location until she began to make exploitation flights there 9 days later, suggesting that location memories can be maintained for at least this long without reinforcement.

#### Bee 2

By the time Bee 2 was released (see Table 1), thistles were in bloom and covered almost the entire study field in dense patches. Her initial flight was comparable in structure to that of Bee 1 (Fig 2B). She made four further flights on her first day of foraging, each of which consisted of a single large loop covering a large area, each in a different direction and each containing multiple stops. By converting the radar coordinates to GPS and using a handheld GPS unit in the field, we were able to make 15 visual observations of Bee 2 visiting natural flower locations over the course of her foraging career, every one of which found her feeding on a thistle.

Starting with the first flight made the following day, Bee 2 made only seven (out of 31) flights that satisfy the description of exploitation flight. Each of these flights initially took a similar direction to the first exploited location of Bee 1, but the radar tracks of most were curtailed when the bee entered the radar-shadow of a tree at the corner of the study field and Park Grass. The most complete track (flight #15, see S172 Fig) shows the bee heading for the woodland edge about halfway along (≈ 210 m from the nest). In addition to these seven flights, Bee 2 visited the same area on a further 16 flights that were not considered exploitation flights as they included several distinct loops. These flights generally covered a larger area and took inconsistent flight paths by comparison to the flights we classified as exploitation flights. The bee was observed feeding on thistles in other locations on multiple occasions during this period and also made a further seven flights in which she did not visit the destination of her exploitation flights. Tracking of Bee 2 terminated at around 2pm on the 6^th^ day of tracking when, after approximately 45 minutes of flight during which she was observed feeding on thistles, she made a fast, straight flight toward the Southeast, disappearing beyond the range of the radar and never returned. There was a severe rain storm that night, which it is likely that the bee did not survive.

#### Bee 3

Like Bee 1, Bee 3 visited two main foraging locations during flights fitting the definition of exploitation flight, but whereas Bee 1 visited only one on each flight and switched between the two, Bee 3 made four flights that included stops in both locations. Interestingly, as with Bee 1, she appears to have discovered both exploitation locations on her first ever flight (see Fig 2C). This initial flight had multiple loops in the manner of those made by the other bees, but is notable for the fact that the bee spent a long time foraging on thistles in a large patch approximately 55 m Southeast of the nest. This was the first of her exploitation locations and the bee was visually observed feeding from thistle flowers in this location both on this flight and a number of subsequent flights. Although the patch was rather large (3800 m^2^) and appeared uniform in thistle age and density, the bee concentrated exclusively on the North-east corner. Her second flight involved several loops all of which took her back to the same thistle patch and was followed by two exploratory flights to other parts of the field. Following this, high wind and heavy rainfall made it impossible to run the radar for 5 consecutive days, during which Bee 3 was confined to the nest. When released for the second day of tracking she made two exploratory flights both of which covered large areas of the field, but both of which included stops at the favoured thistle patch.

The first flight on the third day of tracking was the first flight by Bee 3 that met our definition of an exploitation flight: on leaving the nest she stopped at the thistle patch she regularly visited, but then flew across a low hedge between fields to a spot South-southeast of the nest in another fallow field in which scattered thistles were the main potential source of forage (≈ 190 m from the nest). This would become her second exploitation location. The following two flights were also defined as exploitation flights but were anomalous in that, unlike every other exploitation flight we observed, they included detours to other, non-revisited, parts of the field, in addition to stops at the exploitation location in the thistle patch. These were the only flights made by this bee in which she stopped at the thistle patch but not the second exploitation location. Beginning the following morning, Bee 3 made 17 more flights over five days, all but two of them fitting the definition of exploitation flight and including a stop at the second exploitation location in the next field. Three of these flights also involved a stop at the first exploitation location during the outbound portion of the trip. All, including those with two destinations, were characterised by fast, straight flights with no detours, although the outbound and return trips appeared less similar to one another than was observed in Bee 1, the outward trips always passing over a low hedge to get to the foraging site, while the return trips often skirted around the end of the hedge.

#### Bee 4

At the time Bee 4 was recorded, the majority of the thistles in the field site had gone to seed. Around 10% of thistles were still flowering, scattered randomly among the seed heads. The Park Grass field to the Northwest had some scattered flowers, mainly dandelion. The initial flight of Bee 4 was unusual (Fig 2D): after initially circling the nest a number of times, as is commonly observed in an orientation flight [20], she landed on a path of thistles just 18 m West of the nest where she remained for approximately 75 minutes and was observed sitting on a dead thistle head without moving or feeding. The following seven flights, over the course of two days, took the form of one or two loops, each flight heading in a different direction so that taken, as a whole, the bee explored the field site over a series of flights to a similar degree to which the other bees had explored it during a single exploratory flight.

Beginning in mid-afternoon of the second day of tracking, Bee 4 made a series of nine flights that we classified as exploitation flights, heading directly Southeast from the nest. These flights took her further away from the nest than the other bees ever travelled and the end-point remains unknown since she travelled beyond the range of the radar in most of them. There was no available forage at the location at which we typically lost the signal and an observer in the field was unable to find the bee, suggesting that she had continued flying past the point where we lost her signal. Two flights recorded her crossing the road that forms the boundary of Rothamsted Research, losing the signal in the neighbouring residential area, ≈ 575 m from the nest (flights #17 and #19, see S231 and S233 Figs). We consider it likely that she was foraging in one or more domestic gardens. Unlike Bees 1 and 3, which never returned to exploratory flights after commencing exploitation, this series of nine flights was interrupted by two exploration flights consisting of loops within the main experimental field.

On the fourth day of tracking, Bee 4 made another exploration flight which included a stop in Park Grass (≈ 240 m from the nest). Over the next four days she made four flights to the same location that met the definition of exploitation flight, interspersed with another four explorations that included a stop in Park Grass and two that did not. Of particular interest, one flight (#27, see S241 Fig) included visits to both exploitation locations, which were approximately 800 m apart; first visiting Park Grass and then, in a separate loop, flying in the direction of the residential area she visited on her initial exploitation flights. Her final flight took her in an unfamiliar direction, from which she never returned.

### Exploitation vs exploration

**Fig 3:**
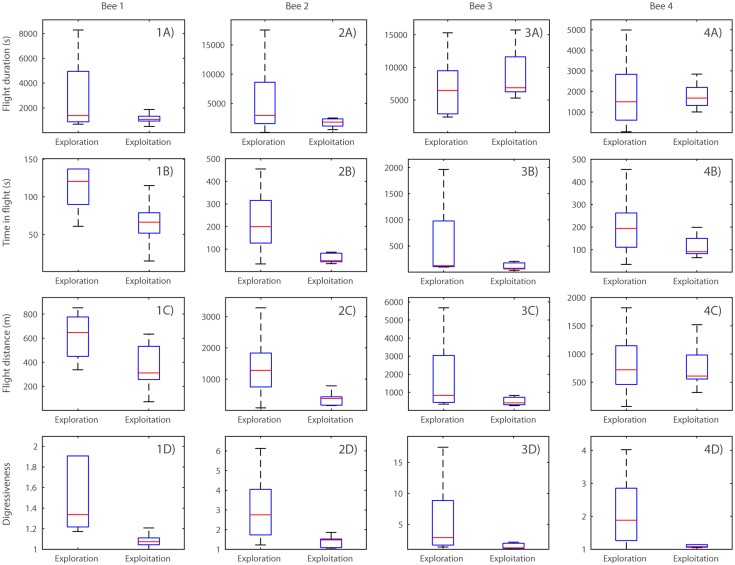
Comparison of flight characteristics of exploitation and exploration flights. Boxes show median and interquartile range, and whiskers represent the range of the data. Outliers have been removed for clarity (outliers defined as data-points lying more than 1.5 times the interquartile range outside the quartiles). 1-4A) flight duration of exploration and exploitation flights for Bee 1–4 respectively; 1-4B) time in flight; 1-4C) flight distance; 1-4D) digressiveness scores. Note that the ordinates differ in scale due to high levels of inter-individual variation.

We used GLMMs to compare flight characteristics of flights categorised as exploration and exploitation flights (Fig 3). These were the total duration of each flight, from leaving the nest to returning; the time spent actually in flight; the total distance flown; and *digressiveness*, a way of quantifying how much greater the distance actually flown was than an efficient straight-line path to the same destination (see Methods). There were significant differences between individual bees in their flight duration, time in flight, flight distance and digressiveness (all P < 0.01), but there were also differences between the two flight categories: The time in flight was significantly smaller in flights classified as exploitation flights (t_242_ = -5.53, P < 0.01) although there was no difference in total flight duration (t_242_ = -0.81, P = 0.42). Exploitation flights were shorter (t_242_ = 2.25, P = 0.03) and had a lower digressiveness score (t_242_ = -7.57, P < 0.01).

Two of our bees (Bee 1 and Bee 4) switched the destination of their exploitation flights and subsequently never returned to exploiting the original location. Comparison of the exploitation flights to each of the two locations for each bee revealed that the distance flown was lower after switching sites (t_156_ = -2.04, P = 0.04; Fig 41C and 42C). Digressiveness was higher in flights to the second exploitation locations (t_156_ = 2.20, P = 0.03; Fig 41D and 42D). There was no significant difference between the duration of exploitation flights visiting the two locations (t_156_ = 0.50, P = 0.62), although the flight time was reduced in flights to the second exploitation locations (t_156_ = -2.16, P = 0.03). There were significant difference between the two bees in flight duration (t_156_ = -49.40, P < 0.01), time in flight (t_156_ = 23.45, P < 0.01) and flight distance (t_156_ = 80.06, P < 0.01), but not in digressiveness (t_156_ = 0.60, P = 0.55).

**Fig 4:**
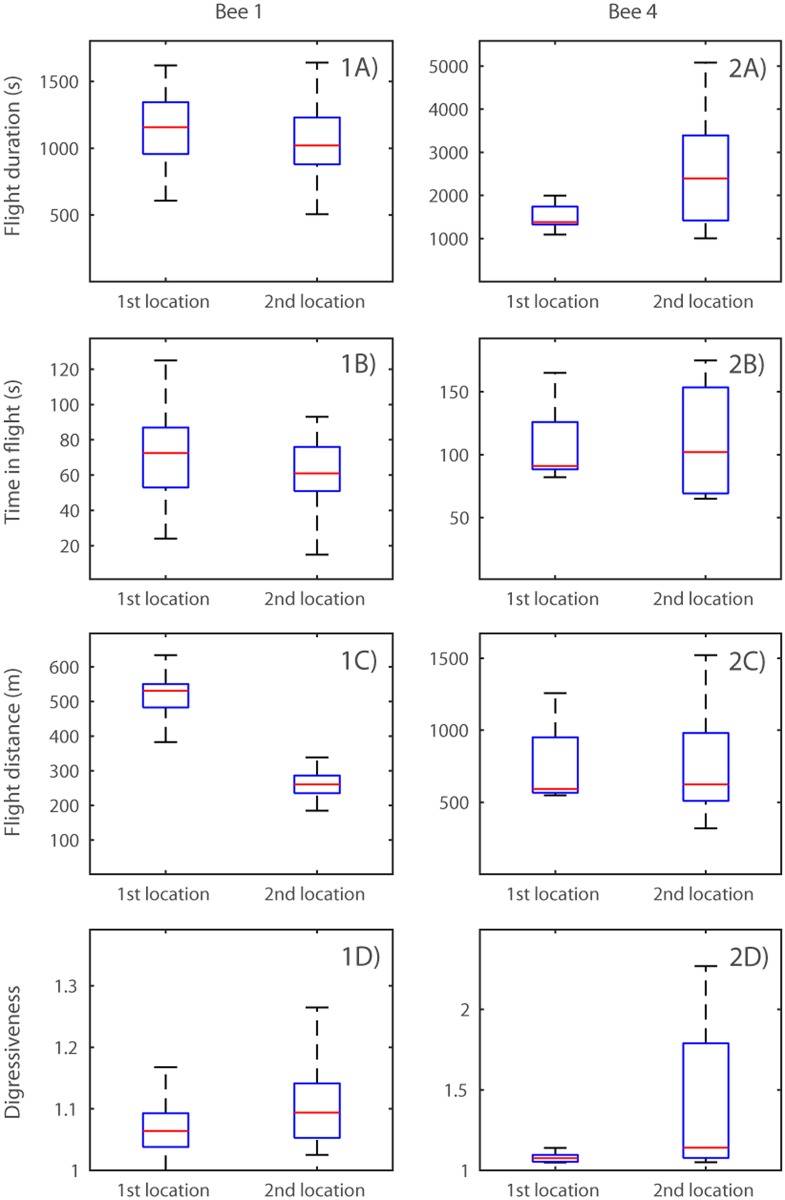
Comparison of characteristics of exploitation flights made to the first or second destination exploited by Bees 1 and 4. Boxes show median and interquartile range, and whiskers represent the range of the data. Outliers have been removed for clarity (outliers defined as data points lying more than 1.5 times the interquartile range outside the quartiles). 1-2A) Flight duration of exploration visits to the 1^st^ and 2^nd^ location exploited by Bees 1 and 4, respectively; 1-2B) time in flight; 1-2C) flight distance; 1-2D) digressiveness score. Note that the ordinates differ in scale due to high levels of inter-individual variation.

Thus, the flights we classified as exploitation flights differ systematically from all other flights in a variety of flight characteristics which are not a necessary consequence of the definition of exploitation but suggest a difference in function. When bees switched the destination of their exploitation flights, the only apparent difference in flight characteristics was a reduction in the distance flown in flights visiting the second location.

### Field observations

We were able in some circumstances to convert the radar position of a stationary bee into GPS coordinates which an observer used to look for the focal bee in the field. We were not able to make visual observations of Bee 1 outside the nest. Relatively frequent observations were made of Bees 2 and 3, and Bee 4 was observed twice. In every case, the bees were found on thistles and, with the single exception of the first flight of Bee 4, they were nectar feeding during every observation.

The radar data suggest that all of our focal bees returned to particular forage patches repeatedly during their exploitation flights. Visual observation of Bee 3 at her first exploitation location confirmed that she returned frequently to the same part of one particular large thistle patch, where she was observed feeding. However, the spatial resolution of the radar data is not fine enough to address the question of whether they return to individual plants or flowers. We used individually numbered stakes to mark every thistle plant on which any of our focal bees was observed to feed, marking 88 in total over 29 foraging events by three bees. No bee was ever observed to return to a plant on which it had previously fed, although we cannot rule out the possibility that they did so on other occasions.

It was very common for the bees to move from plant to plant within individual thistle patches, frequently moving to the nearest neighbours of the plant they were on, interspersed with occasional flights of 5–10 m to other parts of the same flower patch. We observed a mean of 3 thistles fed from in each thistle patch, but this is certainly an underestimate since it took several minutes for an observer to arrive at a patch after the bee stopped flying. We never observed any of our focal bees foraging for pollen.

## Discussion

In this study we have followed the life-long flight activity of bumblebee foragers (S2-245 Figs), in sufficient temporal and spatial resolution to examine foraging movements at a local scale. We have gathered a wealth of data on the movements of individual bees and shed light on several important aspects of their foraging behaviour, including quantifying the relative frequency of flights fitting our definitions of exploration and exploitation flights, examining when forage sites are first discovered and illuminating the high degree of inter-individual variation in foraging behaviour. Although we did not directly quantify foraging behaviour, analysis of our data on spatial movement can help to illuminate how exploitation is balanced with the need to explore the landscape for potential food sources.

### Exploration vs. exploitation flight

Osborne et al. compared the flight characteristics of flights recorded from bees with differing levels of experience [20]. They found that the first flights of inexperienced bumblebees were looping and digressive, while later flights were straighter [20]. It is not possible to determine from their data how the flight structures developed between the two extremes, nor whether digressive, presumably exploratory flights ever occurred later in life. In order to examine the ontogeny of exploratory and exploitatory flights, we sorted the flights in our dataset into two categories we called *exploration* and *exploitation*. These categories do not stem from any description of the flight characteristics *per se*, nor do they depend on timing or on the experience of the bees, rather they arise from an attempt to identify flights with a shared primary function. Specifically, our definition of exploitation flight contains what seems to us to be the minimum necessary criteria to identify flights that are likely to serve the sole purpose of foraging at known locations, without presupposing anything about the form or structure of such flights. Exploitation flights were defined as any flights with a stop in at least one previously visited location, and which consisted of a single loop. By single loop, we mean that we excluded flights in which the bee returned to the vicinity of the nest but flew out again, since this behaviour strongly indicates to us a flight with multiple purposes. The category *exploration flight* simply contains any flight that did not stop at any previously visited locations *or* that involved multiple loops. Thus flights that were categorised as exploration may not have been wholly exploratory in function. In practice, though, as will be seen below, the majority of these flights had characteristics suggesting the bees were indeed exploring the environment. A large proportion of flights, from 23–91% of the flights made by each bee, met our definition of exploitation flight. This is strongly suggestive that a large part of each bee’s behavioural repertoire consists of flights whose function is to return to previously known locations, presumably for the purpose of foraging. Observations of Bee 3 at her first foraging location confirmed that she was feeding from thistles during stops at this location. We did not observe any of the other bees at the end-points of their exploitation flights, so could not confirm that they were foraging, although we did observe two of them feeding during stops on their initial exploratory flights, providing some evidence that foraging (rather than just spatial memory acquisition) was an important motivator behind their flight behaviour. The fact that bees were observed feeding on both exploration and exploitation flights demonstrates that food collection is not strictly confined to a particular category of flight.

Because our flight categories are not defined by the timing or path structure of the flights there is no reason *a priori* to expect that flights falling into the two categories would vary systematically from each other. For example, there is no reason why flights to previously unvisited locations should be digressive, or why exploitation flights, with their visits to memorised locations, should not also contain looping digressions to explore other areas. In practice, however, the flights we classified as exploitation flights differed significantly from exploration flights in several ways. This strongly suggests that our category definitions are not arbitrary, but have captured some real variation present between flights with different function. Exploitation flights were more direct and efficient than those classified as explorations, with a low score for digressiveness. This can be seen clearly in the tracks of individual flights (see supplementary material, S2-S245 Figs). Only two exploitation flights by Bee 3 (#8–9, S196-S197 Figs) combined a visit to a learned location with flight to other parts of the field that were not on a direct route to and from the nest. Our bees often stopped and fed on flowers during their exploration flights, as confirmed by field observation, but our data suggest it is rare for foraging bees to make trips with the dual purpose of exploiting learned food sources and exploring unfamiliar areas. Exploration of the environment and exploitation of already discovered patches appear to be separate functions, accomplished on separate occasions. Interestingly, in every flight where a bee stopped both at a habitual location and in less familiar places, she visited the habitual location first. This might suggest a lower-than-expected reward at the accustomed site is what triggers further exploration of the landscape by experienced bees, although confirming this would require detailed measurement of the food availability at different sites which was beyond the scope of this study.

The flights fitting our definition of exploitation flight covered a shorter distance than other flights, which can be explained by their lower digressiveness: it seems that the destinations visited during exploitation flights are just as far away as the furthest distances reached during exploration, but that by flying straight paths towards their goal, the actual distance travelled by each is reduced. The bees also spent less time in flight during exploitation flights. However the total bout duration (the time that the bee spent out of the nest), was not lower in exploitation flights. This discrepancy can be explained by the fact that the time in flight accounted for less than 5% of the duration of a typical foraging bout. It is likely that almost all the time spent out of the nest is spent actually feeding and that this determines the length of a foraging bout. Thus, bees exploit known resources by developing and following efficient routes which are likely to reduce the energetic costs of foraging, so may increase their foraging efficiency (the ratio of energetic gain to cost [25,26]). Whether they also increase the net rate at which they provision the nest depends on whether they return from exploitation flights with greater nectar loads than they do from explorations, which should be the subject of further study.

### Balancing exploration with exploitation

Osborne et al. noted that the flights of more experienced foragers were longer, straighter and less looping than their initial flights [20], but did not define or quantify a difference between exploration and exploitation flight. In many respects the flight characteristics of the flights we categorised as exploration and exploitation flights match the descriptions given by Osborne et al. of preliminary and later flights of their bees, respectively [20]. However, our analysis of the full sequence of flights by identified foragers suggests that rather than a gradual change in flight characteristics over time, the difference between early and later behaviour is caused by a change in the frequency of the two flight types: more experienced bees primarily perform exploitation flights while the initial few flights always conform to our definition of exploration flights.

Because no prior study has been able to follow the entire foraging career of individual bees, it has never previously been possible to pinpoint when a bee first discovered a location that later became an important forage source. All bees in our study started their foraging career with several exploration flights and our data suggest it is during these flights that they discover most or all of the sites that they will return to for the rest of their lives. Bees 1 and 3 seem to have discovered both of their locations during their initial exploratory flight; Bee 4 visited her second exploitation location, in Park Grass, on her third exploration (seven days before she visited it as part of an exploitation flight), although we have no evidence that she had prior knowledge of her first location before her first exploitation flight; it is not clear from the radar tracks exactly where Bee 2 was foraging during her exploitation flights, so we cannot determine when she discovered that location.

Our data on the preliminary flights of our bees suggest that there may be a greater range of exploration strategies in use by bumblebees than previously thought: Bees 1 and 3 explored their environment with a small number of arcing, looping flights, similar to those described by Osborne et al. [20], but the preliminary flights of Bees 2 and 4 took the form of a greater number of individual flights, each describing a single loop. Each flight headed in different directions, such that between them they covered most of the area around the hive. This pattern has something in common with the way Capaldi et al. suggested that inexperienced honeybees familiarise themselves with the landscape [19]. It is not clear whether these apparently different ways of exploring the area led to differences in how thoroughly or efficiently the bees searched for available food sources.

There does not seem to be a clear temporal break between exploration and exploitation behaviour—all bees interspersed exploration flights with those we classified as exploitation flights—but exploration flights were rare after the onset of exploitation. Bee 4 made three flights that resemble the initial explorations, with looping paths covering large areas in less familiar parts of the field. As noted above, the availability of bramble and thistle was much reduced at the time Bee 4 was recorded and her initial exploitation location was much further from the nest than those favoured by the other bees. Although our data cannot determine what caused this bee to change its behaviour, one intriguing possibility may be that the familiar foraging location experienced a change in profitability, causing the bee to abandon the location in search of better forage conditions. Bee 2 interspersed flights fitting the definition of exploitation flight with others classified as exploration flights, visiting other parts of the field throughout its life. One hypothesis that may be worthy of future investigation is that a lack of suitable foraging locations motivated these bees to continue searching for new food sources when other bees had switched to an exploitation phase.

It is clear that exploration of the environment does not necessarily end with the initial orientation flights and that there is a wide range of variation in how individual bees allocate resources between exploiting known food supplies and exploring. Further work will be required to determine whether the amount of additional exploration is dependent on the quality and quantity of available resources or whether it represents inter-individual differences in foraging strategy.

### Foraging sites

All our bees apart from Bee 2 visited locations, likely to be forage patches, to which they were faithful for up to six days at a time. Each exploitation flight appeared to have only a single destination, with the exception of four flights by Bee 3. In most cases we did not have radar coverage of the places where feeding actually took place but the return tracks originated close to the end of the outbound journeys, suggesting that the bees were not performing a trapline between multiple distant patches of forage. Prior to the advent of harmonic radar, studies of the foraging patterns of bumblebees and honeybees within floral patches demonstrated that individual bees repeatedly visited a single foraging location, and it was inferred that foraging bees restrict their activity to a single patch in the environment [27–29]. The earlier results of Osborne et al. suggested that the majority of flights they recorded took in only a single foraging destination [21] and our continuous, life-long monitoring confirms this. Nearly all work on traplining has been done over small spatial scales equivalent to movement within rather than between patches ([30,31], but see [22]). It is possible that our bees followed traplines within their chosen foraging patches (or between proximate patches within a few meters distance), although our visual observations did not reveal a single instance of a bee returning to an individual plant it had previously fed from. Manning found that when foraging on Hound’s tongue flowers less than about 2 m of each other, bumblebees moved between plants in an ‘irregular fashion’, while once plants were around 4 m away the bees formed a distinct route between plants [28]. Our visual observations suggested that most plant-to-plant transitions within patches of thistle involved flights of less than 2 m.

The spatial movement patterns of pollinators have important implications for plant gene flow throughout the landscape [32] as well as the spread of insect pathogens [33]. The movement patterns of the bumblebee foragers we tracked in our study suggest that patch-exploiting bumblebee foragers provide a pollination service within spatially restricted patches of flowers. However, it is not likely that they provide frequent mixing of pollen between discrete patches of flowers at larger spatial scales, because the evidence suggests they exploit only a single forage patch at a time. In contrast, during exploratory flights all foragers appeared to land, and feed, at multiple flower patches, potentially allowing for the dispersal of pollen over a wider range (mean ± s.d. area covered by exploration flights: 24000 m^2^ ± 32000 m^2^), but is likely that only a tiny fraction of flowers are pollinated during these exploratory events.

As described above, it was only very rarely that flights we classified as exploration flights were interspersed with exploitations. There was no switching back and forth between foraging destinations. However, over the course of their lifetimes, two of our bees (1 and 4) made an abrupt switch of destinations, after several days of constancy. Interestingly, in the case of Bee 1, she had visited this second destination only once, on her initial exploratory flight nine days earlier. This suggests that forager bumblebees must be able to memorise potential foraging sites over at least that period, and that further exploration is not required in order to forage based on these memories. Bee 4 preceded her switch with an exploration flight that involved a stop at what became her second destination, but interestingly she had already visited that location during exploration flights several days previously, so might also have been acting on a stored memory of an alternative potential site.

Analysis of the characteristics of flights to each of the two destinations visited by each bee reveals that the second destinations were closer to the nest and that the bees’ flights to them were shorter and involved less flying time, although the total duration of flights did not decrease. Was reduction of the flight distance the motivation behind switching? The bumblebees’ condition may have declined with cumulative flight activity, so reducing the distance flown per trip may allow them to prolong their working life. Bumblebees with high levels of wing wear are more likely to forage in high density floral resources, and spend less time in flight [34]. Another potential reason for switching may be that the original patches declined in quality due to flowers dying or increased competition. Heinrich demonstrated that the time required to gather a nectar load increased dramatically when there was heavy competition [24]. In that study, bees were restricted to an enclosure and responded to increased competition by becoming less specialised on certain plants [24]. Under natural conditions they may respond to competition by changing their foraging destination. We were not able to make observations of the nectar availability or level of competition in any of the foraging locations in question. Future work would benefit from addressing the ways in which different patches differ in quality and how this changes with time, along with the physical condition of workers.

### Inter-individual variation

One of the most striking results to emerge from these data is the large degree to which our bees differed from one another. Over 90% of the flights of Bee 1 fit our definition of exploitation flights, a much higher proportion than any of the other bees. In the case of Bees 2 and 3, this is partly explained by the fact that they spent longer exploring before beginning their exploitations. Bees 1 and 3 made few further exploration flights after switching to exploitation whereas Bees 2 and 4 interspersed continued explorations among the flights we categorised as exploitation. Bees 2 and 3 made exploitation flights to only one destination (although both were observed to feed in a variety of other locations during flights that could not be categorised as exploitation), whereas Bees 1 and 4 each switched the destination of the flights we classified as exploitation flights over the course of their foraging career, neither ever returning to their first destination afterwards.

Bee 1 made an average of 15 flights per day, while the other three bees only averaged 3–5 flights per day. Similarly large variations were seen in the duration of flights, the latency between flights and the distance travelled in each flight (see Table 1). The flights of Bees 2 and 3 lasted longer and covered a greater distance (see Table 1): it seems likely that they took longer to acquire a full honey stomach and return to the nest, so could not undertake so many trips. Bee 4, by contrast, took no longer to complete a flight than Bee 1; in her case the difference in flight frequency is caused by the fact that she spent longer in the nest between flights. These differences are likely to translate into a large difference in the rate at which they can provision the nest.

Since each bee was from a different colony and each individual was tested in a different floral environment (because they had to be tested sequentially) we cannot be certain whether the heterogeneity of behavioural strategies was a result of heritable (colony) variation or the result of resource distribution (or a combination of both). A number of uncontrollable factors may have changed between the times we tracked each bee. The most important such factor is likely to be seasonal variation in what sources of forage were available. No two bees visited the same areas of the landscape on their exploitation flights (S1 Fig), which may be partially explained by changes in where flowers were in bloom over the course of the experiment. Bee 1 probably fed largely or exclusively on bramble; there were few brambles in flower when the other bees were recorded, but large numbers of thistles dominated the site while Bees 2 and 3 were recorded; Bee 4 had far fewer flowers available near the nest, none of which occurred in dense patches like the brambles and thistles. Other differences cannot be explained by variation in forage availability: Bees 2 and 3 foraged on thistle in roughly the same area, but Bee 2 made only about a third as many exploitation flights as Bee 3, and spent five times as long in the nest between flights.

Changing weather conditions might also account for some of the variation between bees. For example, Comba observed that the number of bumblebees visiting a forage patch was correlated with temperature [35]. Movements between plants within a patch appeared to be affected by wind speed and direction, although bumblebees are able to maintain accurate headings despite strong winds [36], so wind may not influence the choice of patches. Examination of the flight paths (see S2-S245 Figs), suggest the individual bees had recognisable ‘styles’ over the course of up to several weeks, despite changeable weather during these periods, but it remains possible that on a larger timescale, seasonal variations in weather conditions may account for some inter-individual variation.

Other potential sources of variation include the age and size of the bees, and the amount of competition they faced in exploring floral resources. We did not know the age of our bees when we began recording. We were able to track three of our focal bees for roughly two weeks apiece (and Bees 1 and 3 disappeared on otherwise typical seeming exploration bouts suggesting they might have been predated and would otherwise have continued to forage), but Bee 2 lasted only 6 days before flying away from the nest in a novel direction from which she never returned. It is possible that this bee was aged or sick, which might account for her unusual behaviour.

Although it is expected that randomly chosen individuals will tend to show variation in behaviour [37–39], the extent of the inter-individual differences we observed in flight behaviour is dramatic. These differences appear to persist over the bees’ entire foraging career, and are likely to lead to high levels of variation in the contribution different foragers make to provisioning the colony. The extreme time demands of undertaking life-long tracking demands a trade-off: observing individuals in greater detail than previous studies meant we were only able to follow a small number of individuals. In the absence of far larger sample sizes, it is impossible to quantify the distribution of different flight behaviours or to know how common the types of flight behaviour we have observed are. Equally, the requirement of tracking bees sequentially introduced a number of uncontrollable variables—variation between colonies, variation in the amount of competition faced, differences in weather conditions, floral resources and season—which might have played a part in determining foraging strategies and behaviour. Nonetheless, our data demonstrate the extent of variation that can exist between individual foragers, although not its distribution. Do the majority of foragers efficiently provision the nest like Bee 1, with only rare exceptions? And how common are foragers that resemble Bee 2, spending most of their time exploring for previously unvisited flower patches but rarely returning with resources for the nest? Future advances in tracking technology, allowing us to track multiple individuals at the same time, may provide the answers.

## Supporting Information

S1 DatasetData describing every flight made by four bees.These are the data extracted from the raw tracking files and used in the analyses in this manuscript. The data are presented in an Excel spreadsheet with one row for every flight we recorded. The columns give the following information: *Bee* gives the number code used to refer to the bee that made each flight; *Flight no* gives the order each flight was made; *Date* is the date on which each flight was recorded; *Time* is the time that the bee left the nest for each flight; *Flight duration* is the total duration of the flight, in seconds, from leaving to re-entering the nest; *Time in flight* is the time that the bee could be shown to be actually flying, in seconds; *Flight distance* is the total distance flown during the flight, in meters; *Maximum distance* is the greatest distance from the nest, in meters, reached during the flight; *Digressiveness* is the digressiveness score for the flight, defined as $\frac{Flight\;distance}{2*Maximum\;distance}$; *Exp* is a code describing whether each flight was categorised as exploration (0) or exploitation (1).(XLSX)

S1 Fig Flight paths of every exploitation flight made by four bees. The position of the nest is marked by a blue circle. Each panel shows every flight of a single bee that met the definition of an exploitation flight. Each individual flight is shown in a different colour, the earliest flights undertaken by each bee in green, changing smoothly through yellow until the last exploitation flights in each bee’s life are shown in red. Grey dashed lines are used to join radar observations made more than 30 s apart, when the bee’s location was uncertain. A-D): flights of Bee 1–4 respectively.
(TIF)

S1 File S2-S40 Figs: Flight paths of every flight made by Bee 1, part 1. All flights made by Bee 1 during the period 25/06/2015–27/06/2015. Each figure represents a single flight. Bee ID, flight number, date of recording and the duration of each flight are shown in the bottom left corner of each figure. The position of the nest is marked by a blue circle. Colours represent the time from the start of each flight: initial portion of each flight is in green, changing smoothly through yellow until the end of the flight is shown in red. Grey dashed lines are used to join radar observations made more than 30 s apart, when the bee’s location was uncertain.
(ZIP)

S2 File S41-S84 Figs: Flight paths of every flight made by Bee 1, part 2. All flights made by Bee 1 during the period 28/06/2015–03/07/2015. Each figure represents a single flight. Bee ID, flight number, date of recording and the duration of each flight are shown in the bottom left corner of each figure. The position of the nest is marked by a blue circle. Colours represent the time from the start of each flight: initial portion of each flight is in green, changing smoothly through yellow until the end of the flight is shown in red. Grey dashed lines are used to join radar observations made more than 30 s apart, when the bee’s location was uncertain.
(ZIP)

S3 File S85-S109 Figs: Flight paths of every flight made by Bee 1, part 3. All flights made by Bee 1 on 04/07/2015. Each figure represents a single flight. Bee ID, flight number, date of recording and the duration of each flight are shown in the bottom left corner of each figure. The position of the nest is marked by a blue circle. Colours represent the time from the start of each flight: initial portion of each flight is in green, changing smoothly through yellow until the end of the flight is shown in red. Grey dashed lines are used to join radar observations made more than 30 s apart, when the bee’s location was uncertain.
(ZIP)

S4 File S110-S157 Figs: Flight paths of every flight made by Bee 1, part 4. All flights made by Bee 1 during the period 05/07/15–06/07/15. Each figure represents a single flight. Bee ID, flight number, date of recording and the duration of each flight are shown in the bottom left corner of each figure. The position of the nest is marked by a blue circle. Colours represent the time from the start of each flight: initial portion of each flight is in green, changing smoothly through yellow until the end of the flight is shown in red. Grey dashed lines are used to join radar observations made more than 30 s apart, when the bee’s location was uncertain.
(ZIP)

S5 File S158-S188 Figs: Flight paths of every flight made by Bee 2. All flights made by Bee 2 during the period 11/07/15–16/07/15. Each figure represents a single flight. Bee ID, flight number, date of recording and the duration of each flight are shown in the bottom left corner of each figure. The position of the nest is marked by a blue circle. Colours represent the time from the start of each flight: initial portion of each flight is in green, changing smoothly through yellow until the end of the flight is shown in red. Grey dashed lines are used to join radar observations made more than 30 s apart, when the bee’s location was uncertain.
(ZIP)

S6 File S189-S214 Figs: Flight paths of every flight made by Bee 3. All flights made by Bee 3 during the period 23/07/15–06/08/15. Each figure represents a single flight. Bee ID, flight number, date of recording and the duration of each flight are shown in the bottom left corner of each figure. The position of the nest is marked by a blue circle. Colours represent the time from the start of each flight: initial portion of each flight is in green, changing smoothly through yellow until the end of the flight is shown in red. Grey dashed lines are used to join radar observations made more than 30 s apart, when the bee’s location was uncertain.
(ZIP)

S7 File S215-S245 Figs: Flight paths of every flight made by Bee 4. All flights made by Bee 4 during the period 21/08/15–03/09/15. Each figure represents a single flight. Bee ID, flight number, date of recording and the duration of each flight are shown in the bottom left corner of each figure. The position of the nest is marked by a blue circle. Colours represent the time from the start of each flight: initial portion of each flight is in green, changing smoothly through yellow until the end of the flight is shown in red. Grey dashed lines are used to join radar observations made more than 30 s apart, when the bee’s location was uncertain.
(ZIP)
