# Supplementary figures and images for: Life-Long Radar Tracking of Bumblebees (part 1 of 3)
Source: PLoS One. 2016 Aug 4;11(8):e0160333. doi: 10.1371/journal.pone.0160333 (PMC4973990; doi:10.1371/journal.pone.0160333)

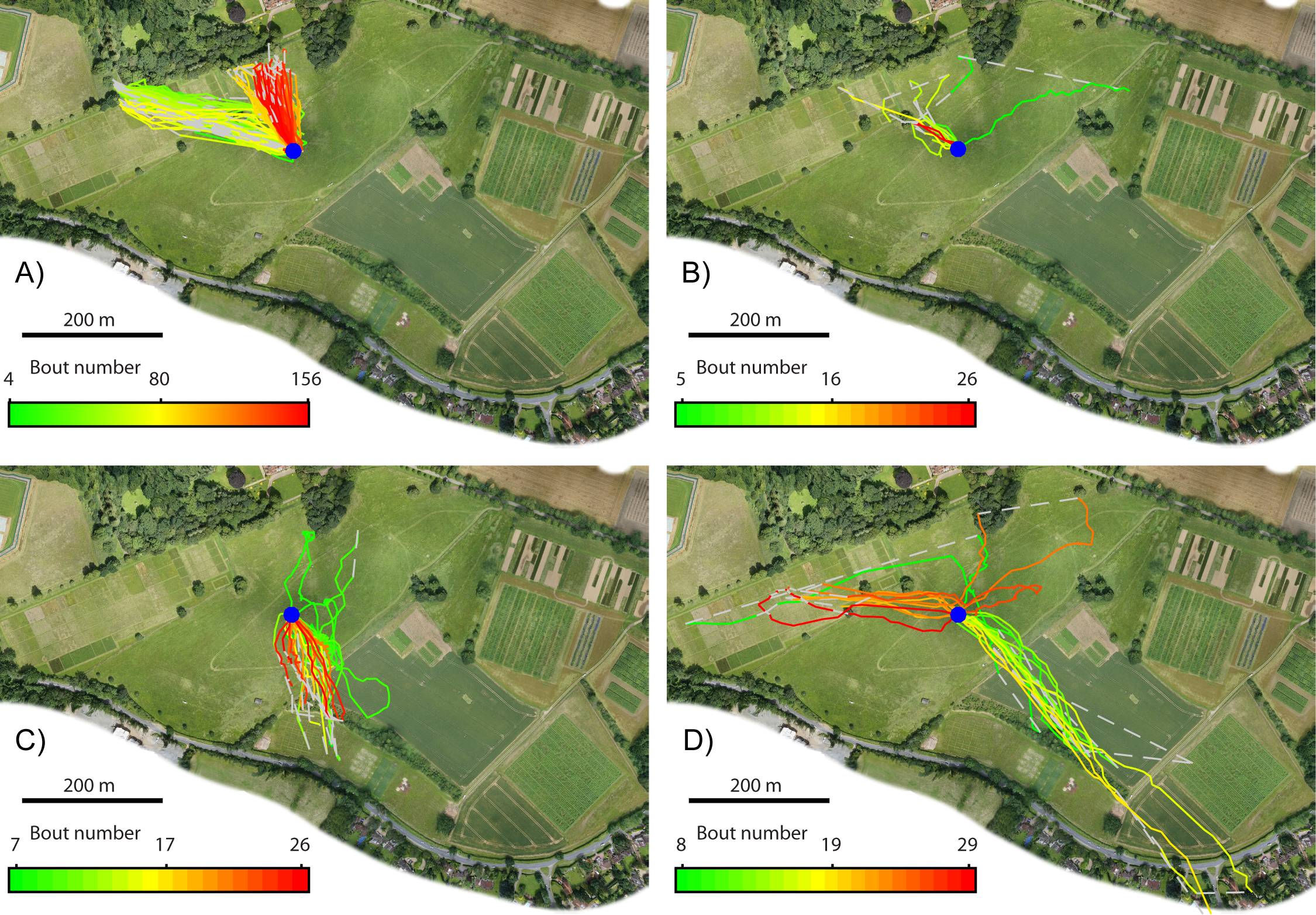

Supplement: S1 Fig — The position of the nest is marked by a blue circle. Each panel shows every flight of a single bee that met the definition of an exploitation flight. Each individual flight is shown in a different colour, the earliest flights undertaken by each bee in green, changing smoothly through yellow until the last exploitation flights in each bee’s life are shown in red. Grey dashed lines are used to join radar observations made more than 30 s apart, when the bee’s location was uncertain. A-D): flights of Bee 1–4 respectively. (TIF) [file pone.0160333.s002.tif]

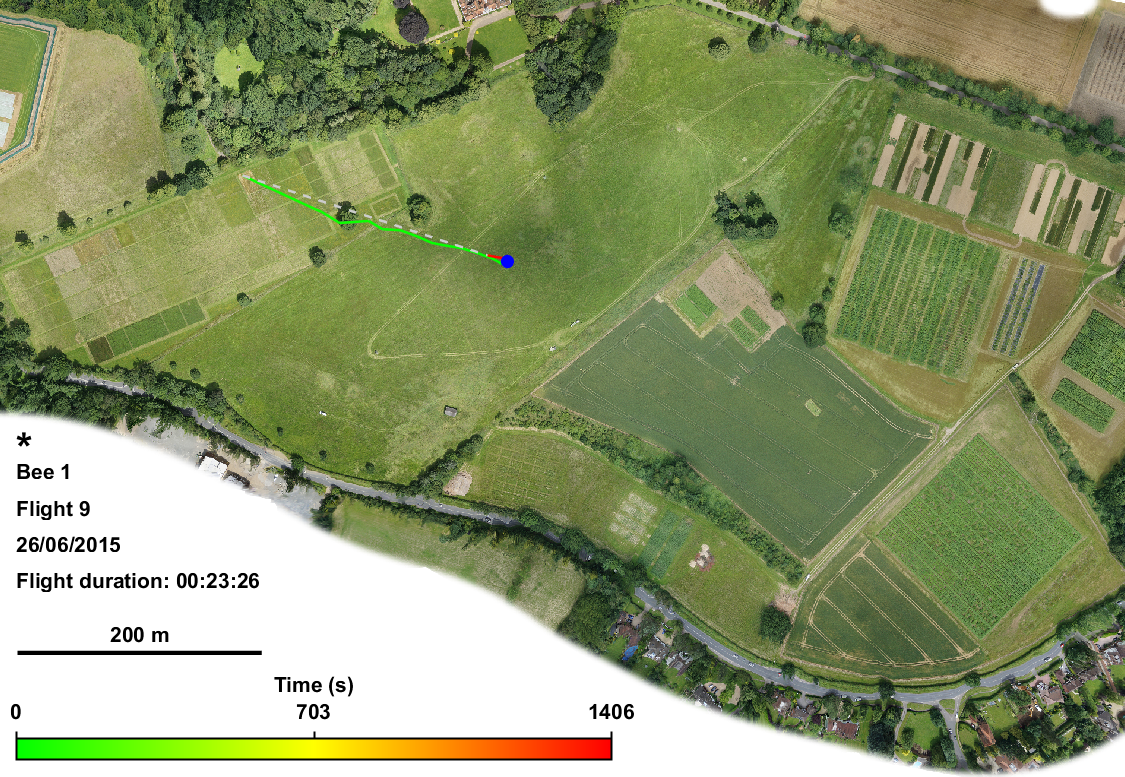

Supplement: S1 File — All flights made by Bee 1 during the period 25/06/2015–27/06/2015. Each figure represents a single flight. Bee ID, flight number, date of recording and the duration of each flight are shown in the bottom left corner of each figure. The position of the nest is marked by a blue circle. Colours represent the time from the start of each flight: initial portion of each flight is in green, changing smoothly through yellow until the end of the flight is shown in red. Grey dashed lines are used to join radar observations made more than 30 s apart, when the bee’s location was uncertain. (ZIP) [file pone.0160333.s003.zip › S10 Fig.tif]

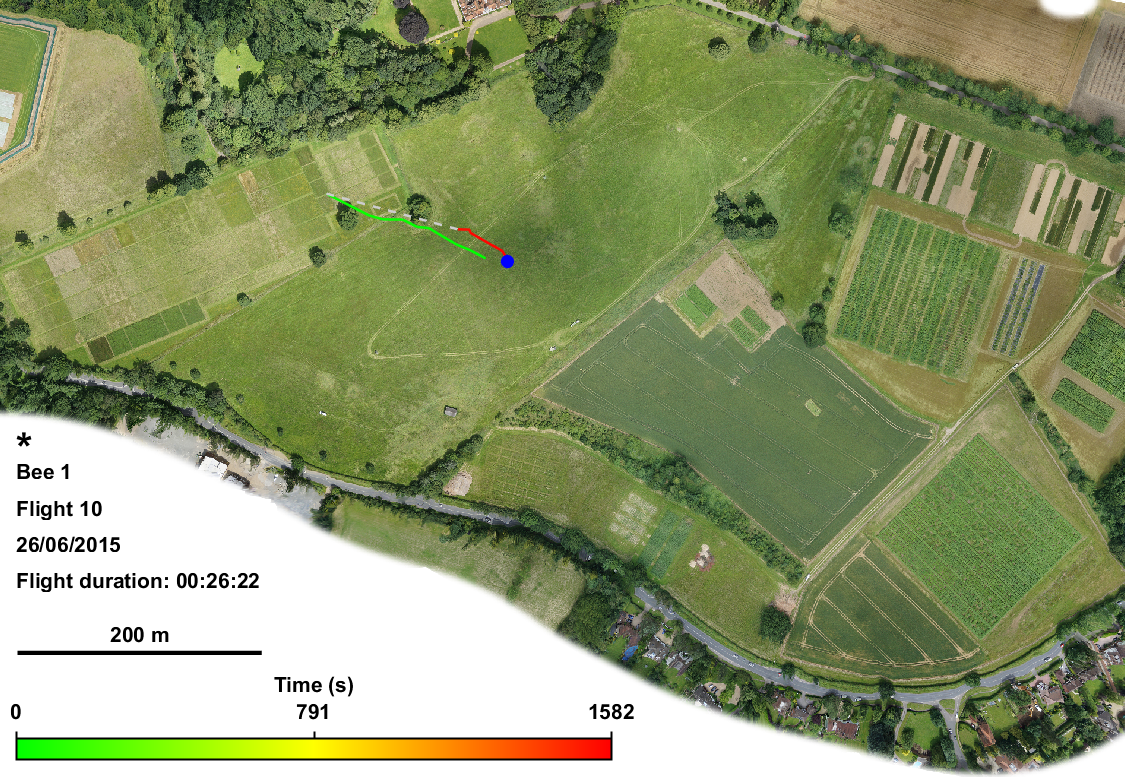

Supplement: S1 File — All flights made by Bee 1 during the period 25/06/2015–27/06/2015. Each figure represents a single flight. Bee ID, flight number, date of recording and the duration of each flight are shown in the bottom left corner of each figure. The position of the nest is marked by a blue circle. Colours represent the time from the start of each flight: initial portion of each flight is in green, changing smoothly through yellow until the end of the flight is shown in red. Grey dashed lines are used to join radar observations made more than 30 s apart, when the bee’s location was uncertain. (ZIP) [file pone.0160333.s003.zip › S11 Fig.tif]

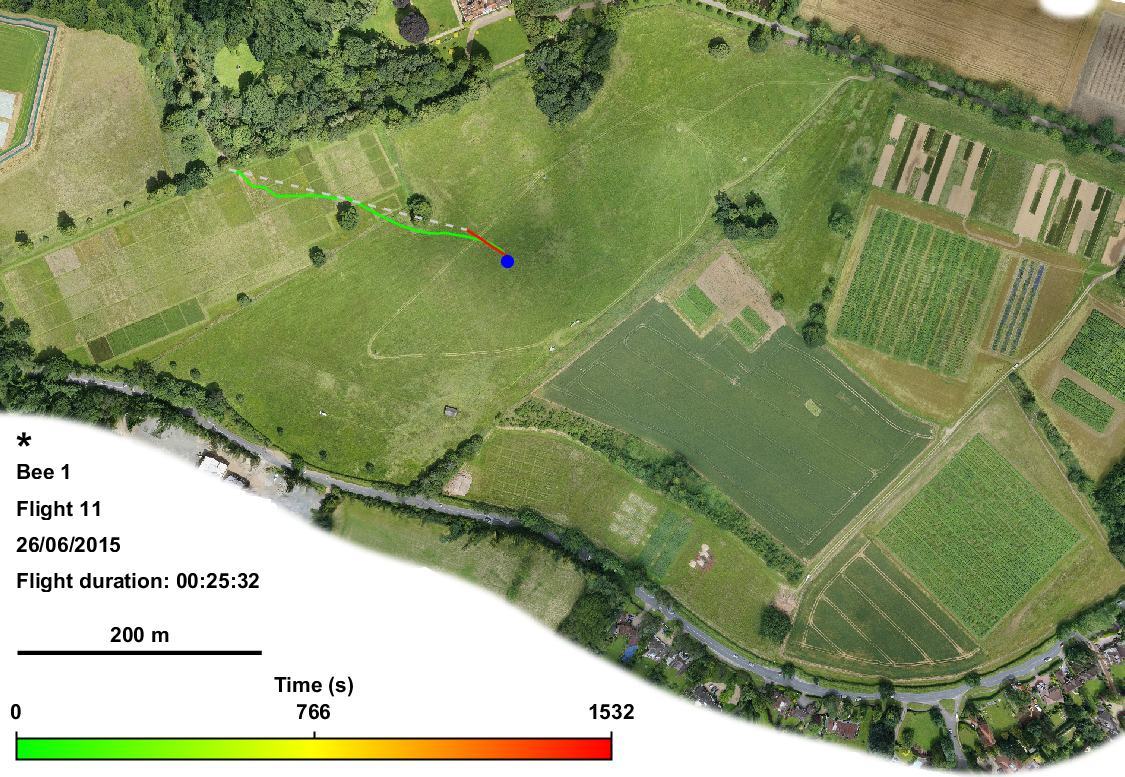

Supplement: S1 File — All flights made by Bee 1 during the period 25/06/2015–27/06/2015. Each figure represents a single flight. Bee ID, flight number, date of recording and the duration of each flight are shown in the bottom left corner of each figure. The position of the nest is marked by a blue circle. Colours represent the time from the start of each flight: initial portion of each flight is in green, changing smoothly through yellow until the end of the flight is shown in red. Grey dashed lines are used to join radar observations made more than 30 s apart, when the bee’s location was uncertain. (ZIP) [file pone.0160333.s003.zip › S12 Fig.tif]

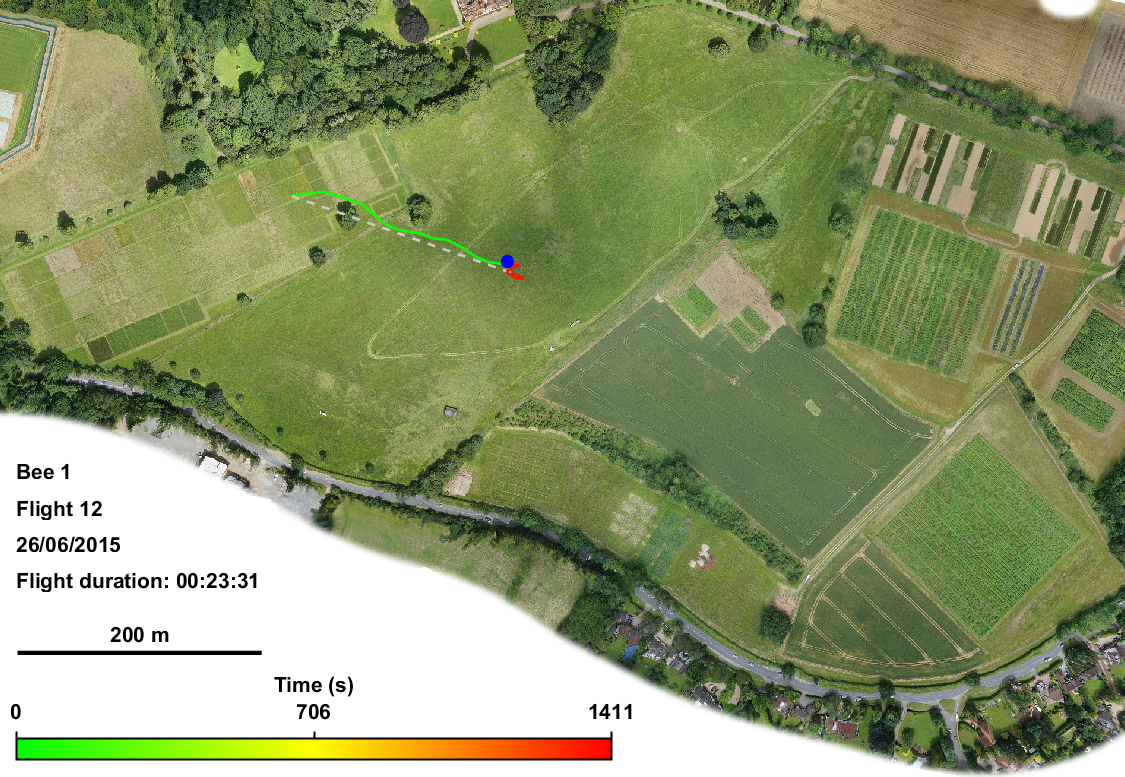

Supplement: S1 File — All flights made by Bee 1 during the period 25/06/2015–27/06/2015. Each figure represents a single flight. Bee ID, flight number, date of recording and the duration of each flight are shown in the bottom left corner of each figure. The position of the nest is marked by a blue circle. Colours represent the time from the start of each flight: initial portion of each flight is in green, changing smoothly through yellow until the end of the flight is shown in red. Grey dashed lines are used to join radar observations made more than 30 s apart, when the bee’s location was uncertain. (ZIP) [file pone.0160333.s003.zip › S13 Fig.tif]

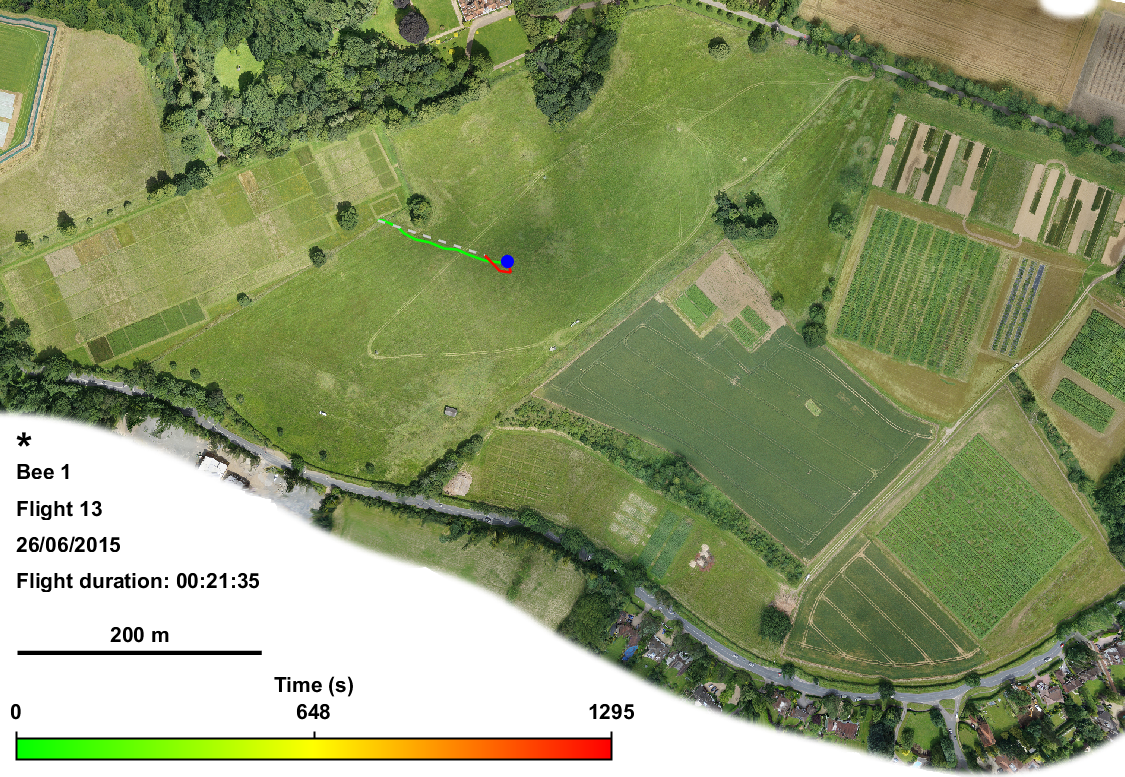

Supplement: S1 File — All flights made by Bee 1 during the period 25/06/2015–27/06/2015. Each figure represents a single flight. Bee ID, flight number, date of recording and the duration of each flight are shown in the bottom left corner of each figure. The position of the nest is marked by a blue circle. Colours represent the time from the start of each flight: initial portion of each flight is in green, changing smoothly through yellow until the end of the flight is shown in red. Grey dashed lines are used to join radar observations made more than 30 s apart, when the bee’s location was uncertain. (ZIP) [file pone.0160333.s003.zip › S14 Fig.tif]

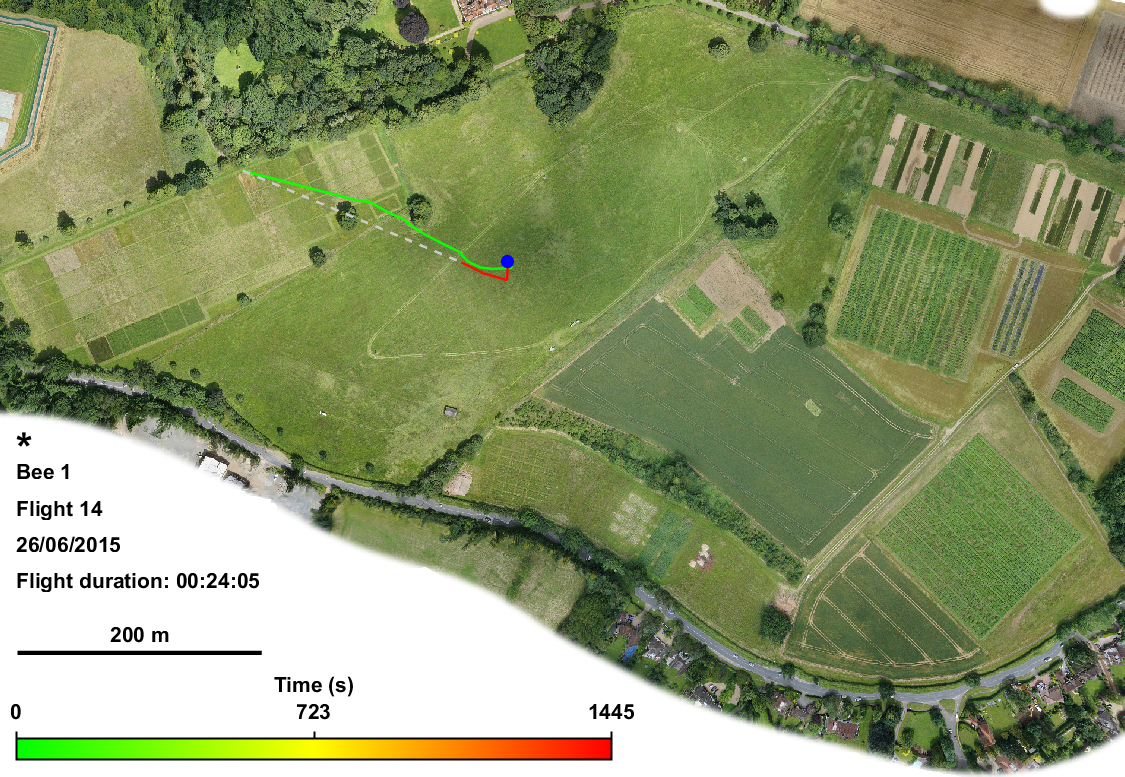

Supplement: S1 File — All flights made by Bee 1 during the period 25/06/2015–27/06/2015. Each figure represents a single flight. Bee ID, flight number, date of recording and the duration of each flight are shown in the bottom left corner of each figure. The position of the nest is marked by a blue circle. Colours represent the time from the start of each flight: initial portion of each flight is in green, changing smoothly through yellow until the end of the flight is shown in red. Grey dashed lines are used to join radar observations made more than 30 s apart, when the bee’s location was uncertain. (ZIP) [file pone.0160333.s003.zip › S15 Fig.tif]

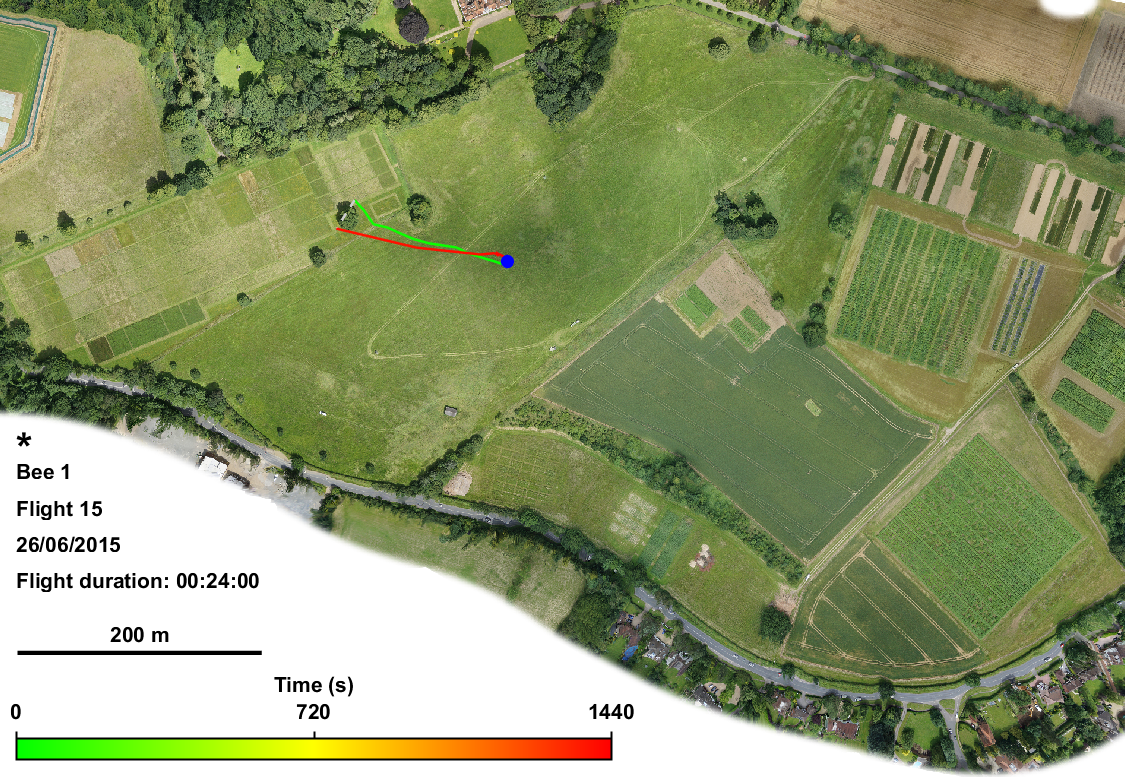

Supplement: S1 File — All flights made by Bee 1 during the period 25/06/2015–27/06/2015. Each figure represents a single flight. Bee ID, flight number, date of recording and the duration of each flight are shown in the bottom left corner of each figure. The position of the nest is marked by a blue circle. Colours represent the time from the start of each flight: initial portion of each flight is in green, changing smoothly through yellow until the end of the flight is shown in red. Grey dashed lines are used to join radar observations made more than 30 s apart, when the bee’s location was uncertain. (ZIP) [file pone.0160333.s003.zip › S16 Fig.tif]

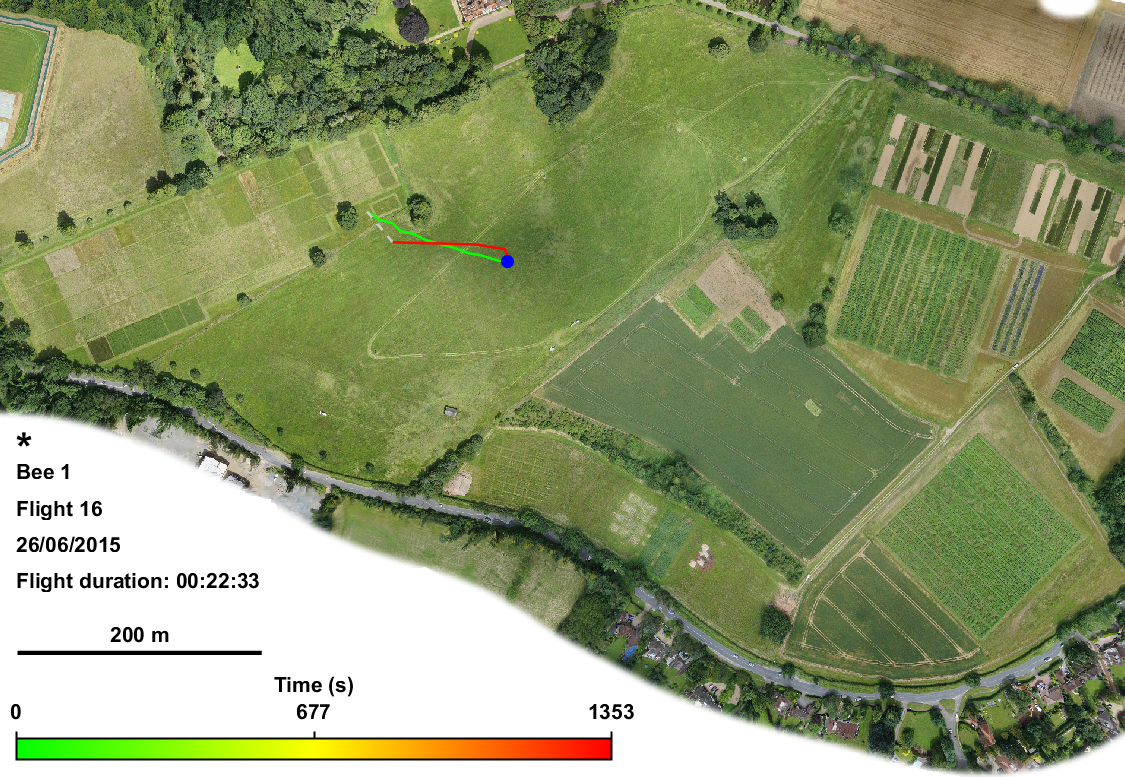

Supplement: S1 File — All flights made by Bee 1 during the period 25/06/2015–27/06/2015. Each figure represents a single flight. Bee ID, flight number, date of recording and the duration of each flight are shown in the bottom left corner of each figure. The position of the nest is marked by a blue circle. Colours represent the time from the start of each flight: initial portion of each flight is in green, changing smoothly through yellow until the end of the flight is shown in red. Grey dashed lines are used to join radar observations made more than 30 s apart, when the bee’s location was uncertain. (ZIP) [file pone.0160333.s003.zip › S17 Fig.tif]

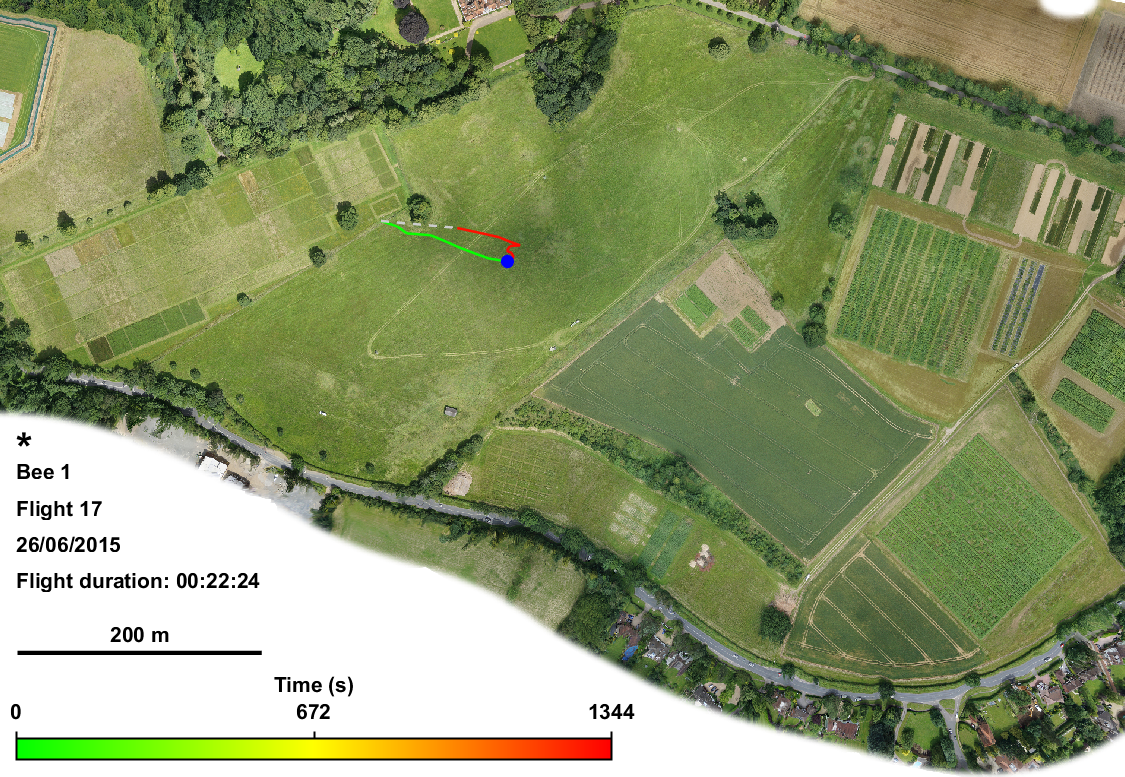

Supplement: S1 File — All flights made by Bee 1 during the period 25/06/2015–27/06/2015. Each figure represents a single flight. Bee ID, flight number, date of recording and the duration of each flight are shown in the bottom left corner of each figure. The position of the nest is marked by a blue circle. Colours represent the time from the start of each flight: initial portion of each flight is in green, changing smoothly through yellow until the end of the flight is shown in red. Grey dashed lines are used to join radar observations made more than 30 s apart, when the bee’s location was uncertain. (ZIP) [file pone.0160333.s003.zip › S18 Fig.tif]

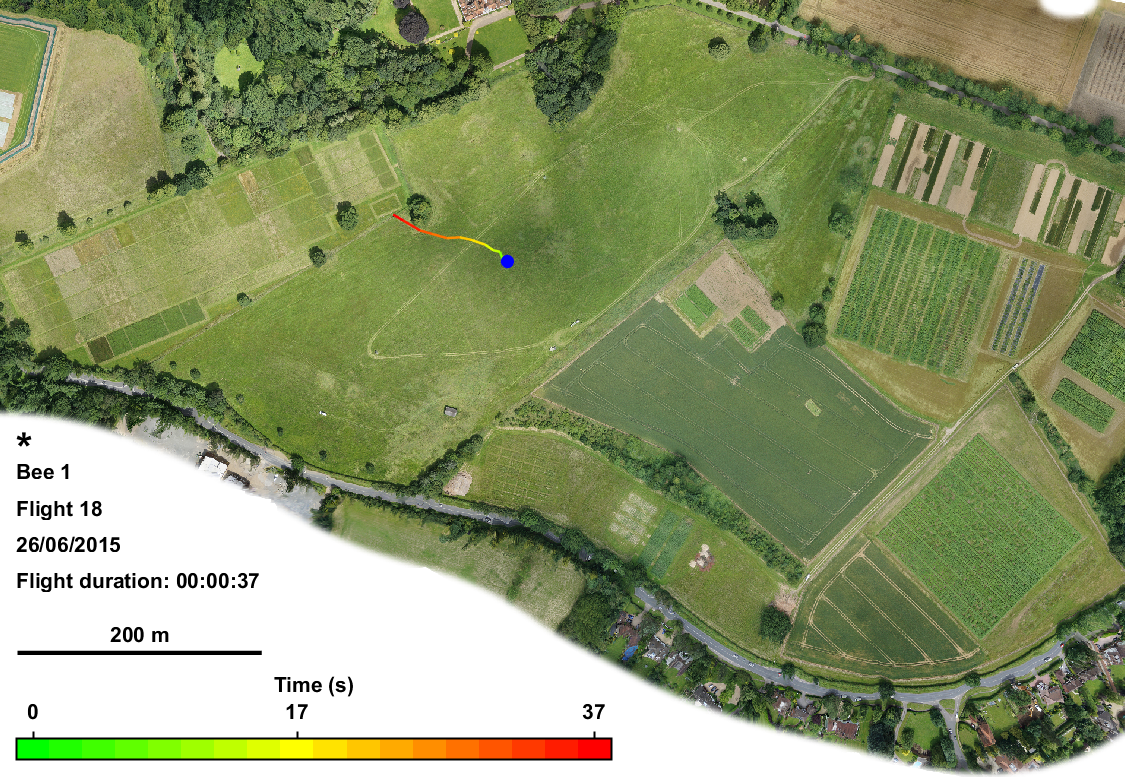

Supplement: S1 File — All flights made by Bee 1 during the period 25/06/2015–27/06/2015. Each figure represents a single flight. Bee ID, flight number, date of recording and the duration of each flight are shown in the bottom left corner of each figure. The position of the nest is marked by a blue circle. Colours represent the time from the start of each flight: initial portion of each flight is in green, changing smoothly through yellow until the end of the flight is shown in red. Grey dashed lines are used to join radar observations made more than 30 s apart, when the bee’s location was uncertain. (ZIP) [file pone.0160333.s003.zip › S19 Fig.tif]

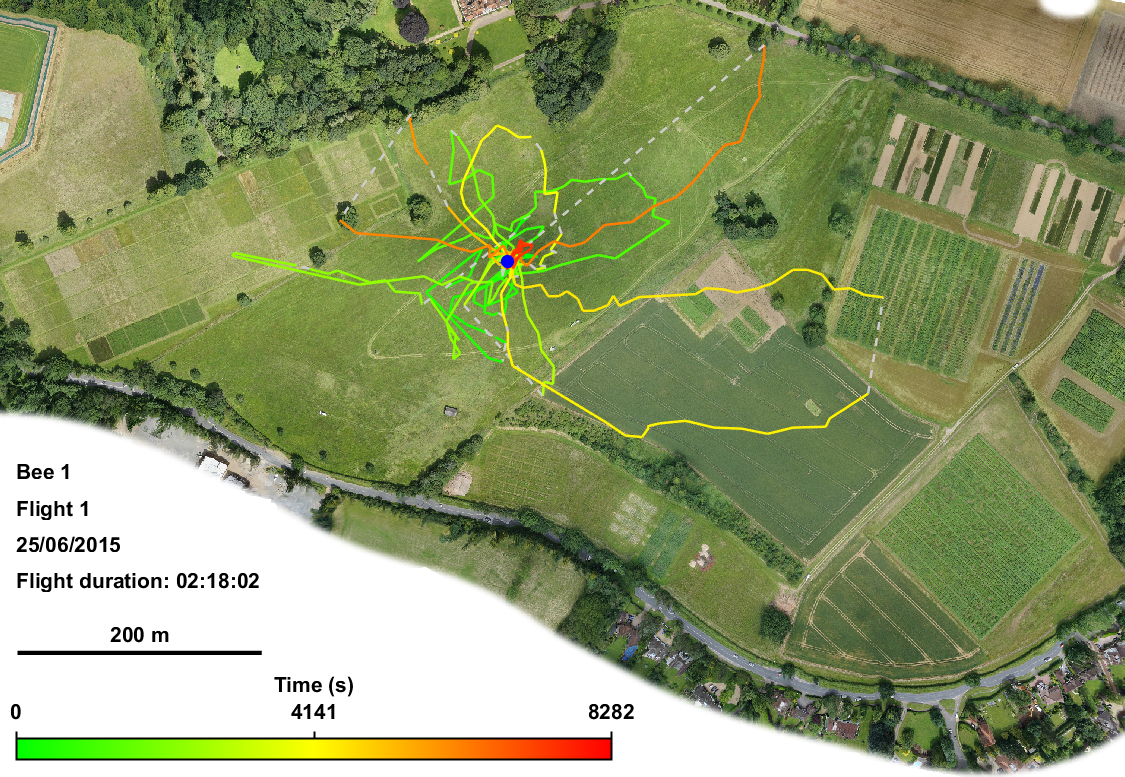

Supplement: S1 File — All flights made by Bee 1 during the period 25/06/2015–27/06/2015. Each figure represents a single flight. Bee ID, flight number, date of recording and the duration of each flight are shown in the bottom left corner of each figure. The position of the nest is marked by a blue circle. Colours represent the time from the start of each flight: initial portion of each flight is in green, changing smoothly through yellow until the end of the flight is shown in red. Grey dashed lines are used to join radar observations made more than 30 s apart, when the bee’s location was uncertain. (ZIP) [file pone.0160333.s003.zip › S2 Fig.tif]

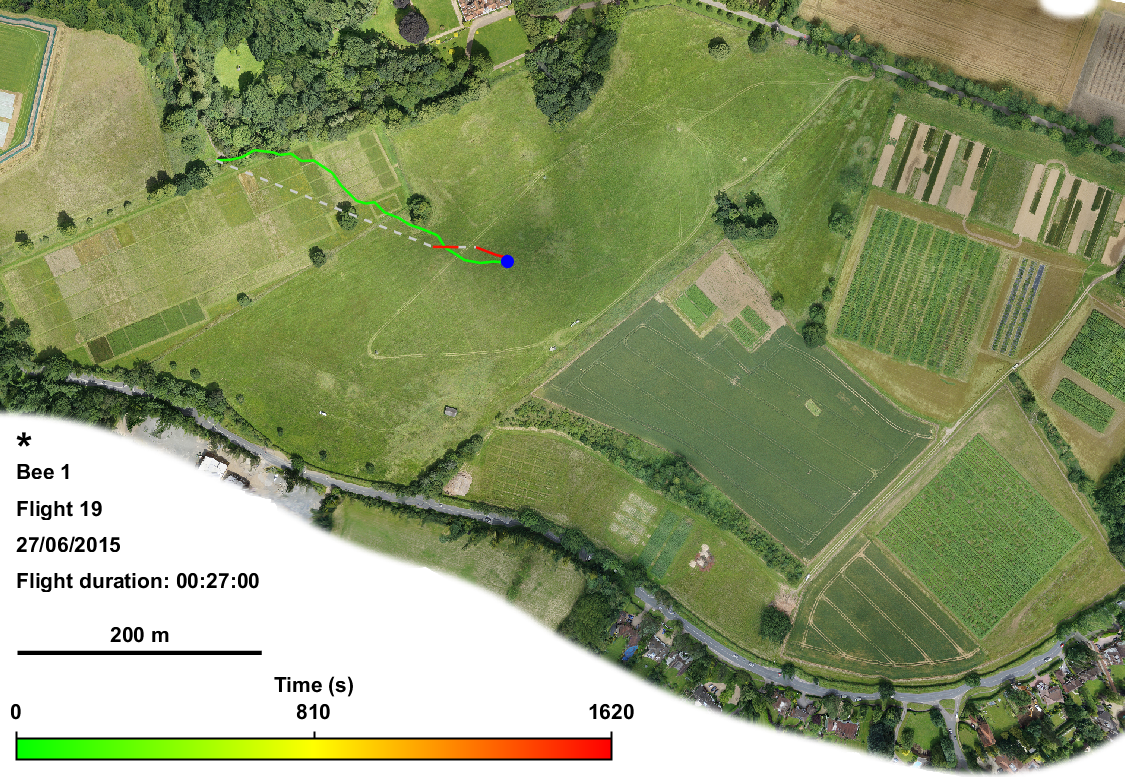

Supplement: S1 File — All flights made by Bee 1 during the period 25/06/2015–27/06/2015. Each figure represents a single flight. Bee ID, flight number, date of recording and the duration of each flight are shown in the bottom left corner of each figure. The position of the nest is marked by a blue circle. Colours represent the time from the start of each flight: initial portion of each flight is in green, changing smoothly through yellow until the end of the flight is shown in red. Grey dashed lines are used to join radar observations made more than 30 s apart, when the bee’s location was uncertain. (ZIP) [file pone.0160333.s003.zip › S20 Fig.tif]

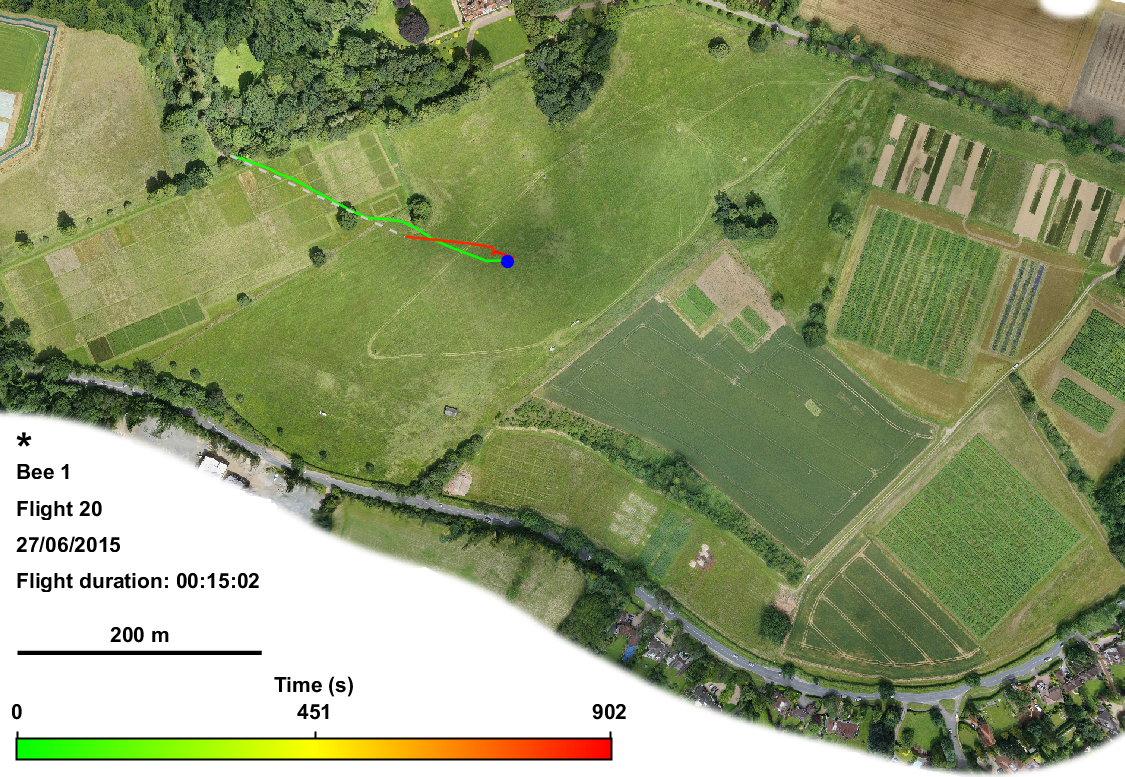

Supplement: S1 File — All flights made by Bee 1 during the period 25/06/2015–27/06/2015. Each figure represents a single flight. Bee ID, flight number, date of recording and the duration of each flight are shown in the bottom left corner of each figure. The position of the nest is marked by a blue circle. Colours represent the time from the start of each flight: initial portion of each flight is in green, changing smoothly through yellow until the end of the flight is shown in red. Grey dashed lines are used to join radar observations made more than 30 s apart, when the bee’s location was uncertain. (ZIP) [file pone.0160333.s003.zip › S21 Fig.tif]

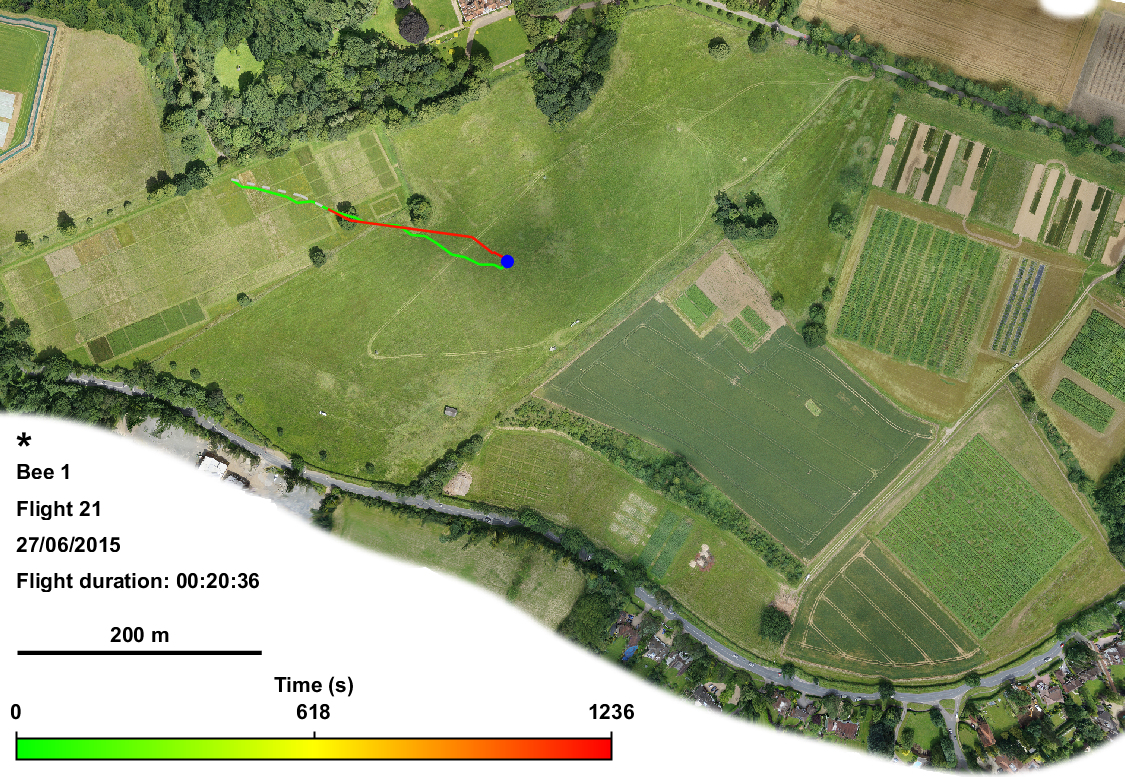

Supplement: S1 File — All flights made by Bee 1 during the period 25/06/2015–27/06/2015. Each figure represents a single flight. Bee ID, flight number, date of recording and the duration of each flight are shown in the bottom left corner of each figure. The position of the nest is marked by a blue circle. Colours represent the time from the start of each flight: initial portion of each flight is in green, changing smoothly through yellow until the end of the flight is shown in red. Grey dashed lines are used to join radar observations made more than 30 s apart, when the bee’s location was uncertain. (ZIP) [file pone.0160333.s003.zip › S22 Fig.tif]

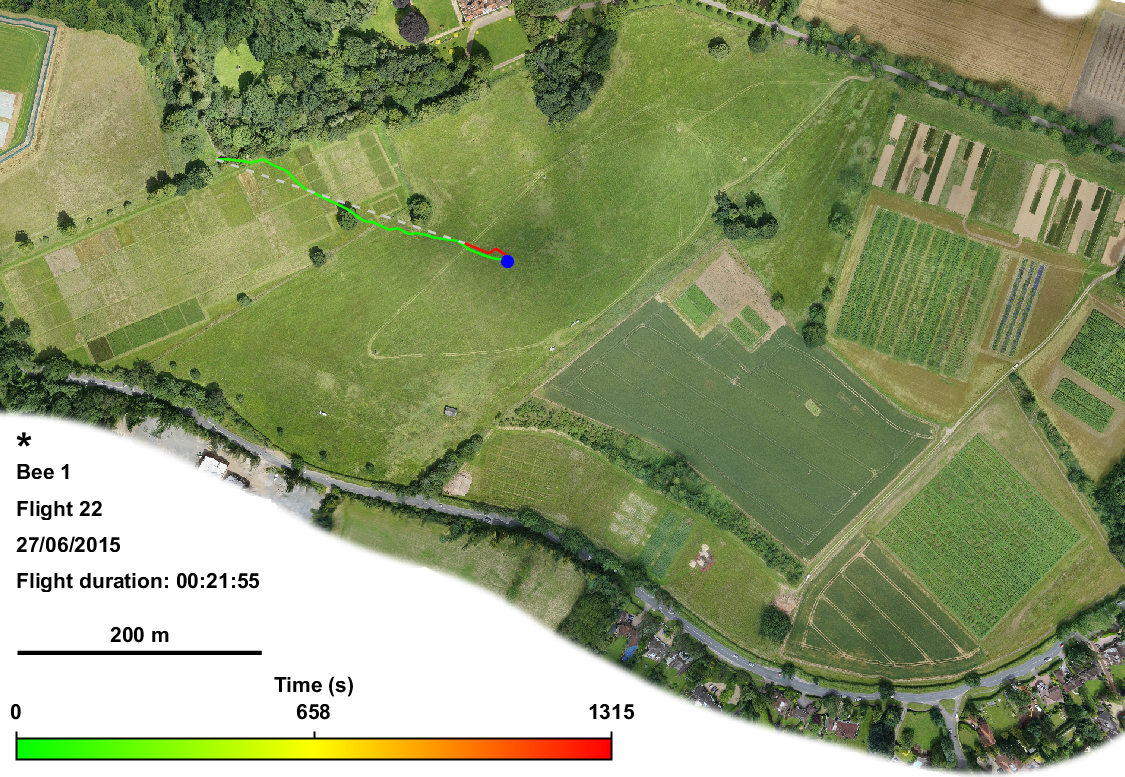

Supplement: S1 File — All flights made by Bee 1 during the period 25/06/2015–27/06/2015. Each figure represents a single flight. Bee ID, flight number, date of recording and the duration of each flight are shown in the bottom left corner of each figure. The position of the nest is marked by a blue circle. Colours represent the time from the start of each flight: initial portion of each flight is in green, changing smoothly through yellow until the end of the flight is shown in red. Grey dashed lines are used to join radar observations made more than 30 s apart, when the bee’s location was uncertain. (ZIP) [file pone.0160333.s003.zip › S23 Fig.tif]

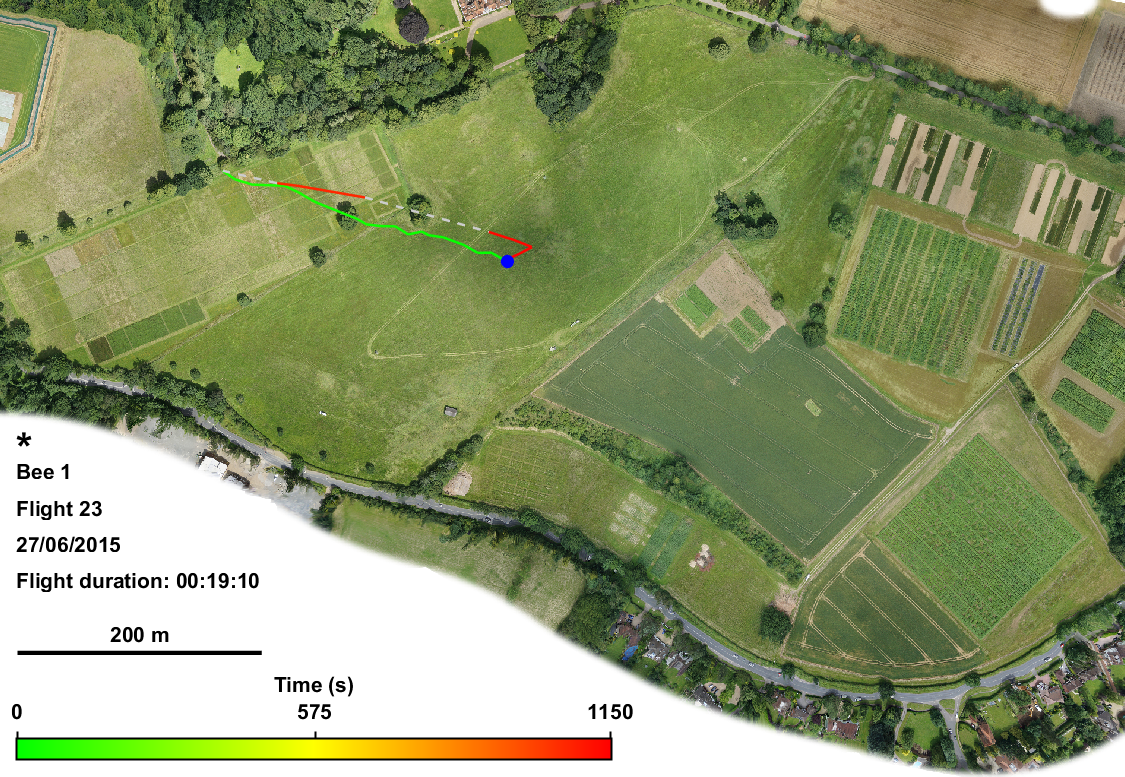

Supplement: S1 File — All flights made by Bee 1 during the period 25/06/2015–27/06/2015. Each figure represents a single flight. Bee ID, flight number, date of recording and the duration of each flight are shown in the bottom left corner of each figure. The position of the nest is marked by a blue circle. Colours represent the time from the start of each flight: initial portion of each flight is in green, changing smoothly through yellow until the end of the flight is shown in red. Grey dashed lines are used to join radar observations made more than 30 s apart, when the bee’s location was uncertain. (ZIP) [file pone.0160333.s003.zip › S24 Fig.tif]

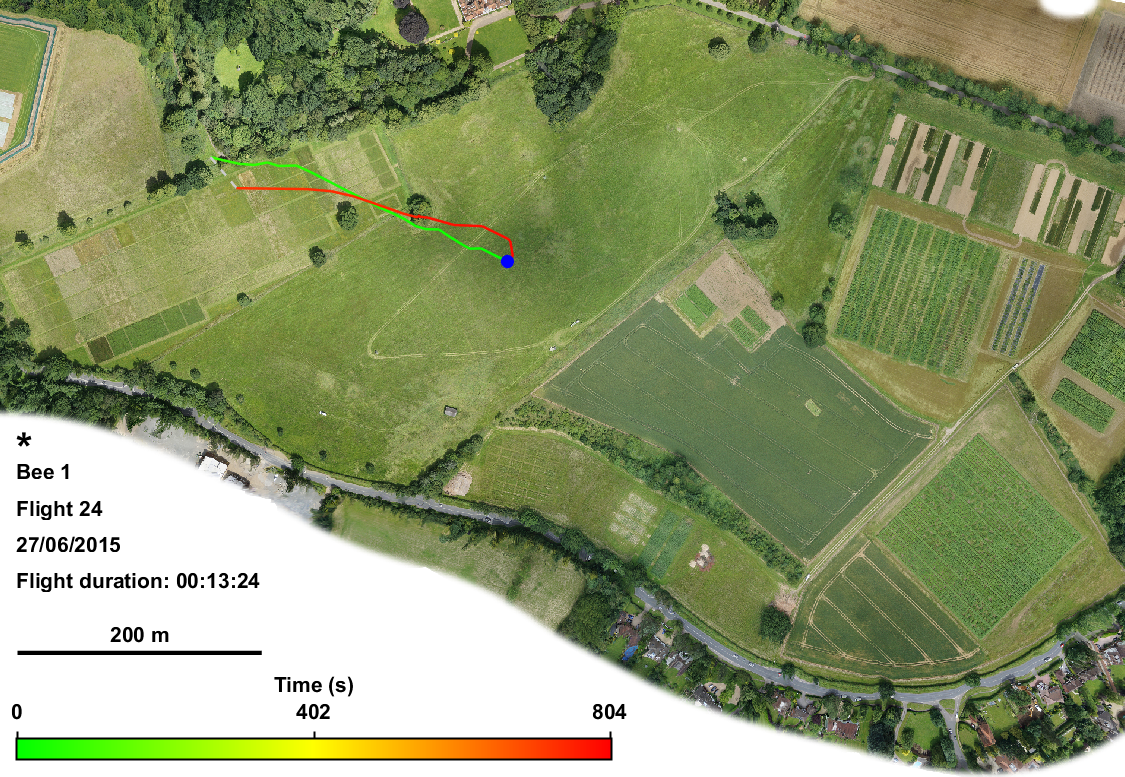

Supplement: S1 File — All flights made by Bee 1 during the period 25/06/2015–27/06/2015. Each figure represents a single flight. Bee ID, flight number, date of recording and the duration of each flight are shown in the bottom left corner of each figure. The position of the nest is marked by a blue circle. Colours represent the time from the start of each flight: initial portion of each flight is in green, changing smoothly through yellow until the end of the flight is shown in red. Grey dashed lines are used to join radar observations made more than 30 s apart, when the bee’s location was uncertain. (ZIP) [file pone.0160333.s003.zip › S25 Fig.tif]

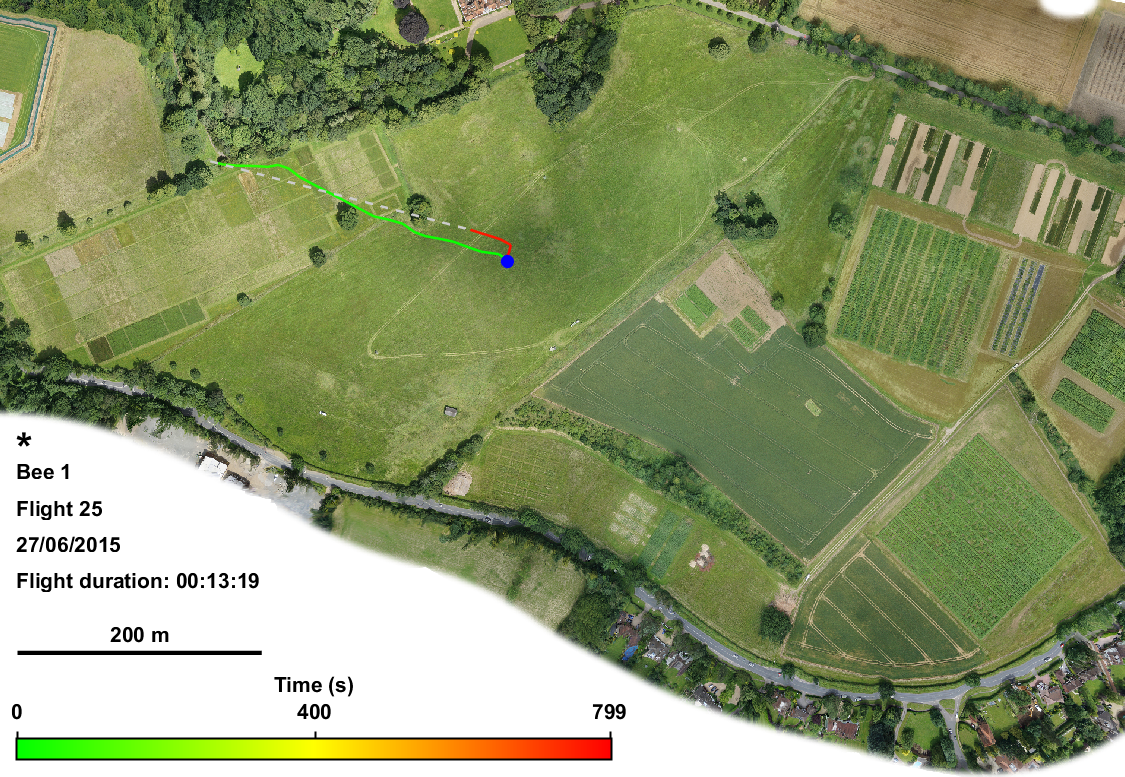

Supplement: S1 File — All flights made by Bee 1 during the period 25/06/2015–27/06/2015. Each figure represents a single flight. Bee ID, flight number, date of recording and the duration of each flight are shown in the bottom left corner of each figure. The position of the nest is marked by a blue circle. Colours represent the time from the start of each flight: initial portion of each flight is in green, changing smoothly through yellow until the end of the flight is shown in red. Grey dashed lines are used to join radar observations made more than 30 s apart, when the bee’s location was uncertain. (ZIP) [file pone.0160333.s003.zip › S26 Fig.tif]

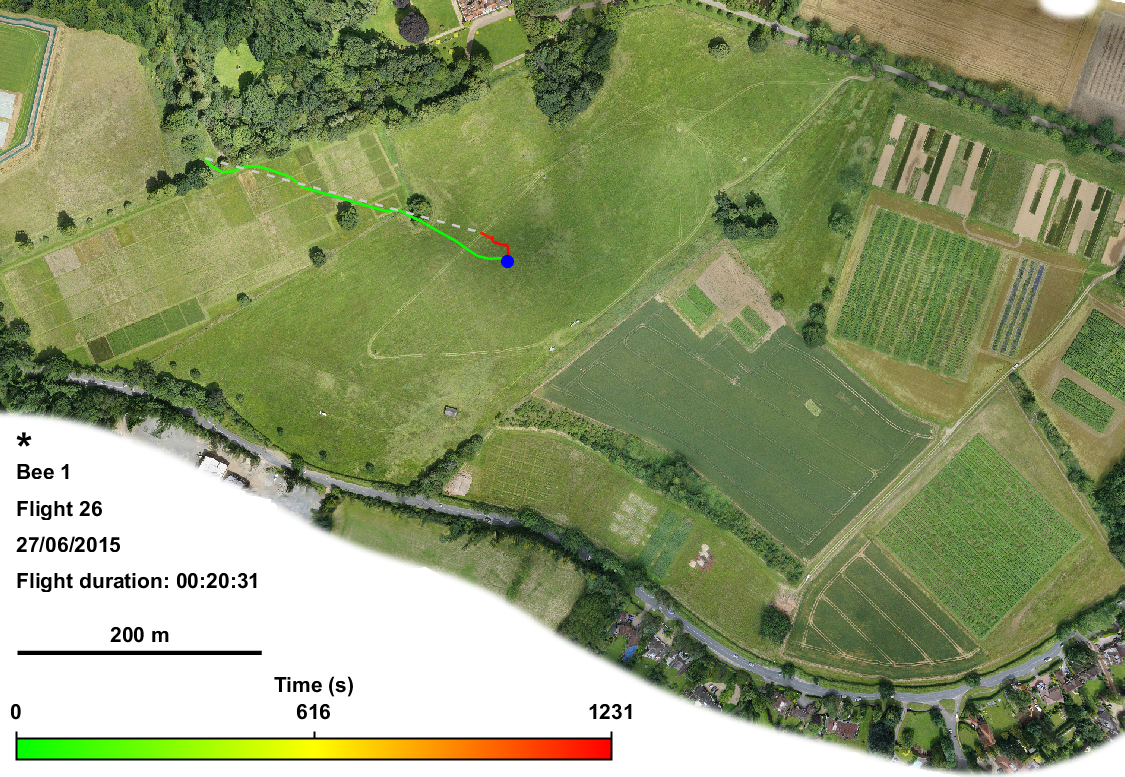

Supplement: S1 File — All flights made by Bee 1 during the period 25/06/2015–27/06/2015. Each figure represents a single flight. Bee ID, flight number, date of recording and the duration of each flight are shown in the bottom left corner of each figure. The position of the nest is marked by a blue circle. Colours represent the time from the start of each flight: initial portion of each flight is in green, changing smoothly through yellow until the end of the flight is shown in red. Grey dashed lines are used to join radar observations made more than 30 s apart, when the bee’s location was uncertain. (ZIP) [file pone.0160333.s003.zip › S27 Fig.tif]

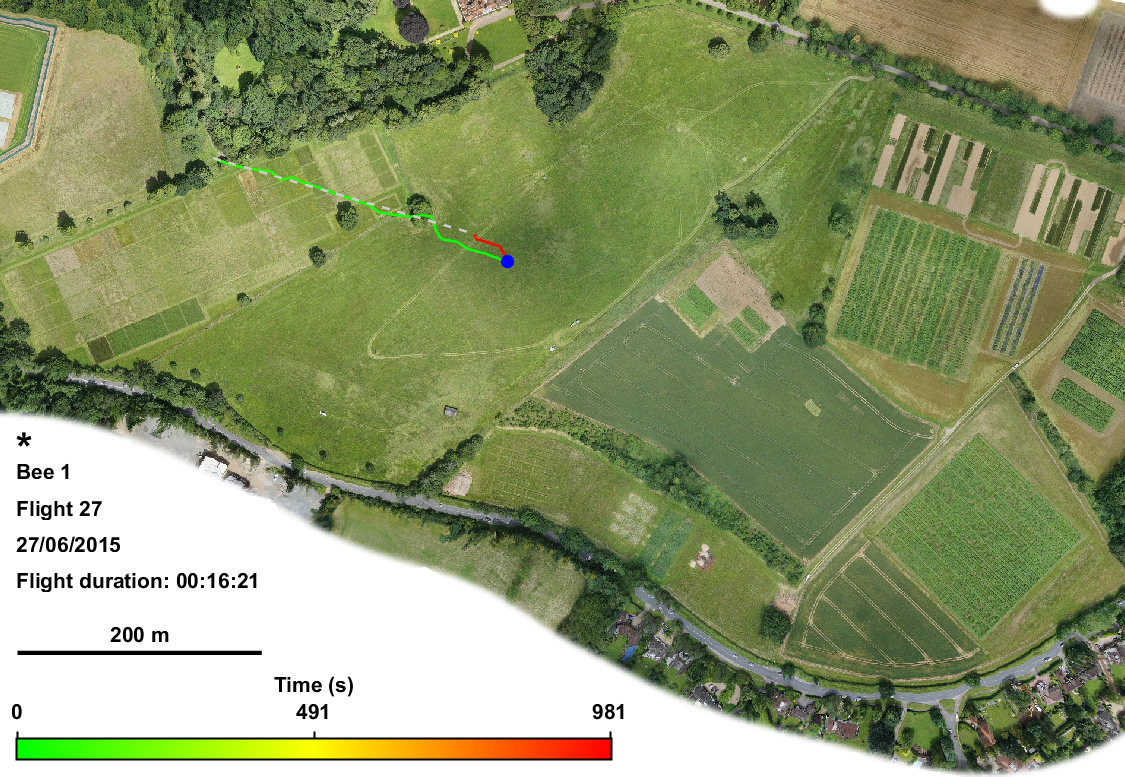

Supplement: S1 File — All flights made by Bee 1 during the period 25/06/2015–27/06/2015. Each figure represents a single flight. Bee ID, flight number, date of recording and the duration of each flight are shown in the bottom left corner of each figure. The position of the nest is marked by a blue circle. Colours represent the time from the start of each flight: initial portion of each flight is in green, changing smoothly through yellow until the end of the flight is shown in red. Grey dashed lines are used to join radar observations made more than 30 s apart, when the bee’s location was uncertain. (ZIP) [file pone.0160333.s003.zip › S28 Fig.tif]

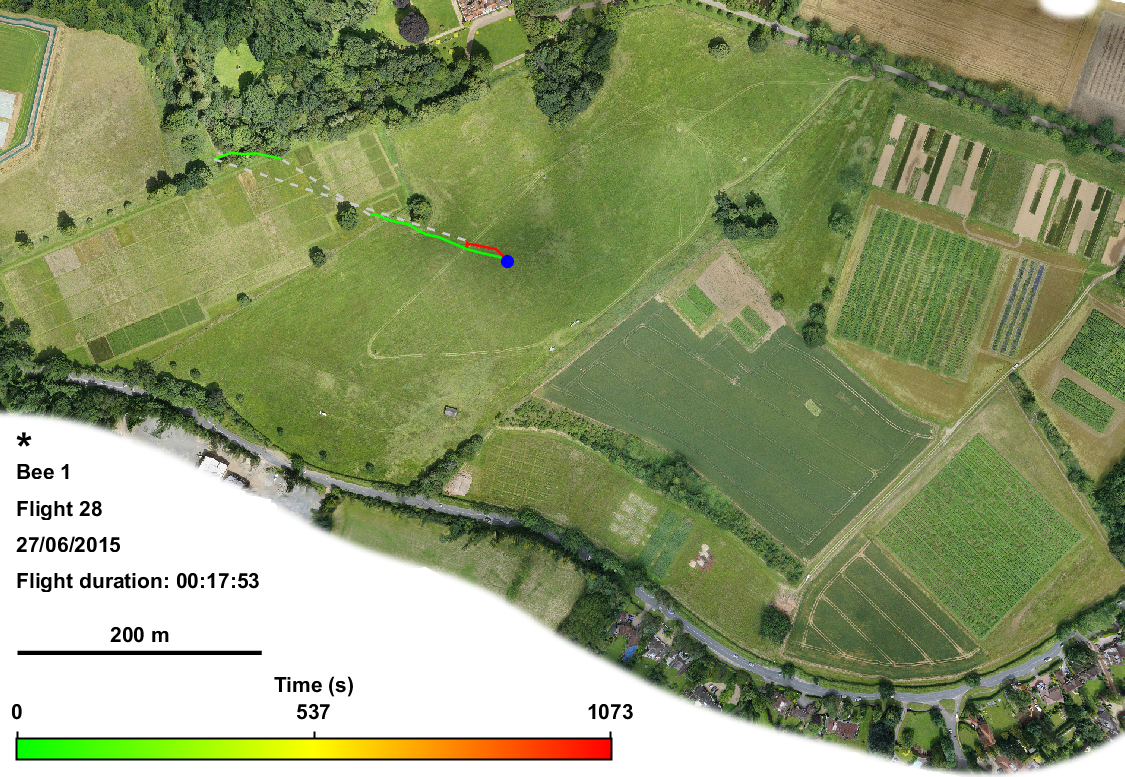

Supplement: S1 File — All flights made by Bee 1 during the period 25/06/2015–27/06/2015. Each figure represents a single flight. Bee ID, flight number, date of recording and the duration of each flight are shown in the bottom left corner of each figure. The position of the nest is marked by a blue circle. Colours represent the time from the start of each flight: initial portion of each flight is in green, changing smoothly through yellow until the end of the flight is shown in red. Grey dashed lines are used to join radar observations made more than 30 s apart, when the bee’s location was uncertain. (ZIP) [file pone.0160333.s003.zip › S29 Fig.tif]

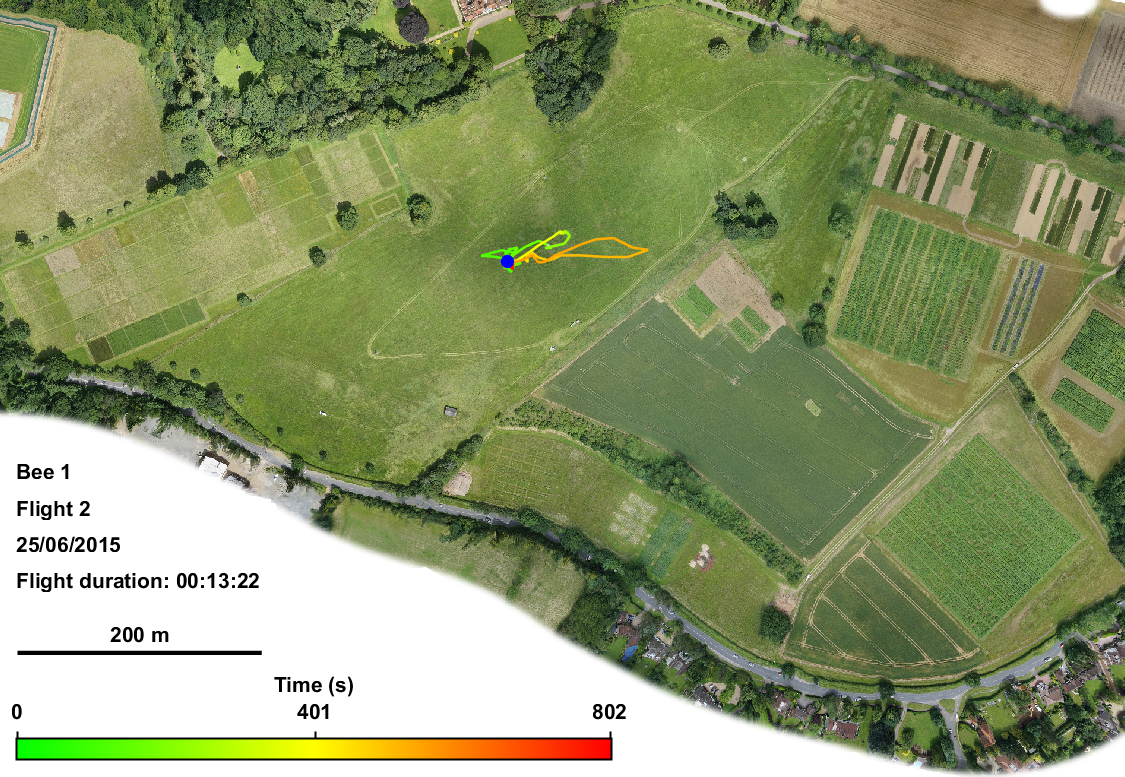

Supplement: S1 File — All flights made by Bee 1 during the period 25/06/2015–27/06/2015. Each figure represents a single flight. Bee ID, flight number, date of recording and the duration of each flight are shown in the bottom left corner of each figure. The position of the nest is marked by a blue circle. Colours represent the time from the start of each flight: initial portion of each flight is in green, changing smoothly through yellow until the end of the flight is shown in red. Grey dashed lines are used to join radar observations made more than 30 s apart, when the bee’s location was uncertain. (ZIP) [file pone.0160333.s003.zip › S3 Fig.tif]

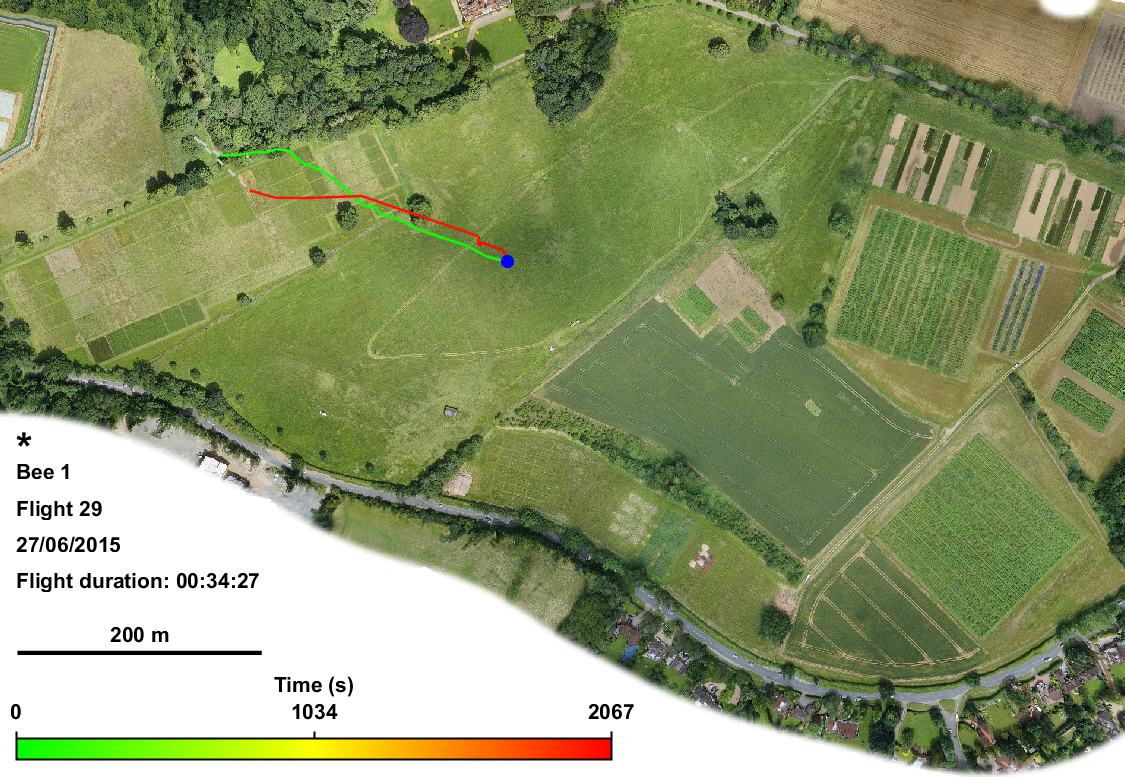

Supplement: S1 File — All flights made by Bee 1 during the period 25/06/2015–27/06/2015. Each figure represents a single flight. Bee ID, flight number, date of recording and the duration of each flight are shown in the bottom left corner of each figure. The position of the nest is marked by a blue circle. Colours represent the time from the start of each flight: initial portion of each flight is in green, changing smoothly through yellow until the end of the flight is shown in red. Grey dashed lines are used to join radar observations made more than 30 s apart, when the bee’s location was uncertain. (ZIP) [file pone.0160333.s003.zip › S30 Fig.tif]

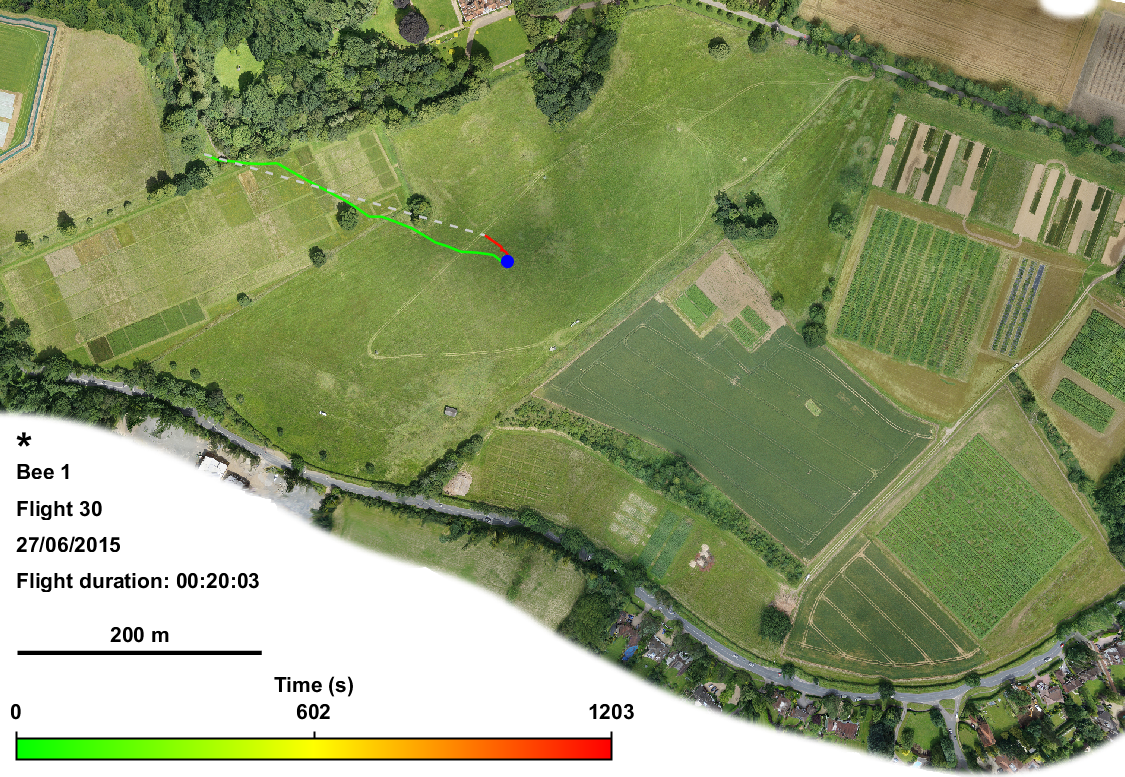

Supplement: S1 File — All flights made by Bee 1 during the period 25/06/2015–27/06/2015. Each figure represents a single flight. Bee ID, flight number, date of recording and the duration of each flight are shown in the bottom left corner of each figure. The position of the nest is marked by a blue circle. Colours represent the time from the start of each flight: initial portion of each flight is in green, changing smoothly through yellow until the end of the flight is shown in red. Grey dashed lines are used to join radar observations made more than 30 s apart, when the bee’s location was uncertain. (ZIP) [file pone.0160333.s003.zip › S31 Fig.tif]

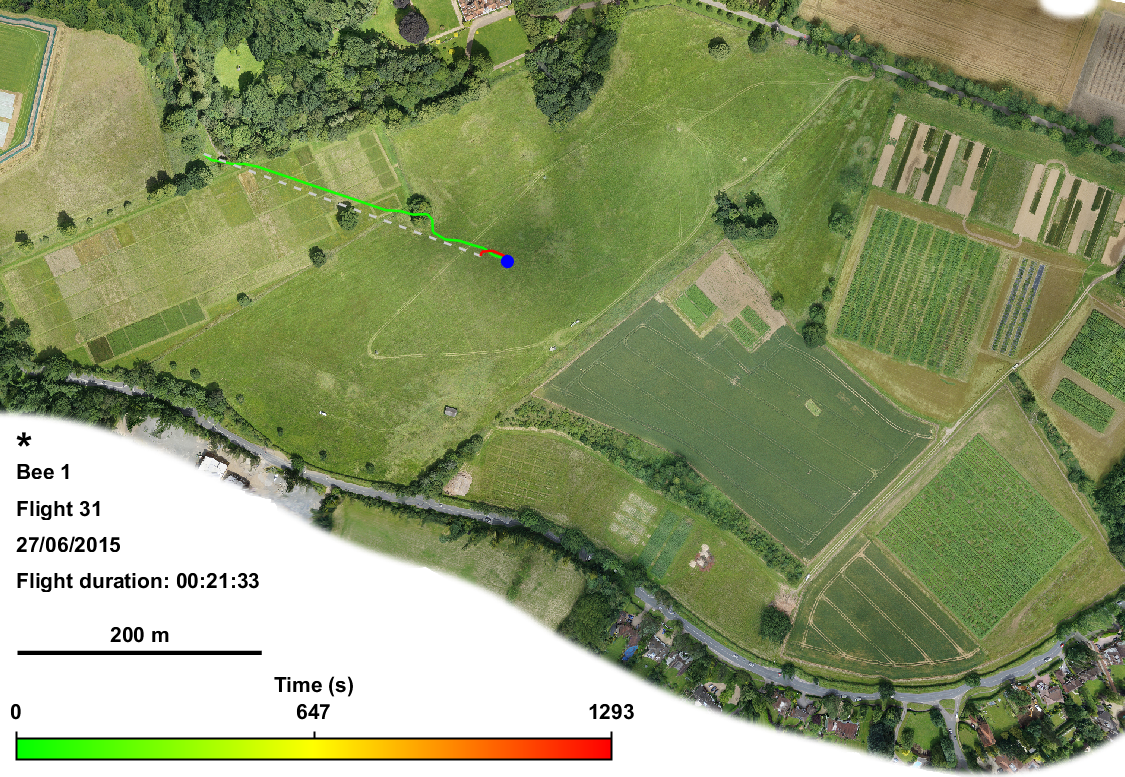

Supplement: S1 File — All flights made by Bee 1 during the period 25/06/2015–27/06/2015. Each figure represents a single flight. Bee ID, flight number, date of recording and the duration of each flight are shown in the bottom left corner of each figure. The position of the nest is marked by a blue circle. Colours represent the time from the start of each flight: initial portion of each flight is in green, changing smoothly through yellow until the end of the flight is shown in red. Grey dashed lines are used to join radar observations made more than 30 s apart, when the bee’s location was uncertain. (ZIP) [file pone.0160333.s003.zip › S32 Fig.tif]

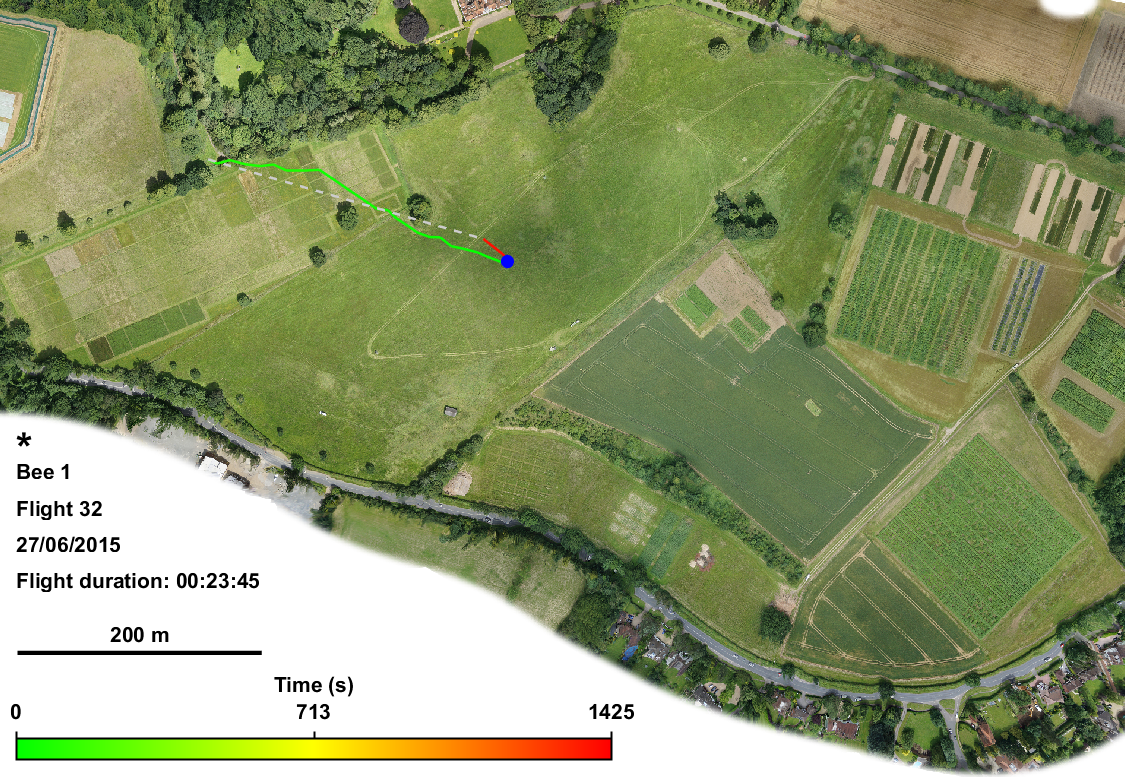

Supplement: S1 File — All flights made by Bee 1 during the period 25/06/2015–27/06/2015. Each figure represents a single flight. Bee ID, flight number, date of recording and the duration of each flight are shown in the bottom left corner of each figure. The position of the nest is marked by a blue circle. Colours represent the time from the start of each flight: initial portion of each flight is in green, changing smoothly through yellow until the end of the flight is shown in red. Grey dashed lines are used to join radar observations made more than 30 s apart, when the bee’s location was uncertain. (ZIP) [file pone.0160333.s003.zip › S33 Fig.tif]

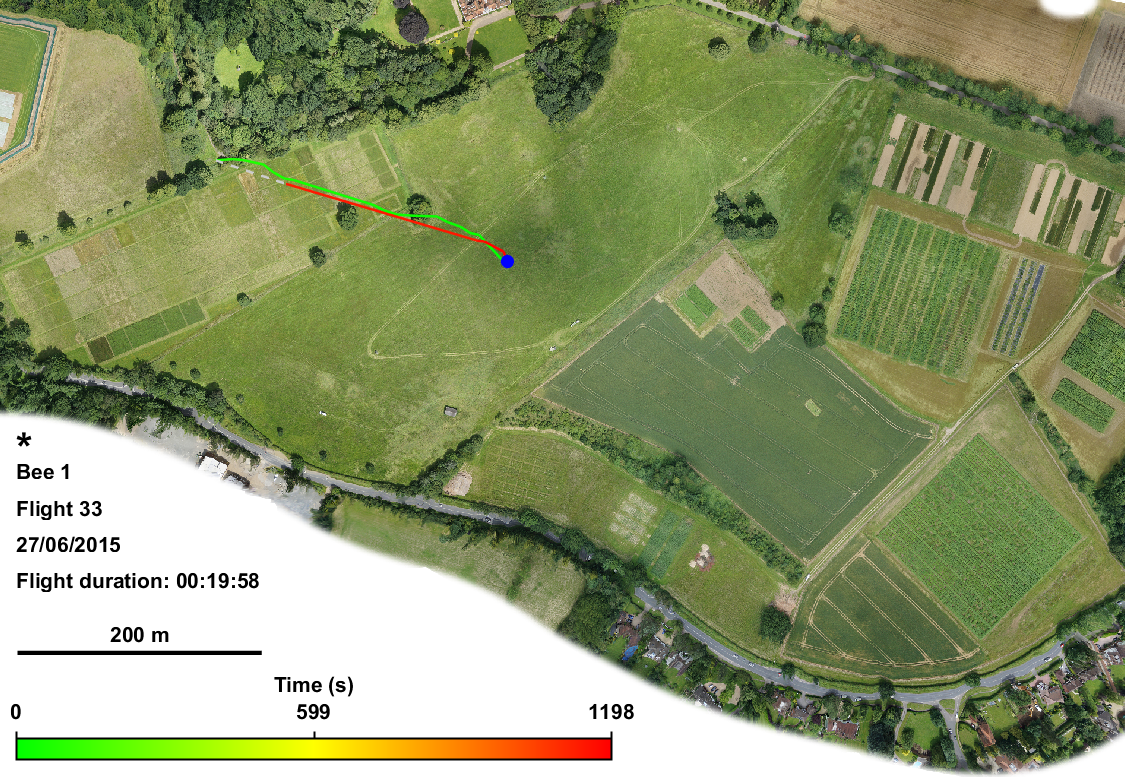

Supplement: S1 File — All flights made by Bee 1 during the period 25/06/2015–27/06/2015. Each figure represents a single flight. Bee ID, flight number, date of recording and the duration of each flight are shown in the bottom left corner of each figure. The position of the nest is marked by a blue circle. Colours represent the time from the start of each flight: initial portion of each flight is in green, changing smoothly through yellow until the end of the flight is shown in red. Grey dashed lines are used to join radar observations made more than 30 s apart, when the bee’s location was uncertain. (ZIP) [file pone.0160333.s003.zip › S34 Fig.tif]

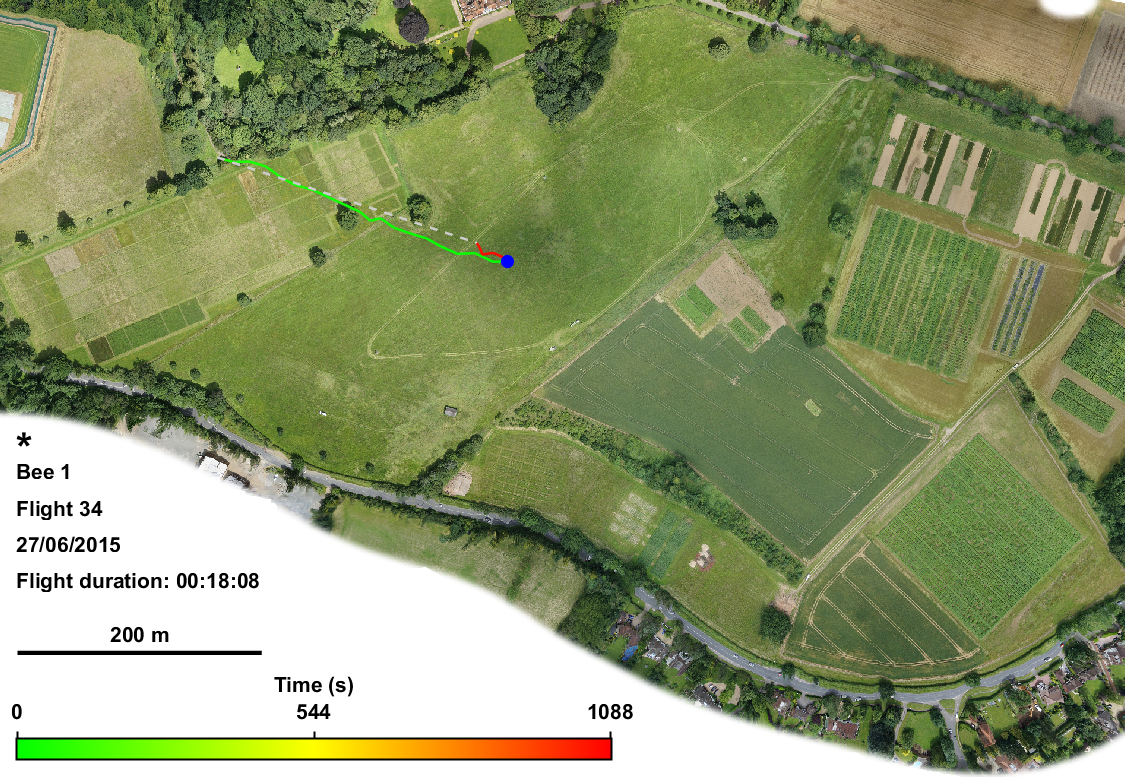

Supplement: S1 File — All flights made by Bee 1 during the period 25/06/2015–27/06/2015. Each figure represents a single flight. Bee ID, flight number, date of recording and the duration of each flight are shown in the bottom left corner of each figure. The position of the nest is marked by a blue circle. Colours represent the time from the start of each flight: initial portion of each flight is in green, changing smoothly through yellow until the end of the flight is shown in red. Grey dashed lines are used to join radar observations made more than 30 s apart, when the bee’s location was uncertain. (ZIP) [file pone.0160333.s003.zip › S35 Fig.tif]

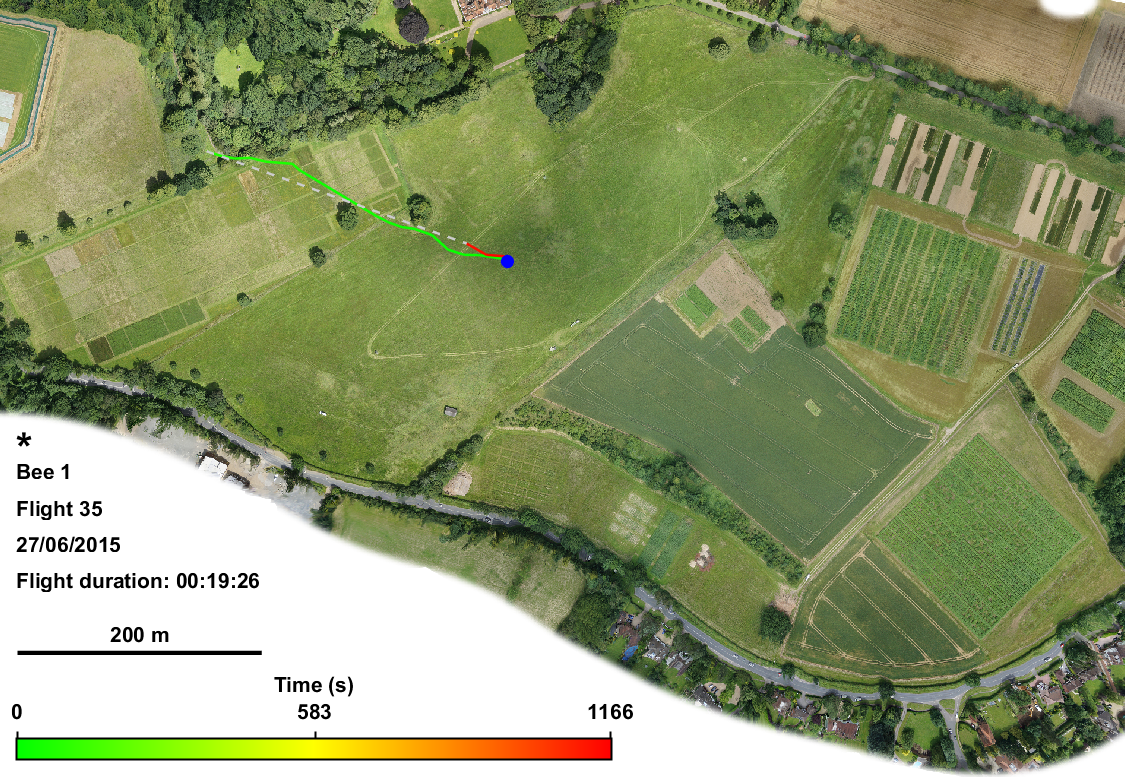

Supplement: S1 File — All flights made by Bee 1 during the period 25/06/2015–27/06/2015. Each figure represents a single flight. Bee ID, flight number, date of recording and the duration of each flight are shown in the bottom left corner of each figure. The position of the nest is marked by a blue circle. Colours represent the time from the start of each flight: initial portion of each flight is in green, changing smoothly through yellow until the end of the flight is shown in red. Grey dashed lines are used to join radar observations made more than 30 s apart, when the bee’s location was uncertain. (ZIP) [file pone.0160333.s003.zip › S36 Fig.tif]

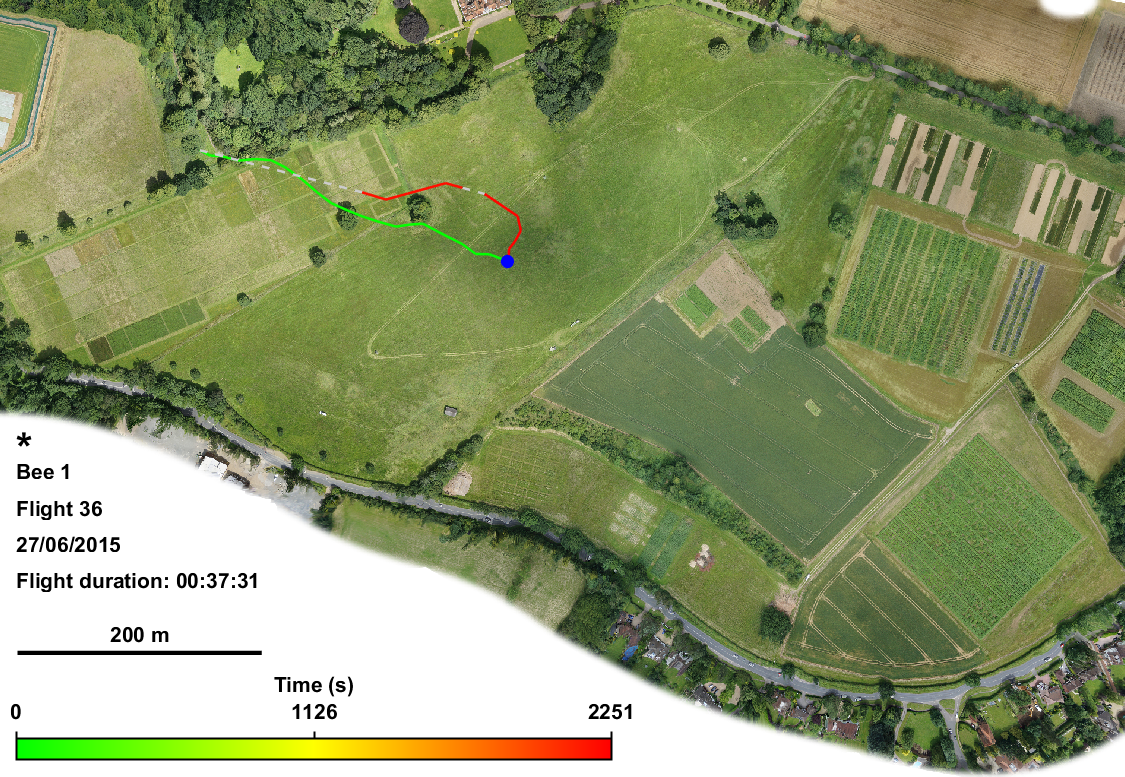

Supplement: S1 File — All flights made by Bee 1 during the period 25/06/2015–27/06/2015. Each figure represents a single flight. Bee ID, flight number, date of recording and the duration of each flight are shown in the bottom left corner of each figure. The position of the nest is marked by a blue circle. Colours represent the time from the start of each flight: initial portion of each flight is in green, changing smoothly through yellow until the end of the flight is shown in red. Grey dashed lines are used to join radar observations made more than 30 s apart, when the bee’s location was uncertain. (ZIP) [file pone.0160333.s003.zip › S37 Fig.tif]

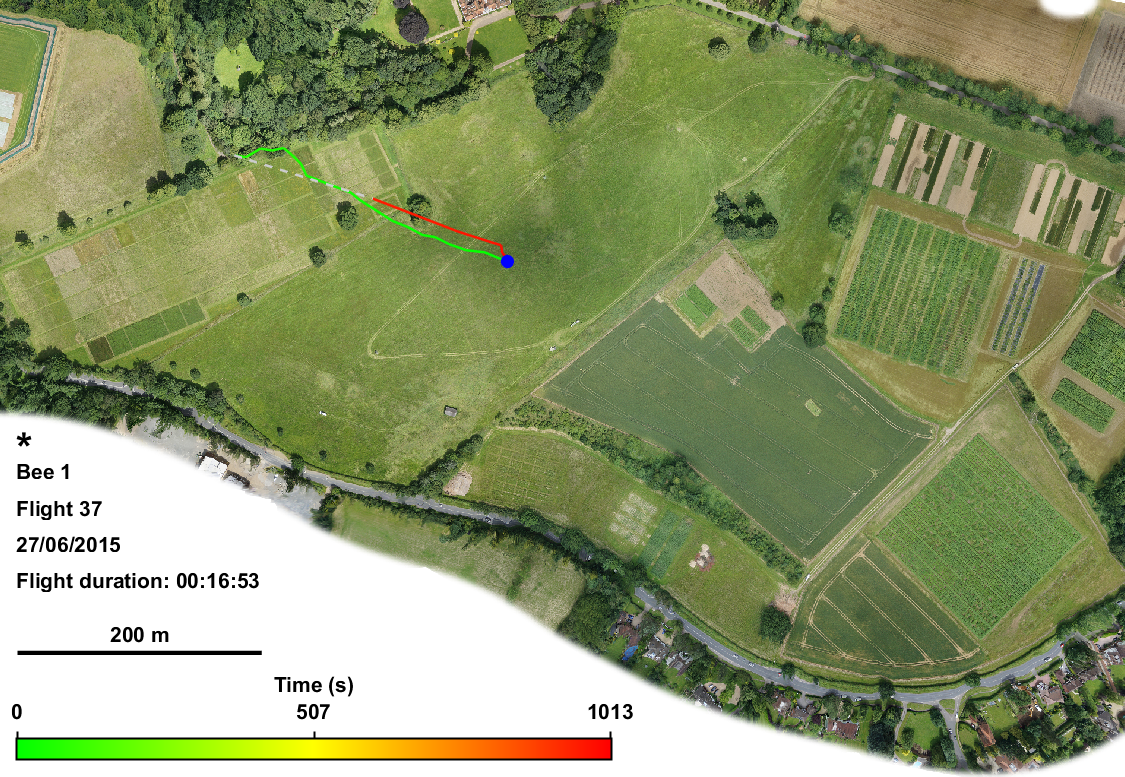

Supplement: S1 File — All flights made by Bee 1 during the period 25/06/2015–27/06/2015. Each figure represents a single flight. Bee ID, flight number, date of recording and the duration of each flight are shown in the bottom left corner of each figure. The position of the nest is marked by a blue circle. Colours represent the time from the start of each flight: initial portion of each flight is in green, changing smoothly through yellow until the end of the flight is shown in red. Grey dashed lines are used to join radar observations made more than 30 s apart, when the bee’s location was uncertain. (ZIP) [file pone.0160333.s003.zip › S38 Fig.tif]

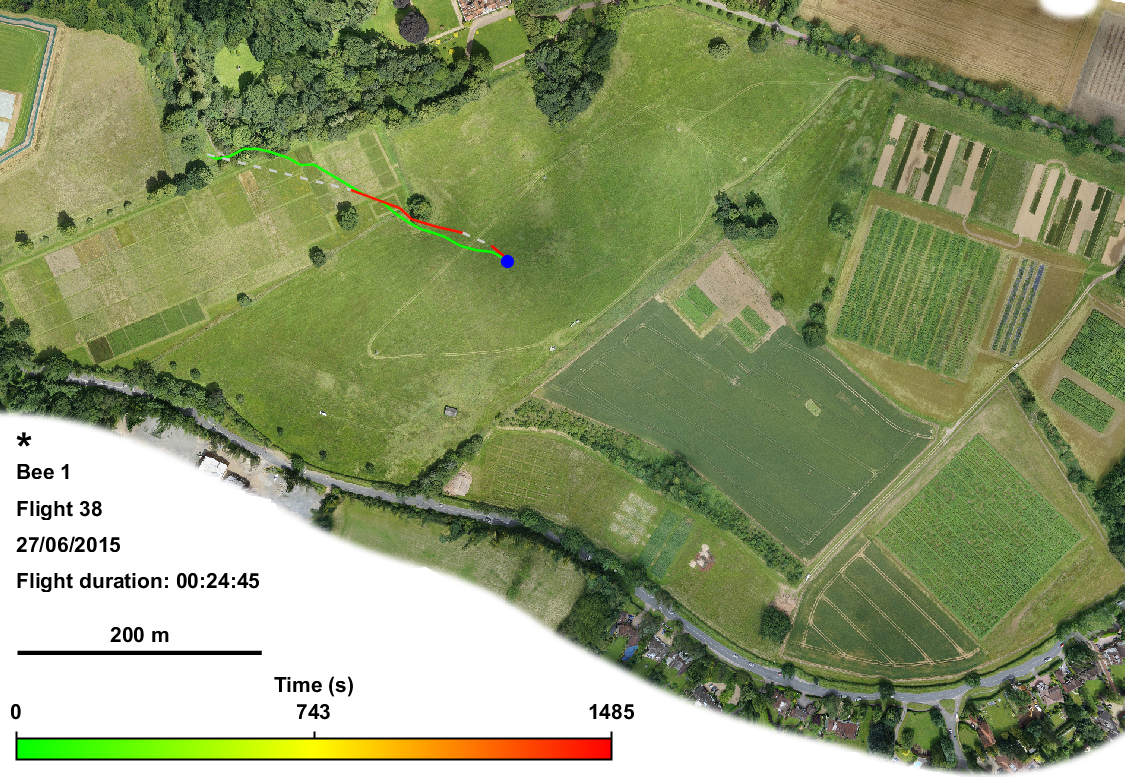

Supplement: S1 File — All flights made by Bee 1 during the period 25/06/2015–27/06/2015. Each figure represents a single flight. Bee ID, flight number, date of recording and the duration of each flight are shown in the bottom left corner of each figure. The position of the nest is marked by a blue circle. Colours represent the time from the start of each flight: initial portion of each flight is in green, changing smoothly through yellow until the end of the flight is shown in red. Grey dashed lines are used to join radar observations made more than 30 s apart, when the bee’s location was uncertain. (ZIP) [file pone.0160333.s003.zip › S39 Fig.tif]

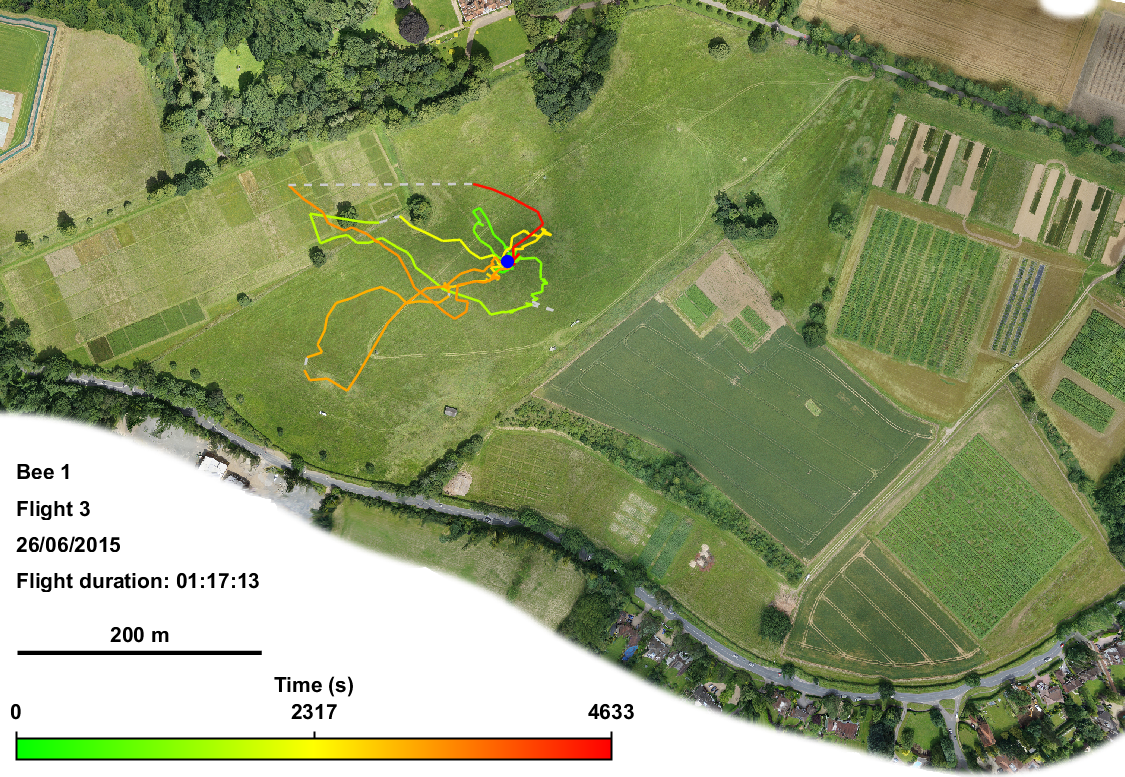

Supplement: S1 File — All flights made by Bee 1 during the period 25/06/2015–27/06/2015. Each figure represents a single flight. Bee ID, flight number, date of recording and the duration of each flight are shown in the bottom left corner of each figure. The position of the nest is marked by a blue circle. Colours represent the time from the start of each flight: initial portion of each flight is in green, changing smoothly through yellow until the end of the flight is shown in red. Grey dashed lines are used to join radar observations made more than 30 s apart, when the bee’s location was uncertain. (ZIP) [file pone.0160333.s003.zip › S4 Fig.tif]

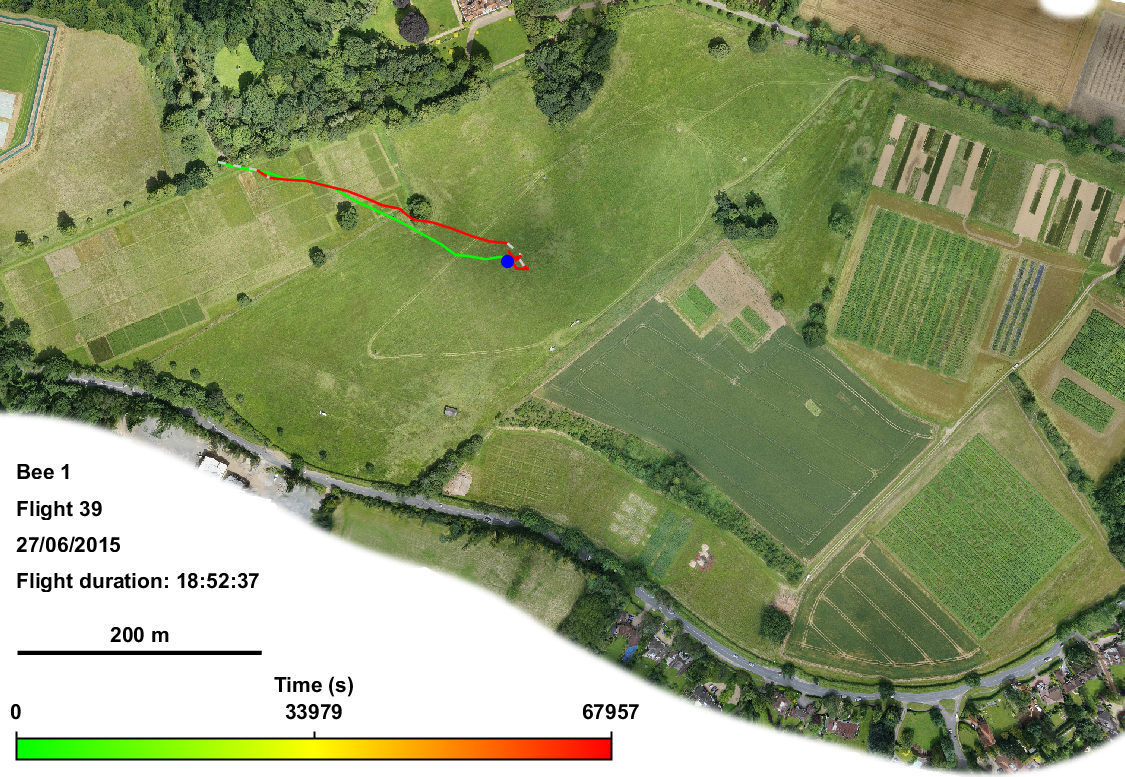

Supplement: S1 File — All flights made by Bee 1 during the period 25/06/2015–27/06/2015. Each figure represents a single flight. Bee ID, flight number, date of recording and the duration of each flight are shown in the bottom left corner of each figure. The position of the nest is marked by a blue circle. Colours represent the time from the start of each flight: initial portion of each flight is in green, changing smoothly through yellow until the end of the flight is shown in red. Grey dashed lines are used to join radar observations made more than 30 s apart, when the bee’s location was uncertain. (ZIP) [file pone.0160333.s003.zip › S40 Fig.tif]

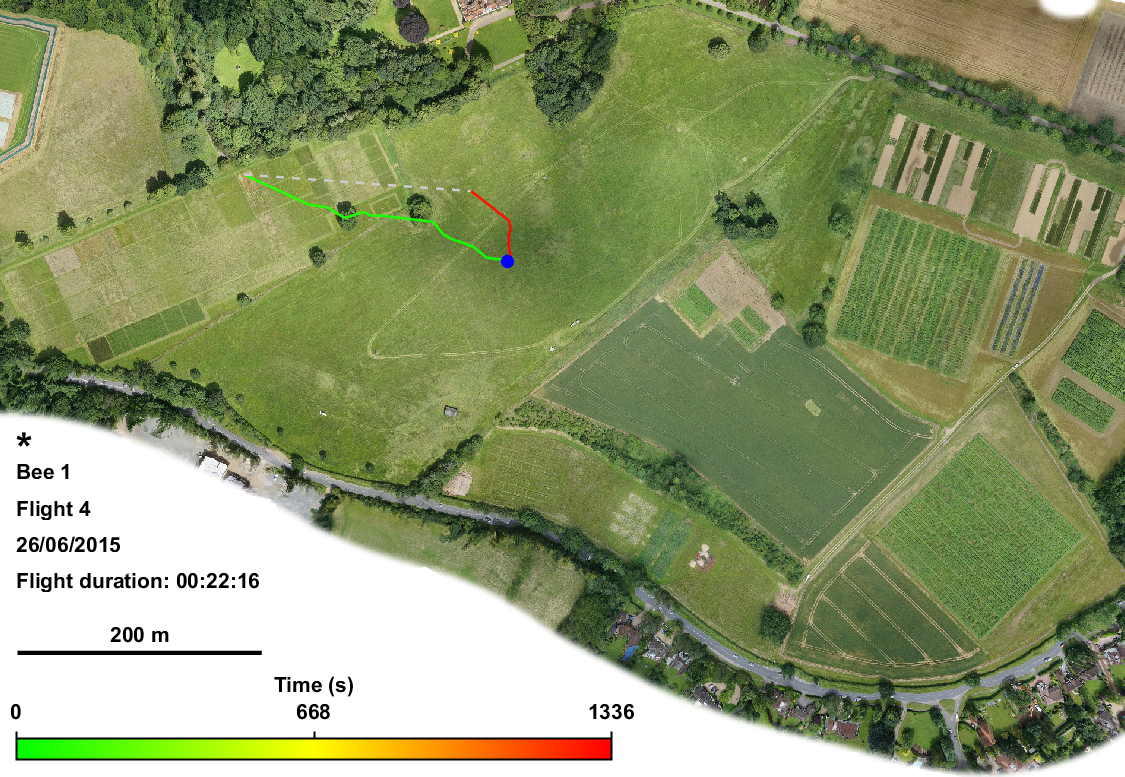

Supplement: S1 File — All flights made by Bee 1 during the period 25/06/2015–27/06/2015. Each figure represents a single flight. Bee ID, flight number, date of recording and the duration of each flight are shown in the bottom left corner of each figure. The position of the nest is marked by a blue circle. Colours represent the time from the start of each flight: initial portion of each flight is in green, changing smoothly through yellow until the end of the flight is shown in red. Grey dashed lines are used to join radar observations made more than 30 s apart, when the bee’s location was uncertain. (ZIP) [file pone.0160333.s003.zip › S5 Fig.tif]

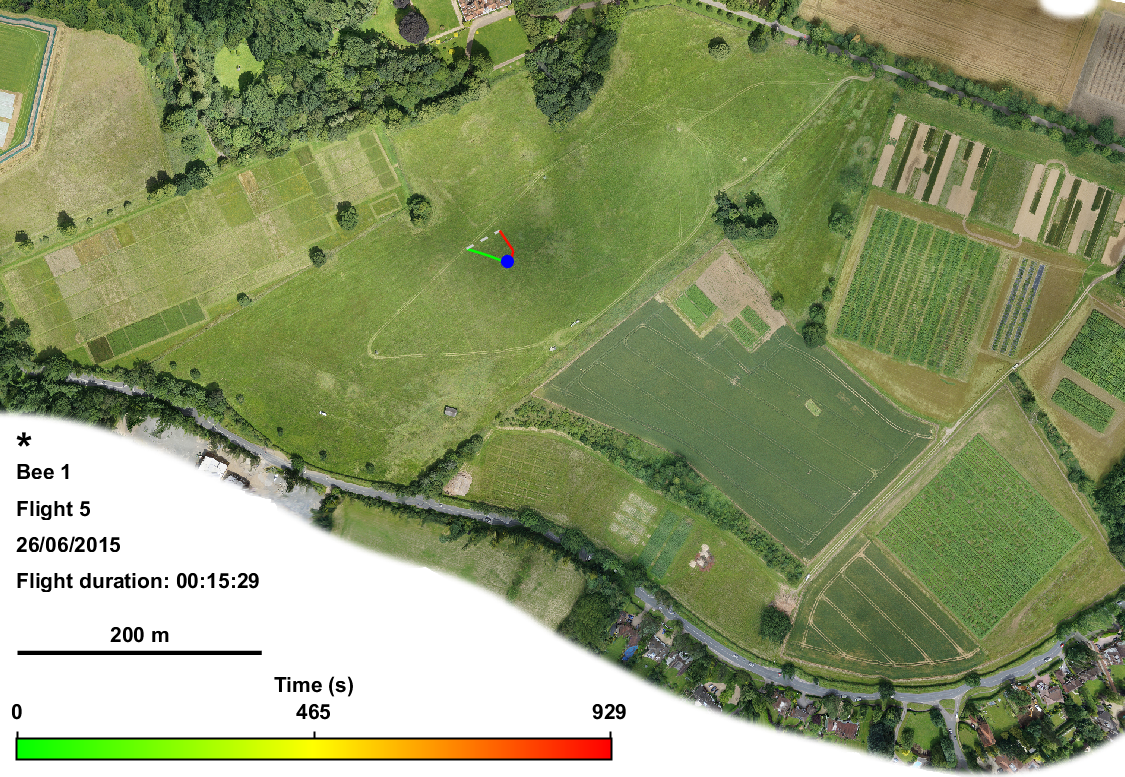

Supplement: S1 File — All flights made by Bee 1 during the period 25/06/2015–27/06/2015. Each figure represents a single flight. Bee ID, flight number, date of recording and the duration of each flight are shown in the bottom left corner of each figure. The position of the nest is marked by a blue circle. Colours represent the time from the start of each flight: initial portion of each flight is in green, changing smoothly through yellow until the end of the flight is shown in red. Grey dashed lines are used to join radar observations made more than 30 s apart, when the bee’s location was uncertain. (ZIP) [file pone.0160333.s003.zip › S6 Fig.tif]

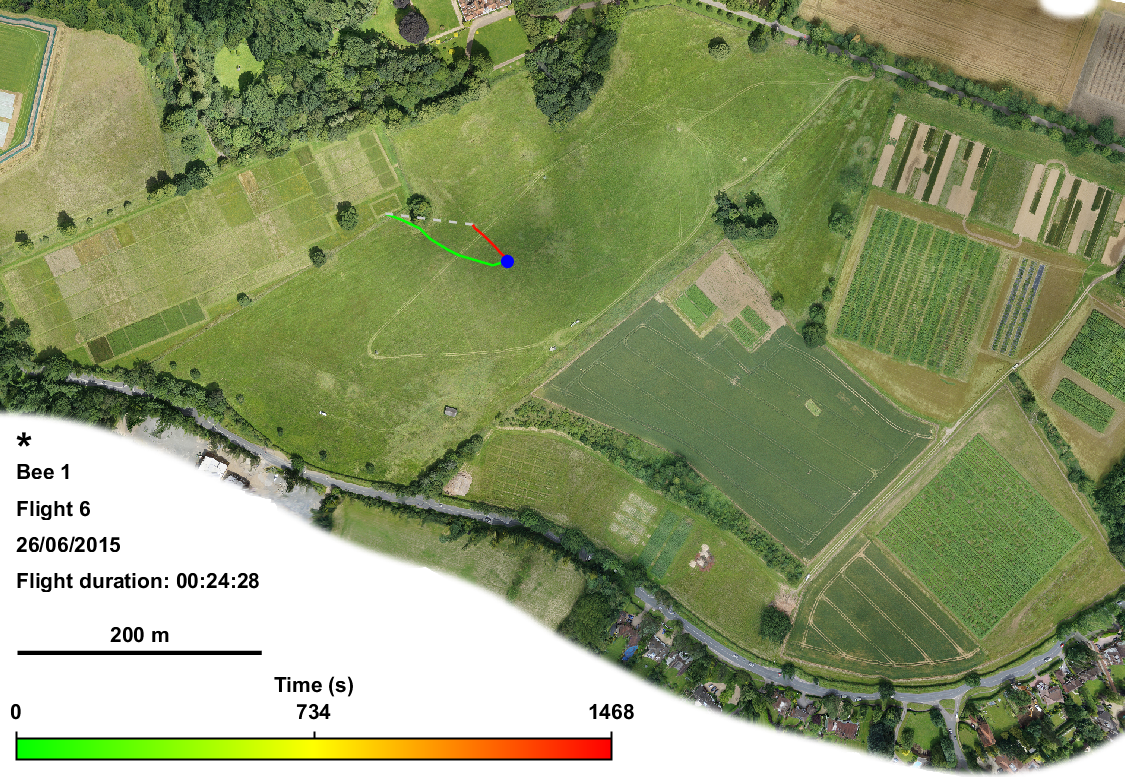

Supplement: S1 File — All flights made by Bee 1 during the period 25/06/2015–27/06/2015. Each figure represents a single flight. Bee ID, flight number, date of recording and the duration of each flight are shown in the bottom left corner of each figure. The position of the nest is marked by a blue circle. Colours represent the time from the start of each flight: initial portion of each flight is in green, changing smoothly through yellow until the end of the flight is shown in red. Grey dashed lines are used to join radar observations made more than 30 s apart, when the bee’s location was uncertain. (ZIP) [file pone.0160333.s003.zip › S7 Fig.tif]

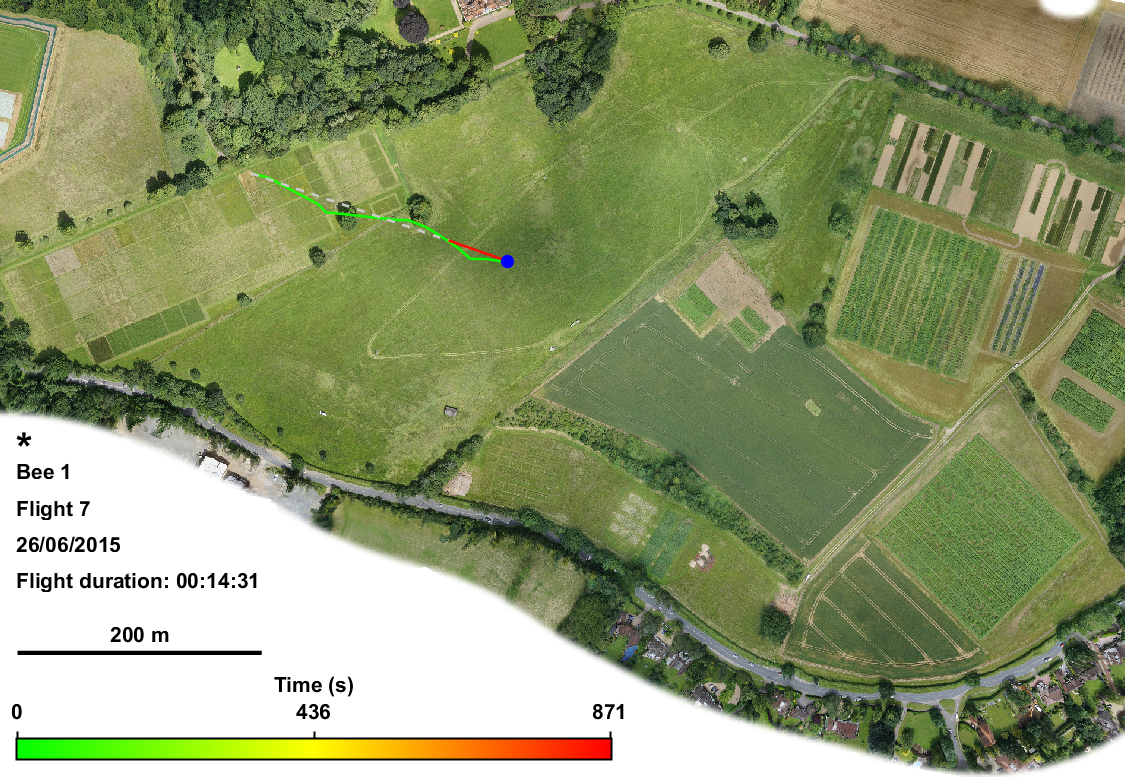

Supplement: S1 File — All flights made by Bee 1 during the period 25/06/2015–27/06/2015. Each figure represents a single flight. Bee ID, flight number, date of recording and the duration of each flight are shown in the bottom left corner of each figure. The position of the nest is marked by a blue circle. Colours represent the time from the start of each flight: initial portion of each flight is in green, changing smoothly through yellow until the end of the flight is shown in red. Grey dashed lines are used to join radar observations made more than 30 s apart, when the bee’s location was uncertain. (ZIP) [file pone.0160333.s003.zip › S8 Fig.tif]

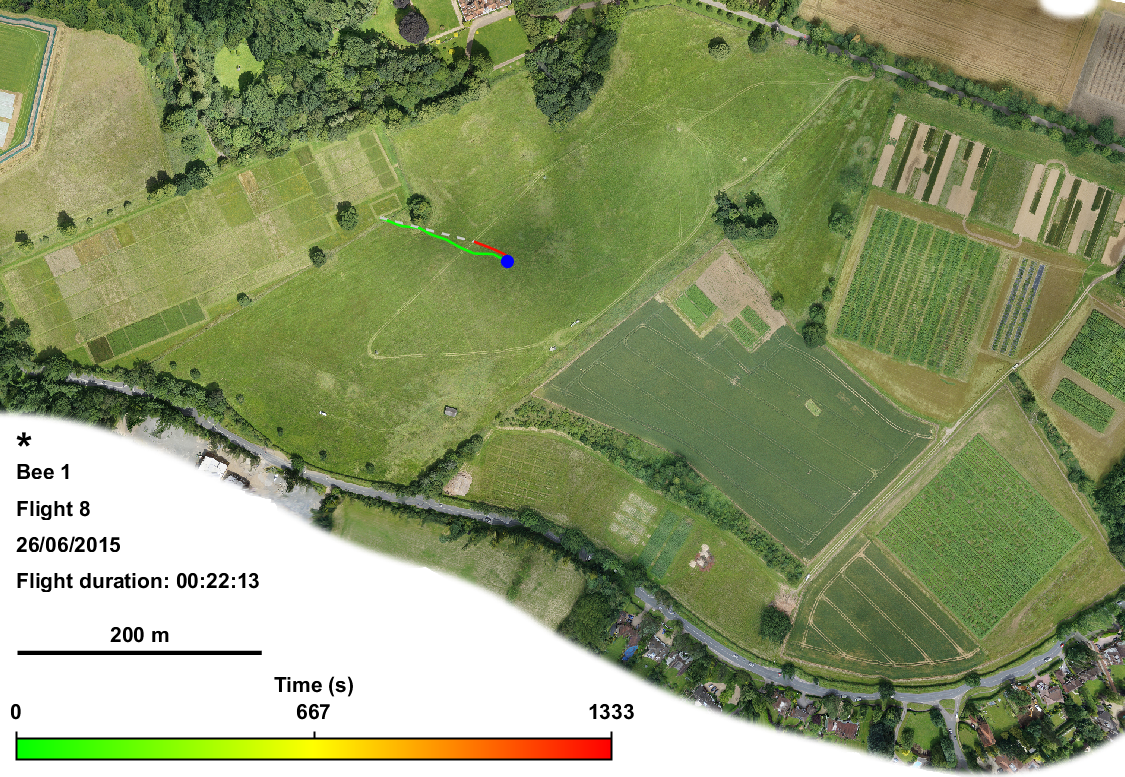

Supplement: S1 File — All flights made by Bee 1 during the period 25/06/2015–27/06/2015. Each figure represents a single flight. Bee ID, flight number, date of recording and the duration of each flight are shown in the bottom left corner of each figure. The position of the nest is marked by a blue circle. Colours represent the time from the start of each flight: initial portion of each flight is in green, changing smoothly through yellow until the end of the flight is shown in red. Grey dashed lines are used to join radar observations made more than 30 s apart, when the bee’s location was uncertain. (ZIP) [file pone.0160333.s003.zip › S9 Fig.tif]

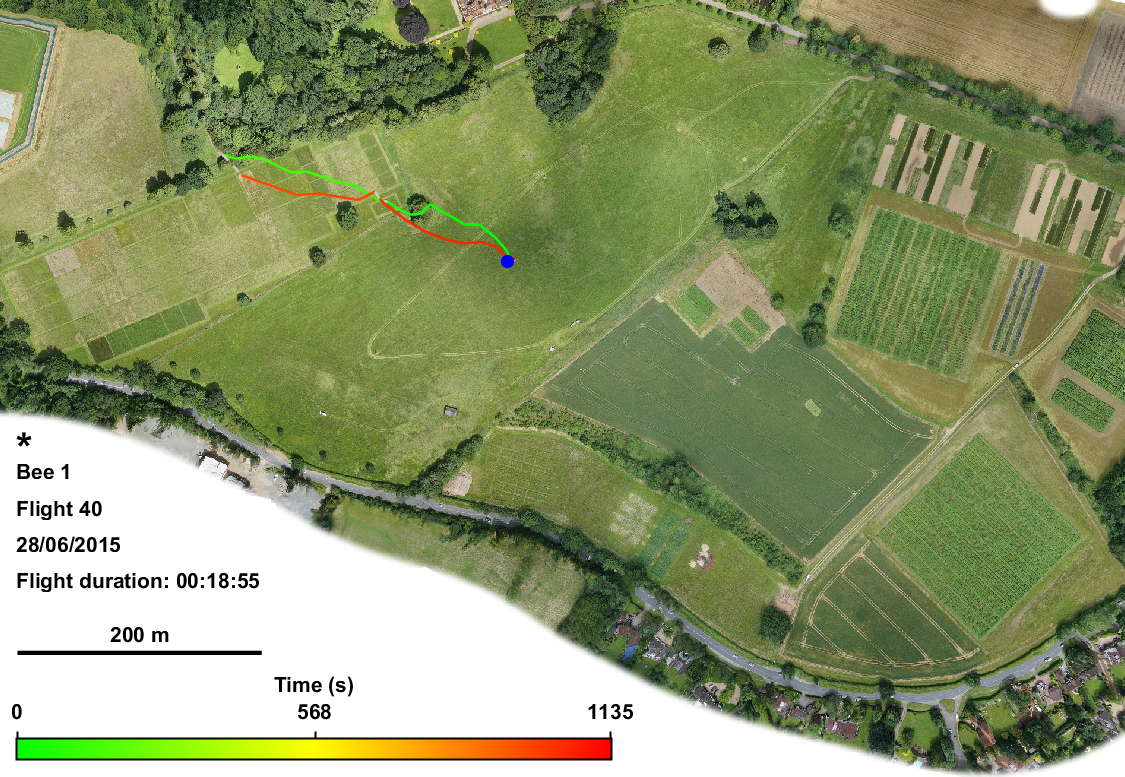

Supplement: S2 File — All flights made by Bee 1 during the period 28/06/2015–03/07/2015. Each figure represents a single flight. Bee ID, flight number, date of recording and the duration of each flight are shown in the bottom left corner of each figure. The position of the nest is marked by a blue circle. Colours represent the time from the start of each flight: initial portion of each flight is in green, changing smoothly through yellow until the end of the flight is shown in red. Grey dashed lines are used to join radar observations made more than 30 s apart, when the bee’s location was uncertain. (ZIP) [file pone.0160333.s004.zip › S41 Fig.tif]

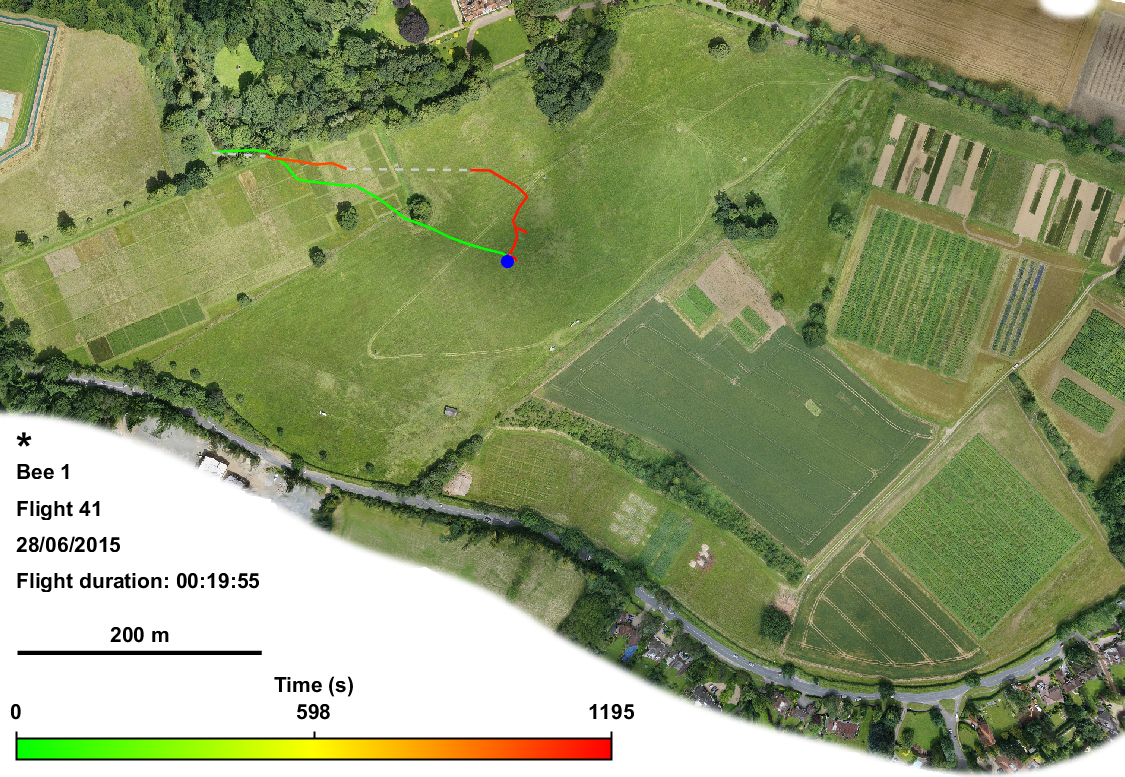

Supplement: S2 File — All flights made by Bee 1 during the period 28/06/2015–03/07/2015. Each figure represents a single flight. Bee ID, flight number, date of recording and the duration of each flight are shown in the bottom left corner of each figure. The position of the nest is marked by a blue circle. Colours represent the time from the start of each flight: initial portion of each flight is in green, changing smoothly through yellow until the end of the flight is shown in red. Grey dashed lines are used to join radar observations made more than 30 s apart, when the bee’s location was uncertain. (ZIP) [file pone.0160333.s004.zip › S42 Fig.tif]

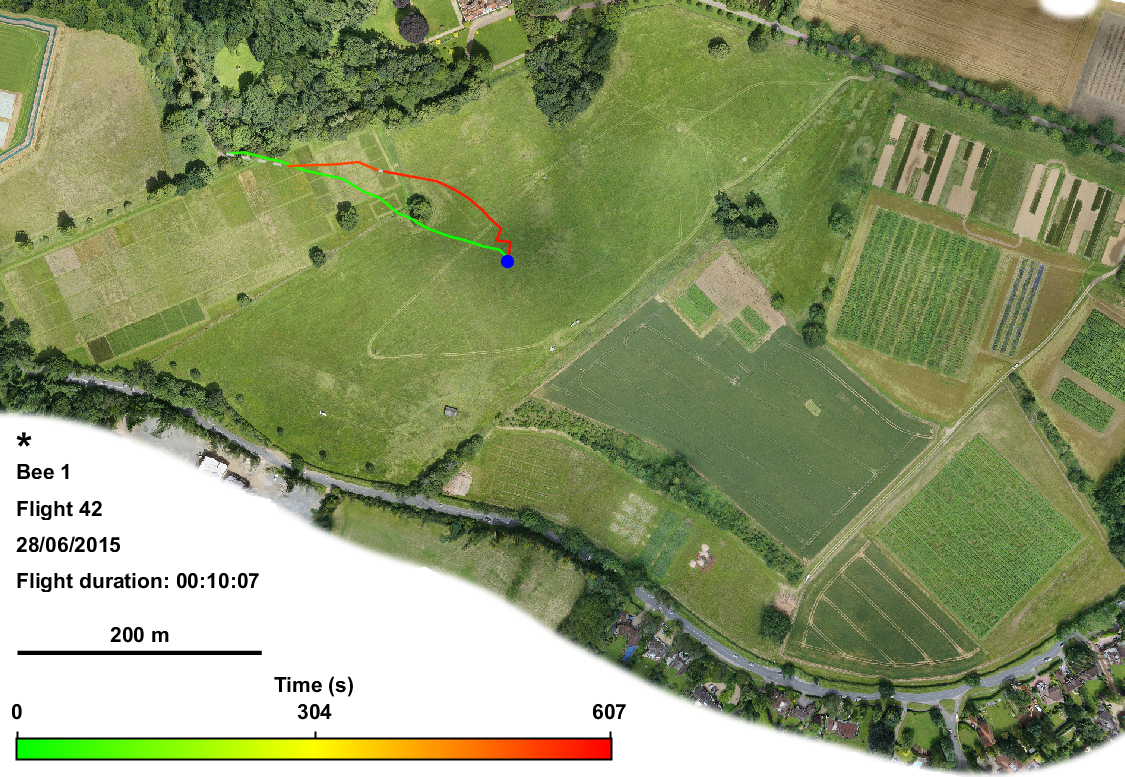

Supplement: S2 File — All flights made by Bee 1 during the period 28/06/2015–03/07/2015. Each figure represents a single flight. Bee ID, flight number, date of recording and the duration of each flight are shown in the bottom left corner of each figure. The position of the nest is marked by a blue circle. Colours represent the time from the start of each flight: initial portion of each flight is in green, changing smoothly through yellow until the end of the flight is shown in red. Grey dashed lines are used to join radar observations made more than 30 s apart, when the bee’s location was uncertain. (ZIP) [file pone.0160333.s004.zip › S43 Fig.tif]

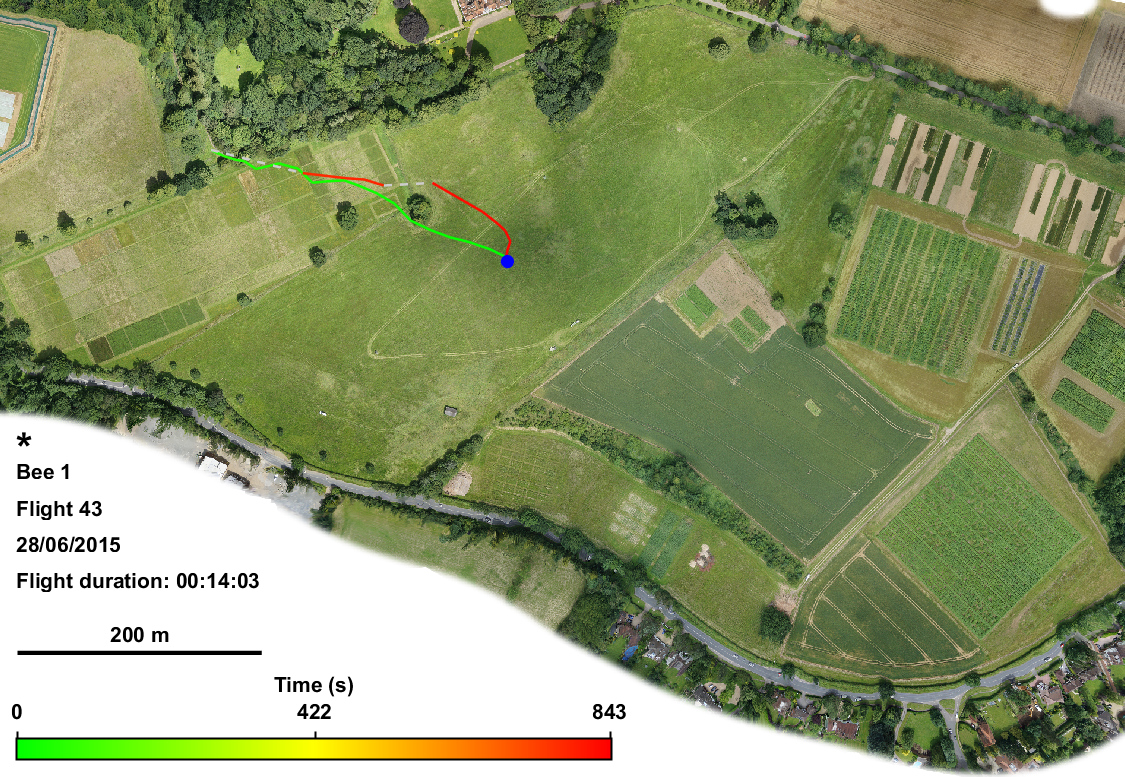

Supplement: S2 File — All flights made by Bee 1 during the period 28/06/2015–03/07/2015. Each figure represents a single flight. Bee ID, flight number, date of recording and the duration of each flight are shown in the bottom left corner of each figure. The position of the nest is marked by a blue circle. Colours represent the time from the start of each flight: initial portion of each flight is in green, changing smoothly through yellow until the end of the flight is shown in red. Grey dashed lines are used to join radar observations made more than 30 s apart, when the bee’s location was uncertain. (ZIP) [file pone.0160333.s004.zip › S44 Fig.tif]

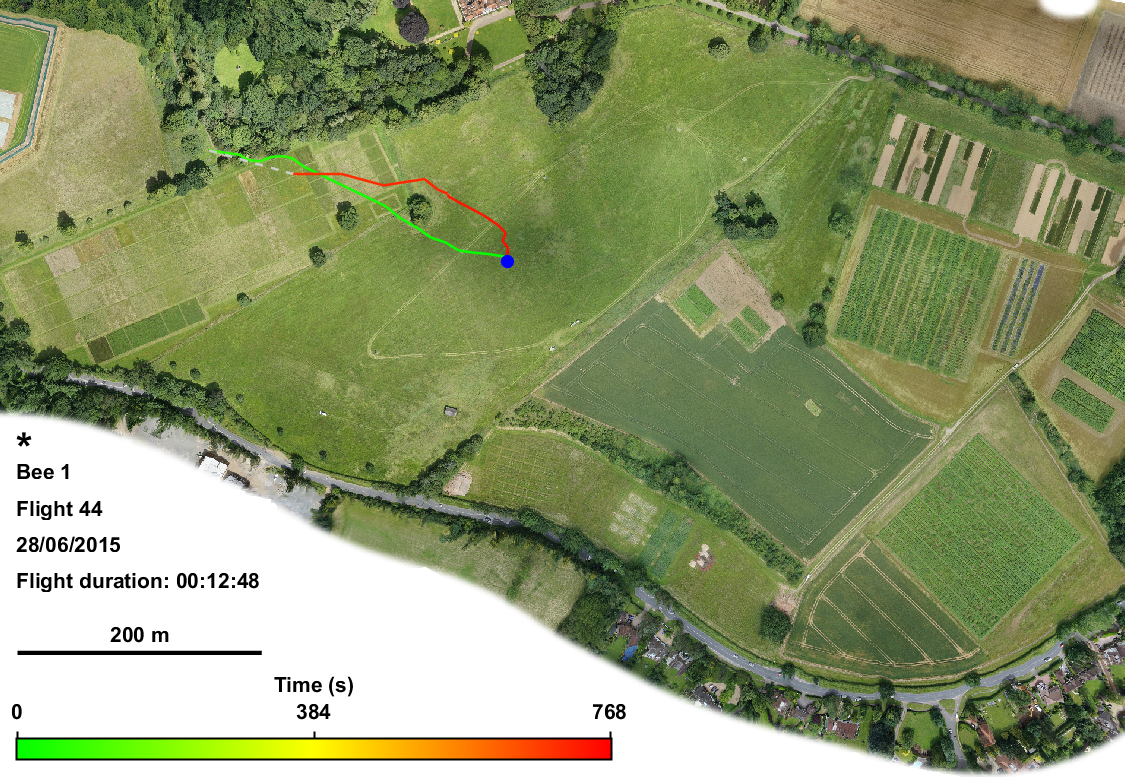

Supplement: S2 File — All flights made by Bee 1 during the period 28/06/2015–03/07/2015. Each figure represents a single flight. Bee ID, flight number, date of recording and the duration of each flight are shown in the bottom left corner of each figure. The position of the nest is marked by a blue circle. Colours represent the time from the start of each flight: initial portion of each flight is in green, changing smoothly through yellow until the end of the flight is shown in red. Grey dashed lines are used to join radar observations made more than 30 s apart, when the bee’s location was uncertain. (ZIP) [file pone.0160333.s004.zip › S45 Fig.tif]

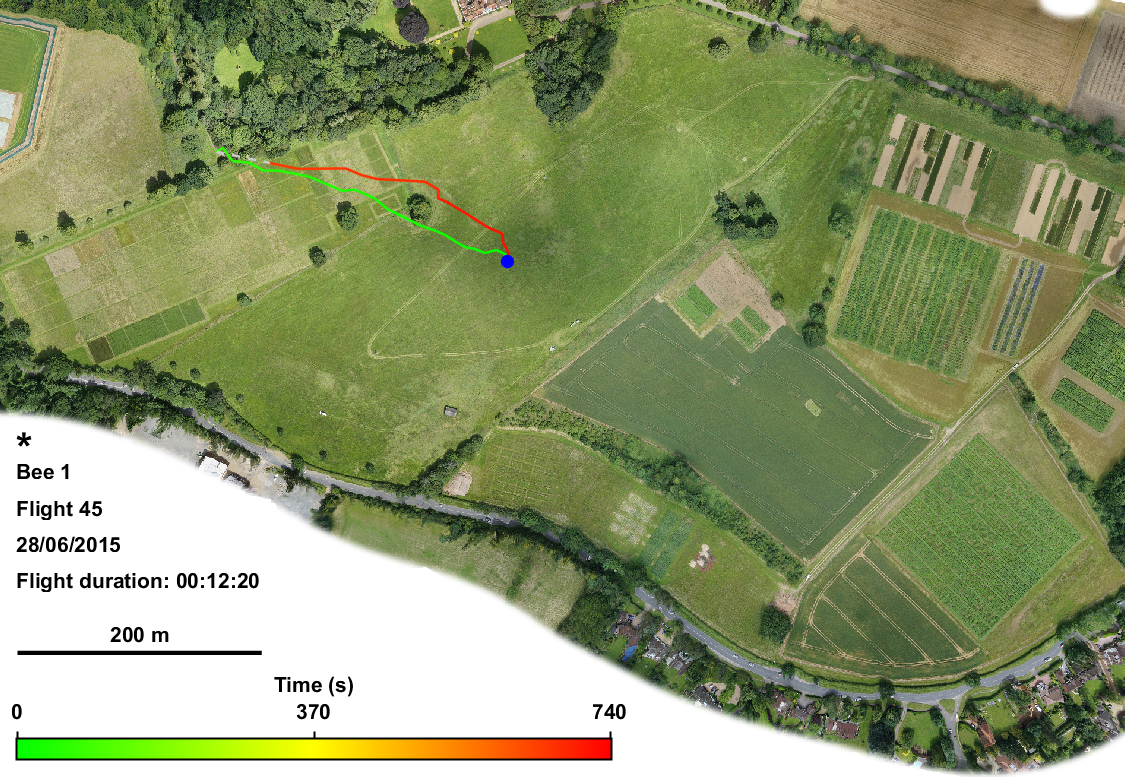

Supplement: S2 File — All flights made by Bee 1 during the period 28/06/2015–03/07/2015. Each figure represents a single flight. Bee ID, flight number, date of recording and the duration of each flight are shown in the bottom left corner of each figure. The position of the nest is marked by a blue circle. Colours represent the time from the start of each flight: initial portion of each flight is in green, changing smoothly through yellow until the end of the flight is shown in red. Grey dashed lines are used to join radar observations made more than 30 s apart, when the bee’s location was uncertain. (ZIP) [file pone.0160333.s004.zip › S46 Fig.tif]

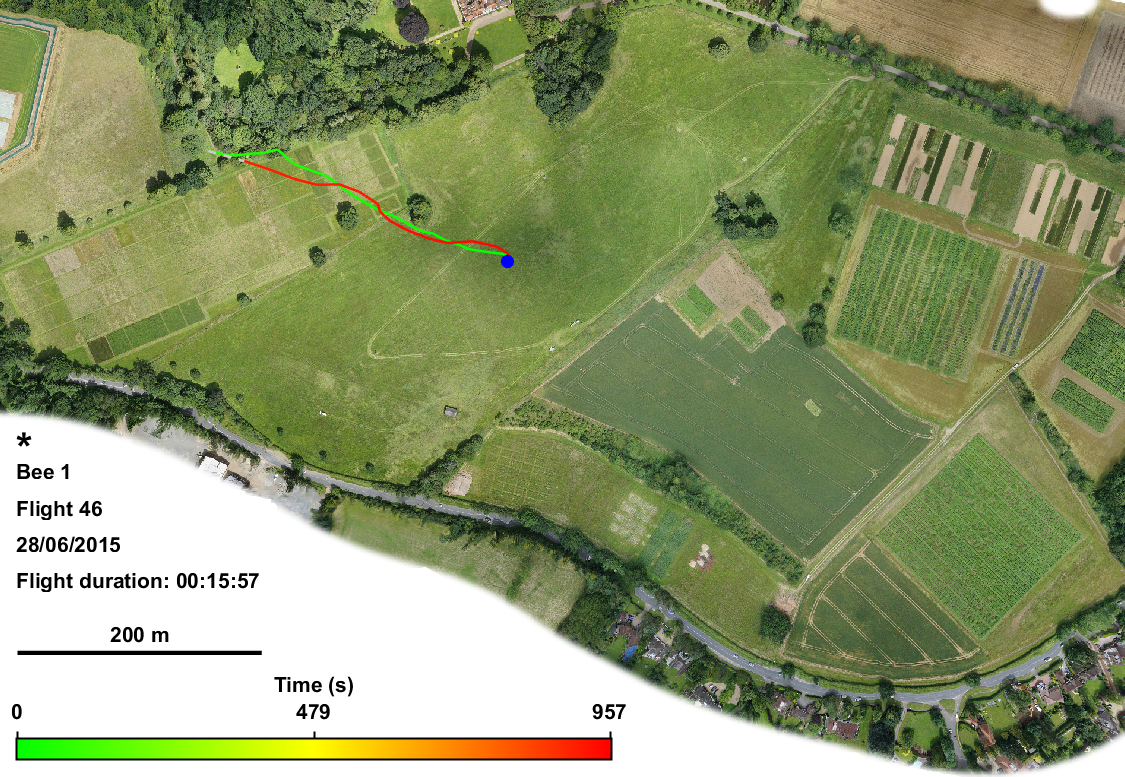

Supplement: S2 File — All flights made by Bee 1 during the period 28/06/2015–03/07/2015. Each figure represents a single flight. Bee ID, flight number, date of recording and the duration of each flight are shown in the bottom left corner of each figure. The position of the nest is marked by a blue circle. Colours represent the time from the start of each flight: initial portion of each flight is in green, changing smoothly through yellow until the end of the flight is shown in red. Grey dashed lines are used to join radar observations made more than 30 s apart, when the bee’s location was uncertain. (ZIP) [file pone.0160333.s004.zip › S47 Fig.tif]

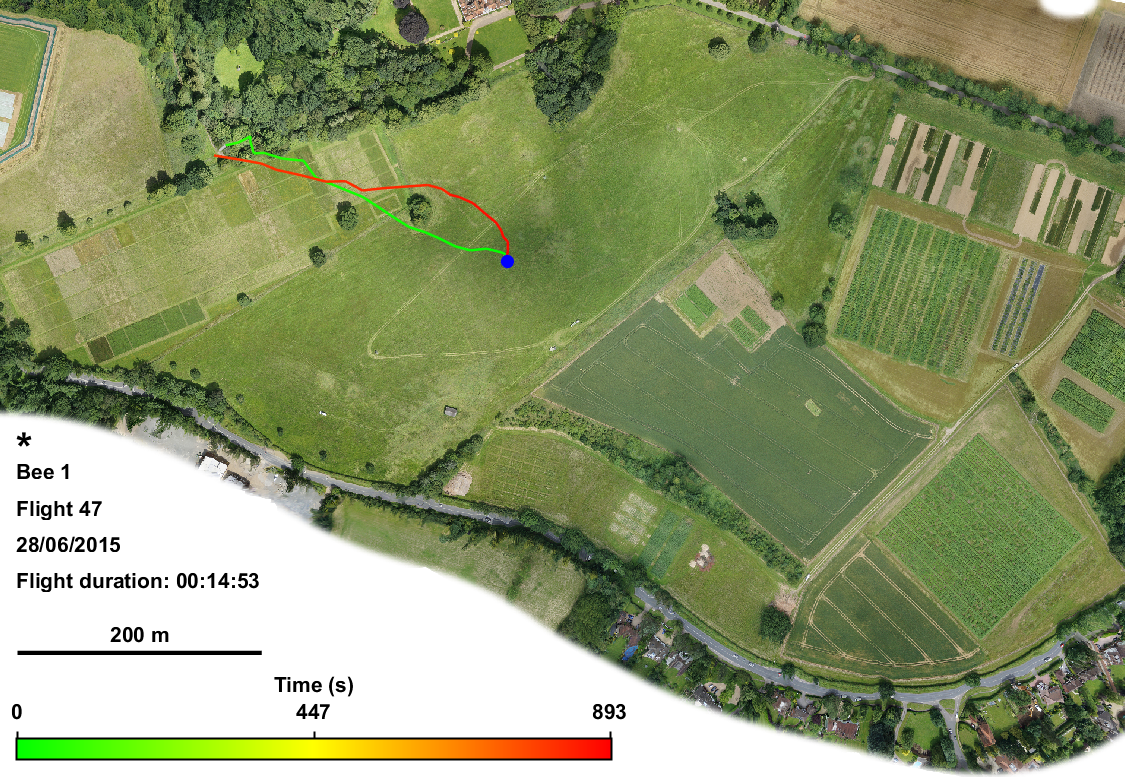

Supplement: S2 File — All flights made by Bee 1 during the period 28/06/2015–03/07/2015. Each figure represents a single flight. Bee ID, flight number, date of recording and the duration of each flight are shown in the bottom left corner of each figure. The position of the nest is marked by a blue circle. Colours represent the time from the start of each flight: initial portion of each flight is in green, changing smoothly through yellow until the end of the flight is shown in red. Grey dashed lines are used to join radar observations made more than 30 s apart, when the bee’s location was uncertain. (ZIP) [file pone.0160333.s004.zip › S48 Fig.tif]

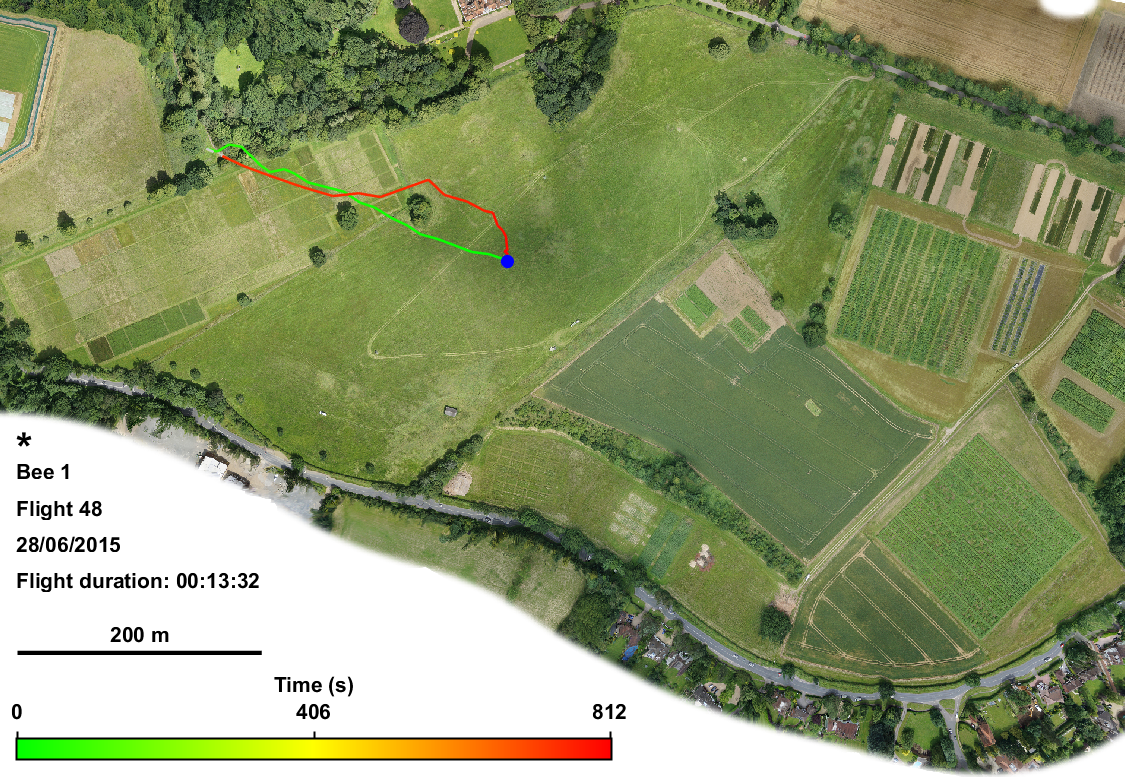

Supplement: S2 File — All flights made by Bee 1 during the period 28/06/2015–03/07/2015. Each figure represents a single flight. Bee ID, flight number, date of recording and the duration of each flight are shown in the bottom left corner of each figure. The position of the nest is marked by a blue circle. Colours represent the time from the start of each flight: initial portion of each flight is in green, changing smoothly through yellow until the end of the flight is shown in red. Grey dashed lines are used to join radar observations made more than 30 s apart, when the bee’s location was uncertain. (ZIP) [file pone.0160333.s004.zip › S49 Fig.tif]

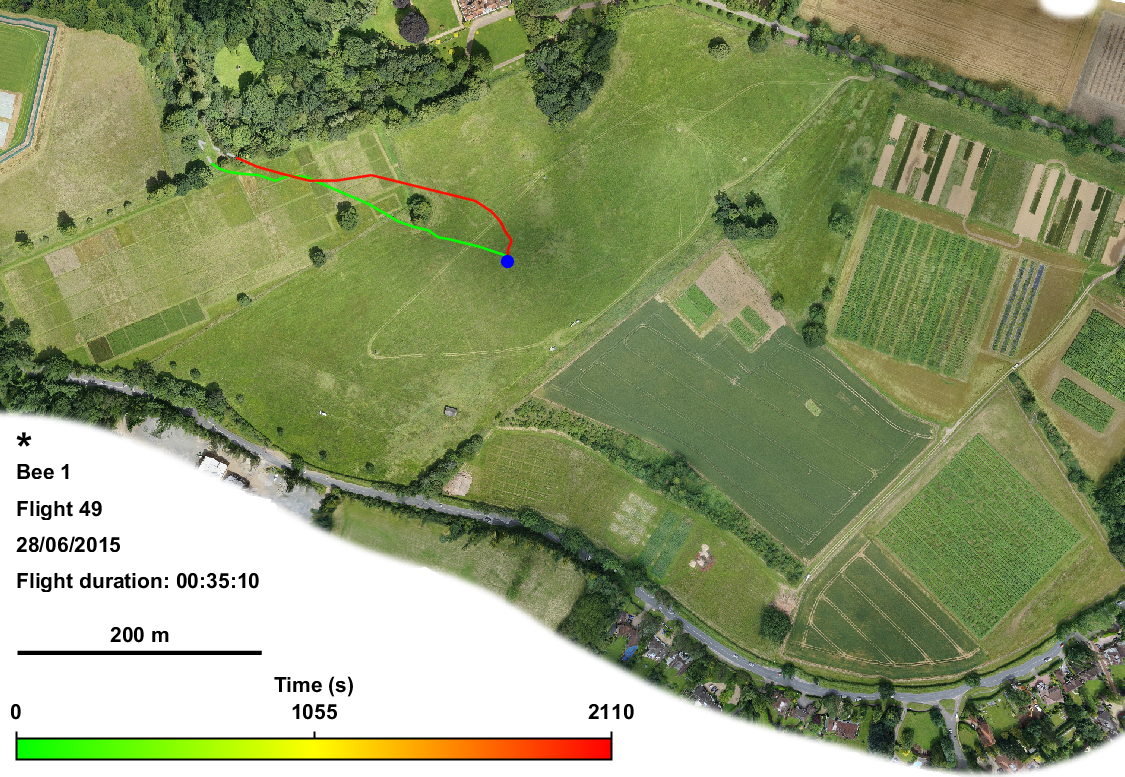

Supplement: S2 File — All flights made by Bee 1 during the period 28/06/2015–03/07/2015. Each figure represents a single flight. Bee ID, flight number, date of recording and the duration of each flight are shown in the bottom left corner of each figure. The position of the nest is marked by a blue circle. Colours represent the time from the start of each flight: initial portion of each flight is in green, changing smoothly through yellow until the end of the flight is shown in red. Grey dashed lines are used to join radar observations made more than 30 s apart, when the bee’s location was uncertain. (ZIP) [file pone.0160333.s004.zip › S50 Fig.tif]

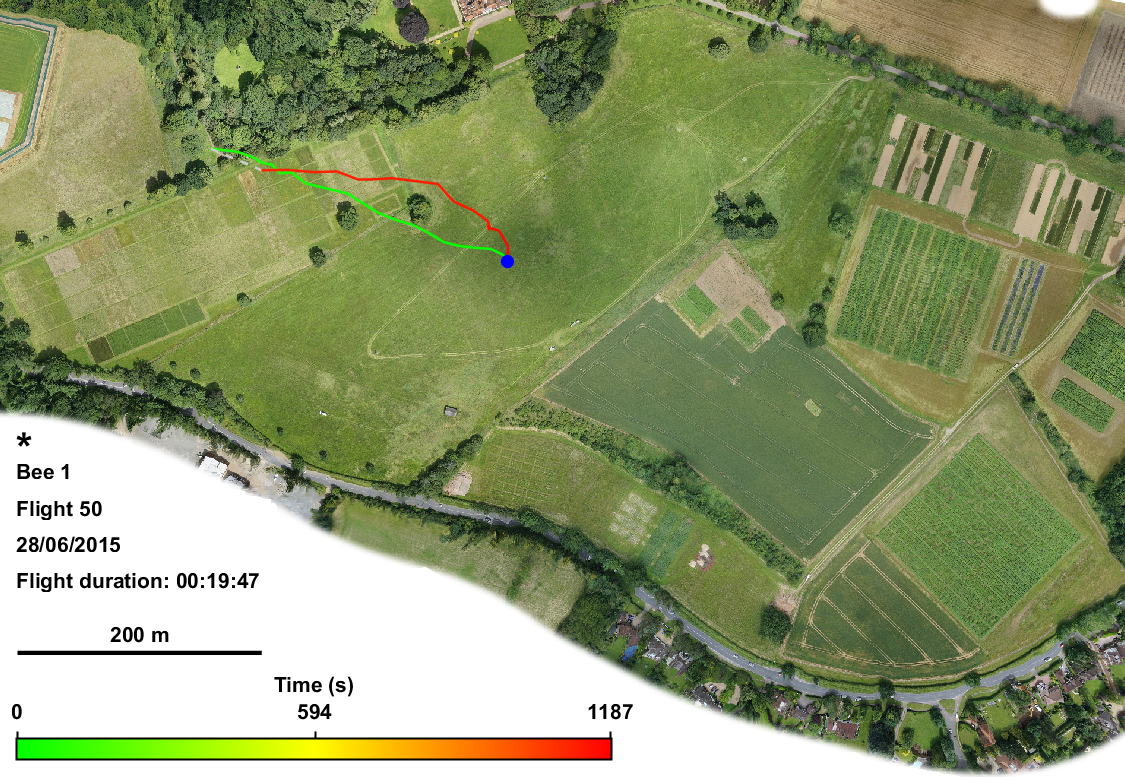

Supplement: S2 File — All flights made by Bee 1 during the period 28/06/2015–03/07/2015. Each figure represents a single flight. Bee ID, flight number, date of recording and the duration of each flight are shown in the bottom left corner of each figure. The position of the nest is marked by a blue circle. Colours represent the time from the start of each flight: initial portion of each flight is in green, changing smoothly through yellow until the end of the flight is shown in red. Grey dashed lines are used to join radar observations made more than 30 s apart, when the bee’s location was uncertain. (ZIP) [file pone.0160333.s004.zip › S51 Fig.tif]

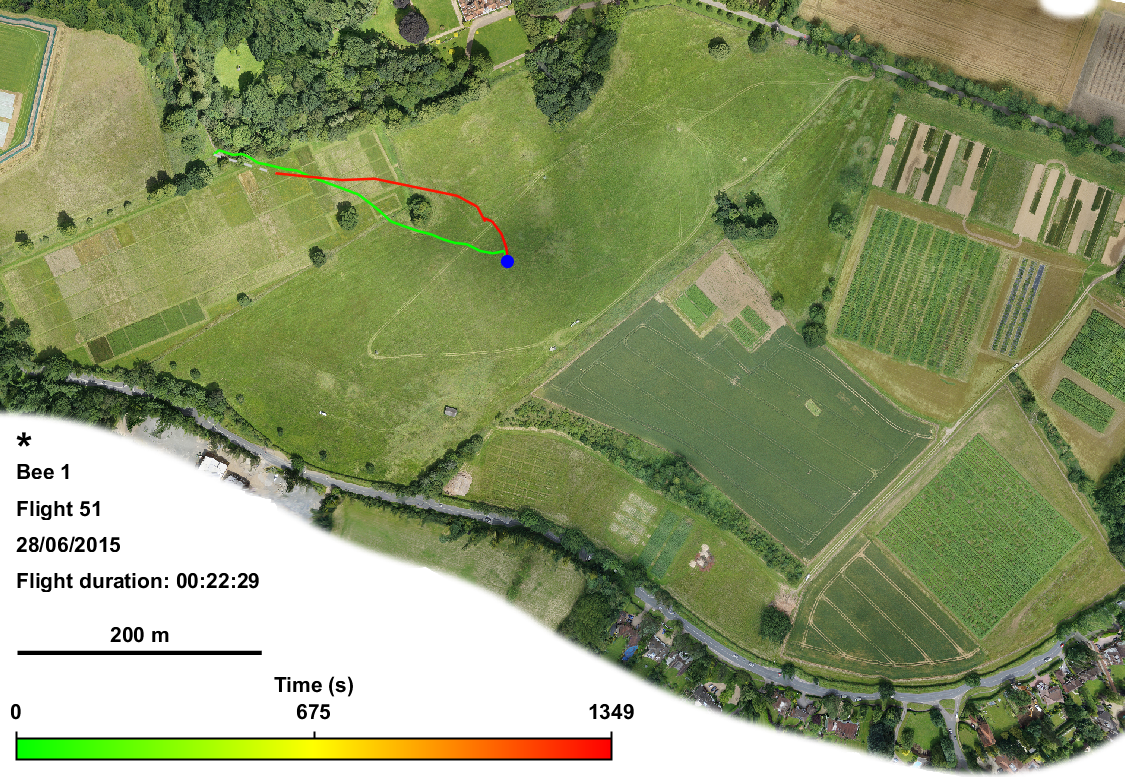

Supplement: S2 File — All flights made by Bee 1 during the period 28/06/2015–03/07/2015. Each figure represents a single flight. Bee ID, flight number, date of recording and the duration of each flight are shown in the bottom left corner of each figure. The position of the nest is marked by a blue circle. Colours represent the time from the start of each flight: initial portion of each flight is in green, changing smoothly through yellow until the end of the flight is shown in red. Grey dashed lines are used to join radar observations made more than 30 s apart, when the bee’s location was uncertain. (ZIP) [file pone.0160333.s004.zip › S52 Fig.tif]

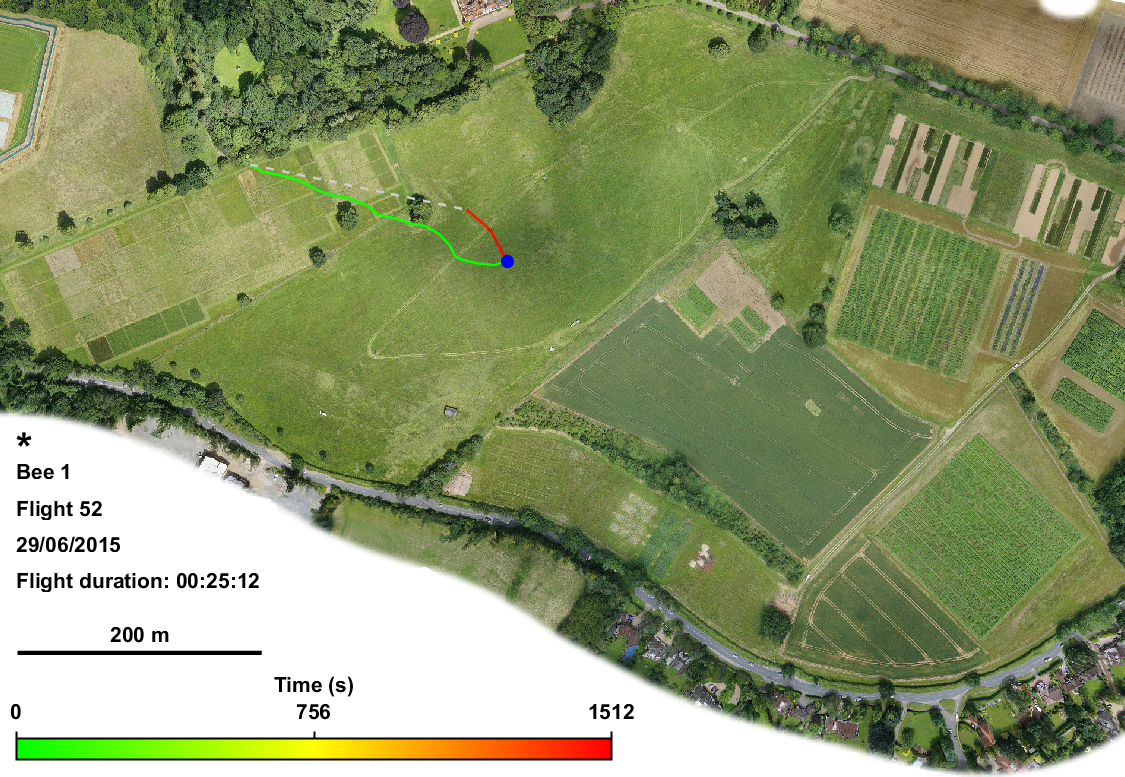

Supplement: S2 File — All flights made by Bee 1 during the period 28/06/2015–03/07/2015. Each figure represents a single flight. Bee ID, flight number, date of recording and the duration of each flight are shown in the bottom left corner of each figure. The position of the nest is marked by a blue circle. Colours represent the time from the start of each flight: initial portion of each flight is in green, changing smoothly through yellow until the end of the flight is shown in red. Grey dashed lines are used to join radar observations made more than 30 s apart, when the bee’s location was uncertain. (ZIP) [file pone.0160333.s004.zip › S53 Fig.tif]

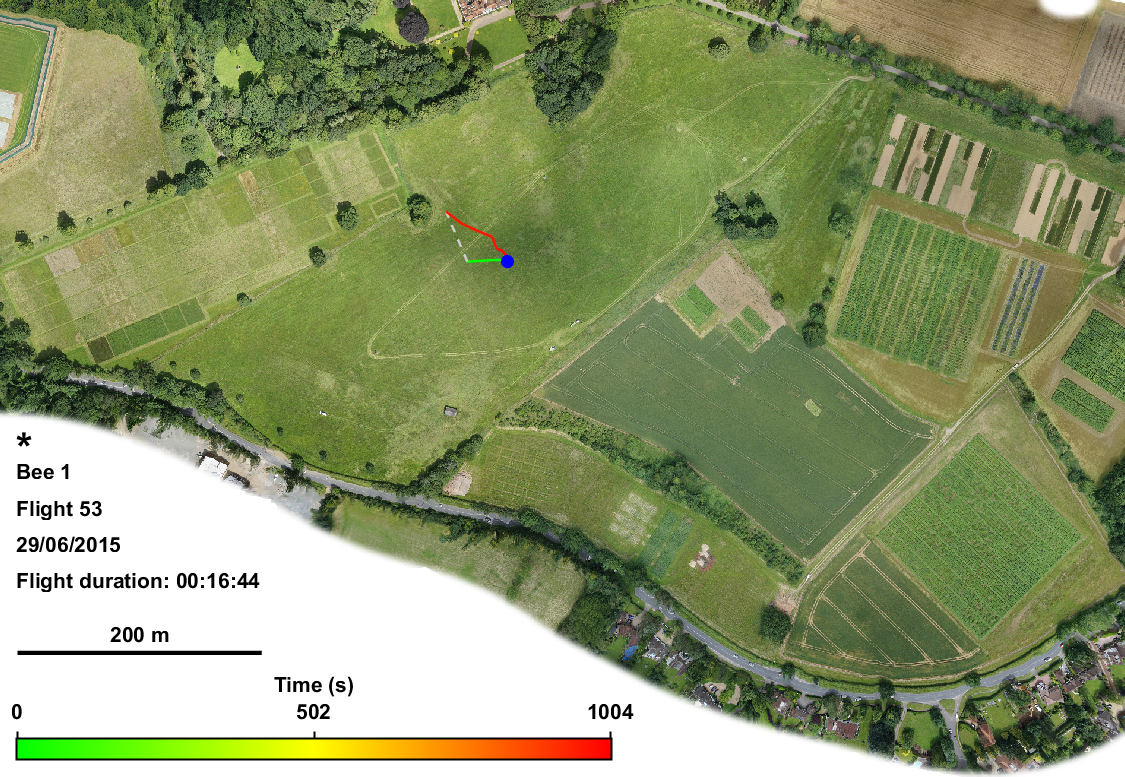

Supplement: S2 File — All flights made by Bee 1 during the period 28/06/2015–03/07/2015. Each figure represents a single flight. Bee ID, flight number, date of recording and the duration of each flight are shown in the bottom left corner of each figure. The position of the nest is marked by a blue circle. Colours represent the time from the start of each flight: initial portion of each flight is in green, changing smoothly through yellow until the end of the flight is shown in red. Grey dashed lines are used to join radar observations made more than 30 s apart, when the bee’s location was uncertain. (ZIP) [file pone.0160333.s004.zip › S54 Fig.tif]

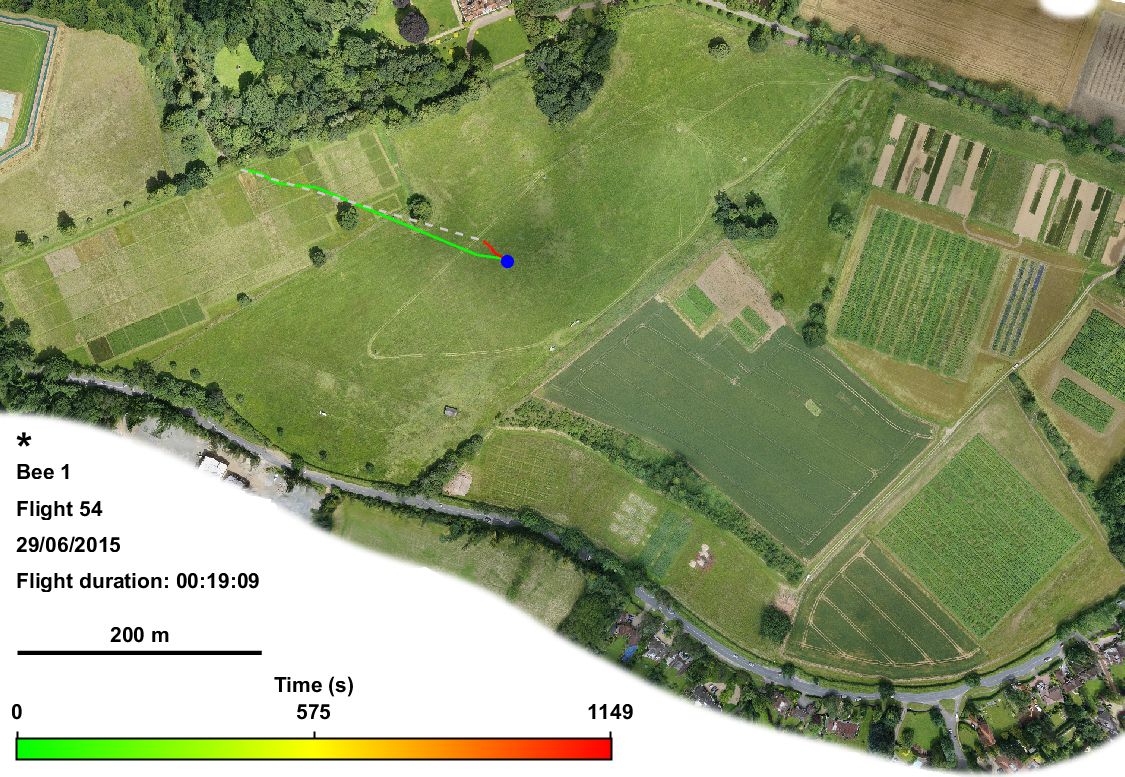

Supplement: S2 File — All flights made by Bee 1 during the period 28/06/2015–03/07/2015. Each figure represents a single flight. Bee ID, flight number, date of recording and the duration of each flight are shown in the bottom left corner of each figure. The position of the nest is marked by a blue circle. Colours represent the time from the start of each flight: initial portion of each flight is in green, changing smoothly through yellow until the end of the flight is shown in red. Grey dashed lines are used to join radar observations made more than 30 s apart, when the bee’s location was uncertain. (ZIP) [file pone.0160333.s004.zip › S55 Fig.tif]

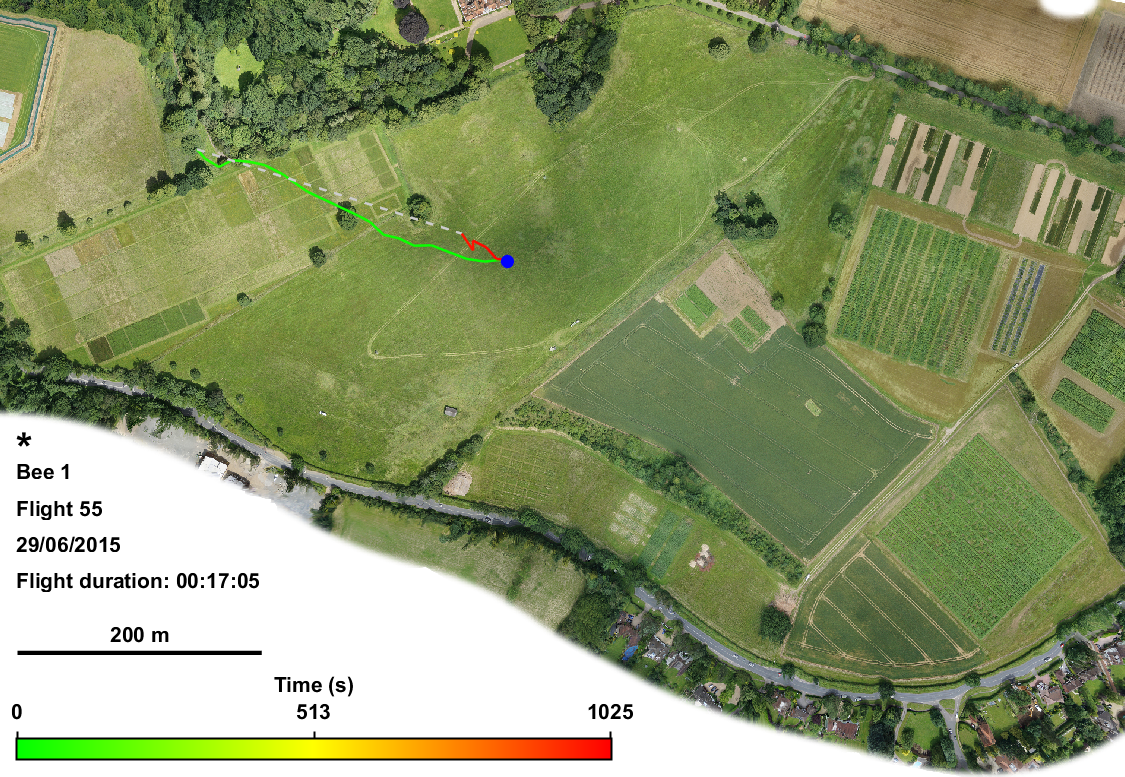

Supplement: S2 File — All flights made by Bee 1 during the period 28/06/2015–03/07/2015. Each figure represents a single flight. Bee ID, flight number, date of recording and the duration of each flight are shown in the bottom left corner of each figure. The position of the nest is marked by a blue circle. Colours represent the time from the start of each flight: initial portion of each flight is in green, changing smoothly through yellow until the end of the flight is shown in red. Grey dashed lines are used to join radar observations made more than 30 s apart, when the bee’s location was uncertain. (ZIP) [file pone.0160333.s004.zip › S56 Fig.tif]

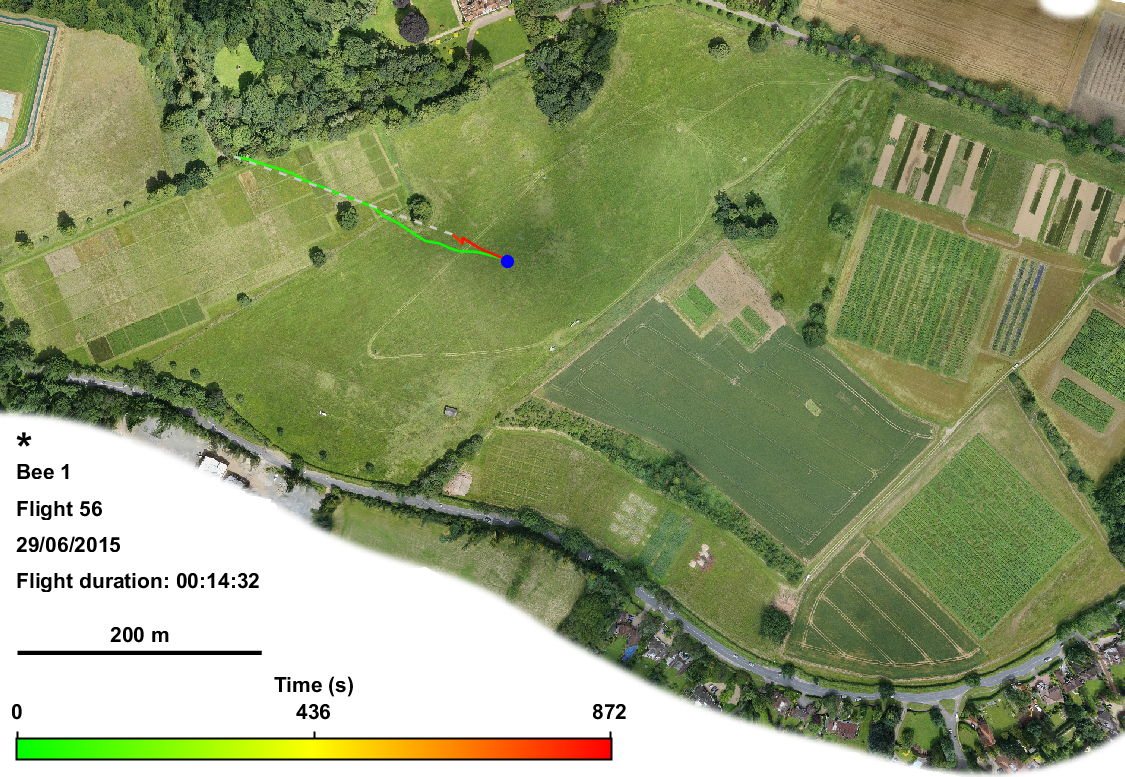

Supplement: S2 File — All flights made by Bee 1 during the period 28/06/2015–03/07/2015. Each figure represents a single flight. Bee ID, flight number, date of recording and the duration of each flight are shown in the bottom left corner of each figure. The position of the nest is marked by a blue circle. Colours represent the time from the start of each flight: initial portion of each flight is in green, changing smoothly through yellow until the end of the flight is shown in red. Grey dashed lines are used to join radar observations made more than 30 s apart, when the bee’s location was uncertain. (ZIP) [file pone.0160333.s004.zip › S57 Fig.tif]

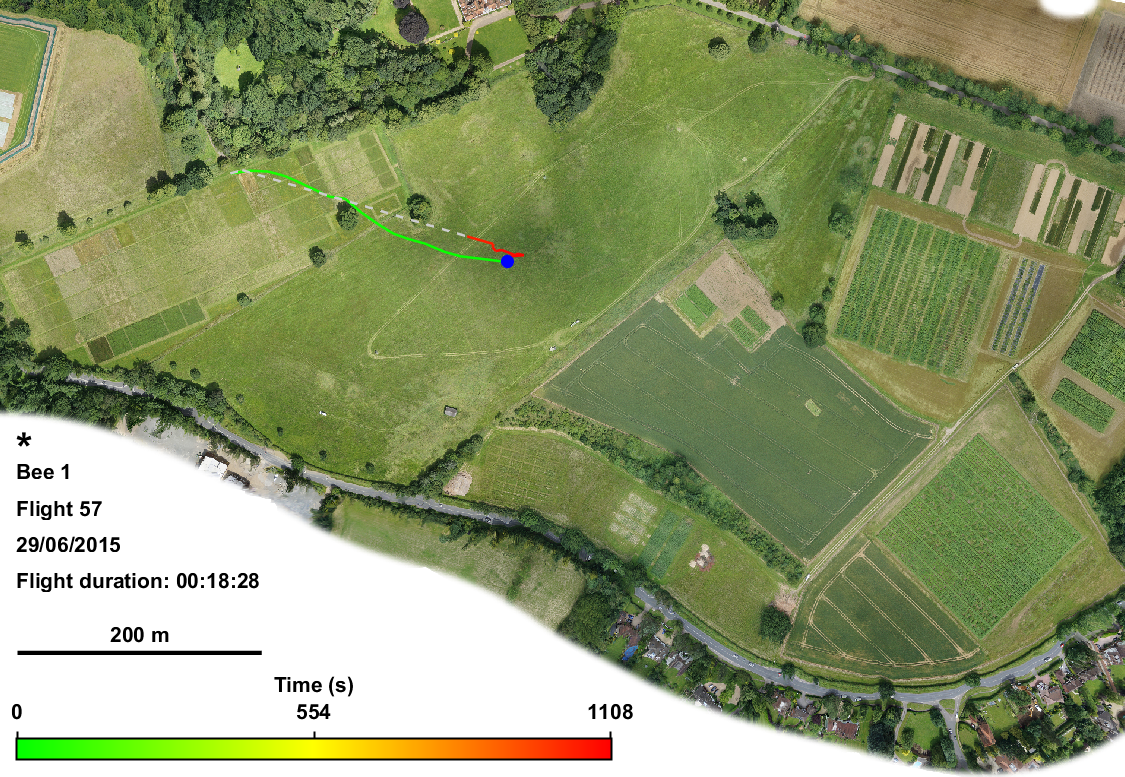

Supplement: S2 File — All flights made by Bee 1 during the period 28/06/2015–03/07/2015. Each figure represents a single flight. Bee ID, flight number, date of recording and the duration of each flight are shown in the bottom left corner of each figure. The position of the nest is marked by a blue circle. Colours represent the time from the start of each flight: initial portion of each flight is in green, changing smoothly through yellow until the end of the flight is shown in red. Grey dashed lines are used to join radar observations made more than 30 s apart, when the bee’s location was uncertain. (ZIP) [file pone.0160333.s004.zip › S58 Fig.tif]

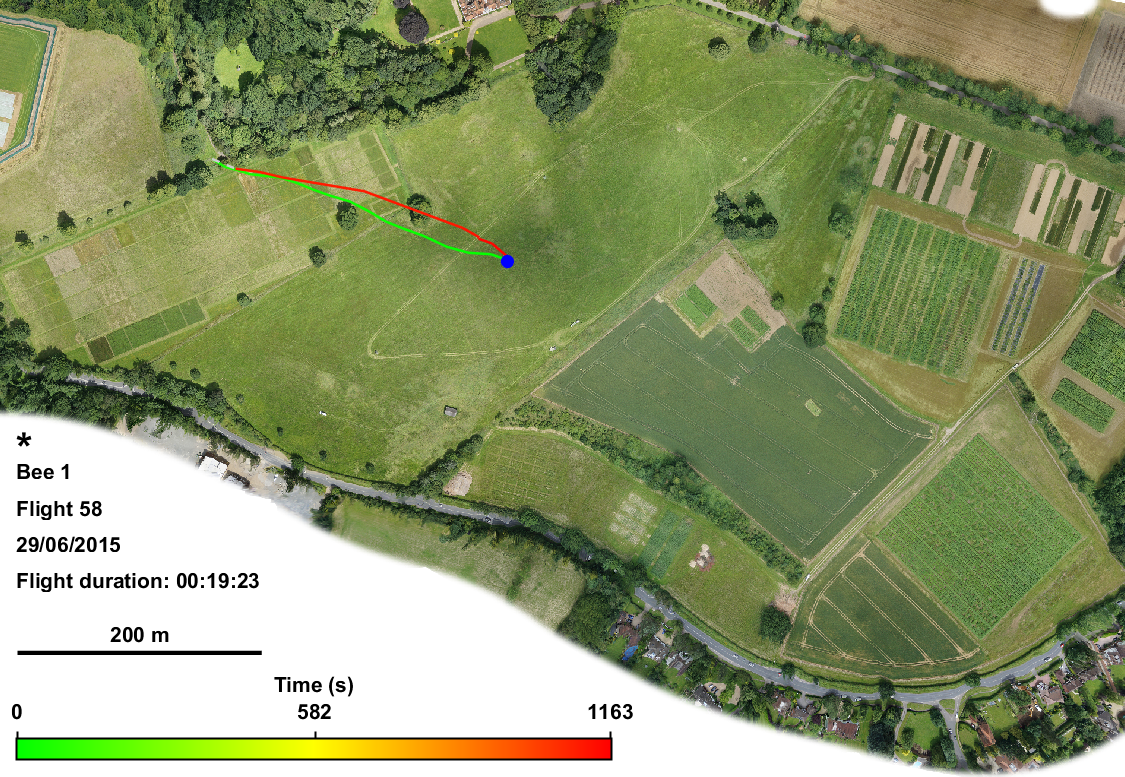

Supplement: S2 File — All flights made by Bee 1 during the period 28/06/2015–03/07/2015. Each figure represents a single flight. Bee ID, flight number, date of recording and the duration of each flight are shown in the bottom left corner of each figure. The position of the nest is marked by a blue circle. Colours represent the time from the start of each flight: initial portion of each flight is in green, changing smoothly through yellow until the end of the flight is shown in red. Grey dashed lines are used to join radar observations made more than 30 s apart, when the bee’s location was uncertain. (ZIP) [file pone.0160333.s004.zip › S59 Fig.tif]

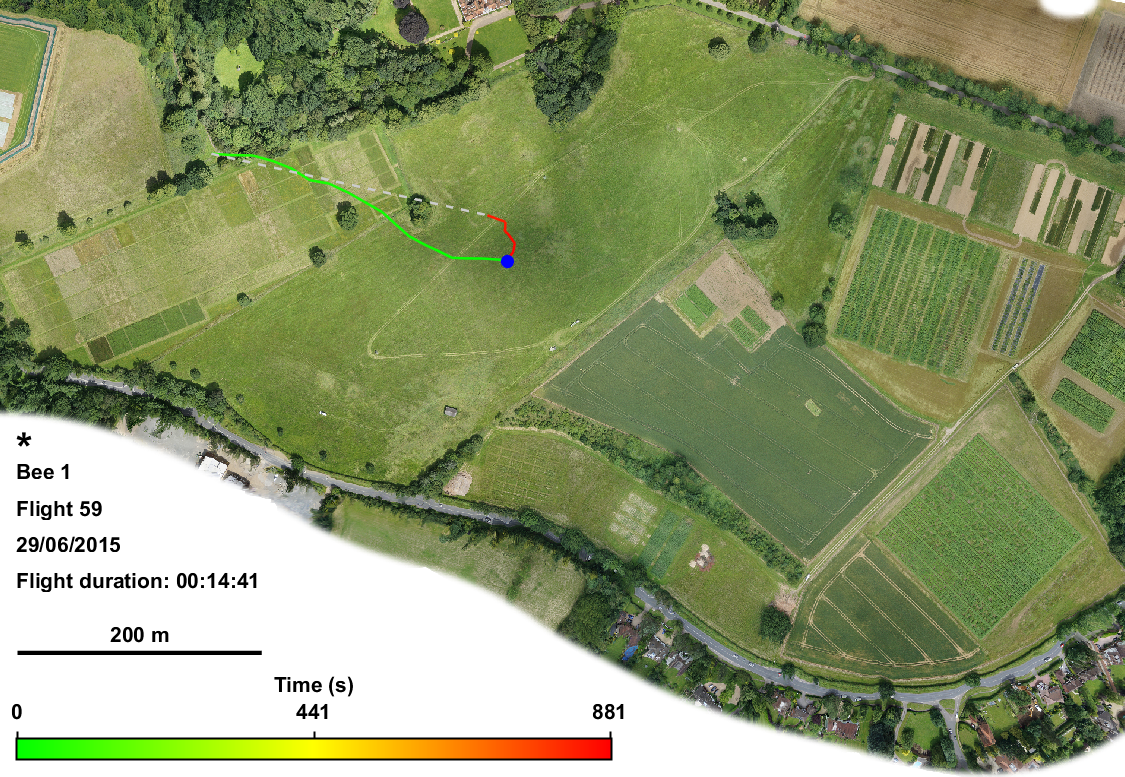

Supplement: S2 File — All flights made by Bee 1 during the period 28/06/2015–03/07/2015. Each figure represents a single flight. Bee ID, flight number, date of recording and the duration of each flight are shown in the bottom left corner of each figure. The position of the nest is marked by a blue circle. Colours represent the time from the start of each flight: initial portion of each flight is in green, changing smoothly through yellow until the end of the flight is shown in red. Grey dashed lines are used to join radar observations made more than 30 s apart, when the bee’s location was uncertain. (ZIP) [file pone.0160333.s004.zip › S60 Fig.tif]

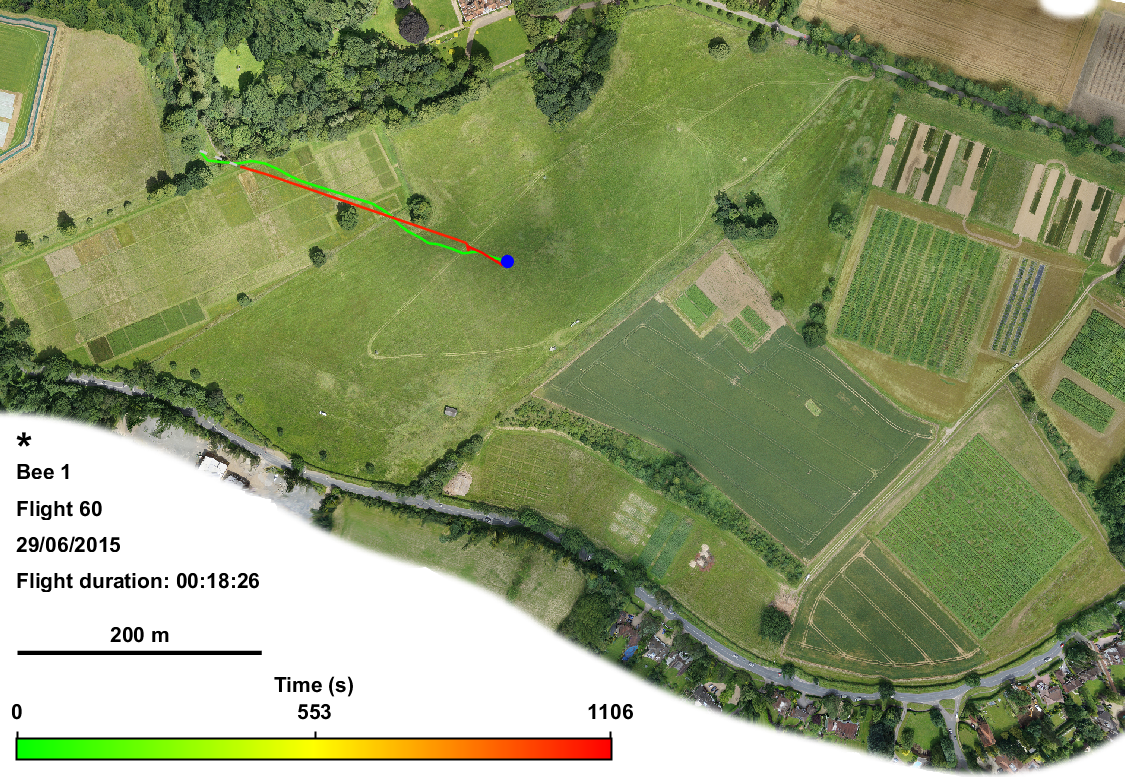

Supplement: S2 File — All flights made by Bee 1 during the period 28/06/2015–03/07/2015. Each figure represents a single flight. Bee ID, flight number, date of recording and the duration of each flight are shown in the bottom left corner of each figure. The position of the nest is marked by a blue circle. Colours represent the time from the start of each flight: initial portion of each flight is in green, changing smoothly through yellow until the end of the flight is shown in red. Grey dashed lines are used to join radar observations made more than 30 s apart, when the bee’s location was uncertain. (ZIP) [file pone.0160333.s004.zip › S61 Fig.tif]

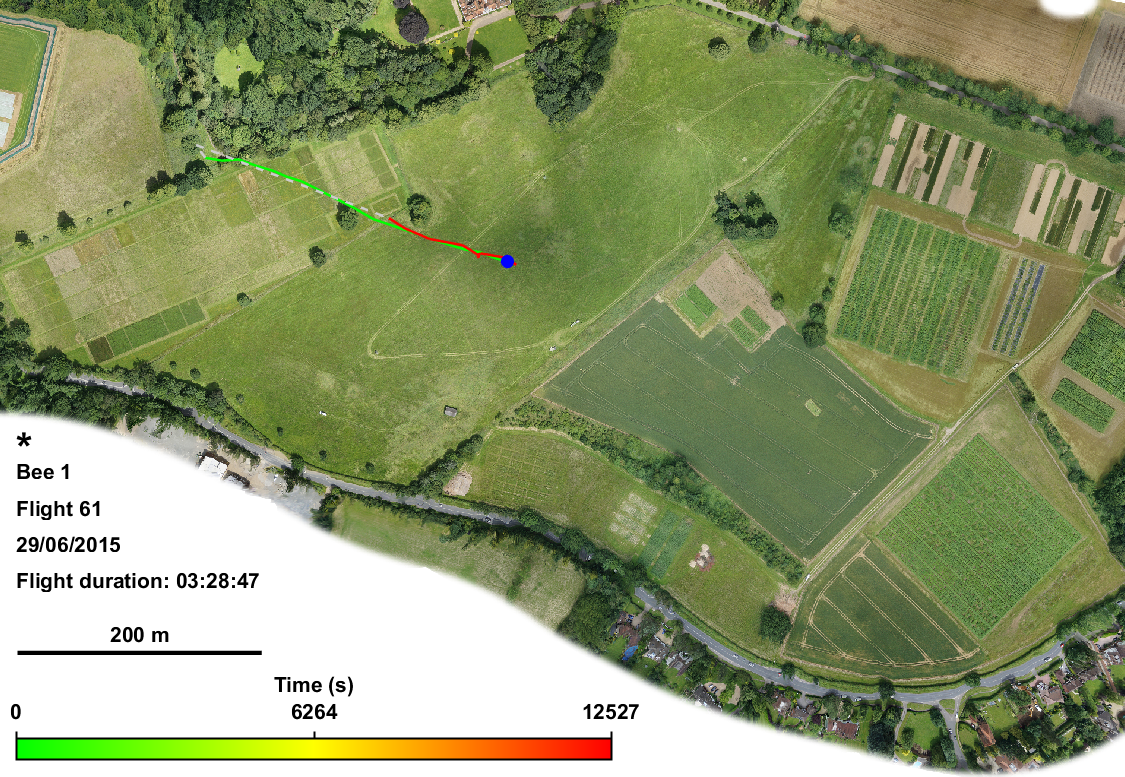

Supplement: S2 File — All flights made by Bee 1 during the period 28/06/2015–03/07/2015. Each figure represents a single flight. Bee ID, flight number, date of recording and the duration of each flight are shown in the bottom left corner of each figure. The position of the nest is marked by a blue circle. Colours represent the time from the start of each flight: initial portion of each flight is in green, changing smoothly through yellow until the end of the flight is shown in red. Grey dashed lines are used to join radar observations made more than 30 s apart, when the bee’s location was uncertain. (ZIP) [file pone.0160333.s004.zip › S62 Fig.tif]

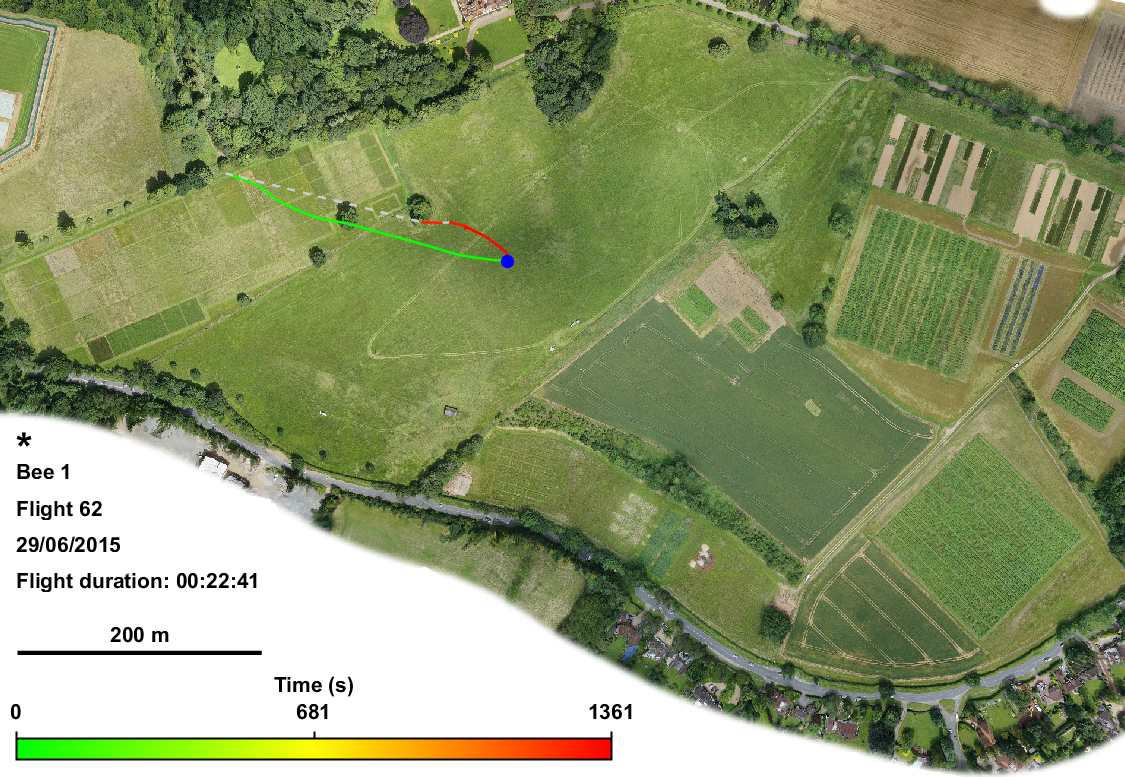

Supplement: S2 File — All flights made by Bee 1 during the period 28/06/2015–03/07/2015. Each figure represents a single flight. Bee ID, flight number, date of recording and the duration of each flight are shown in the bottom left corner of each figure. The position of the nest is marked by a blue circle. Colours represent the time from the start of each flight: initial portion of each flight is in green, changing smoothly through yellow until the end of the flight is shown in red. Grey dashed lines are used to join radar observations made more than 30 s apart, when the bee’s location was uncertain. (ZIP) [file pone.0160333.s004.zip › S63 Fig.tif]

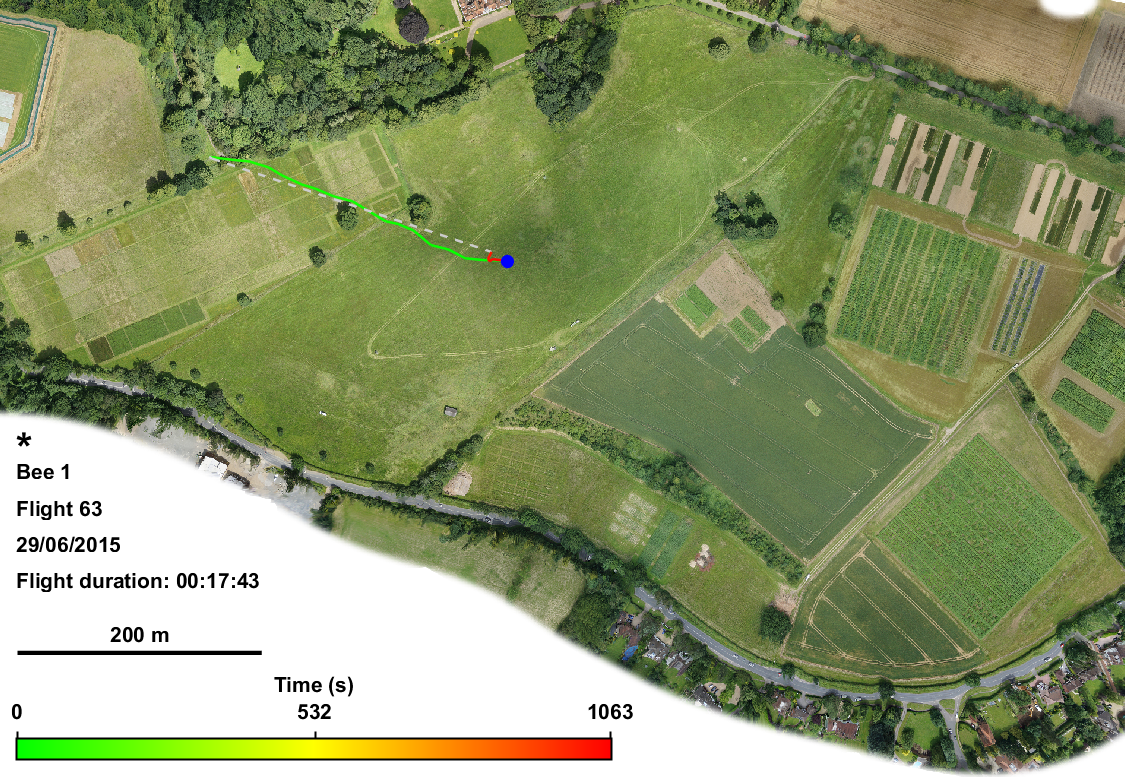

Supplement: S2 File — All flights made by Bee 1 during the period 28/06/2015–03/07/2015. Each figure represents a single flight. Bee ID, flight number, date of recording and the duration of each flight are shown in the bottom left corner of each figure. The position of the nest is marked by a blue circle. Colours represent the time from the start of each flight: initial portion of each flight is in green, changing smoothly through yellow until the end of the flight is shown in red. Grey dashed lines are used to join radar observations made more than 30 s apart, when the bee’s location was uncertain. (ZIP) [file pone.0160333.s004.zip › S64 Fig.tif]

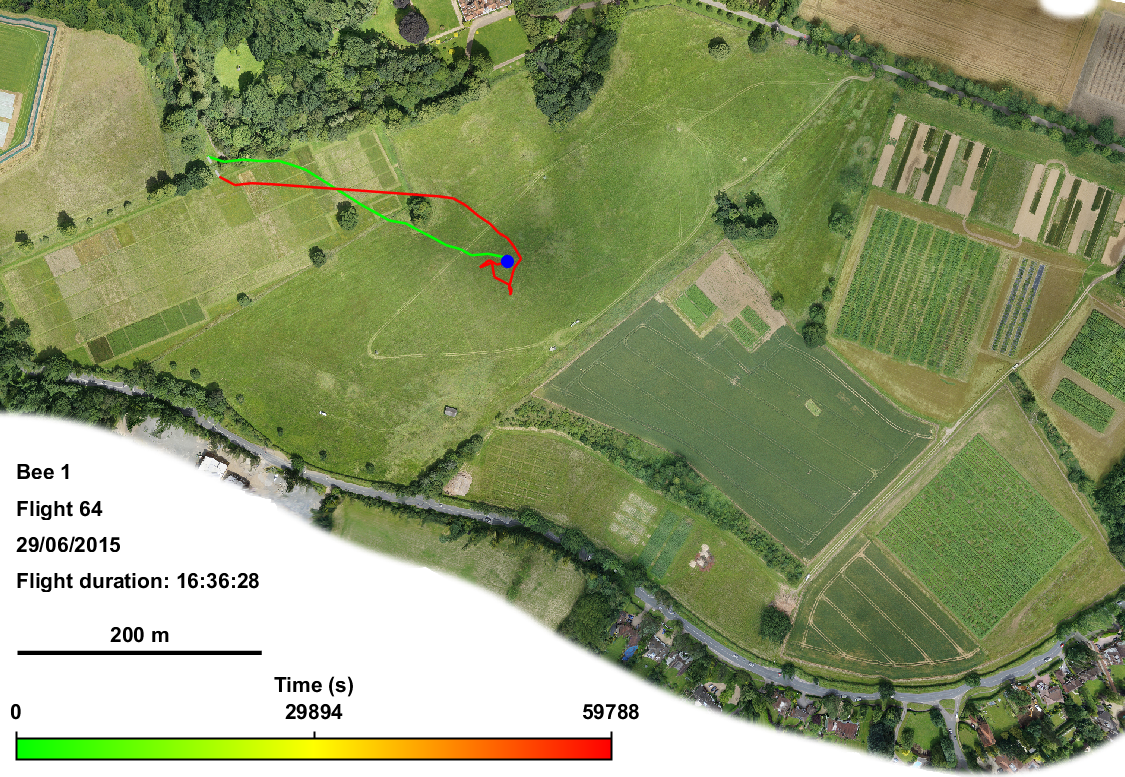

Supplement: S2 File — All flights made by Bee 1 during the period 28/06/2015–03/07/2015. Each figure represents a single flight. Bee ID, flight number, date of recording and the duration of each flight are shown in the bottom left corner of each figure. The position of the nest is marked by a blue circle. Colours represent the time from the start of each flight: initial portion of each flight is in green, changing smoothly through yellow until the end of the flight is shown in red. Grey dashed lines are used to join radar observations made more than 30 s apart, when the bee’s location was uncertain. (ZIP) [file pone.0160333.s004.zip › S65 Fig.tif]

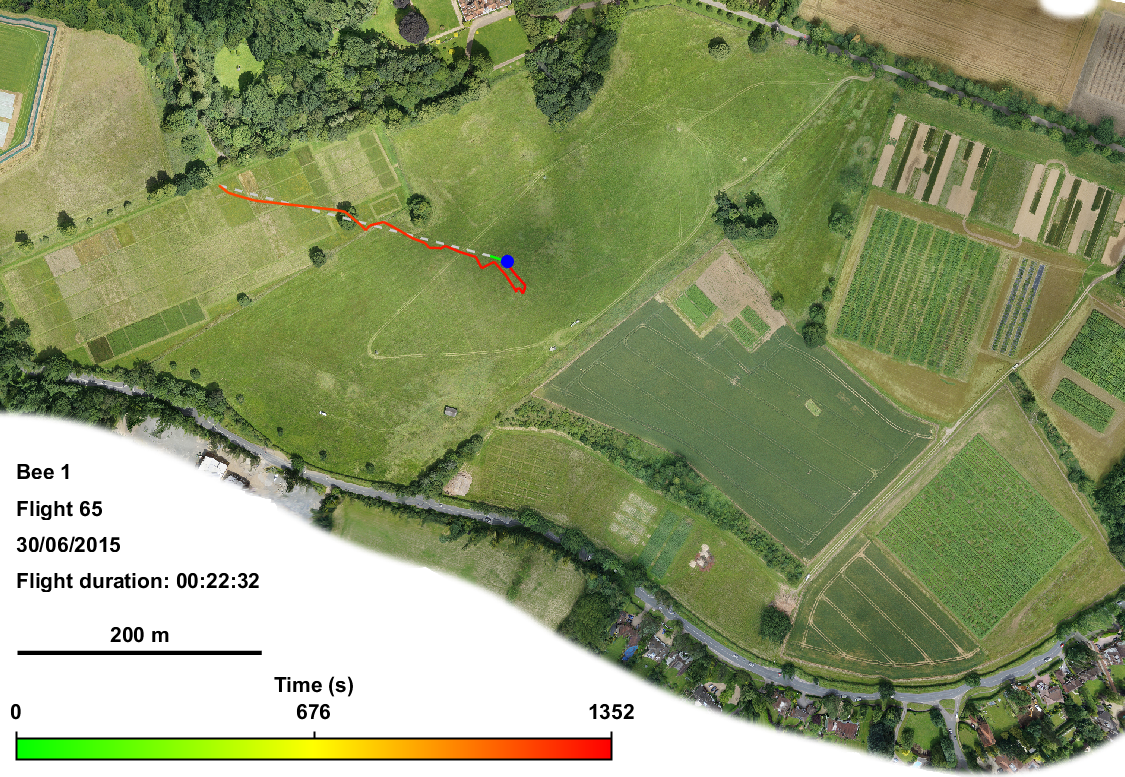

Supplement: S2 File — All flights made by Bee 1 during the period 28/06/2015–03/07/2015. Each figure represents a single flight. Bee ID, flight number, date of recording and the duration of each flight are shown in the bottom left corner of each figure. The position of the nest is marked by a blue circle. Colours represent the time from the start of each flight: initial portion of each flight is in green, changing smoothly through yellow until the end of the flight is shown in red. Grey dashed lines are used to join radar observations made more than 30 s apart, when the bee’s location was uncertain. (ZIP) [file pone.0160333.s004.zip › S66 Fig.tif]

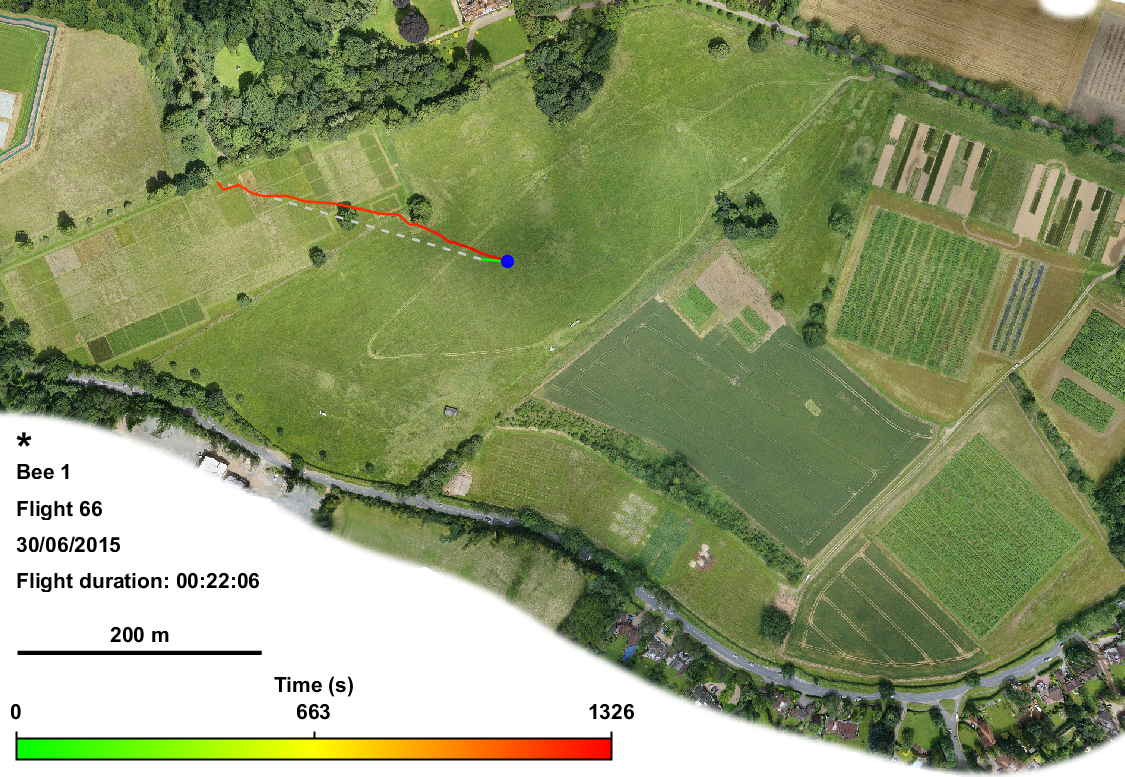

Supplement: S2 File — All flights made by Bee 1 during the period 28/06/2015–03/07/2015. Each figure represents a single flight. Bee ID, flight number, date of recording and the duration of each flight are shown in the bottom left corner of each figure. The position of the nest is marked by a blue circle. Colours represent the time from the start of each flight: initial portion of each flight is in green, changing smoothly through yellow until the end of the flight is shown in red. Grey dashed lines are used to join radar observations made more than 30 s apart, when the bee’s location was uncertain. (ZIP) [file pone.0160333.s004.zip › S67 Fig.tif]

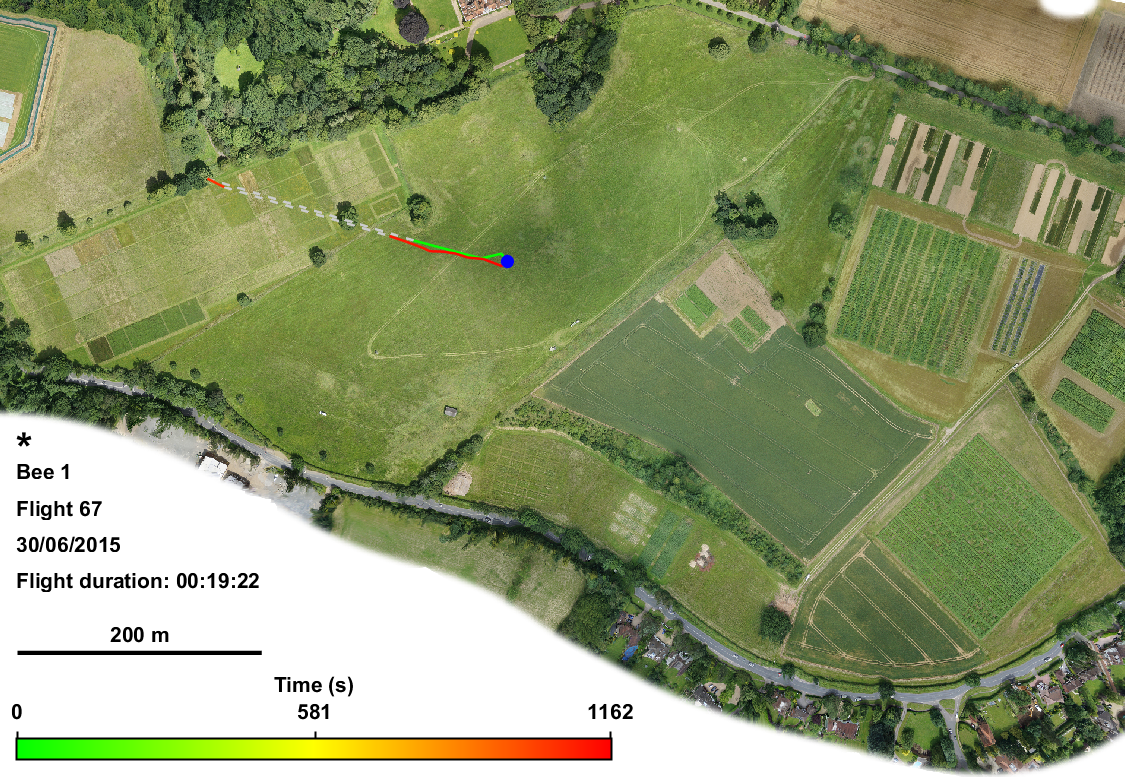

Supplement: S2 File — All flights made by Bee 1 during the period 28/06/2015–03/07/2015. Each figure represents a single flight. Bee ID, flight number, date of recording and the duration of each flight are shown in the bottom left corner of each figure. The position of the nest is marked by a blue circle. Colours represent the time from the start of each flight: initial portion of each flight is in green, changing smoothly through yellow until the end of the flight is shown in red. Grey dashed lines are used to join radar observations made more than 30 s apart, when the bee’s location was uncertain. (ZIP) [file pone.0160333.s004.zip › S68 Fig.tif]

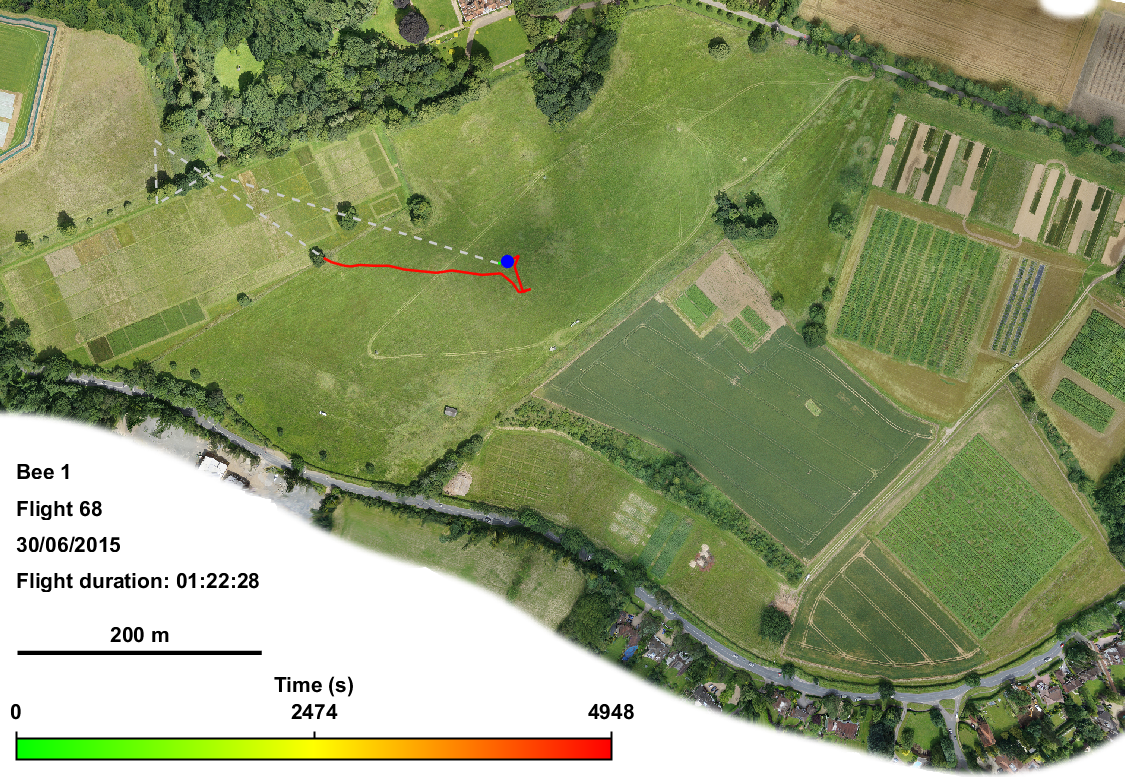

Supplement: S2 File — All flights made by Bee 1 during the period 28/06/2015–03/07/2015. Each figure represents a single flight. Bee ID, flight number, date of recording and the duration of each flight are shown in the bottom left corner of each figure. The position of the nest is marked by a blue circle. Colours represent the time from the start of each flight: initial portion of each flight is in green, changing smoothly through yellow until the end of the flight is shown in red. Grey dashed lines are used to join radar observations made more than 30 s apart, when the bee’s location was uncertain. (ZIP) [file pone.0160333.s004.zip › S69 Fig.tif]

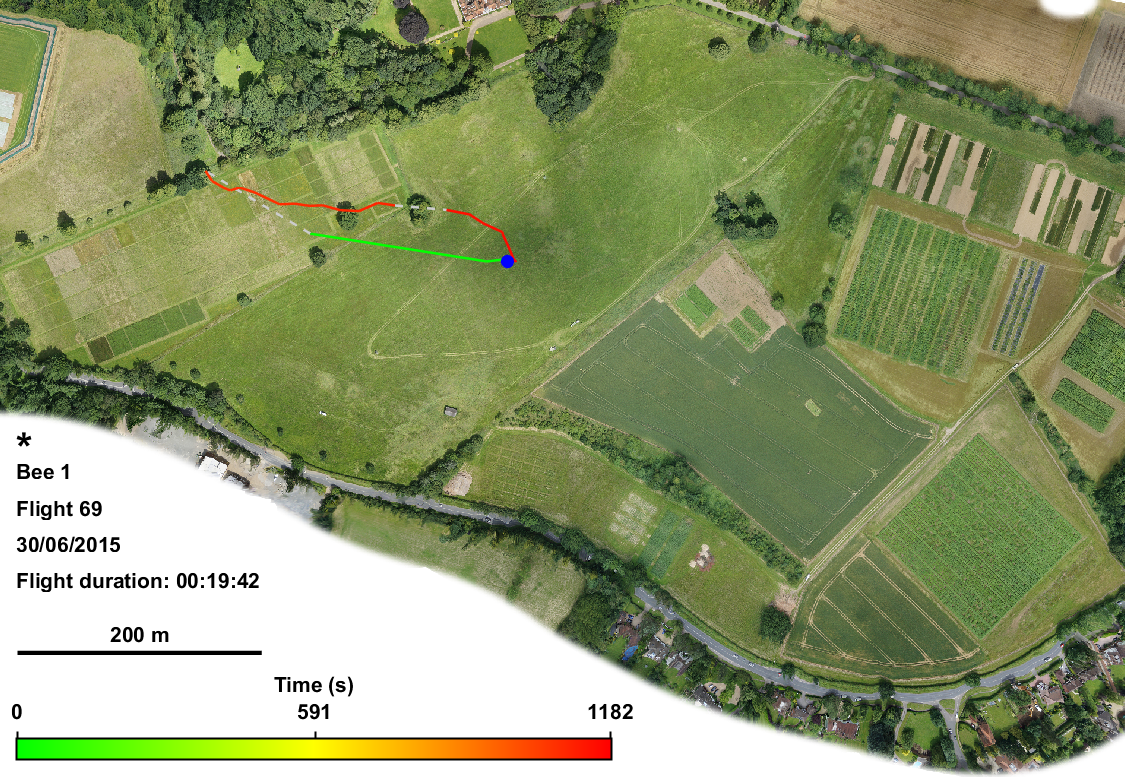

Supplement: S2 File — All flights made by Bee 1 during the period 28/06/2015–03/07/2015. Each figure represents a single flight. Bee ID, flight number, date of recording and the duration of each flight are shown in the bottom left corner of each figure. The position of the nest is marked by a blue circle. Colours represent the time from the start of each flight: initial portion of each flight is in green, changing smoothly through yellow until the end of the flight is shown in red. Grey dashed lines are used to join radar observations made more than 30 s apart, when the bee’s location was uncertain. (ZIP) [file pone.0160333.s004.zip › S70 Fig.tif]

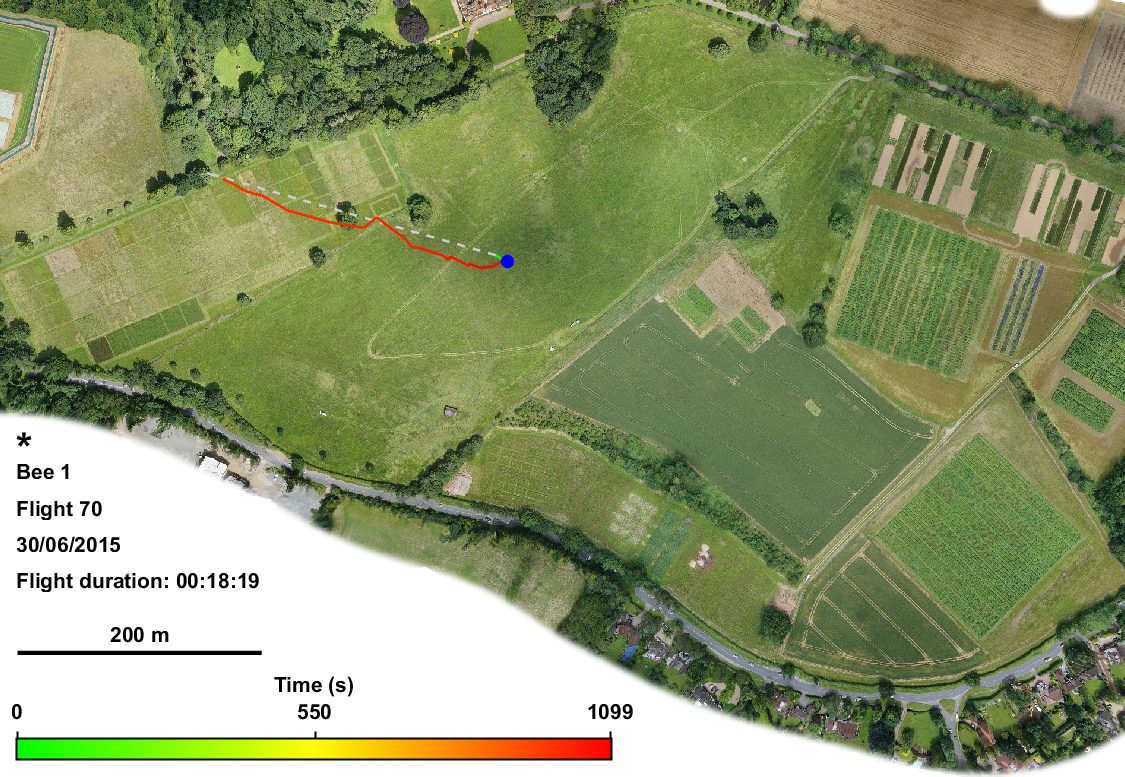

Supplement: S2 File — All flights made by Bee 1 during the period 28/06/2015–03/07/2015. Each figure represents a single flight. Bee ID, flight number, date of recording and the duration of each flight are shown in the bottom left corner of each figure. The position of the nest is marked by a blue circle. Colours represent the time from the start of each flight: initial portion of each flight is in green, changing smoothly through yellow until the end of the flight is shown in red. Grey dashed lines are used to join radar observations made more than 30 s apart, when the bee’s location was uncertain. (ZIP) [file pone.0160333.s004.zip › S71 Fig.tif]

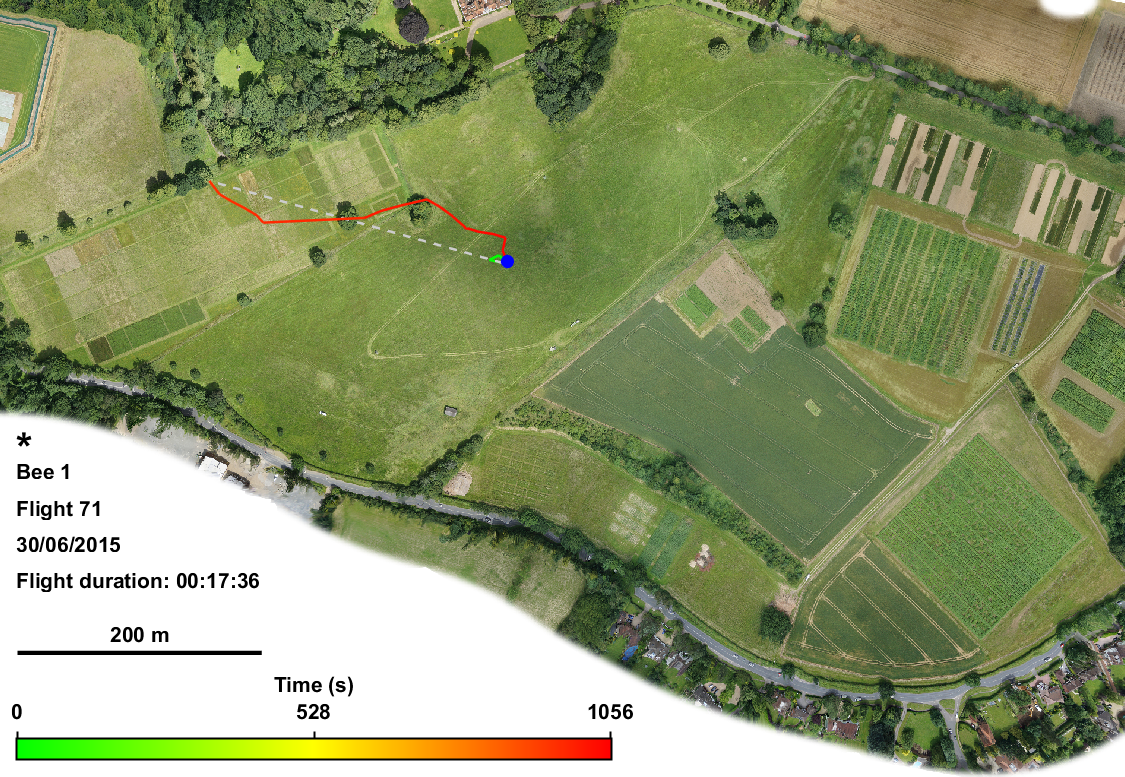

Supplement: S2 File — All flights made by Bee 1 during the period 28/06/2015–03/07/2015. Each figure represents a single flight. Bee ID, flight number, date of recording and the duration of each flight are shown in the bottom left corner of each figure. The position of the nest is marked by a blue circle. Colours represent the time from the start of each flight: initial portion of each flight is in green, changing smoothly through yellow until the end of the flight is shown in red. Grey dashed lines are used to join radar observations made more than 30 s apart, when the bee’s location was uncertain. (ZIP) [file pone.0160333.s004.zip › S72 Fig.tif]

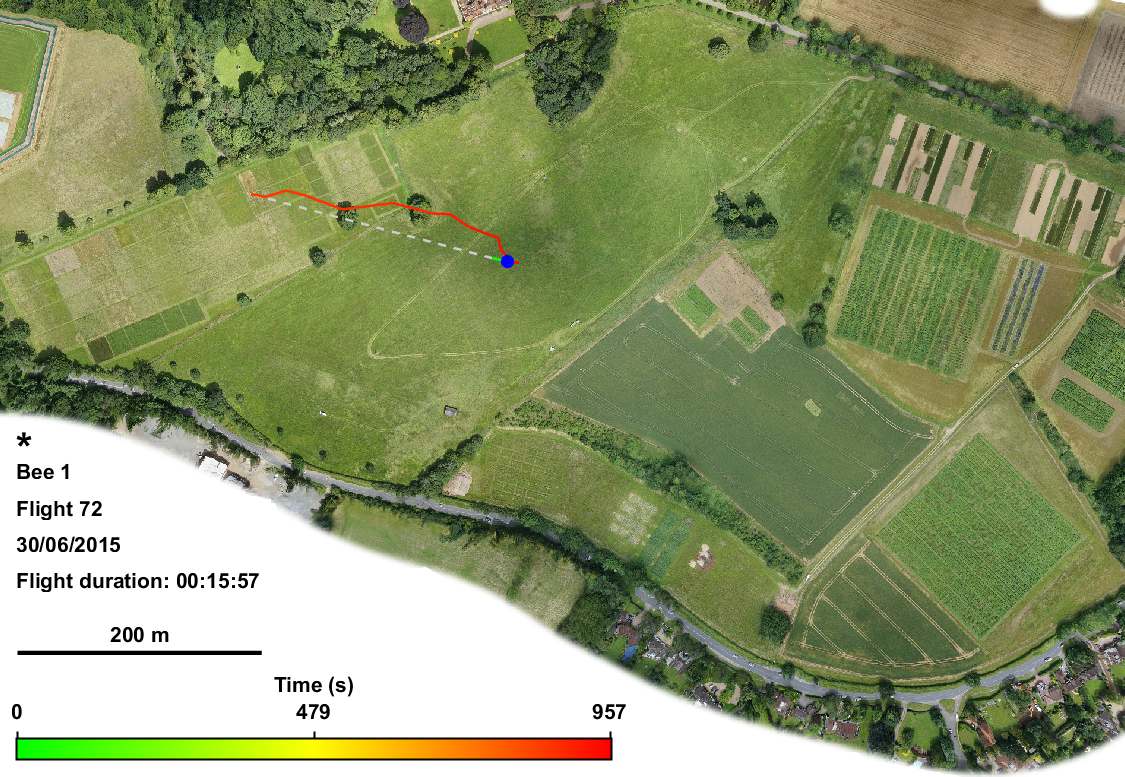

Supplement: S2 File — All flights made by Bee 1 during the period 28/06/2015–03/07/2015. Each figure represents a single flight. Bee ID, flight number, date of recording and the duration of each flight are shown in the bottom left corner of each figure. The position of the nest is marked by a blue circle. Colours represent the time from the start of each flight: initial portion of each flight is in green, changing smoothly through yellow until the end of the flight is shown in red. Grey dashed lines are used to join radar observations made more than 30 s apart, when the bee’s location was uncertain. (ZIP) [file pone.0160333.s004.zip › S73 Fig.tif]

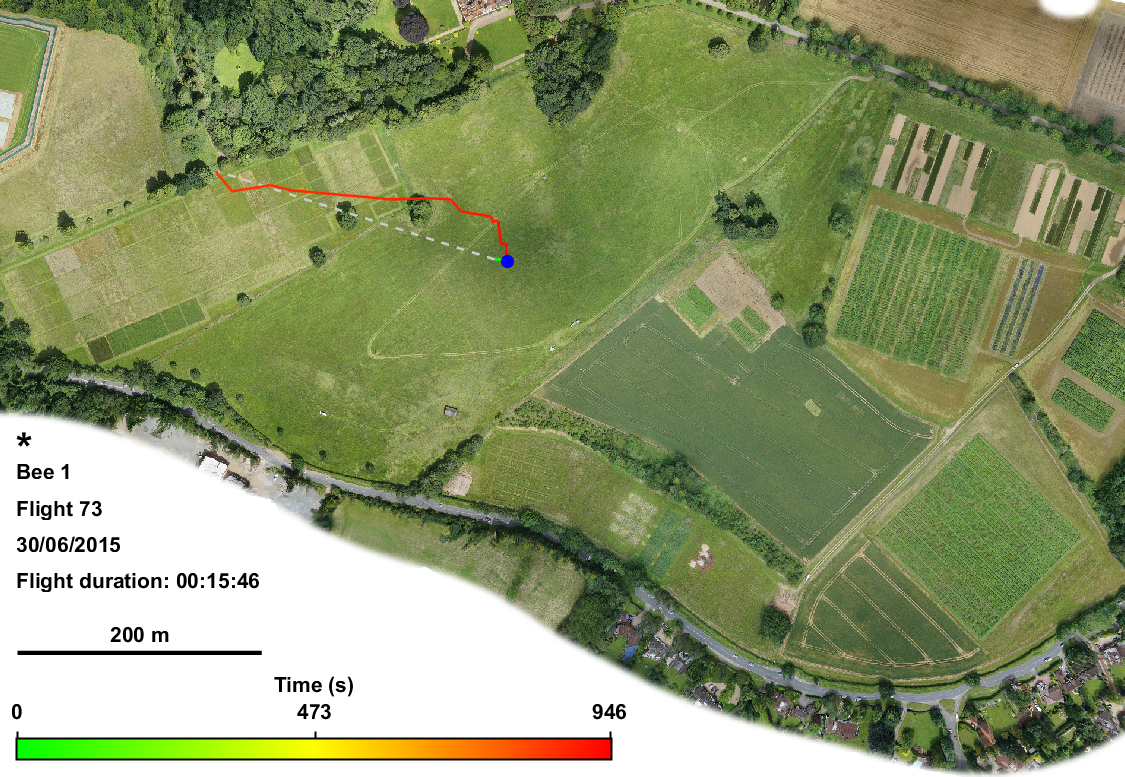

Supplement: S2 File — All flights made by Bee 1 during the period 28/06/2015–03/07/2015. Each figure represents a single flight. Bee ID, flight number, date of recording and the duration of each flight are shown in the bottom left corner of each figure. The position of the nest is marked by a blue circle. Colours represent the time from the start of each flight: initial portion of each flight is in green, changing smoothly through yellow until the end of the flight is shown in red. Grey dashed lines are used to join radar observations made more than 30 s apart, when the bee’s location was uncertain. (ZIP) [file pone.0160333.s004.zip › S74 Fig.tif]

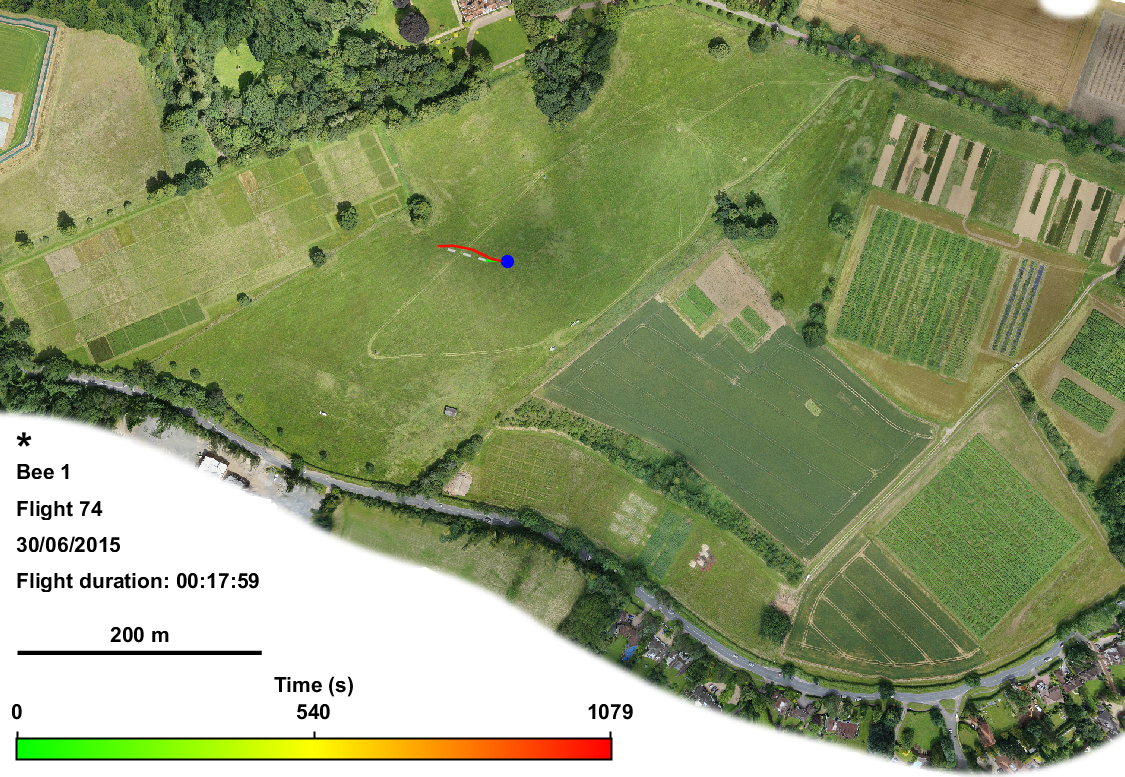

Supplement: S2 File — All flights made by Bee 1 during the period 28/06/2015–03/07/2015. Each figure represents a single flight. Bee ID, flight number, date of recording and the duration of each flight are shown in the bottom left corner of each figure. The position of the nest is marked by a blue circle. Colours represent the time from the start of each flight: initial portion of each flight is in green, changing smoothly through yellow until the end of the flight is shown in red. Grey dashed lines are used to join radar observations made more than 30 s apart, when the bee’s location was uncertain. (ZIP) [file pone.0160333.s004.zip › S75 Fig.tif]

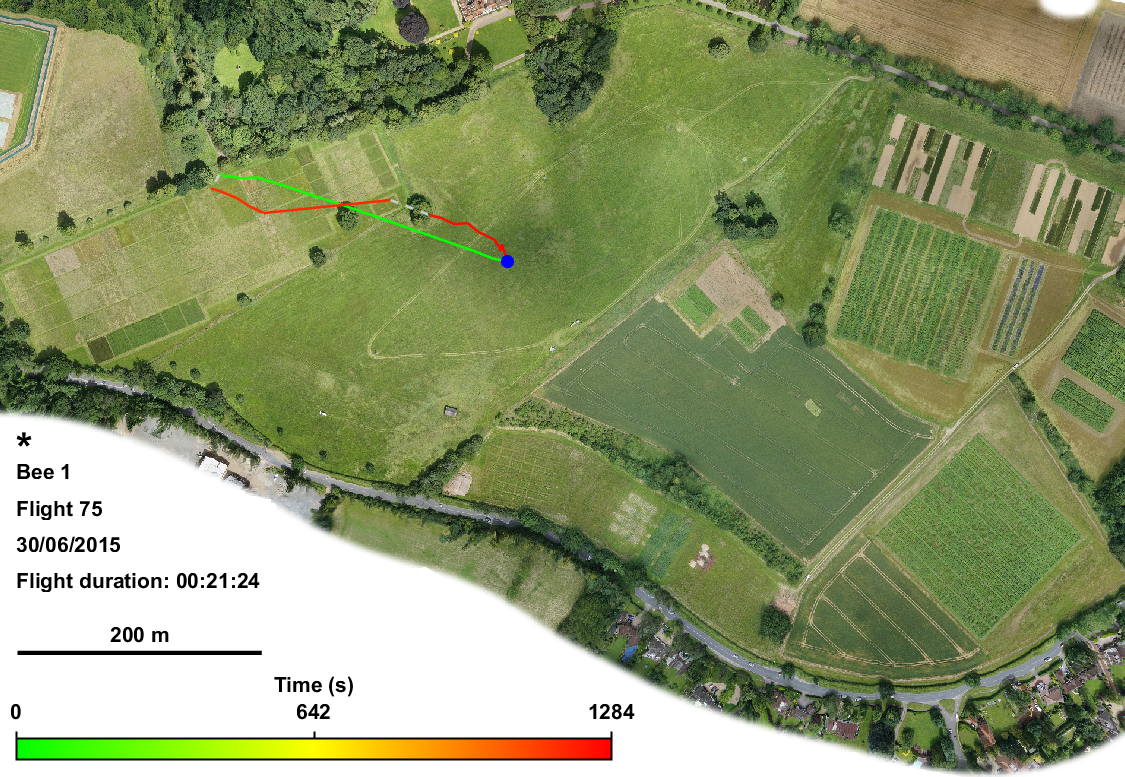

Supplement: S2 File — All flights made by Bee 1 during the period 28/06/2015–03/07/2015. Each figure represents a single flight. Bee ID, flight number, date of recording and the duration of each flight are shown in the bottom left corner of each figure. The position of the nest is marked by a blue circle. Colours represent the time from the start of each flight: initial portion of each flight is in green, changing smoothly through yellow until the end of the flight is shown in red. Grey dashed lines are used to join radar observations made more than 30 s apart, when the bee’s location was uncertain. (ZIP) [file pone.0160333.s004.zip › S76 Fig.tif]

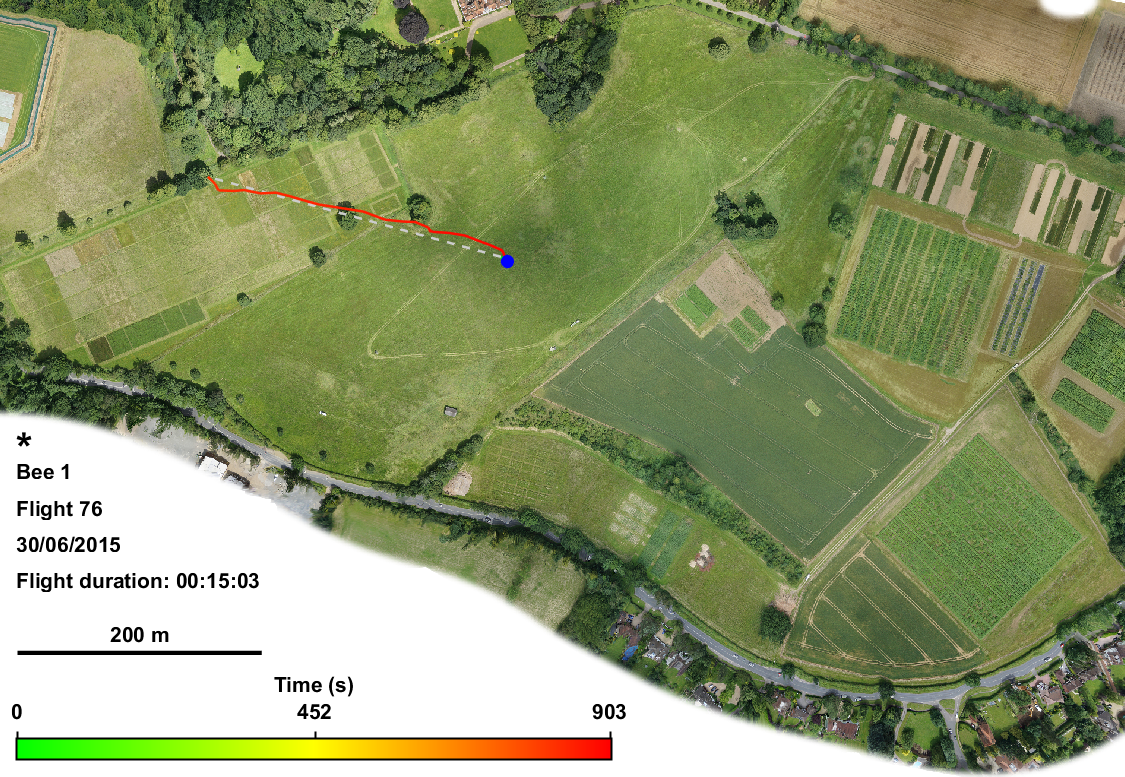

Supplement: S2 File — All flights made by Bee 1 during the period 28/06/2015–03/07/2015. Each figure represents a single flight. Bee ID, flight number, date of recording and the duration of each flight are shown in the bottom left corner of each figure. The position of the nest is marked by a blue circle. Colours represent the time from the start of each flight: initial portion of each flight is in green, changing smoothly through yellow until the end of the flight is shown in red. Grey dashed lines are used to join radar observations made more than 30 s apart, when the bee’s location was uncertain. (ZIP) [file pone.0160333.s004.zip › S77 Fig.tif]

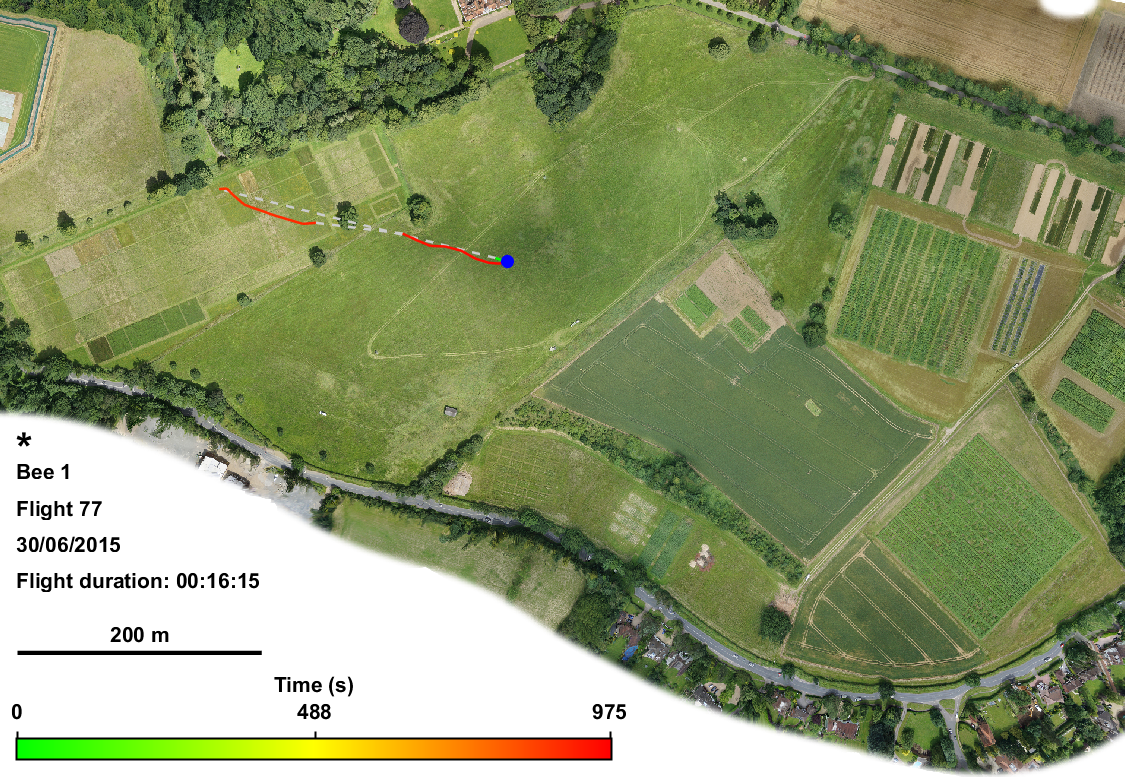

Supplement: S2 File — All flights made by Bee 1 during the period 28/06/2015–03/07/2015. Each figure represents a single flight. Bee ID, flight number, date of recording and the duration of each flight are shown in the bottom left corner of each figure. The position of the nest is marked by a blue circle. Colours represent the time from the start of each flight: initial portion of each flight is in green, changing smoothly through yellow until the end of the flight is shown in red. Grey dashed lines are used to join radar observations made more than 30 s apart, when the bee’s location was uncertain. (ZIP) [file pone.0160333.s004.zip › S78 Fig.tif]

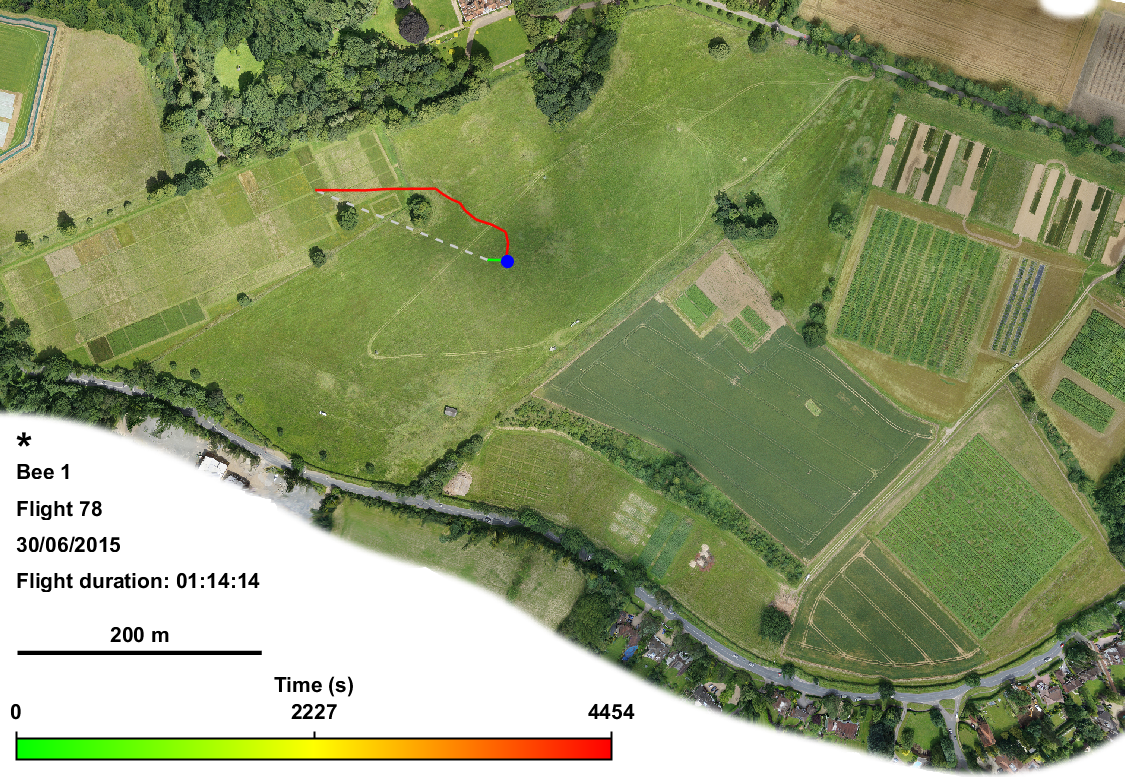

Supplement: S2 File — All flights made by Bee 1 during the period 28/06/2015–03/07/2015. Each figure represents a single flight. Bee ID, flight number, date of recording and the duration of each flight are shown in the bottom left corner of each figure. The position of the nest is marked by a blue circle. Colours represent the time from the start of each flight: initial portion of each flight is in green, changing smoothly through yellow until the end of the flight is shown in red. Grey dashed lines are used to join radar observations made more than 30 s apart, when the bee’s location was uncertain. (ZIP) [file pone.0160333.s004.zip › S79 Fig.tif]

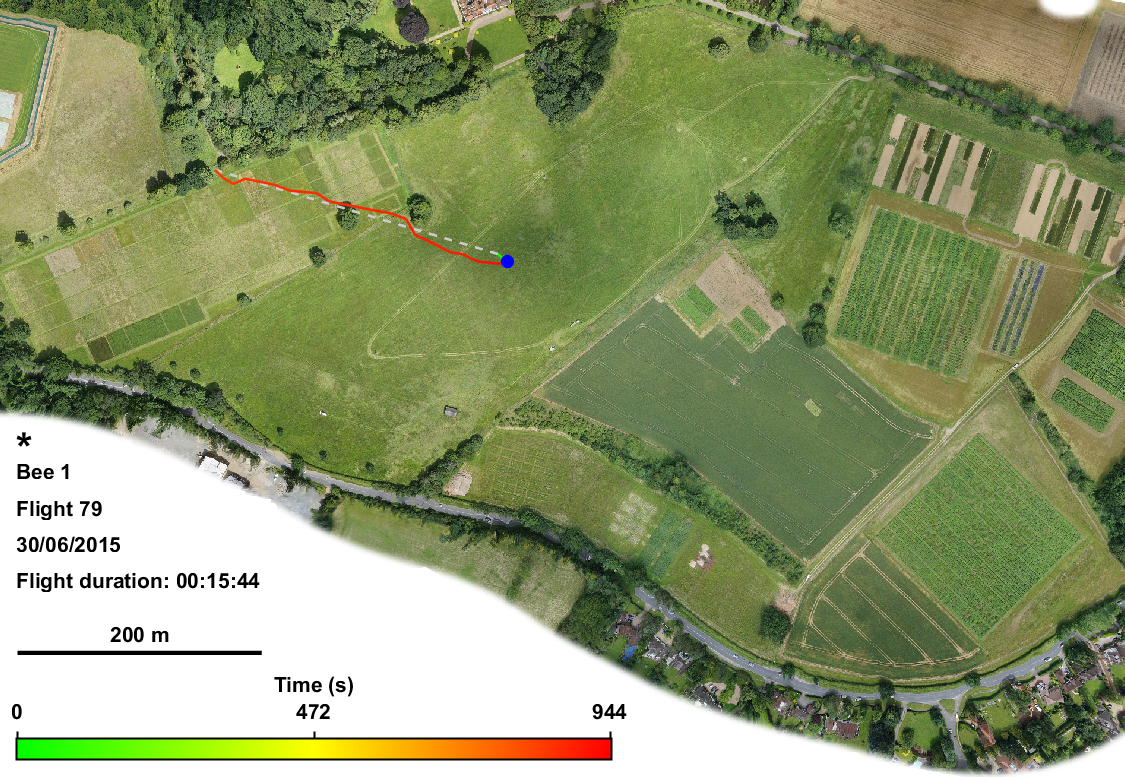

Supplement: S2 File — All flights made by Bee 1 during the period 28/06/2015–03/07/2015. Each figure represents a single flight. Bee ID, flight number, date of recording and the duration of each flight are shown in the bottom left corner of each figure. The position of the nest is marked by a blue circle. Colours represent the time from the start of each flight: initial portion of each flight is in green, changing smoothly through yellow until the end of the flight is shown in red. Grey dashed lines are used to join radar observations made more than 30 s apart, when the bee’s location was uncertain. (ZIP) [file pone.0160333.s004.zip › S80 Fig.tif]

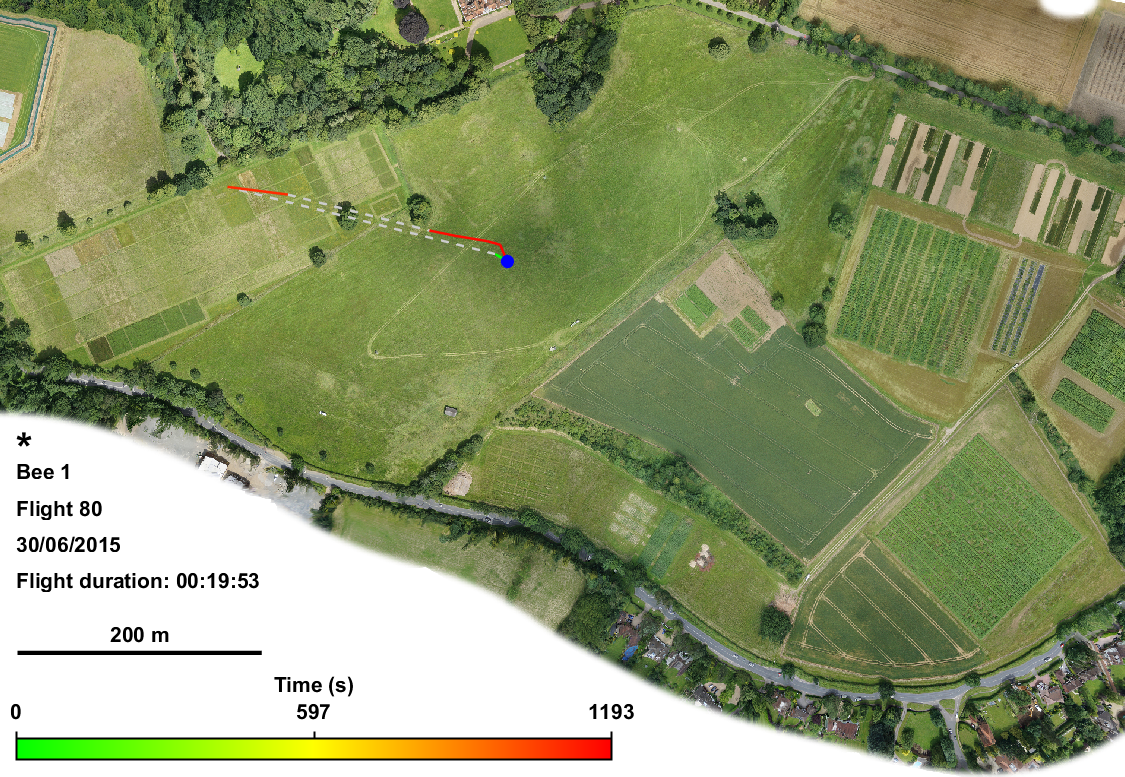

Supplement: S2 File — All flights made by Bee 1 during the period 28/06/2015–03/07/2015. Each figure represents a single flight. Bee ID, flight number, date of recording and the duration of each flight are shown in the bottom left corner of each figure. The position of the nest is marked by a blue circle. Colours represent the time from the start of each flight: initial portion of each flight is in green, changing smoothly through yellow until the end of the flight is shown in red. Grey dashed lines are used to join radar observations made more than 30 s apart, when the bee’s location was uncertain. (ZIP) [file pone.0160333.s004.zip › S81 Fig.tif]

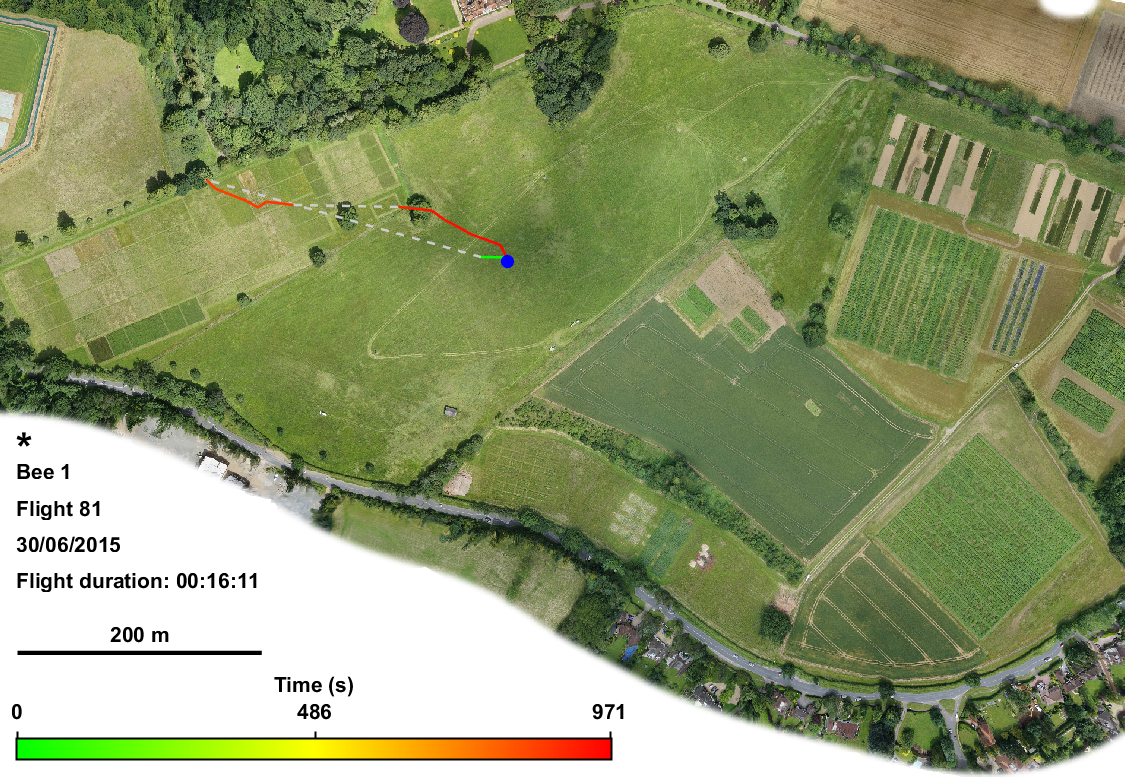

Supplement: S2 File — All flights made by Bee 1 during the period 28/06/2015–03/07/2015. Each figure represents a single flight. Bee ID, flight number, date of recording and the duration of each flight are shown in the bottom left corner of each figure. The position of the nest is marked by a blue circle. Colours represent the time from the start of each flight: initial portion of each flight is in green, changing smoothly through yellow until the end of the flight is shown in red. Grey dashed lines are used to join radar observations made more than 30 s apart, when the bee’s location was uncertain. (ZIP) [file pone.0160333.s004.zip › S82 Fig.tif]

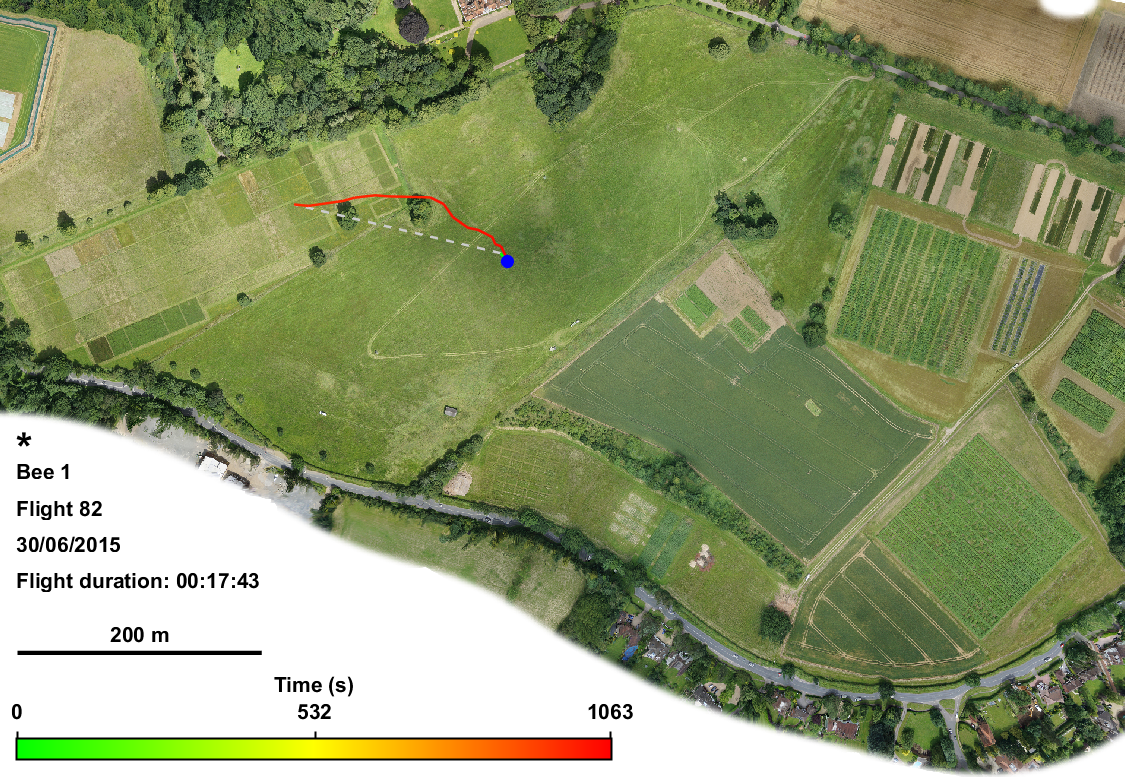

Supplement: S2 File — All flights made by Bee 1 during the period 28/06/2015–03/07/2015. Each figure represents a single flight. Bee ID, flight number, date of recording and the duration of each flight are shown in the bottom left corner of each figure. The position of the nest is marked by a blue circle. Colours represent the time from the start of each flight: initial portion of each flight is in green, changing smoothly through yellow until the end of the flight is shown in red. Grey dashed lines are used to join radar observations made more than 30 s apart, when the bee’s location was uncertain. (ZIP) [file pone.0160333.s004.zip › S83 Fig.tif]

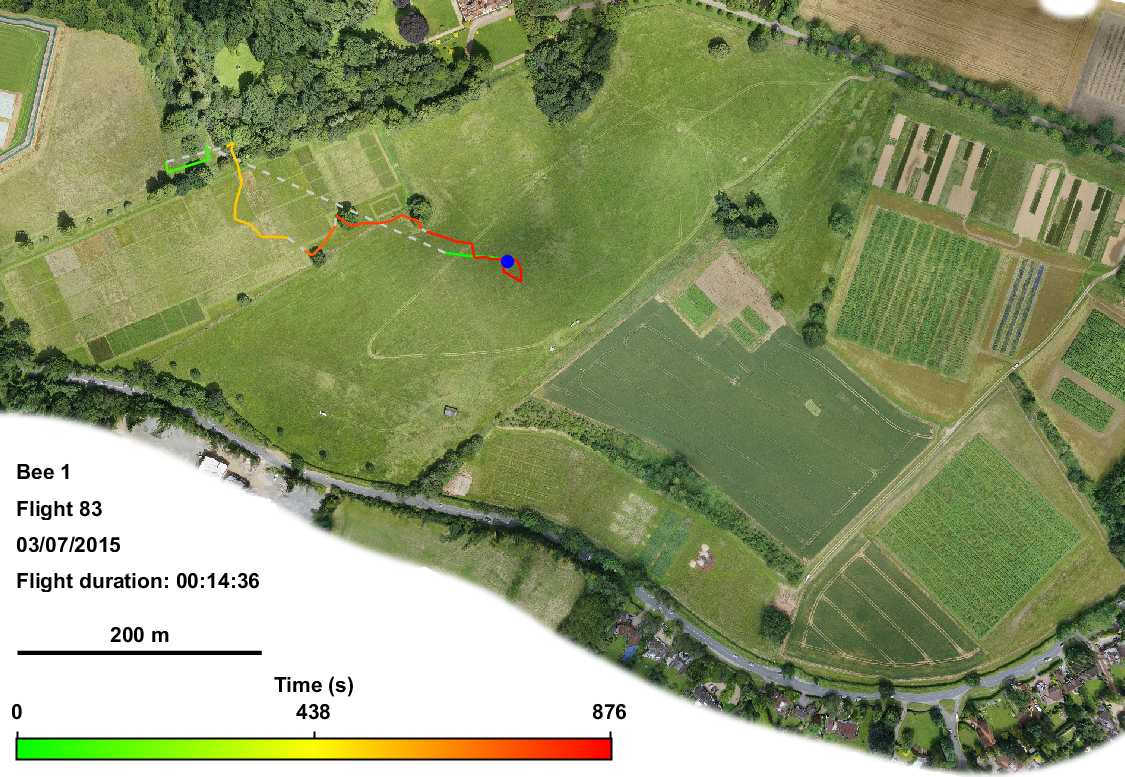

Supplement: S2 File — All flights made by Bee 1 during the period 28/06/2015–03/07/2015. Each figure represents a single flight. Bee ID, flight number, date of recording and the duration of each flight are shown in the bottom left corner of each figure. The position of the nest is marked by a blue circle. Colours represent the time from the start of each flight: initial portion of each flight is in green, changing smoothly through yellow until the end of the flight is shown in red. Grey dashed lines are used to join radar observations made more than 30 s apart, when the bee’s location was uncertain. (ZIP) [file pone.0160333.s004.zip › S84 Fig.tif]

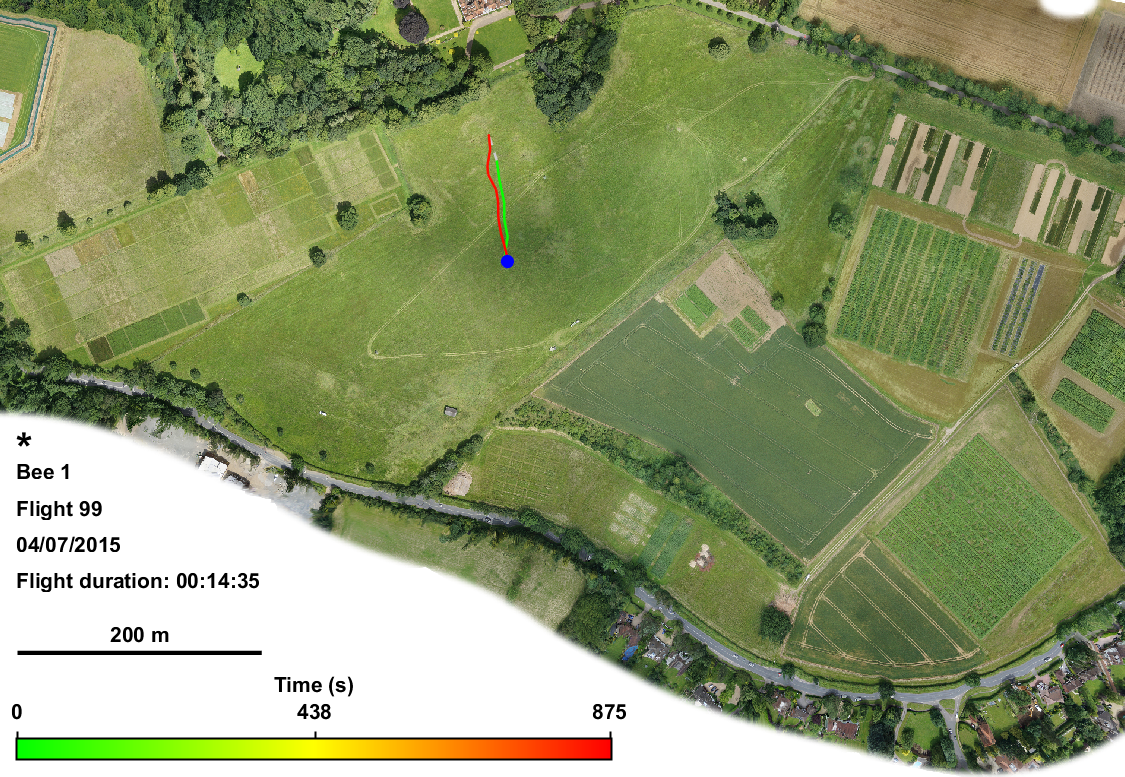

Supplement: S3 File — All flights made by Bee 1 on 04/07/2015. Each figure represents a single flight. Bee ID, flight number, date of recording and the duration of each flight are shown in the bottom left corner of each figure. The position of the nest is marked by a blue circle. Colours represent the time from the start of each flight: initial portion of each flight is in green, changing smoothly through yellow until the end of the flight is shown in red. Grey dashed lines are used to join radar observations made more than 30 s apart, when the bee’s location was uncertain. (ZIP) [file pone.0160333.s005.zip › S100 Fig.tif]

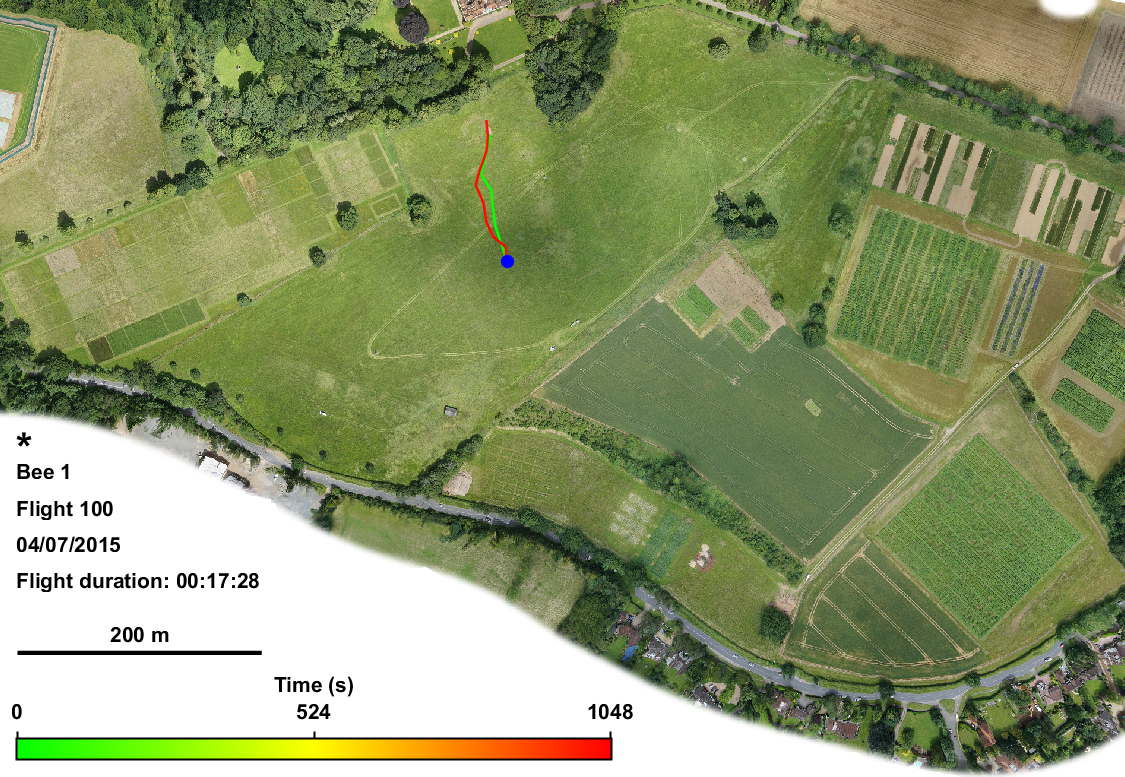

Supplement: S3 File — All flights made by Bee 1 on 04/07/2015. Each figure represents a single flight. Bee ID, flight number, date of recording and the duration of each flight are shown in the bottom left corner of each figure. The position of the nest is marked by a blue circle. Colours represent the time from the start of each flight: initial portion of each flight is in green, changing smoothly through yellow until the end of the flight is shown in red. Grey dashed lines are used to join radar observations made more than 30 s apart, when the bee’s location was uncertain. (ZIP) [file pone.0160333.s005.zip › S101 Fig.tif]

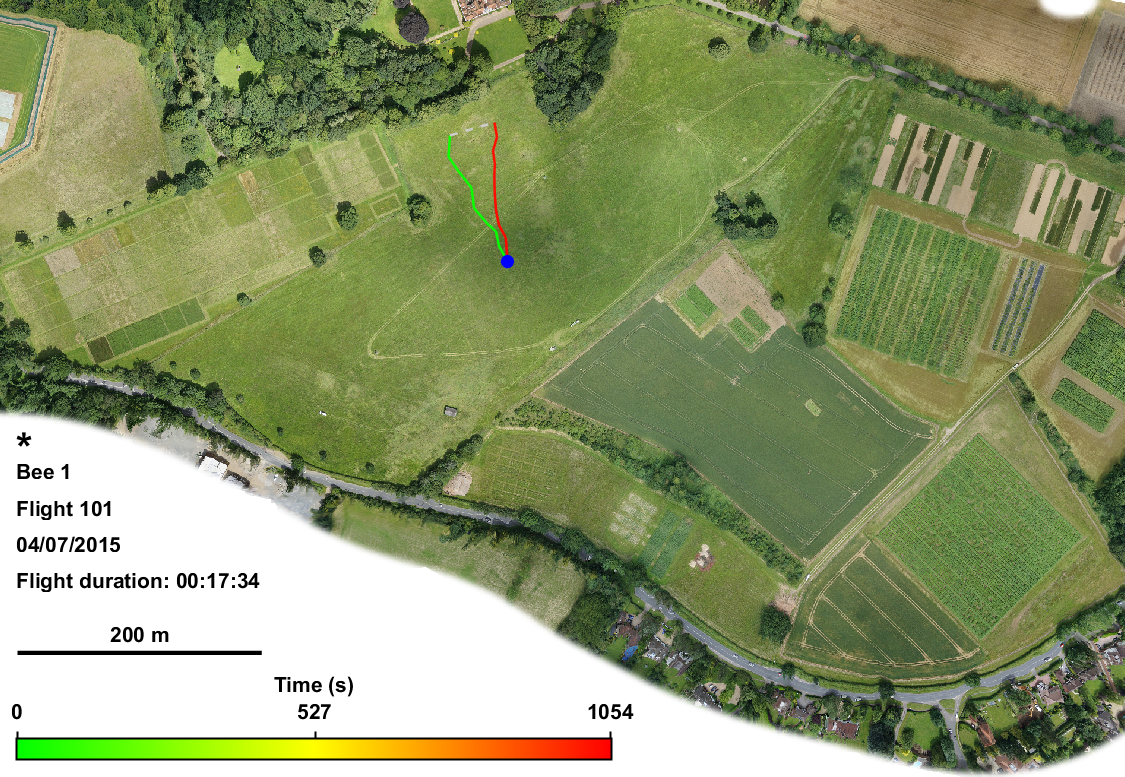

Supplement: S3 File — All flights made by Bee 1 on 04/07/2015. Each figure represents a single flight. Bee ID, flight number, date of recording and the duration of each flight are shown in the bottom left corner of each figure. The position of the nest is marked by a blue circle. Colours represent the time from the start of each flight: initial portion of each flight is in green, changing smoothly through yellow until the end of the flight is shown in red. Grey dashed lines are used to join radar observations made more than 30 s apart, when the bee’s location was uncertain. (ZIP) [file pone.0160333.s005.zip › S102 Fig.tif]

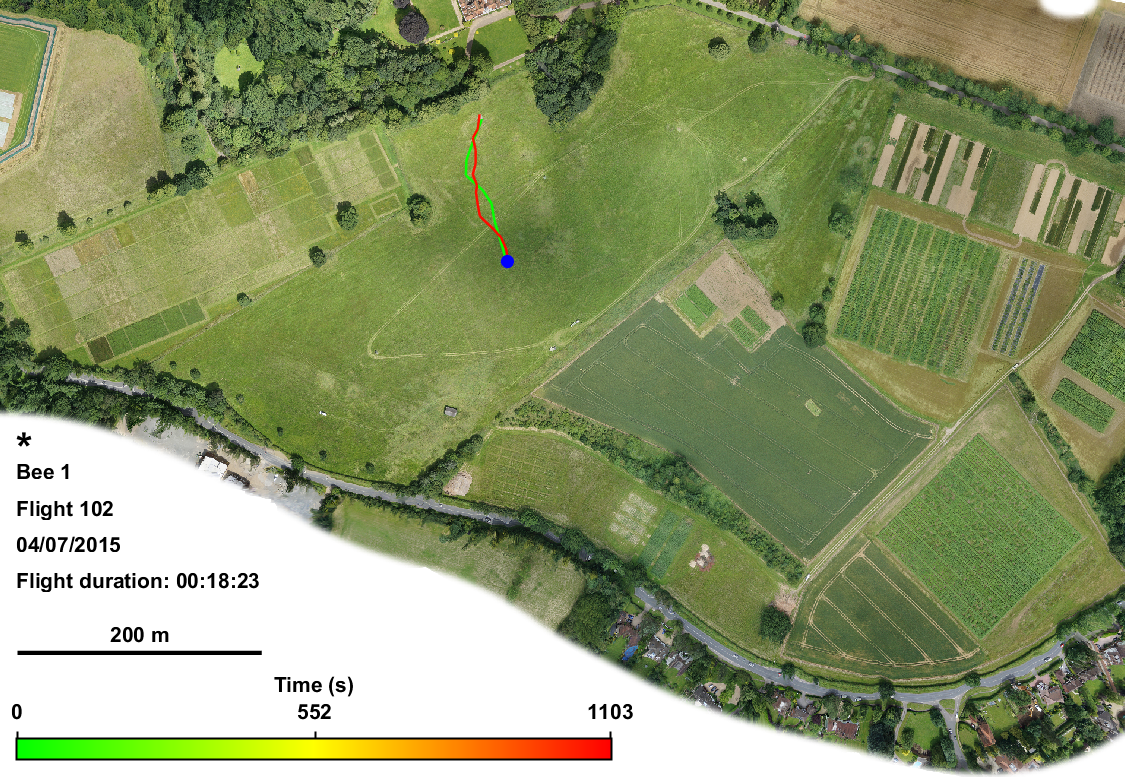

Supplement: S3 File — All flights made by Bee 1 on 04/07/2015. Each figure represents a single flight. Bee ID, flight number, date of recording and the duration of each flight are shown in the bottom left corner of each figure. The position of the nest is marked by a blue circle. Colours represent the time from the start of each flight: initial portion of each flight is in green, changing smoothly through yellow until the end of the flight is shown in red. Grey dashed lines are used to join radar observations made more than 30 s apart, when the bee’s location was uncertain. (ZIP) [file pone.0160333.s005.zip › S103 Fig.tif]

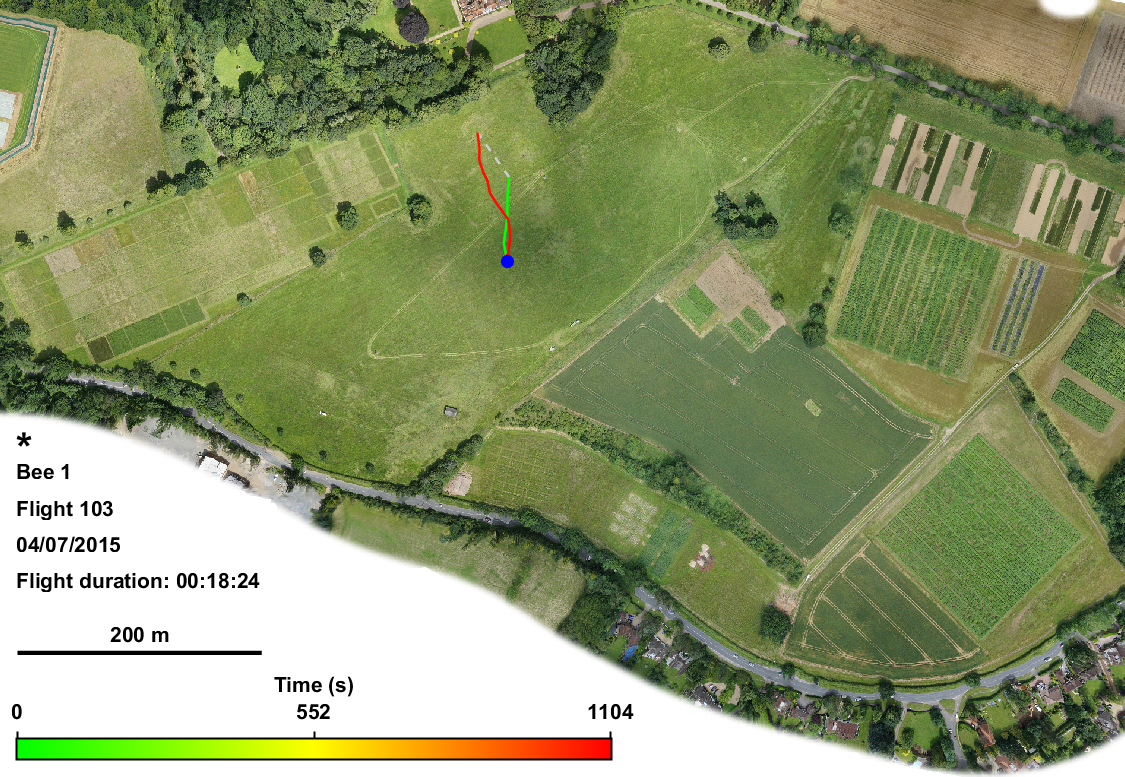

Supplement: S3 File — All flights made by Bee 1 on 04/07/2015. Each figure represents a single flight. Bee ID, flight number, date of recording and the duration of each flight are shown in the bottom left corner of each figure. The position of the nest is marked by a blue circle. Colours represent the time from the start of each flight: initial portion of each flight is in green, changing smoothly through yellow until the end of the flight is shown in red. Grey dashed lines are used to join radar observations made more than 30 s apart, when the bee’s location was uncertain. (ZIP) [file pone.0160333.s005.zip › S104 Fig.tif]

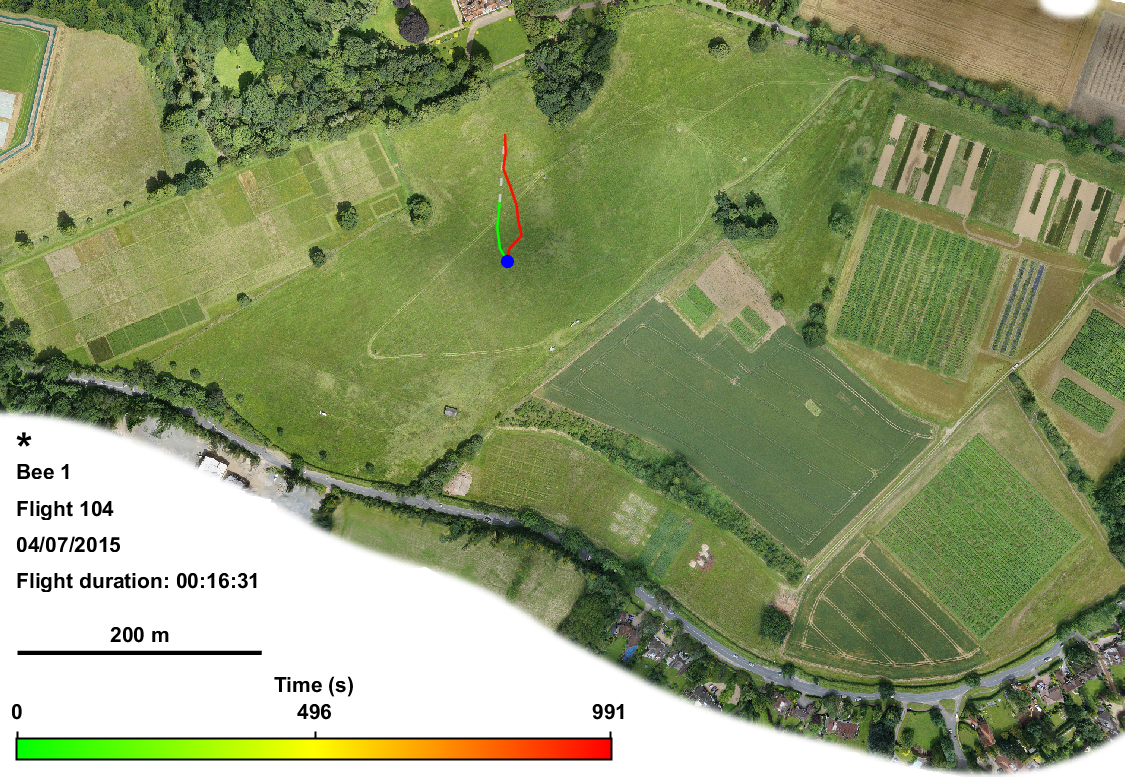

Supplement: S3 File — All flights made by Bee 1 on 04/07/2015. Each figure represents a single flight. Bee ID, flight number, date of recording and the duration of each flight are shown in the bottom left corner of each figure. The position of the nest is marked by a blue circle. Colours represent the time from the start of each flight: initial portion of each flight is in green, changing smoothly through yellow until the end of the flight is shown in red. Grey dashed lines are used to join radar observations made more than 30 s apart, when the bee’s location was uncertain. (ZIP) [file pone.0160333.s005.zip › S105 Fig.tif]

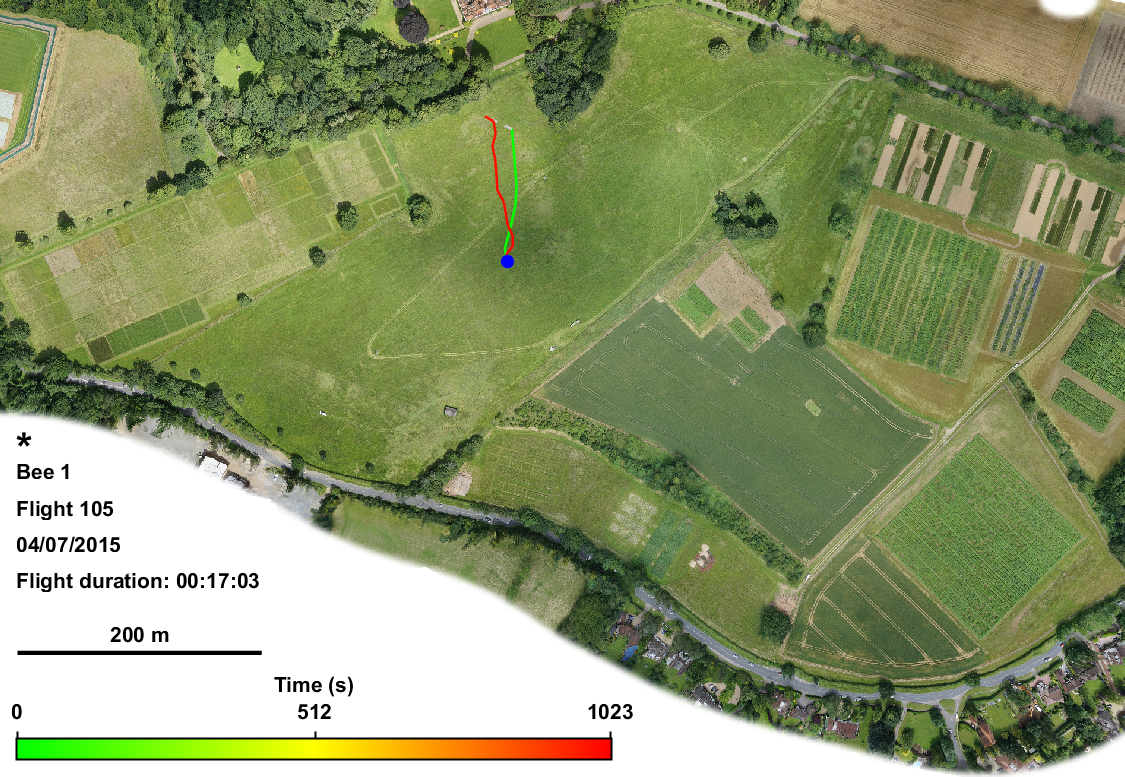

Supplement: S3 File — All flights made by Bee 1 on 04/07/2015. Each figure represents a single flight. Bee ID, flight number, date of recording and the duration of each flight are shown in the bottom left corner of each figure. The position of the nest is marked by a blue circle. Colours represent the time from the start of each flight: initial portion of each flight is in green, changing smoothly through yellow until the end of the flight is shown in red. Grey dashed lines are used to join radar observations made more than 30 s apart, when the bee’s location was uncertain. (ZIP) [file pone.0160333.s005.zip › S106 Fig.tif]

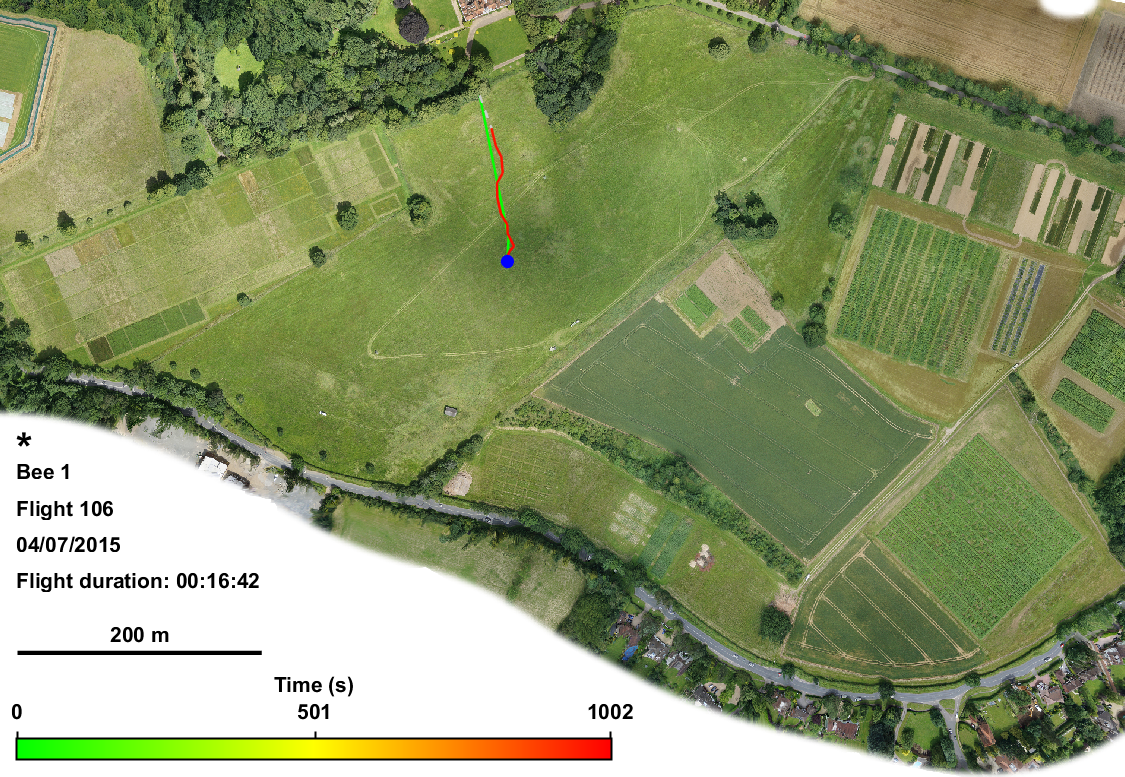

Supplement: S3 File — All flights made by Bee 1 on 04/07/2015. Each figure represents a single flight. Bee ID, flight number, date of recording and the duration of each flight are shown in the bottom left corner of each figure. The position of the nest is marked by a blue circle. Colours represent the time from the start of each flight: initial portion of each flight is in green, changing smoothly through yellow until the end of the flight is shown in red. Grey dashed lines are used to join radar observations made more than 30 s apart, when the bee’s location was uncertain. (ZIP) [file pone.0160333.s005.zip › S107 Fig.tif]

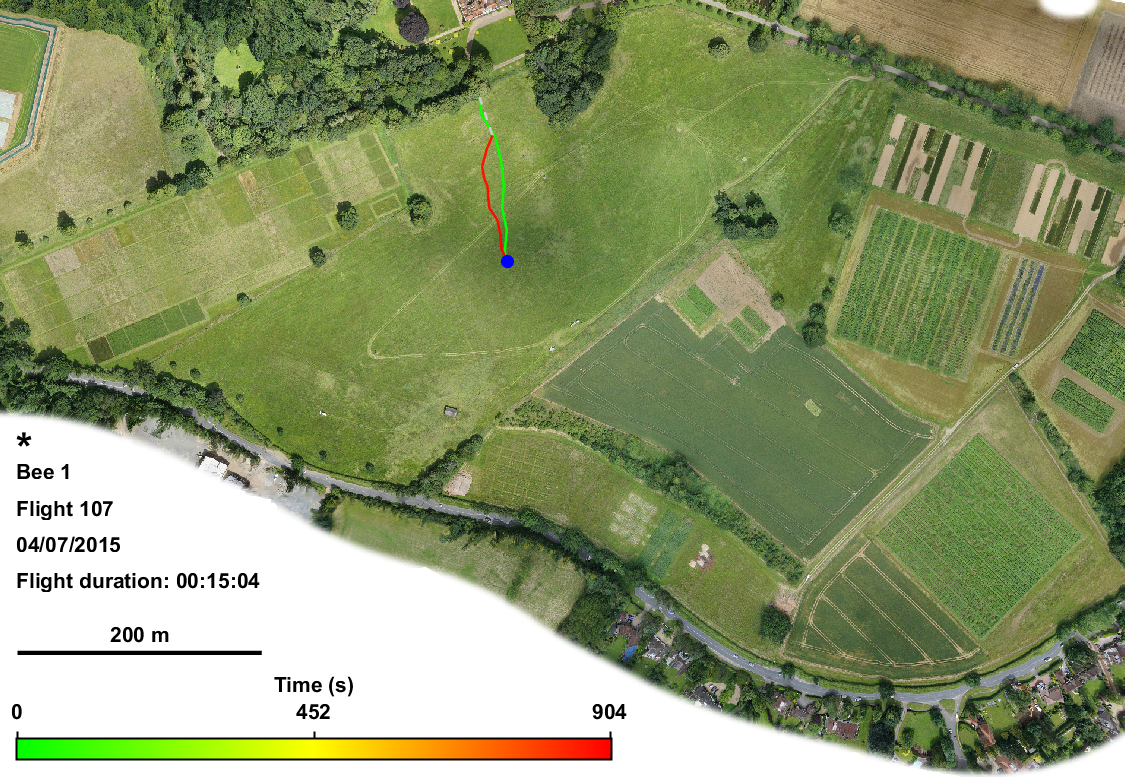

Supplement: S3 File — All flights made by Bee 1 on 04/07/2015. Each figure represents a single flight. Bee ID, flight number, date of recording and the duration of each flight are shown in the bottom left corner of each figure. The position of the nest is marked by a blue circle. Colours represent the time from the start of each flight: initial portion of each flight is in green, changing smoothly through yellow until the end of the flight is shown in red. Grey dashed lines are used to join radar observations made more than 30 s apart, when the bee’s location was uncertain. (ZIP) [file pone.0160333.s005.zip › S108 Fig.tif]

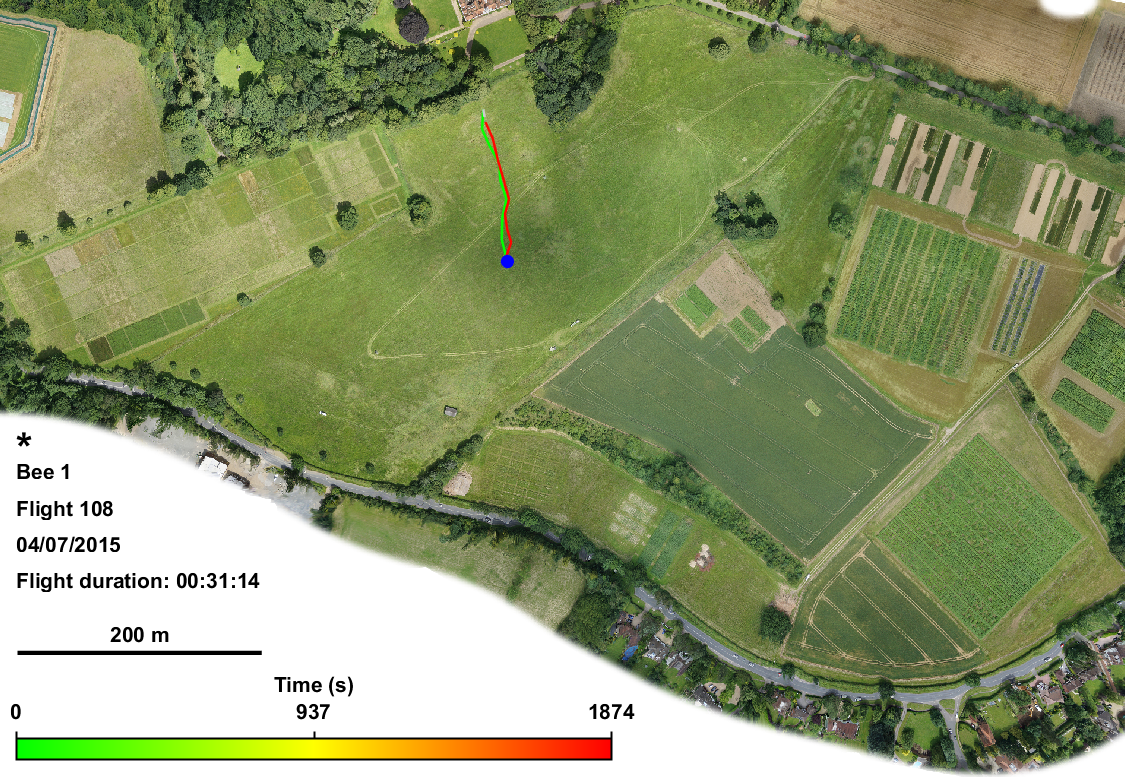

Supplement: S3 File — All flights made by Bee 1 on 04/07/2015. Each figure represents a single flight. Bee ID, flight number, date of recording and the duration of each flight are shown in the bottom left corner of each figure. The position of the nest is marked by a blue circle. Colours represent the time from the start of each flight: initial portion of each flight is in green, changing smoothly through yellow until the end of the flight is shown in red. Grey dashed lines are used to join radar observations made more than 30 s apart, when the bee’s location was uncertain. (ZIP) [file pone.0160333.s005.zip › S109 Fig.tif]

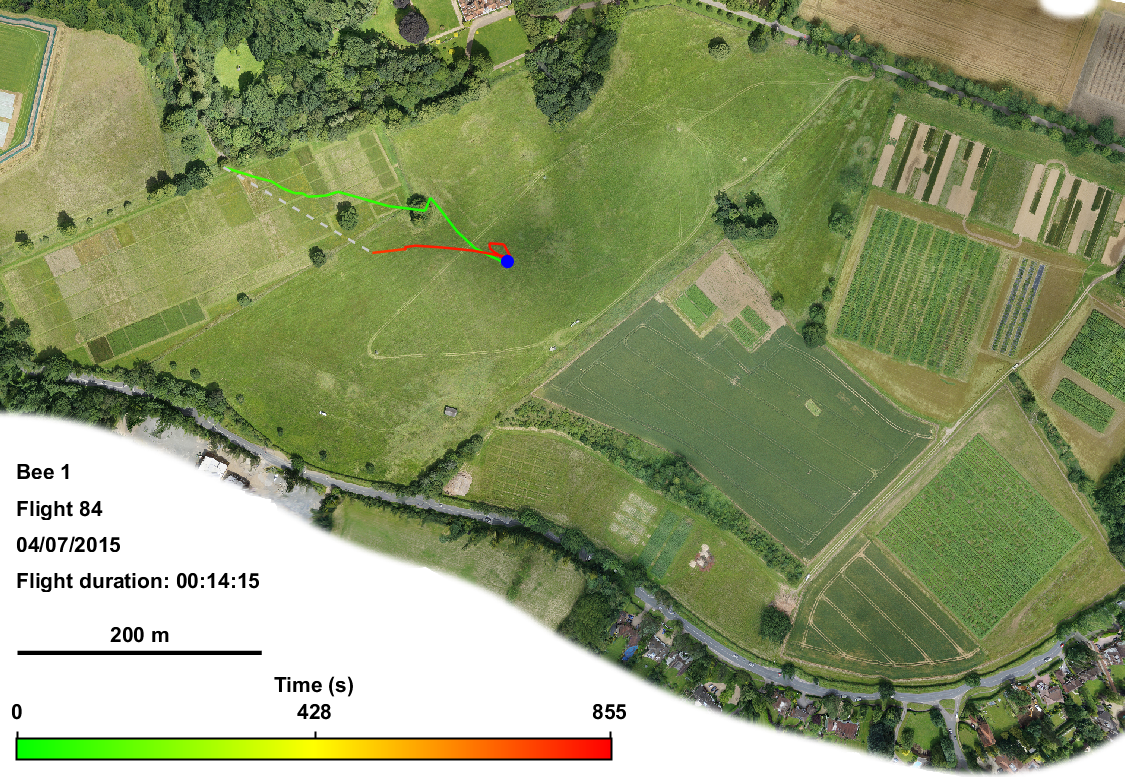

Supplement: S3 File — All flights made by Bee 1 on 04/07/2015. Each figure represents a single flight. Bee ID, flight number, date of recording and the duration of each flight are shown in the bottom left corner of each figure. The position of the nest is marked by a blue circle. Colours represent the time from the start of each flight: initial portion of each flight is in green, changing smoothly through yellow until the end of the flight is shown in red. Grey dashed lines are used to join radar observations made more than 30 s apart, when the bee’s location was uncertain. (ZIP) [file pone.0160333.s005.zip › S85 Fig.tif]

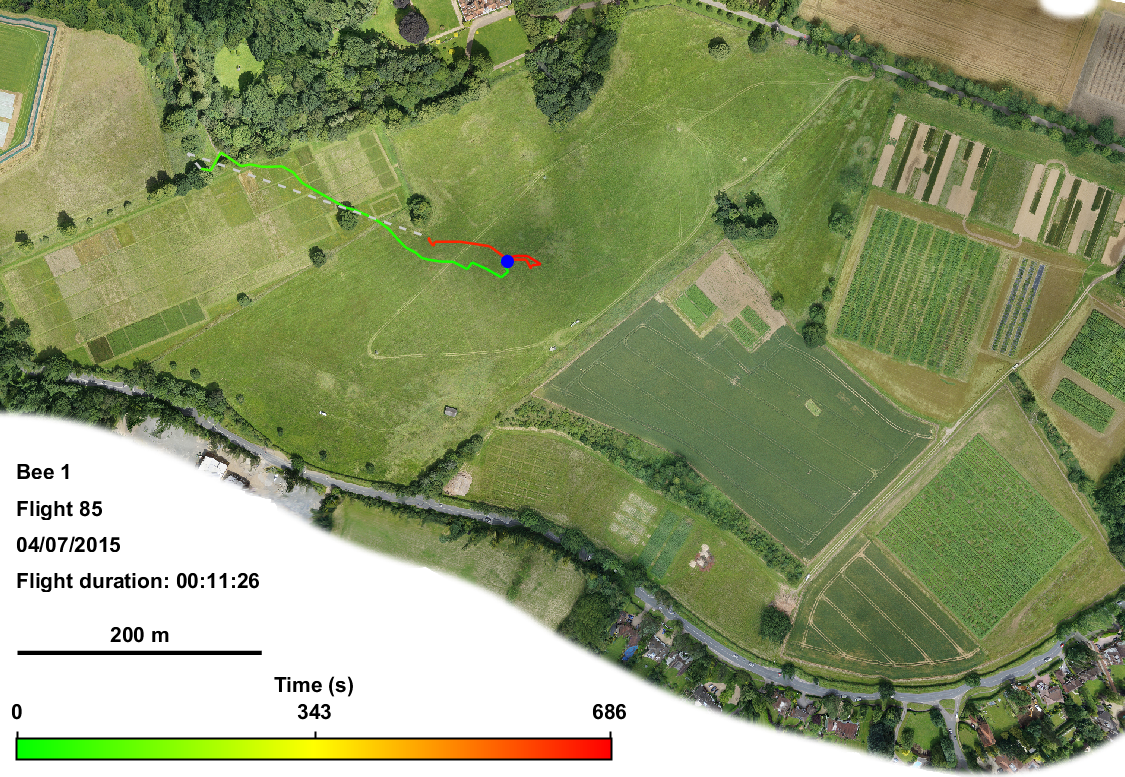

Supplement: S3 File — All flights made by Bee 1 on 04/07/2015. Each figure represents a single flight. Bee ID, flight number, date of recording and the duration of each flight are shown in the bottom left corner of each figure. The position of the nest is marked by a blue circle. Colours represent the time from the start of each flight: initial portion of each flight is in green, changing smoothly through yellow until the end of the flight is shown in red. Grey dashed lines are used to join radar observations made more than 30 s apart, when the bee’s location was uncertain. (ZIP) [file pone.0160333.s005.zip › S86 Fig.tif]

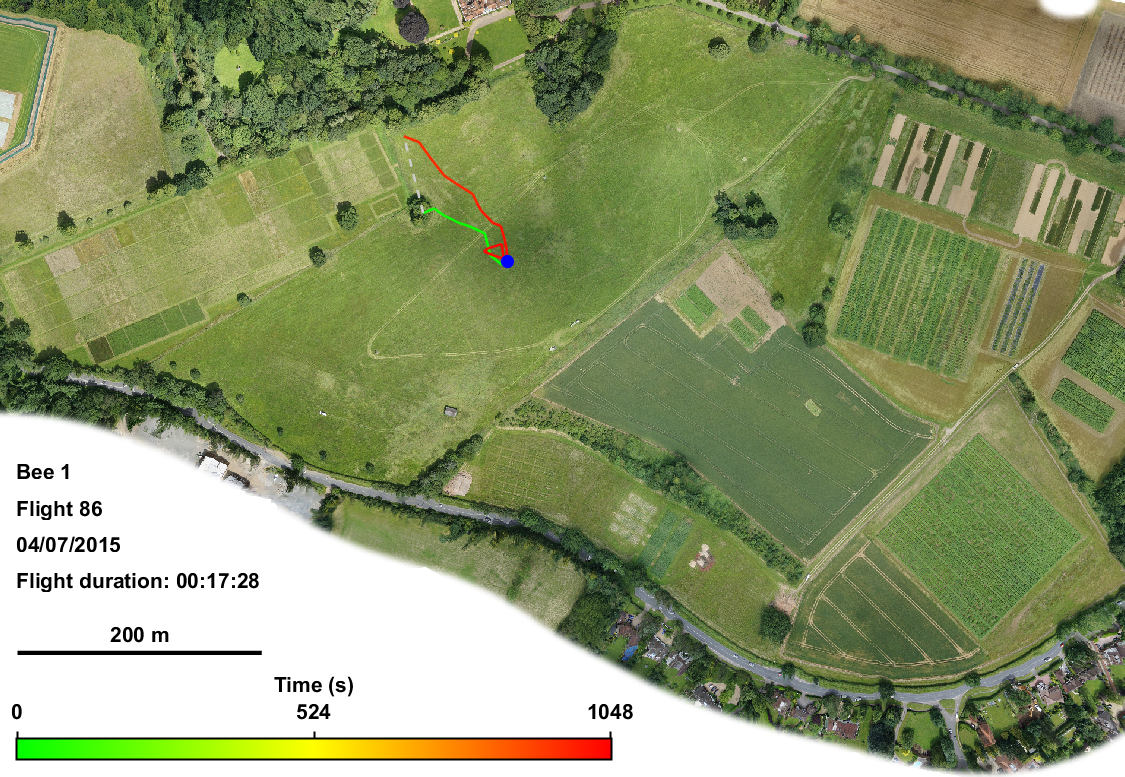

Supplement: S3 File — All flights made by Bee 1 on 04/07/2015. Each figure represents a single flight. Bee ID, flight number, date of recording and the duration of each flight are shown in the bottom left corner of each figure. The position of the nest is marked by a blue circle. Colours represent the time from the start of each flight: initial portion of each flight is in green, changing smoothly through yellow until the end of the flight is shown in red. Grey dashed lines are used to join radar observations made more than 30 s apart, when the bee’s location was uncertain. (ZIP) [file pone.0160333.s005.zip › S87 Fig.tif]

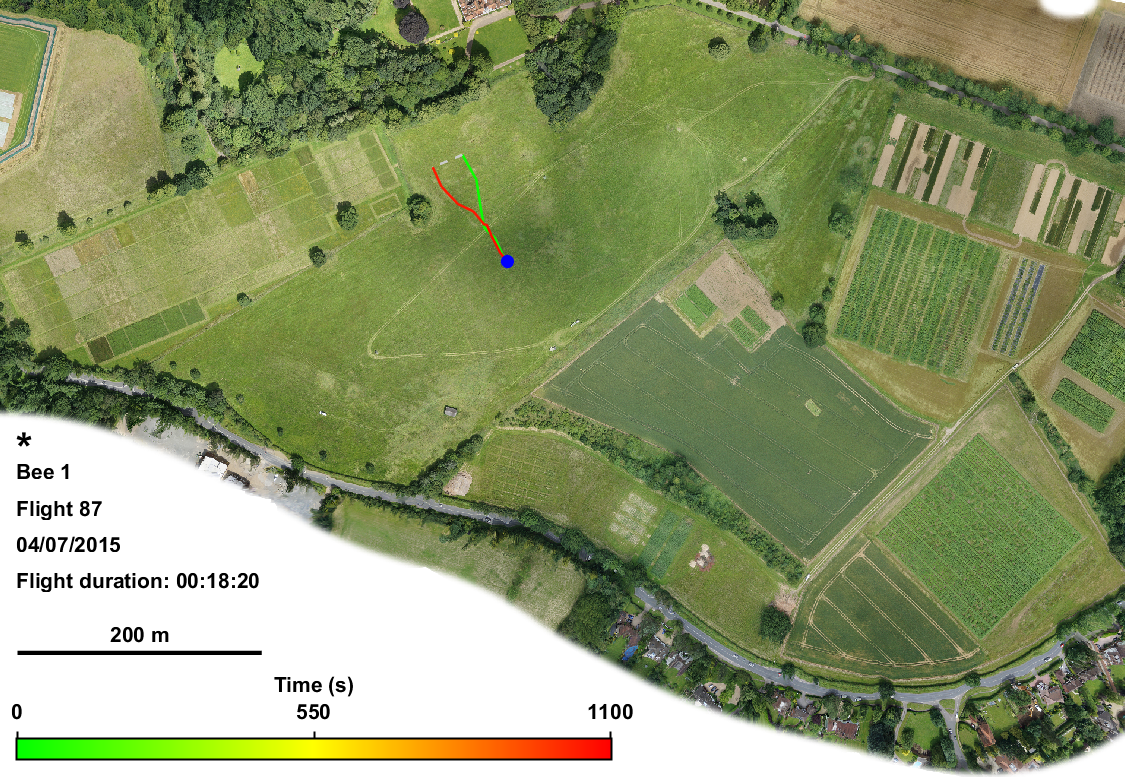

Supplement: S3 File — All flights made by Bee 1 on 04/07/2015. Each figure represents a single flight. Bee ID, flight number, date of recording and the duration of each flight are shown in the bottom left corner of each figure. The position of the nest is marked by a blue circle. Colours represent the time from the start of each flight: initial portion of each flight is in green, changing smoothly through yellow until the end of the flight is shown in red. Grey dashed lines are used to join radar observations made more than 30 s apart, when the bee’s location was uncertain. (ZIP) [file pone.0160333.s005.zip › S88 Fig.tif]

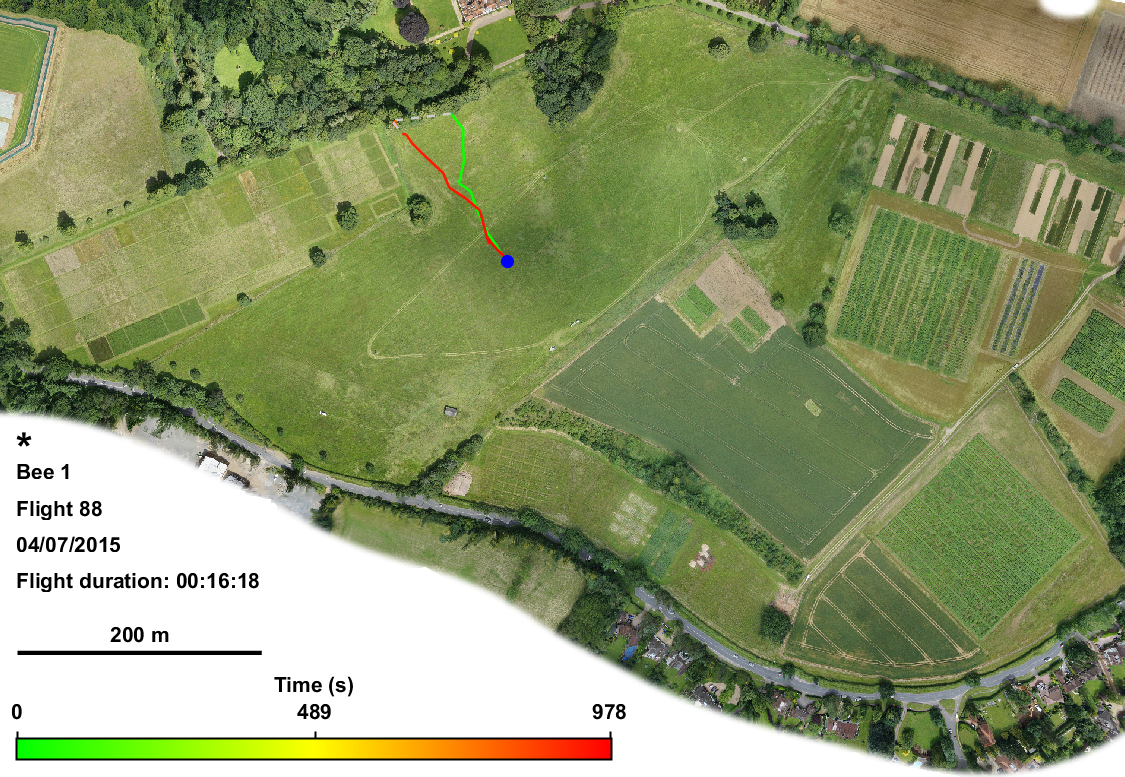

Supplement: S3 File — All flights made by Bee 1 on 04/07/2015. Each figure represents a single flight. Bee ID, flight number, date of recording and the duration of each flight are shown in the bottom left corner of each figure. The position of the nest is marked by a blue circle. Colours represent the time from the start of each flight: initial portion of each flight is in green, changing smoothly through yellow until the end of the flight is shown in red. Grey dashed lines are used to join radar observations made more than 30 s apart, when the bee’s location was uncertain. (ZIP) [file pone.0160333.s005.zip › S89 Fig.tif]

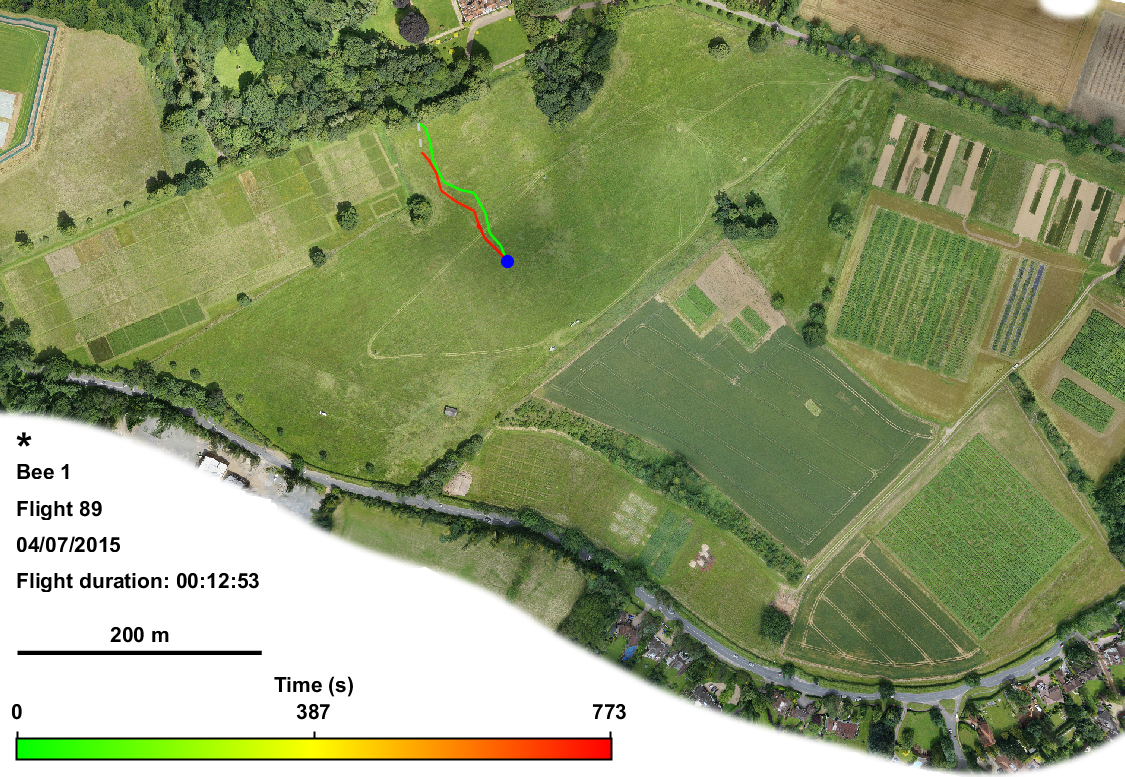

Supplement: S3 File — All flights made by Bee 1 on 04/07/2015. Each figure represents a single flight. Bee ID, flight number, date of recording and the duration of each flight are shown in the bottom left corner of each figure. The position of the nest is marked by a blue circle. Colours represent the time from the start of each flight: initial portion of each flight is in green, changing smoothly through yellow until the end of the flight is shown in red. Grey dashed lines are used to join radar observations made more than 30 s apart, when the bee’s location was uncertain. (ZIP) [file pone.0160333.s005.zip › S90 Fig.tif]
