# Supplementary figures and images for: Life-Long Radar Tracking of Bumblebees (part 2 of 3)
Source: PLoS One. 2016 Aug 4;11(8):e0160333. doi: 10.1371/journal.pone.0160333 (PMC4973990; doi:10.1371/journal.pone.0160333)

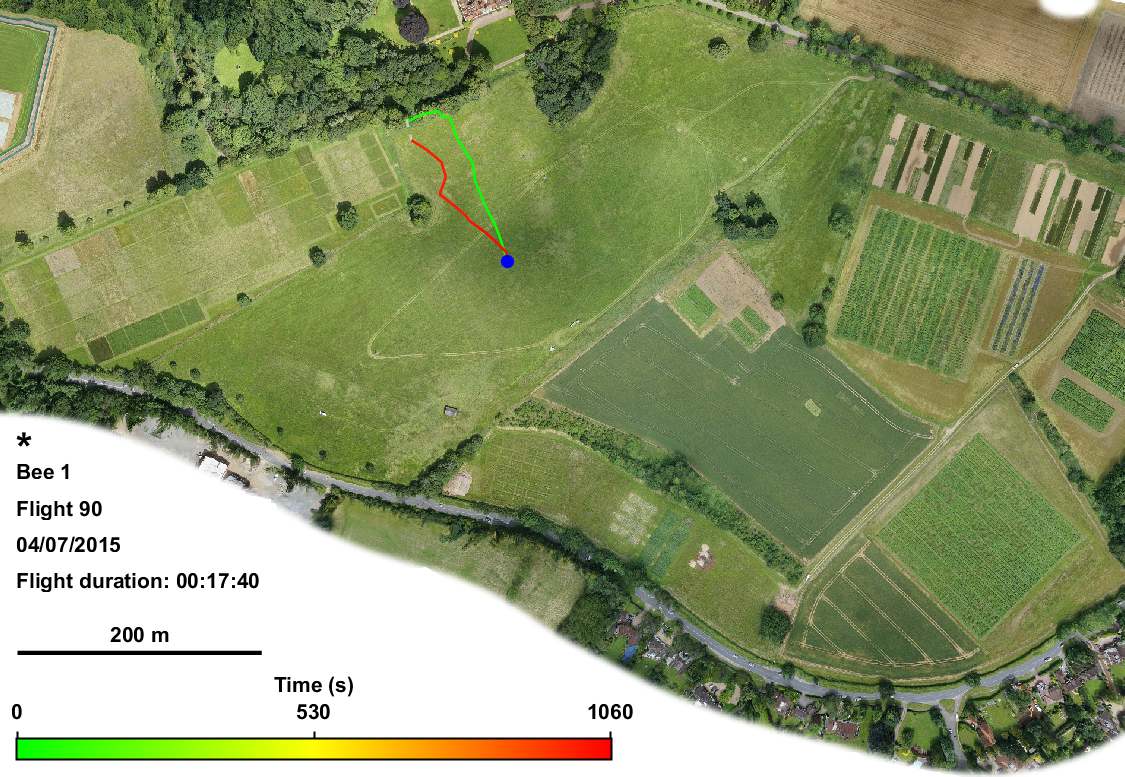

Supplement: S3 File — All flights made by Bee 1 on 04/07/2015. Each figure represents a single flight. Bee ID, flight number, date of recording and the duration of each flight are shown in the bottom left corner of each figure. The position of the nest is marked by a blue circle. Colours represent the time from the start of each flight: initial portion of each flight is in green, changing smoothly through yellow until the end of the flight is shown in red. Grey dashed lines are used to join radar observations made more than 30 s apart, when the bee’s location was uncertain. (ZIP) [file pone.0160333.s005.zip › S91 Fig.tif]

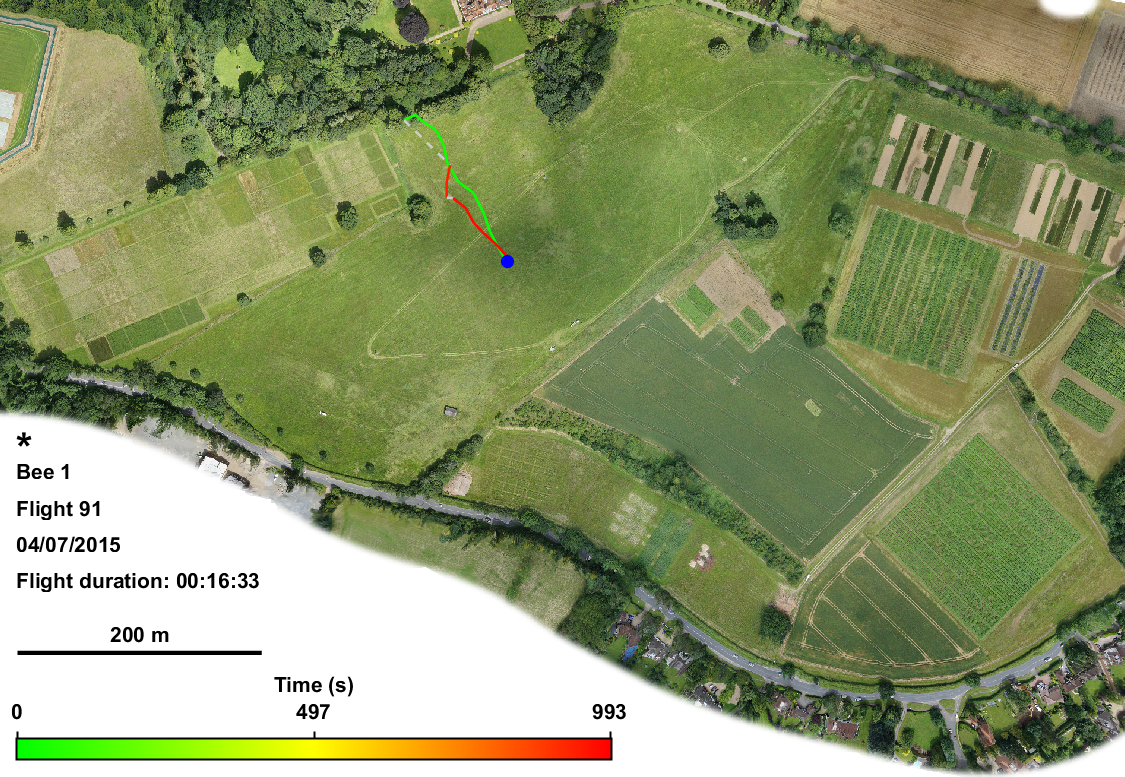

Supplement: S3 File — All flights made by Bee 1 on 04/07/2015. Each figure represents a single flight. Bee ID, flight number, date of recording and the duration of each flight are shown in the bottom left corner of each figure. The position of the nest is marked by a blue circle. Colours represent the time from the start of each flight: initial portion of each flight is in green, changing smoothly through yellow until the end of the flight is shown in red. Grey dashed lines are used to join radar observations made more than 30 s apart, when the bee’s location was uncertain. (ZIP) [file pone.0160333.s005.zip › S92 Fig.tif]

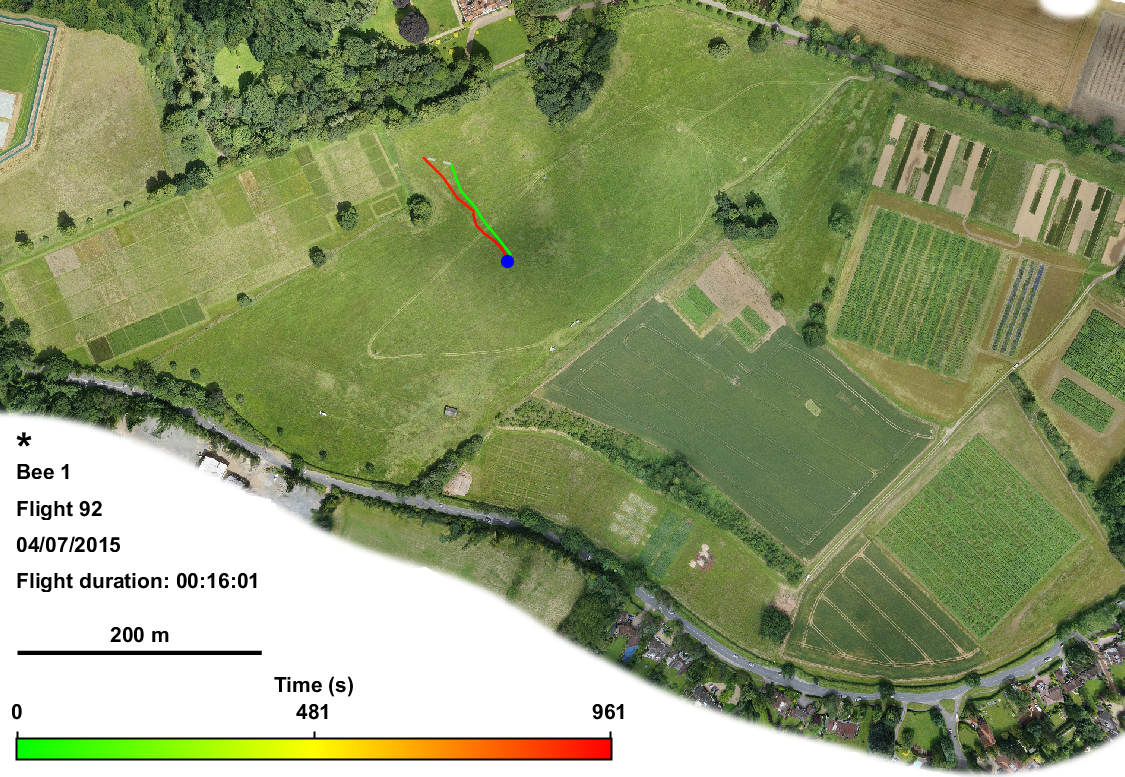

Supplement: S3 File — All flights made by Bee 1 on 04/07/2015. Each figure represents a single flight. Bee ID, flight number, date of recording and the duration of each flight are shown in the bottom left corner of each figure. The position of the nest is marked by a blue circle. Colours represent the time from the start of each flight: initial portion of each flight is in green, changing smoothly through yellow until the end of the flight is shown in red. Grey dashed lines are used to join radar observations made more than 30 s apart, when the bee’s location was uncertain. (ZIP) [file pone.0160333.s005.zip › S93 Fig.tif]

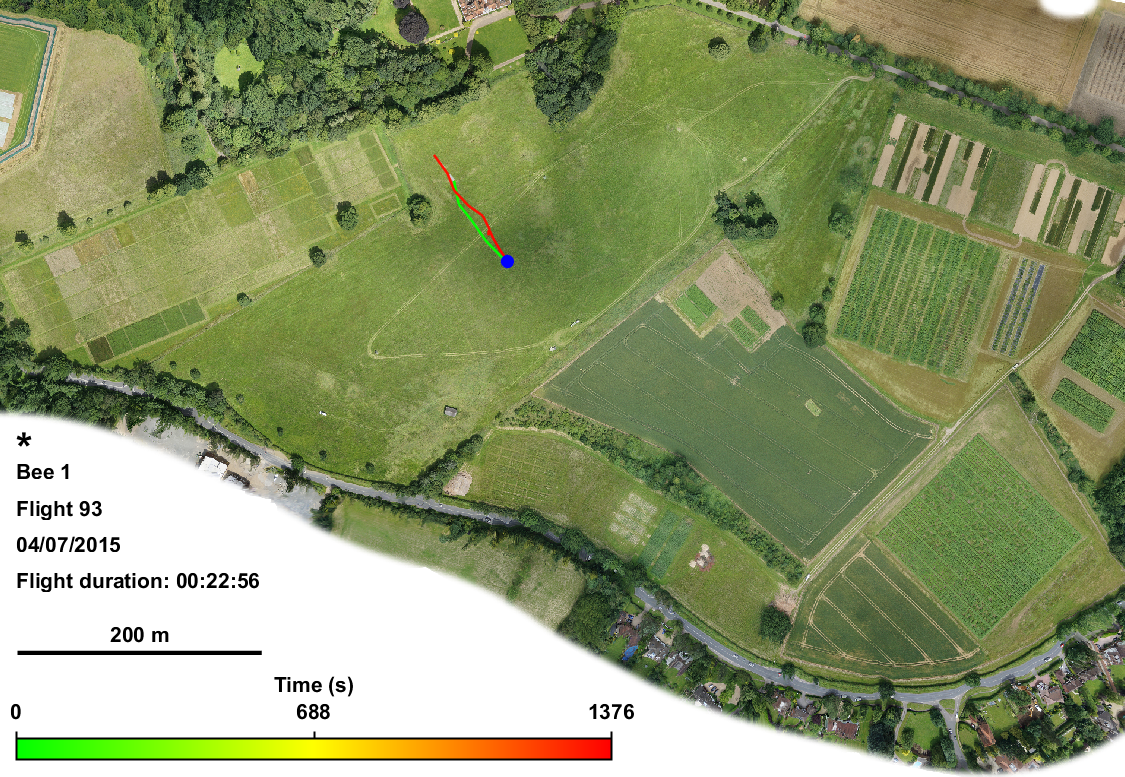

Supplement: S3 File — All flights made by Bee 1 on 04/07/2015. Each figure represents a single flight. Bee ID, flight number, date of recording and the duration of each flight are shown in the bottom left corner of each figure. The position of the nest is marked by a blue circle. Colours represent the time from the start of each flight: initial portion of each flight is in green, changing smoothly through yellow until the end of the flight is shown in red. Grey dashed lines are used to join radar observations made more than 30 s apart, when the bee’s location was uncertain. (ZIP) [file pone.0160333.s005.zip › S94 Fig.tif]

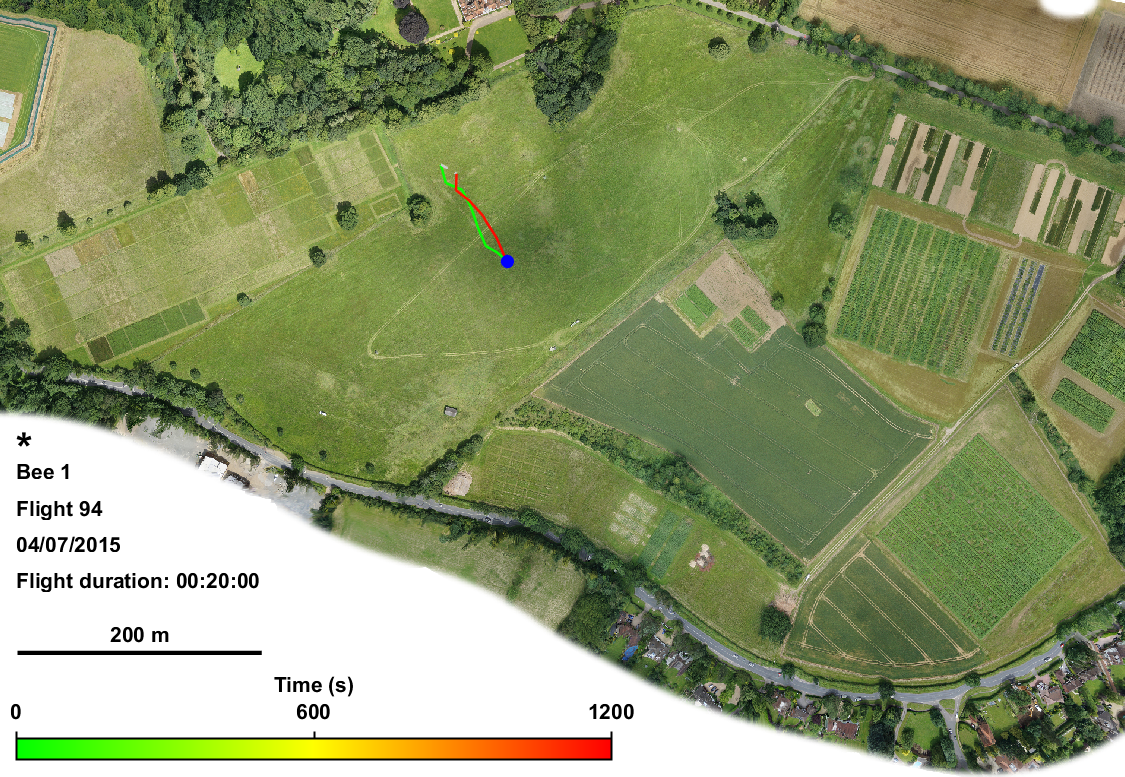

Supplement: S3 File — All flights made by Bee 1 on 04/07/2015. Each figure represents a single flight. Bee ID, flight number, date of recording and the duration of each flight are shown in the bottom left corner of each figure. The position of the nest is marked by a blue circle. Colours represent the time from the start of each flight: initial portion of each flight is in green, changing smoothly through yellow until the end of the flight is shown in red. Grey dashed lines are used to join radar observations made more than 30 s apart, when the bee’s location was uncertain. (ZIP) [file pone.0160333.s005.zip › S95 Fig.tif]

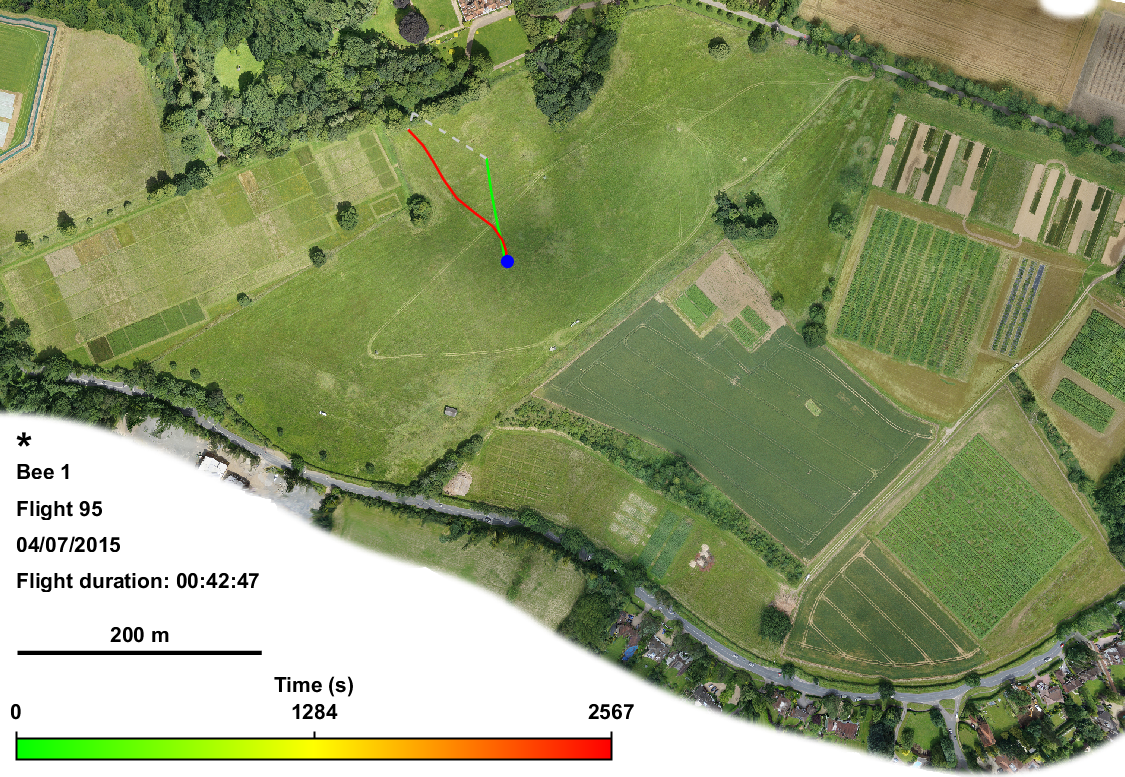

Supplement: S3 File — All flights made by Bee 1 on 04/07/2015. Each figure represents a single flight. Bee ID, flight number, date of recording and the duration of each flight are shown in the bottom left corner of each figure. The position of the nest is marked by a blue circle. Colours represent the time from the start of each flight: initial portion of each flight is in green, changing smoothly through yellow until the end of the flight is shown in red. Grey dashed lines are used to join radar observations made more than 30 s apart, when the bee’s location was uncertain. (ZIP) [file pone.0160333.s005.zip › S96 Fig.tif]

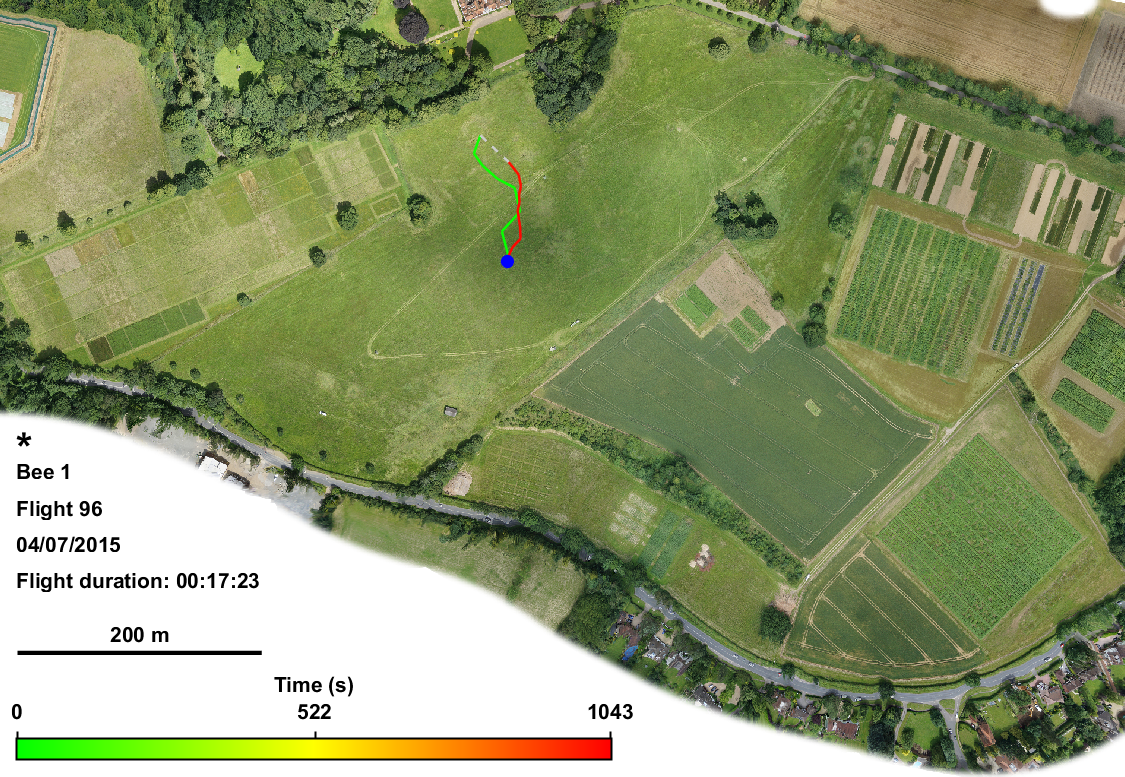

Supplement: S3 File — All flights made by Bee 1 on 04/07/2015. Each figure represents a single flight. Bee ID, flight number, date of recording and the duration of each flight are shown in the bottom left corner of each figure. The position of the nest is marked by a blue circle. Colours represent the time from the start of each flight: initial portion of each flight is in green, changing smoothly through yellow until the end of the flight is shown in red. Grey dashed lines are used to join radar observations made more than 30 s apart, when the bee’s location was uncertain. (ZIP) [file pone.0160333.s005.zip › S97 Fig.tif]

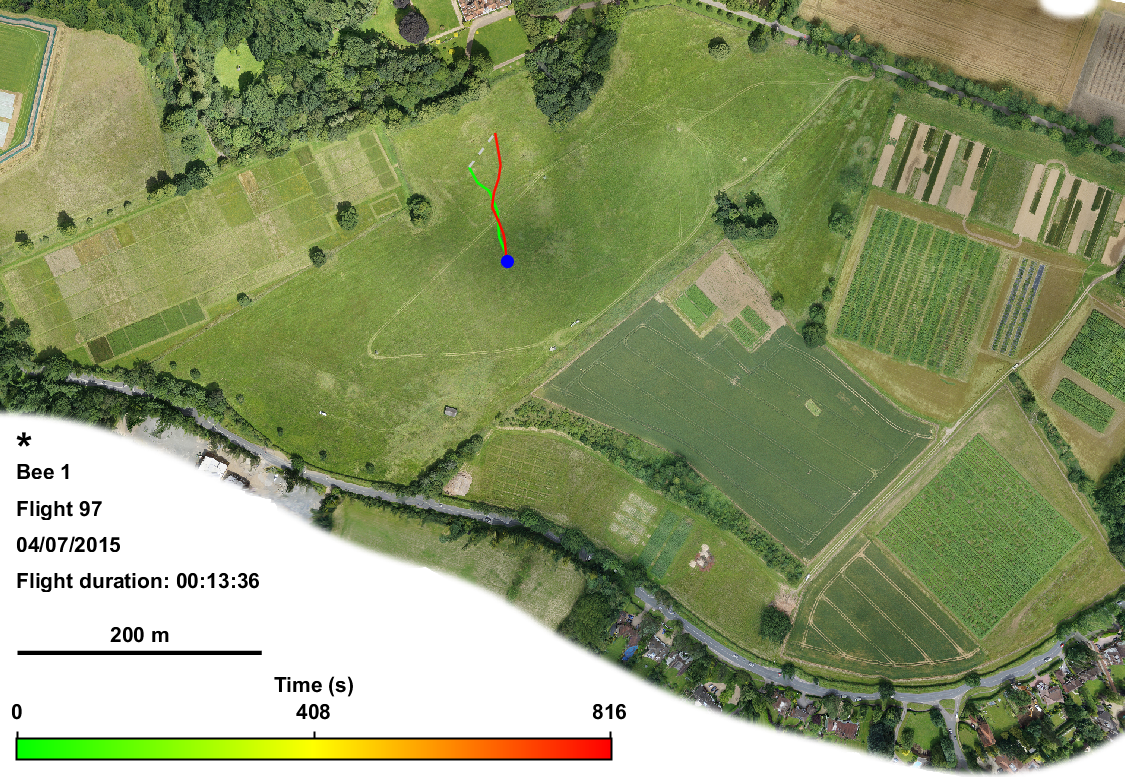

Supplement: S3 File — All flights made by Bee 1 on 04/07/2015. Each figure represents a single flight. Bee ID, flight number, date of recording and the duration of each flight are shown in the bottom left corner of each figure. The position of the nest is marked by a blue circle. Colours represent the time from the start of each flight: initial portion of each flight is in green, changing smoothly through yellow until the end of the flight is shown in red. Grey dashed lines are used to join radar observations made more than 30 s apart, when the bee’s location was uncertain. (ZIP) [file pone.0160333.s005.zip › S98 Fig.tif]

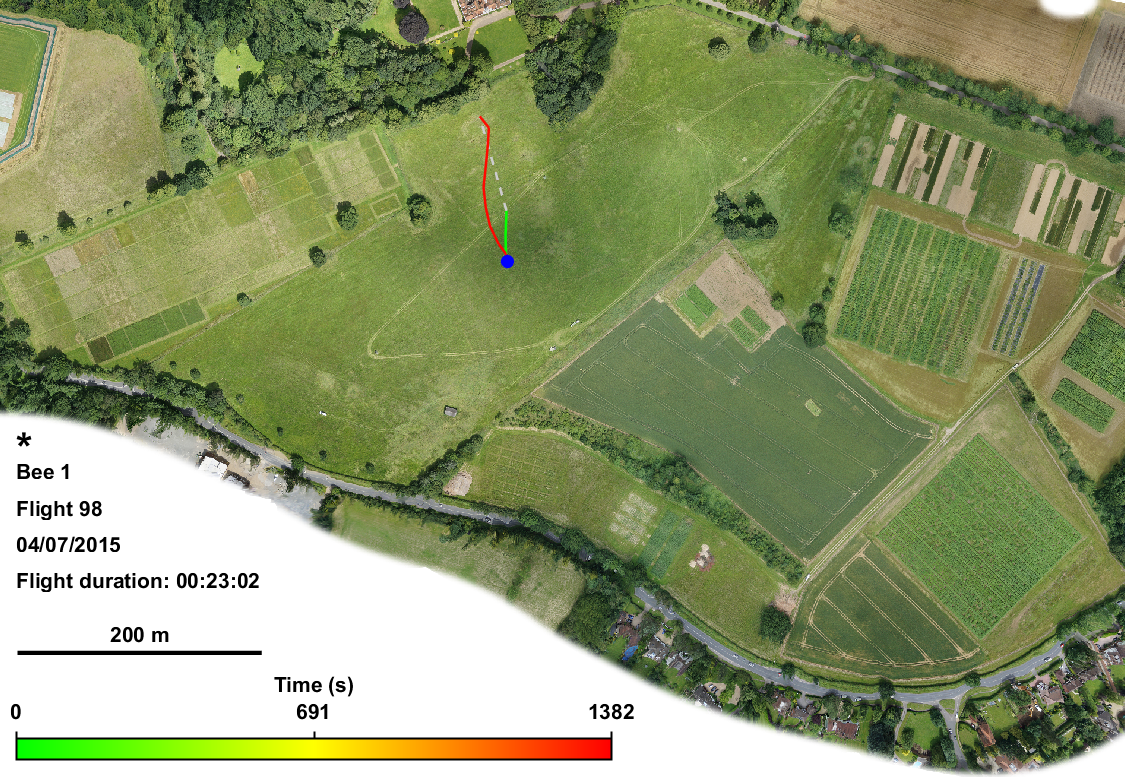

Supplement: S3 File — All flights made by Bee 1 on 04/07/2015. Each figure represents a single flight. Bee ID, flight number, date of recording and the duration of each flight are shown in the bottom left corner of each figure. The position of the nest is marked by a blue circle. Colours represent the time from the start of each flight: initial portion of each flight is in green, changing smoothly through yellow until the end of the flight is shown in red. Grey dashed lines are used to join radar observations made more than 30 s apart, when the bee’s location was uncertain. (ZIP) [file pone.0160333.s005.zip › S99 Fig.tif]

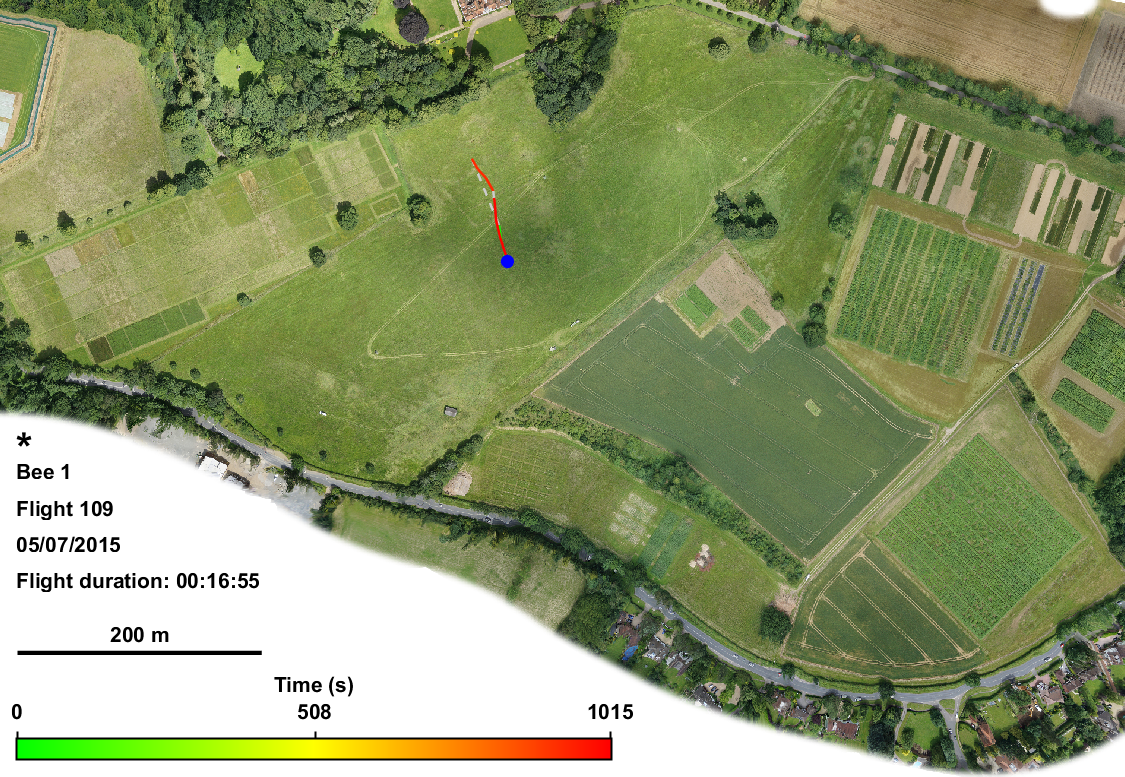

Supplement: S4 File — All flights made by Bee 1 during the period 05/07/15–06/07/15. Each figure represents a single flight. Bee ID, flight number, date of recording and the duration of each flight are shown in the bottom left corner of each figure. The position of the nest is marked by a blue circle. Colours represent the time from the start of each flight: initial portion of each flight is in green, changing smoothly through yellow until the end of the flight is shown in red. Grey dashed lines are used to join radar observations made more than 30 s apart, when the bee’s location was uncertain. (ZIP) [file pone.0160333.s006.zip › S110 Fig.tif]

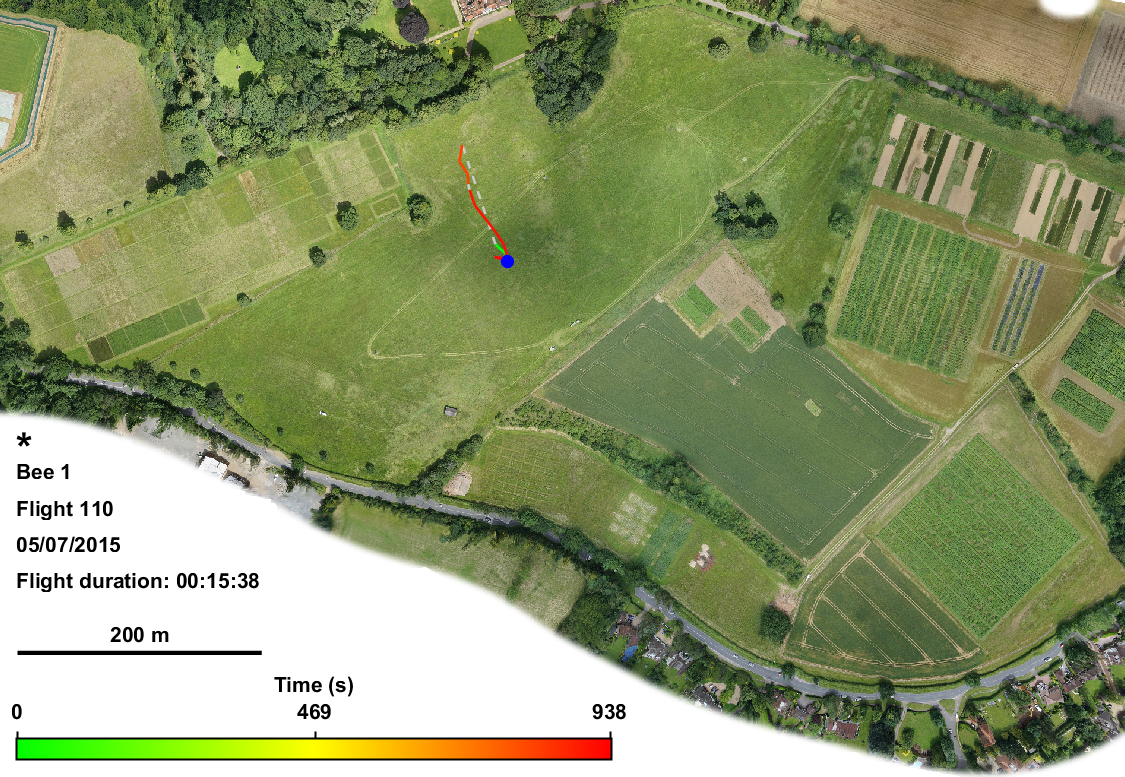

Supplement: S4 File — All flights made by Bee 1 during the period 05/07/15–06/07/15. Each figure represents a single flight. Bee ID, flight number, date of recording and the duration of each flight are shown in the bottom left corner of each figure. The position of the nest is marked by a blue circle. Colours represent the time from the start of each flight: initial portion of each flight is in green, changing smoothly through yellow until the end of the flight is shown in red. Grey dashed lines are used to join radar observations made more than 30 s apart, when the bee’s location was uncertain. (ZIP) [file pone.0160333.s006.zip › S111 Fig.tif]

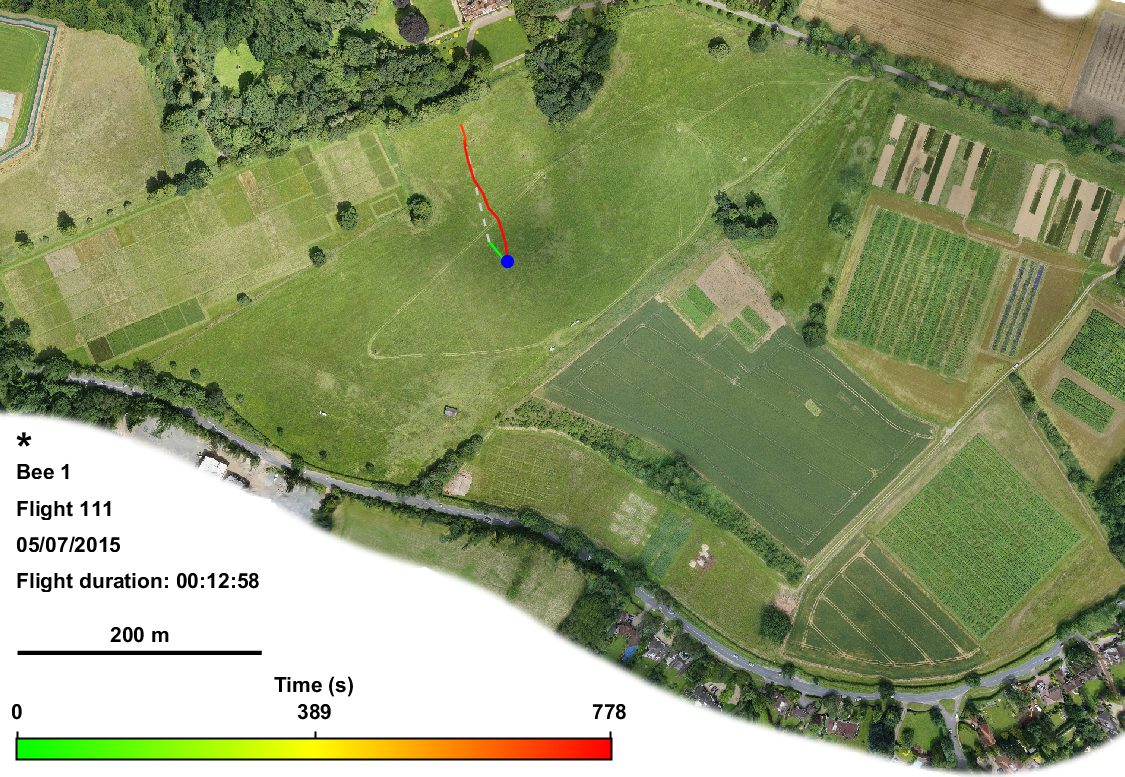

Supplement: S4 File — All flights made by Bee 1 during the period 05/07/15–06/07/15. Each figure represents a single flight. Bee ID, flight number, date of recording and the duration of each flight are shown in the bottom left corner of each figure. The position of the nest is marked by a blue circle. Colours represent the time from the start of each flight: initial portion of each flight is in green, changing smoothly through yellow until the end of the flight is shown in red. Grey dashed lines are used to join radar observations made more than 30 s apart, when the bee’s location was uncertain. (ZIP) [file pone.0160333.s006.zip › S112 Fig.tif]

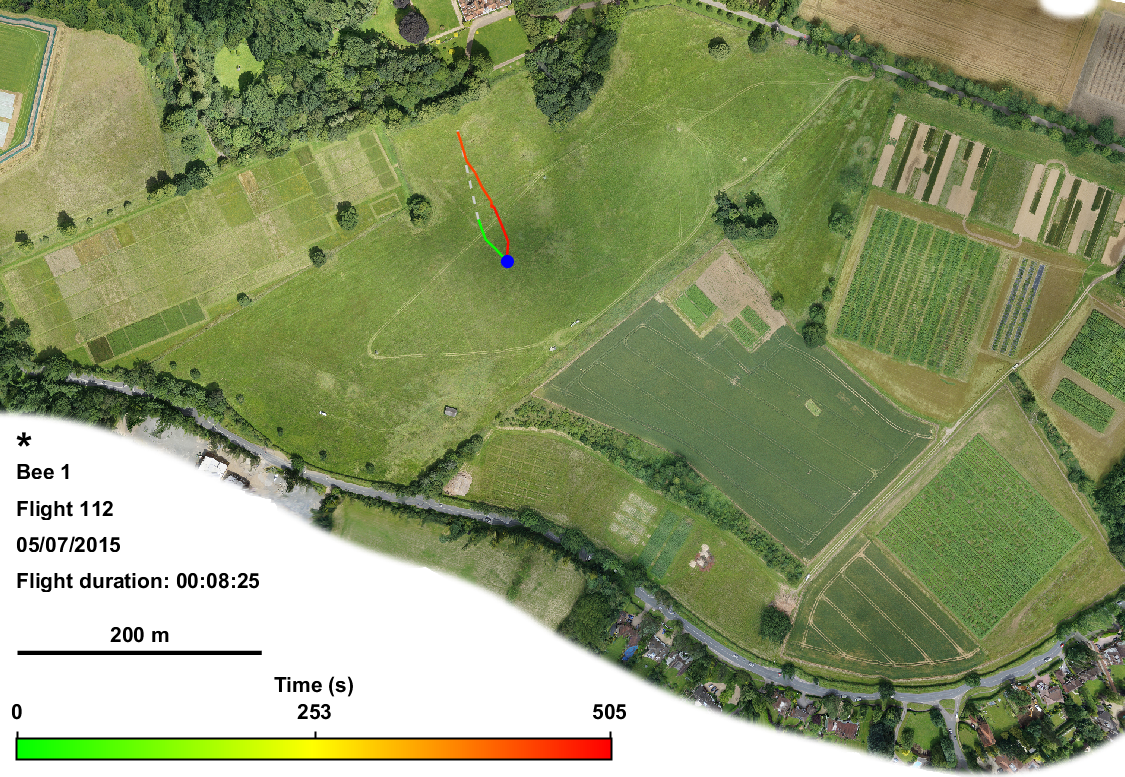

Supplement: S4 File — All flights made by Bee 1 during the period 05/07/15–06/07/15. Each figure represents a single flight. Bee ID, flight number, date of recording and the duration of each flight are shown in the bottom left corner of each figure. The position of the nest is marked by a blue circle. Colours represent the time from the start of each flight: initial portion of each flight is in green, changing smoothly through yellow until the end of the flight is shown in red. Grey dashed lines are used to join radar observations made more than 30 s apart, when the bee’s location was uncertain. (ZIP) [file pone.0160333.s006.zip › S113 Fig.tif]

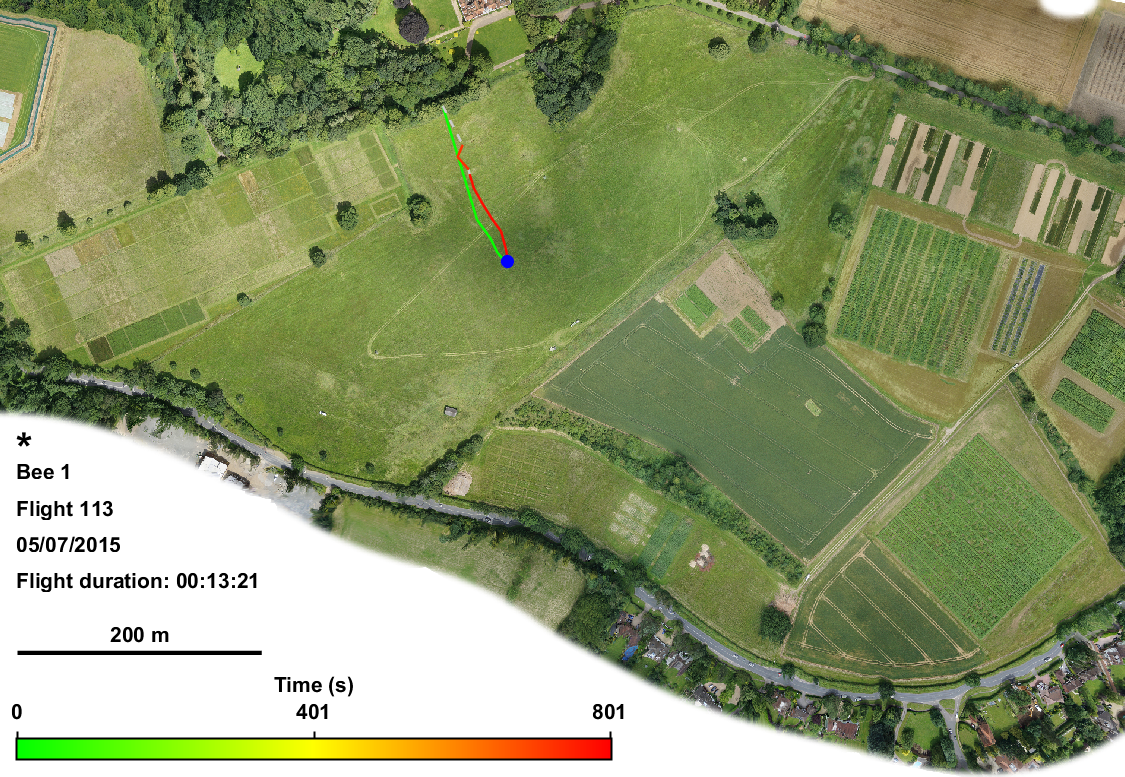

Supplement: S4 File — All flights made by Bee 1 during the period 05/07/15–06/07/15. Each figure represents a single flight. Bee ID, flight number, date of recording and the duration of each flight are shown in the bottom left corner of each figure. The position of the nest is marked by a blue circle. Colours represent the time from the start of each flight: initial portion of each flight is in green, changing smoothly through yellow until the end of the flight is shown in red. Grey dashed lines are used to join radar observations made more than 30 s apart, when the bee’s location was uncertain. (ZIP) [file pone.0160333.s006.zip › S114 Fig.tif]

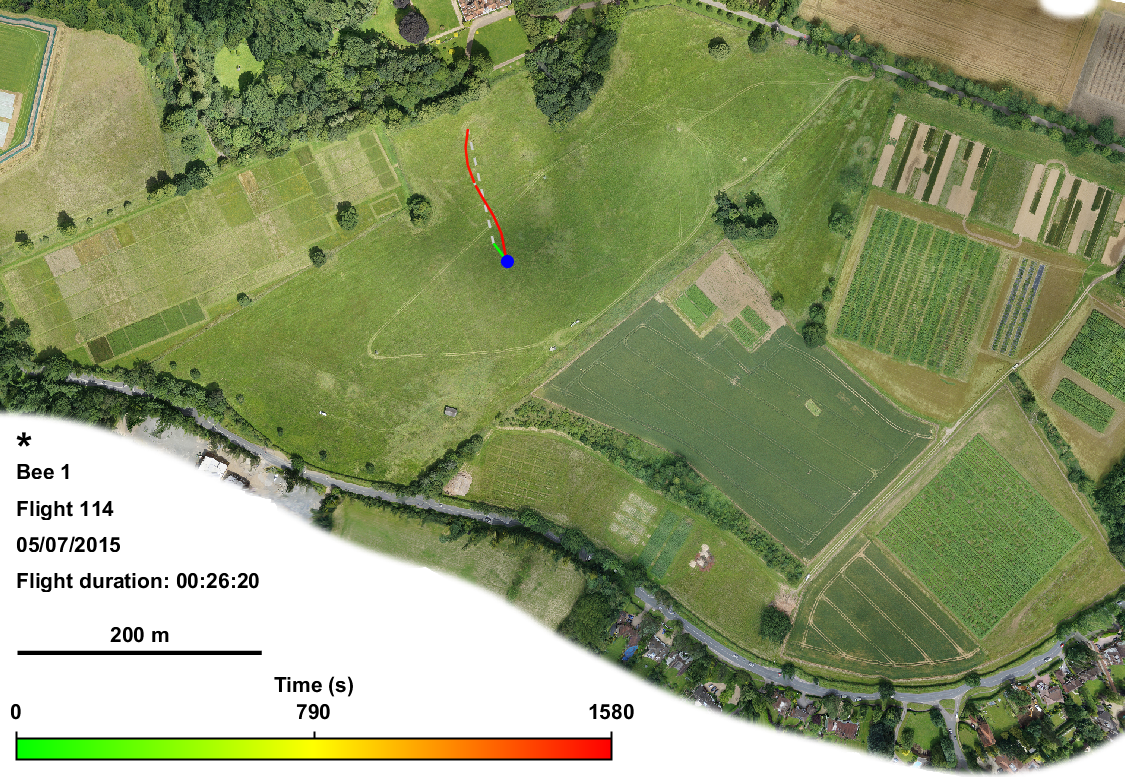

Supplement: S4 File — All flights made by Bee 1 during the period 05/07/15–06/07/15. Each figure represents a single flight. Bee ID, flight number, date of recording and the duration of each flight are shown in the bottom left corner of each figure. The position of the nest is marked by a blue circle. Colours represent the time from the start of each flight: initial portion of each flight is in green, changing smoothly through yellow until the end of the flight is shown in red. Grey dashed lines are used to join radar observations made more than 30 s apart, when the bee’s location was uncertain. (ZIP) [file pone.0160333.s006.zip › S115 Fig.tif]

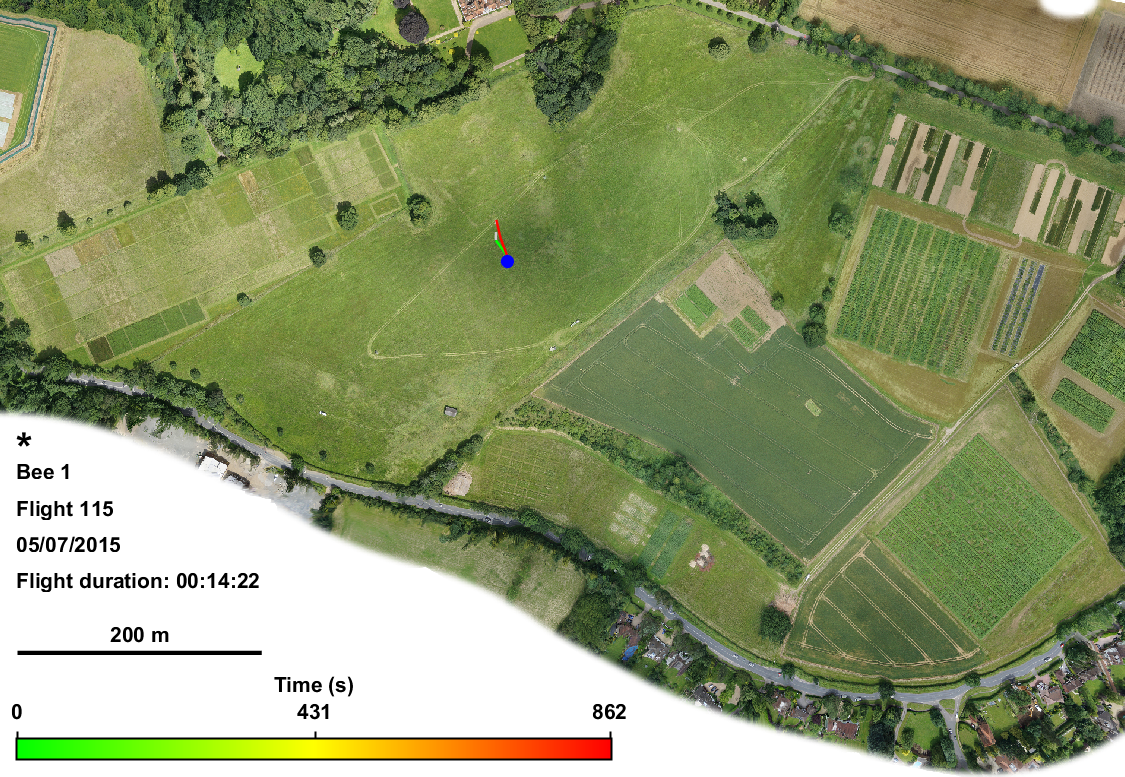

Supplement: S4 File — All flights made by Bee 1 during the period 05/07/15–06/07/15. Each figure represents a single flight. Bee ID, flight number, date of recording and the duration of each flight are shown in the bottom left corner of each figure. The position of the nest is marked by a blue circle. Colours represent the time from the start of each flight: initial portion of each flight is in green, changing smoothly through yellow until the end of the flight is shown in red. Grey dashed lines are used to join radar observations made more than 30 s apart, when the bee’s location was uncertain. (ZIP) [file pone.0160333.s006.zip › S116 Fig.tif]

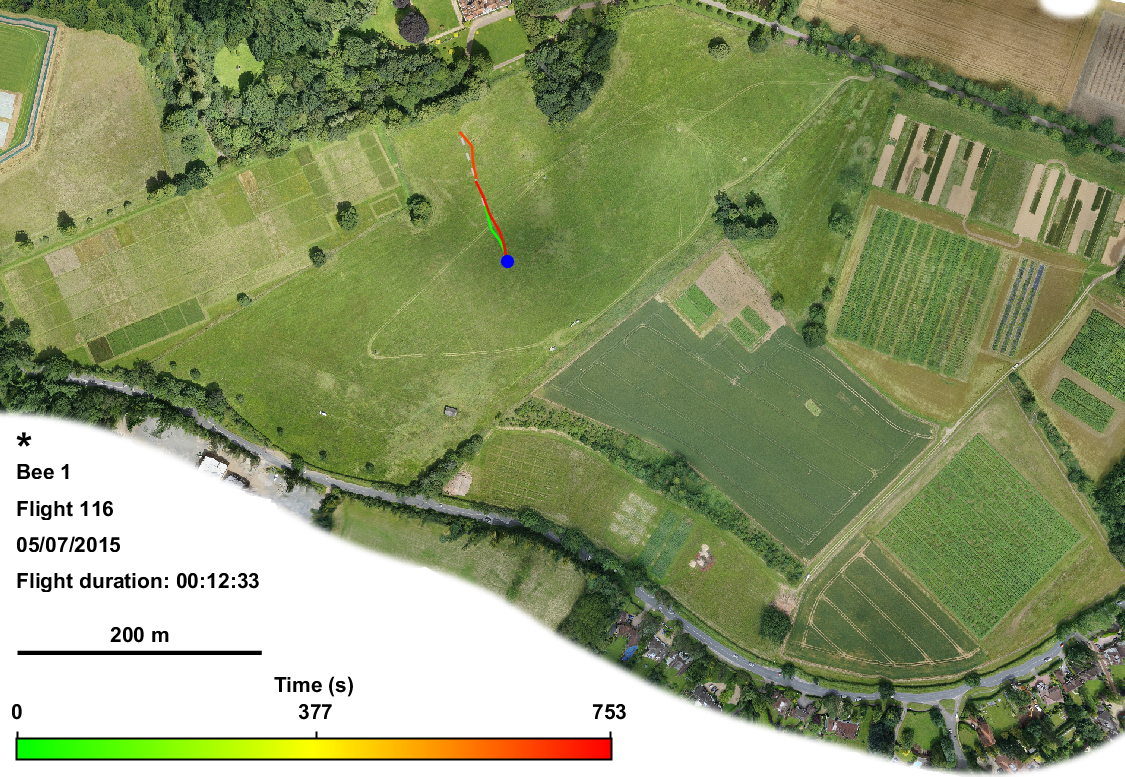

Supplement: S4 File — All flights made by Bee 1 during the period 05/07/15–06/07/15. Each figure represents a single flight. Bee ID, flight number, date of recording and the duration of each flight are shown in the bottom left corner of each figure. The position of the nest is marked by a blue circle. Colours represent the time from the start of each flight: initial portion of each flight is in green, changing smoothly through yellow until the end of the flight is shown in red. Grey dashed lines are used to join radar observations made more than 30 s apart, when the bee’s location was uncertain. (ZIP) [file pone.0160333.s006.zip › S117 Fig.tif]

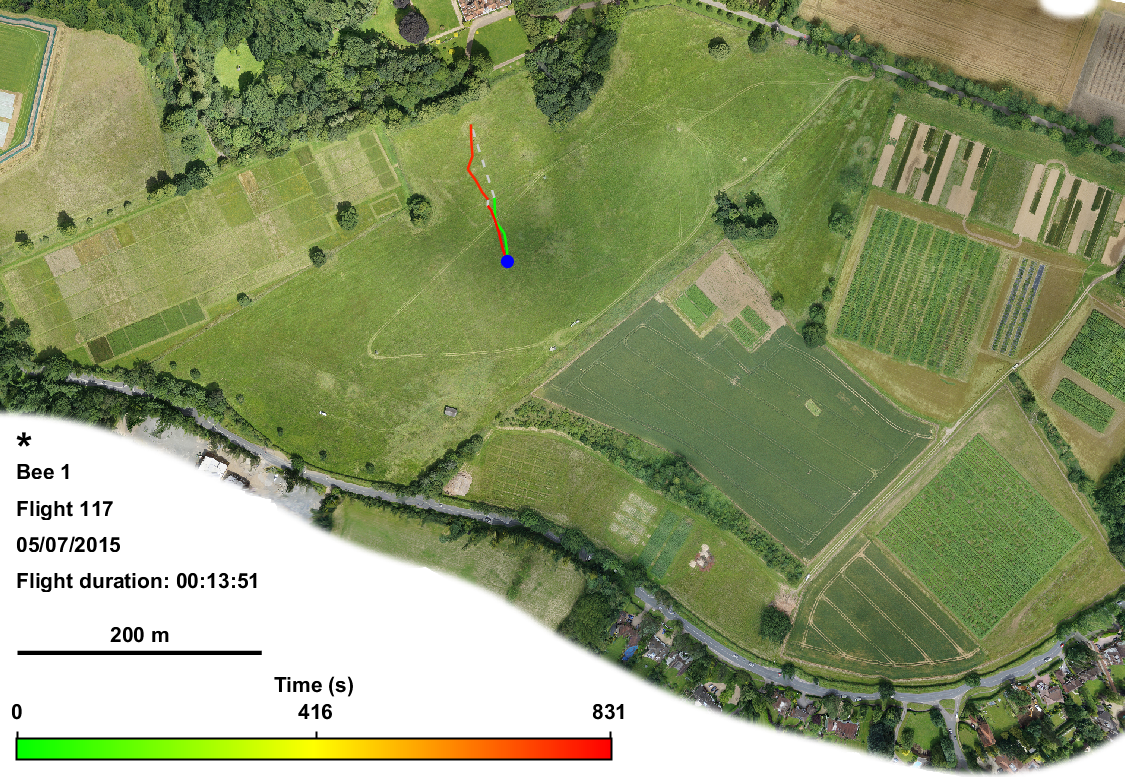

Supplement: S4 File — All flights made by Bee 1 during the period 05/07/15–06/07/15. Each figure represents a single flight. Bee ID, flight number, date of recording and the duration of each flight are shown in the bottom left corner of each figure. The position of the nest is marked by a blue circle. Colours represent the time from the start of each flight: initial portion of each flight is in green, changing smoothly through yellow until the end of the flight is shown in red. Grey dashed lines are used to join radar observations made more than 30 s apart, when the bee’s location was uncertain. (ZIP) [file pone.0160333.s006.zip › S118 Fig.tif]

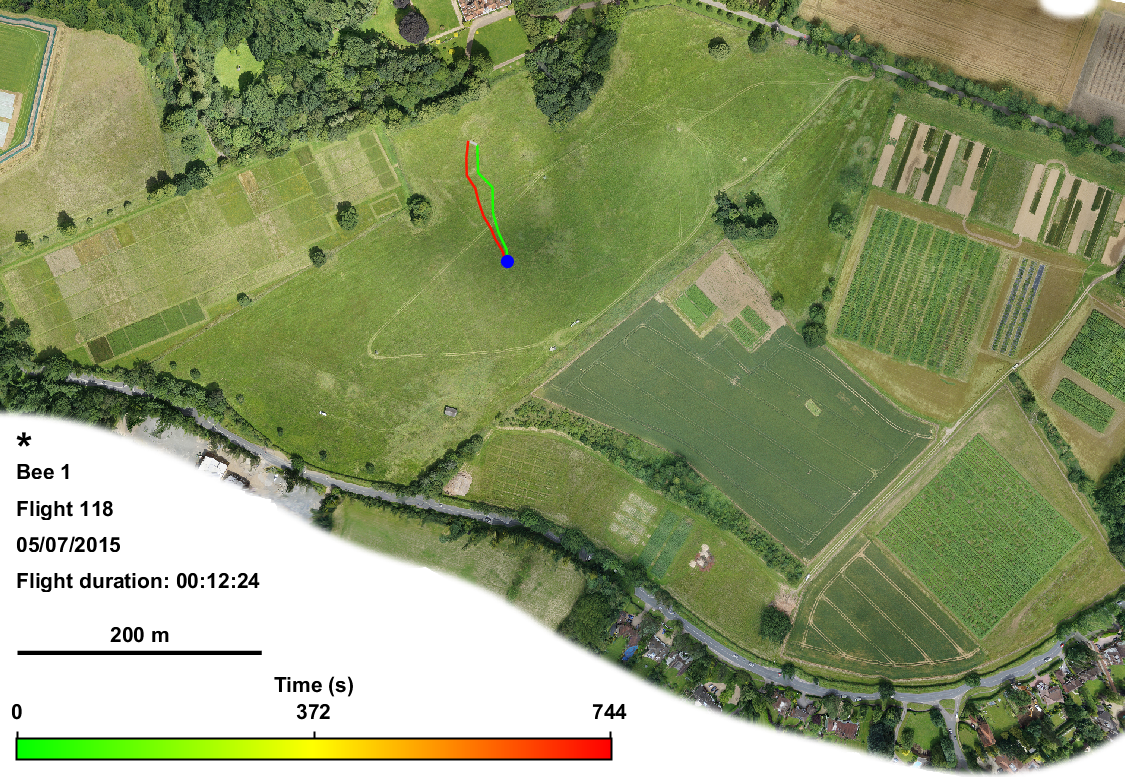

Supplement: S4 File — All flights made by Bee 1 during the period 05/07/15–06/07/15. Each figure represents a single flight. Bee ID, flight number, date of recording and the duration of each flight are shown in the bottom left corner of each figure. The position of the nest is marked by a blue circle. Colours represent the time from the start of each flight: initial portion of each flight is in green, changing smoothly through yellow until the end of the flight is shown in red. Grey dashed lines are used to join radar observations made more than 30 s apart, when the bee’s location was uncertain. (ZIP) [file pone.0160333.s006.zip › S119 Fig.tif]

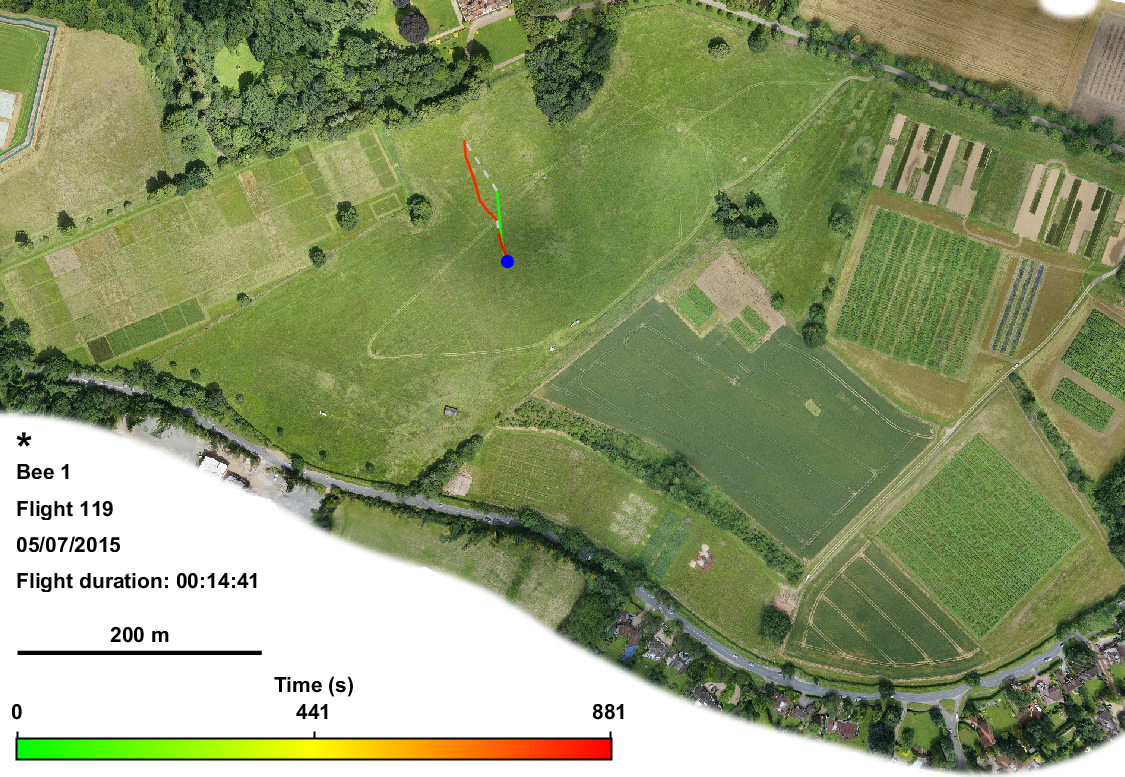

Supplement: S4 File — All flights made by Bee 1 during the period 05/07/15–06/07/15. Each figure represents a single flight. Bee ID, flight number, date of recording and the duration of each flight are shown in the bottom left corner of each figure. The position of the nest is marked by a blue circle. Colours represent the time from the start of each flight: initial portion of each flight is in green, changing smoothly through yellow until the end of the flight is shown in red. Grey dashed lines are used to join radar observations made more than 30 s apart, when the bee’s location was uncertain. (ZIP) [file pone.0160333.s006.zip › S120 Fig.tif]

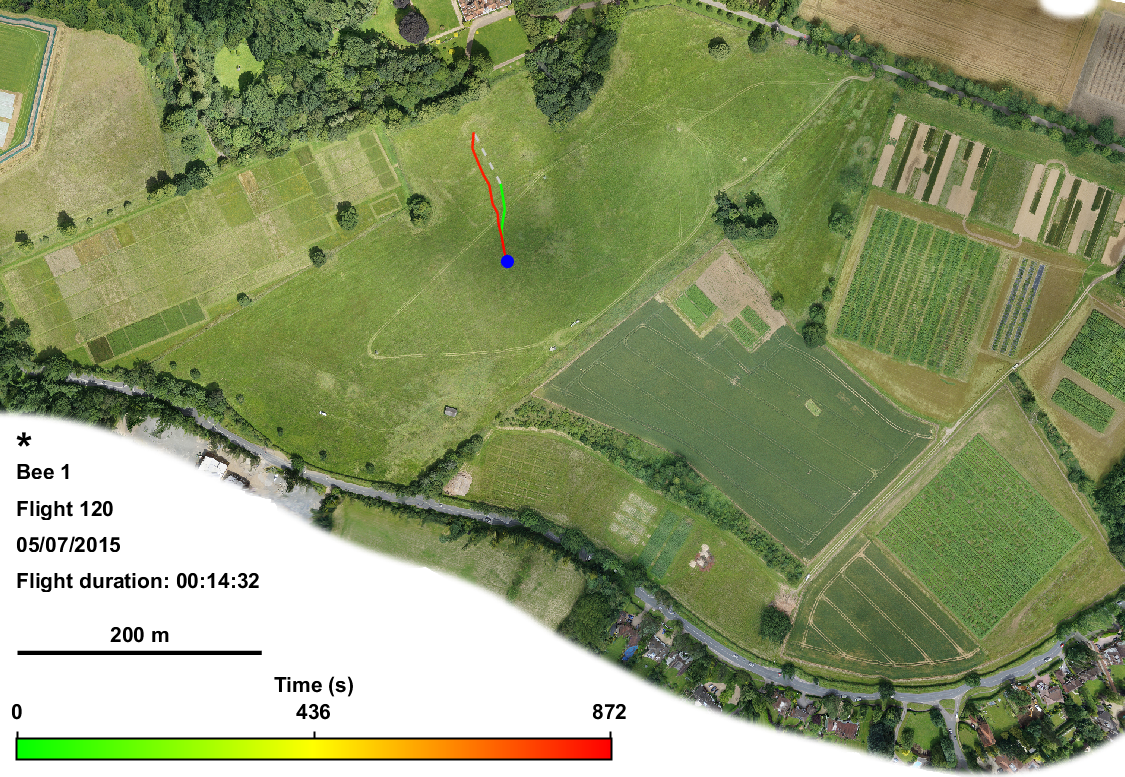

Supplement: S4 File — All flights made by Bee 1 during the period 05/07/15–06/07/15. Each figure represents a single flight. Bee ID, flight number, date of recording and the duration of each flight are shown in the bottom left corner of each figure. The position of the nest is marked by a blue circle. Colours represent the time from the start of each flight: initial portion of each flight is in green, changing smoothly through yellow until the end of the flight is shown in red. Grey dashed lines are used to join radar observations made more than 30 s apart, when the bee’s location was uncertain. (ZIP) [file pone.0160333.s006.zip › S121 Fig.tif]

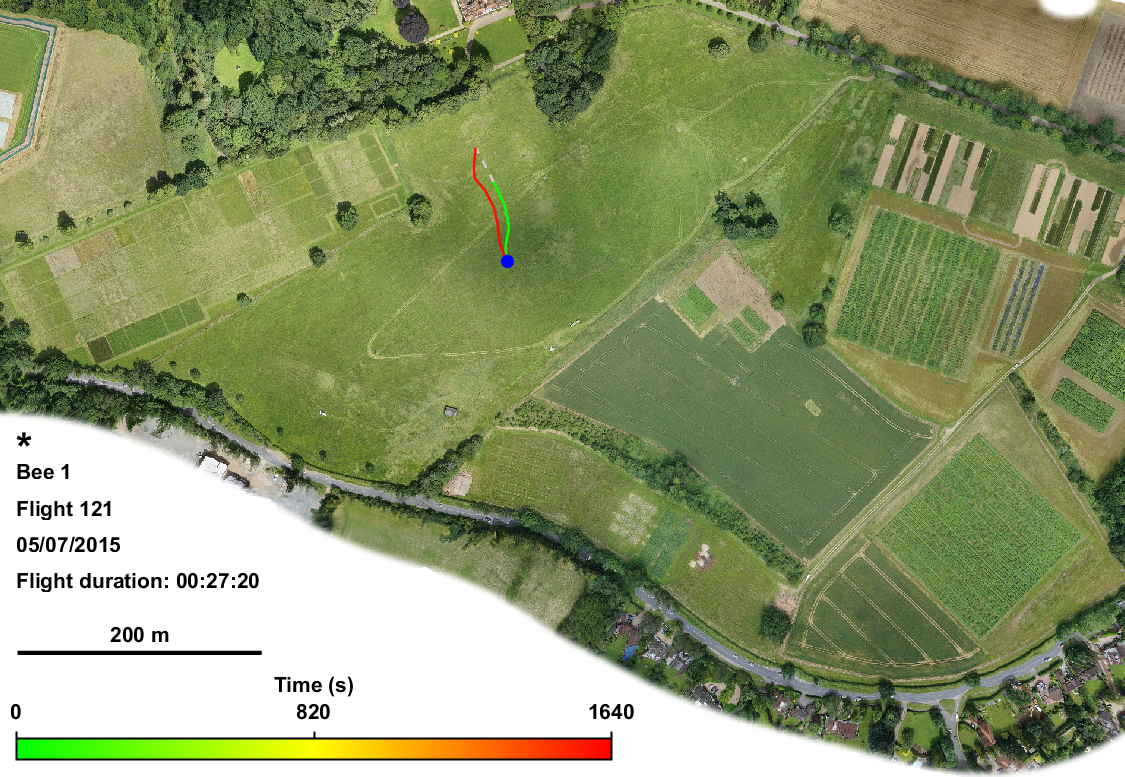

Supplement: S4 File — All flights made by Bee 1 during the period 05/07/15–06/07/15. Each figure represents a single flight. Bee ID, flight number, date of recording and the duration of each flight are shown in the bottom left corner of each figure. The position of the nest is marked by a blue circle. Colours represent the time from the start of each flight: initial portion of each flight is in green, changing smoothly through yellow until the end of the flight is shown in red. Grey dashed lines are used to join radar observations made more than 30 s apart, when the bee’s location was uncertain. (ZIP) [file pone.0160333.s006.zip › S122 Fig.tif]

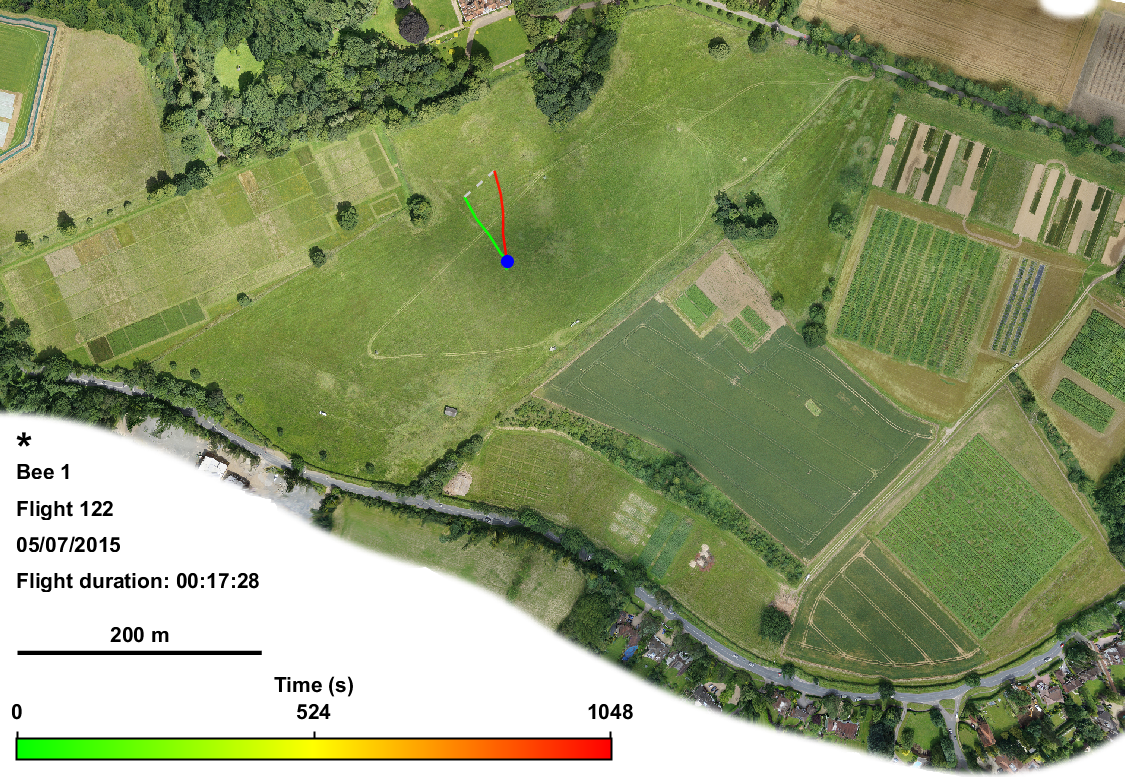

Supplement: S4 File — All flights made by Bee 1 during the period 05/07/15–06/07/15. Each figure represents a single flight. Bee ID, flight number, date of recording and the duration of each flight are shown in the bottom left corner of each figure. The position of the nest is marked by a blue circle. Colours represent the time from the start of each flight: initial portion of each flight is in green, changing smoothly through yellow until the end of the flight is shown in red. Grey dashed lines are used to join radar observations made more than 30 s apart, when the bee’s location was uncertain. (ZIP) [file pone.0160333.s006.zip › S123 Fig.tif]

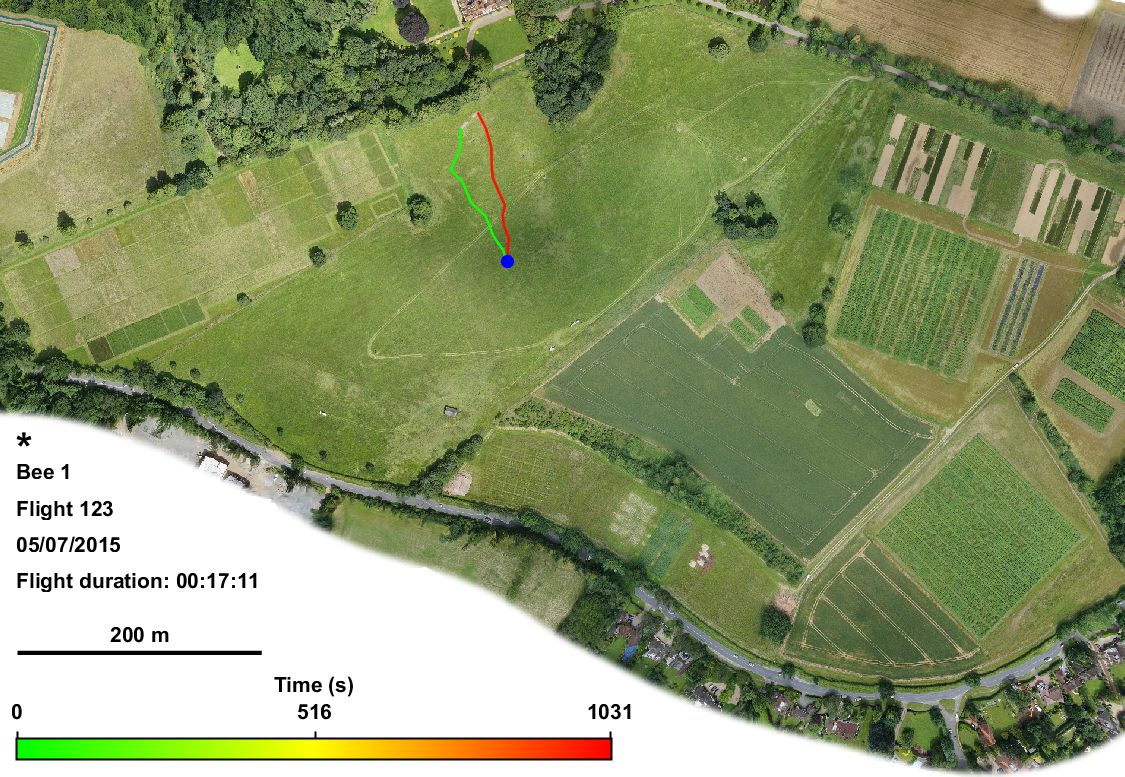

Supplement: S4 File — All flights made by Bee 1 during the period 05/07/15–06/07/15. Each figure represents a single flight. Bee ID, flight number, date of recording and the duration of each flight are shown in the bottom left corner of each figure. The position of the nest is marked by a blue circle. Colours represent the time from the start of each flight: initial portion of each flight is in green, changing smoothly through yellow until the end of the flight is shown in red. Grey dashed lines are used to join radar observations made more than 30 s apart, when the bee’s location was uncertain. (ZIP) [file pone.0160333.s006.zip › S124 Fig.tif]

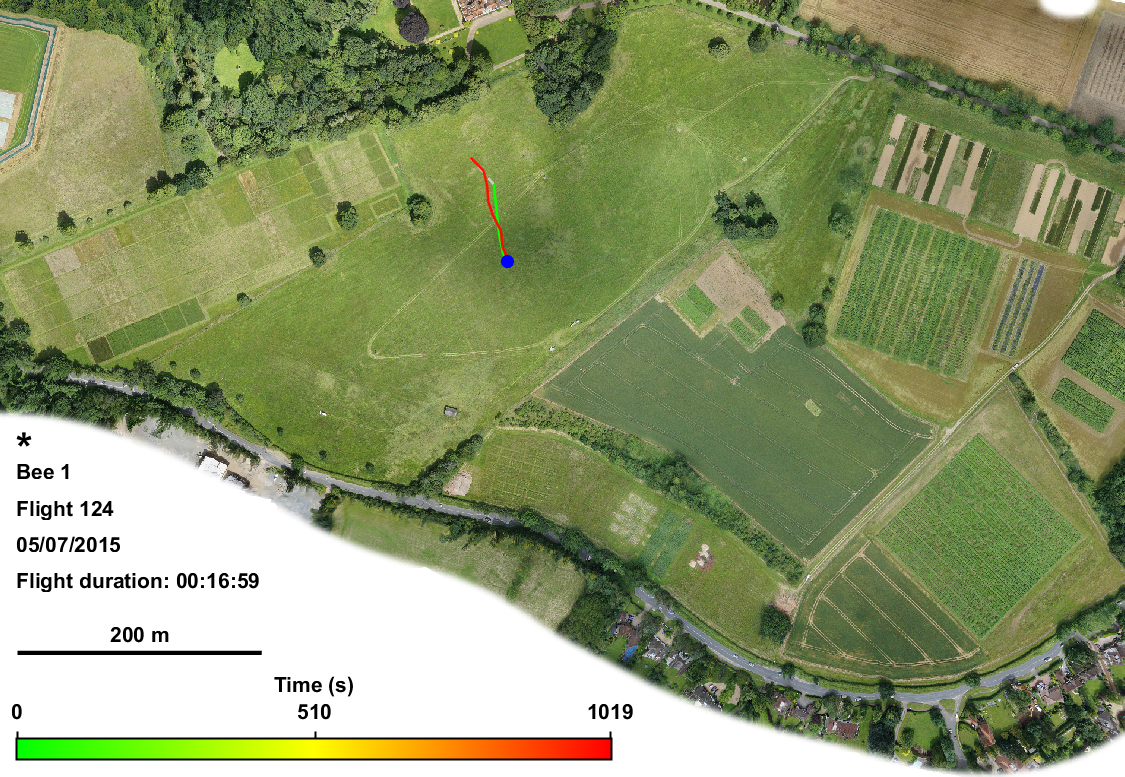

Supplement: S4 File — All flights made by Bee 1 during the period 05/07/15–06/07/15. Each figure represents a single flight. Bee ID, flight number, date of recording and the duration of each flight are shown in the bottom left corner of each figure. The position of the nest is marked by a blue circle. Colours represent the time from the start of each flight: initial portion of each flight is in green, changing smoothly through yellow until the end of the flight is shown in red. Grey dashed lines are used to join radar observations made more than 30 s apart, when the bee’s location was uncertain. (ZIP) [file pone.0160333.s006.zip › S125 Fig.tif]

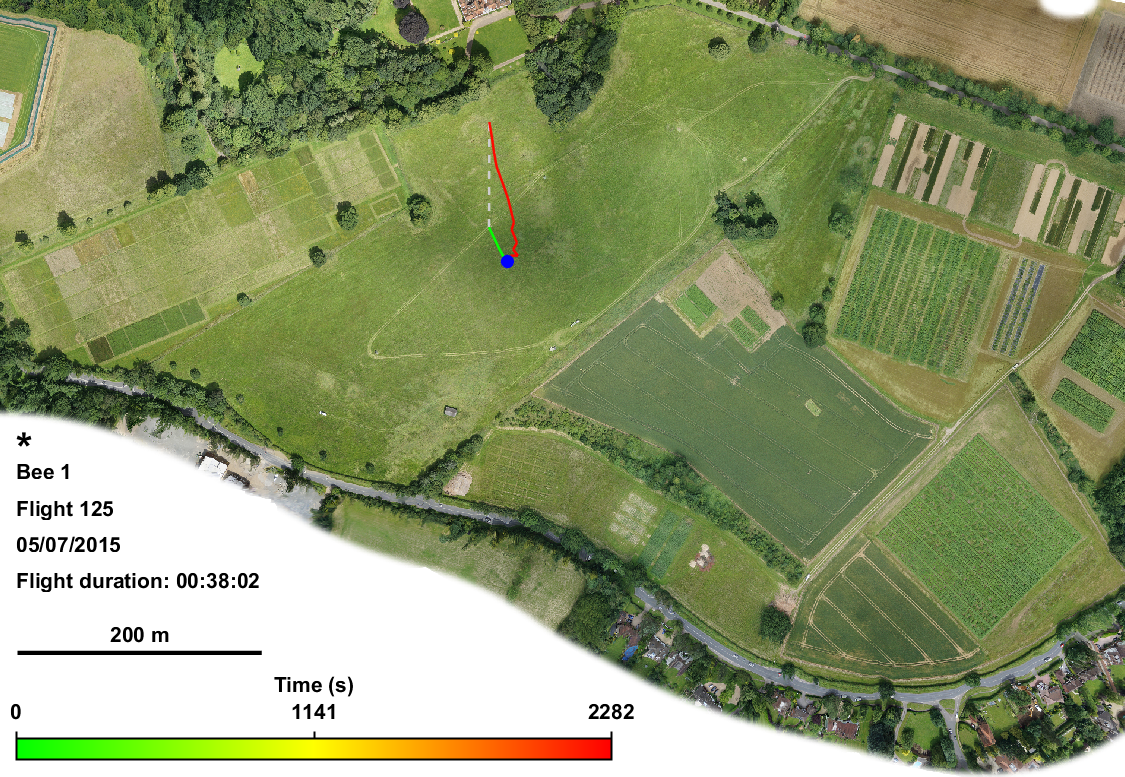

Supplement: S4 File — All flights made by Bee 1 during the period 05/07/15–06/07/15. Each figure represents a single flight. Bee ID, flight number, date of recording and the duration of each flight are shown in the bottom left corner of each figure. The position of the nest is marked by a blue circle. Colours represent the time from the start of each flight: initial portion of each flight is in green, changing smoothly through yellow until the end of the flight is shown in red. Grey dashed lines are used to join radar observations made more than 30 s apart, when the bee’s location was uncertain. (ZIP) [file pone.0160333.s006.zip › S126 Fig.tif]

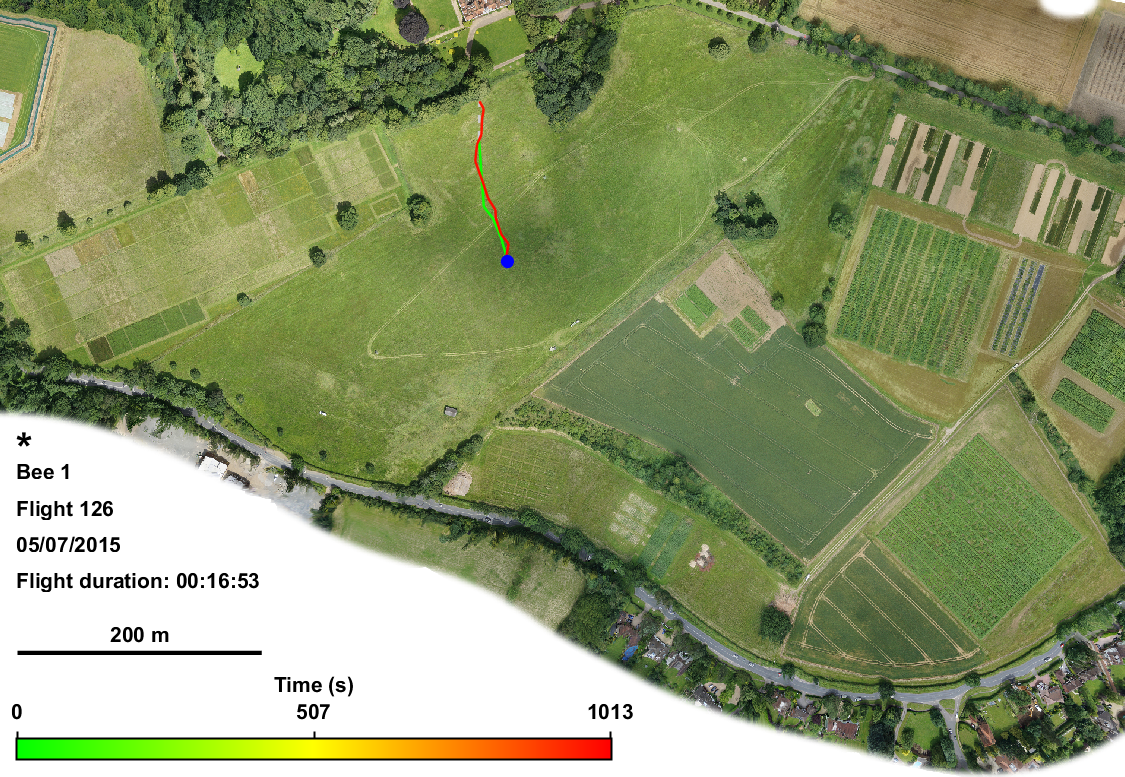

Supplement: S4 File — All flights made by Bee 1 during the period 05/07/15–06/07/15. Each figure represents a single flight. Bee ID, flight number, date of recording and the duration of each flight are shown in the bottom left corner of each figure. The position of the nest is marked by a blue circle. Colours represent the time from the start of each flight: initial portion of each flight is in green, changing smoothly through yellow until the end of the flight is shown in red. Grey dashed lines are used to join radar observations made more than 30 s apart, when the bee’s location was uncertain. (ZIP) [file pone.0160333.s006.zip › S127 Fig.tif]

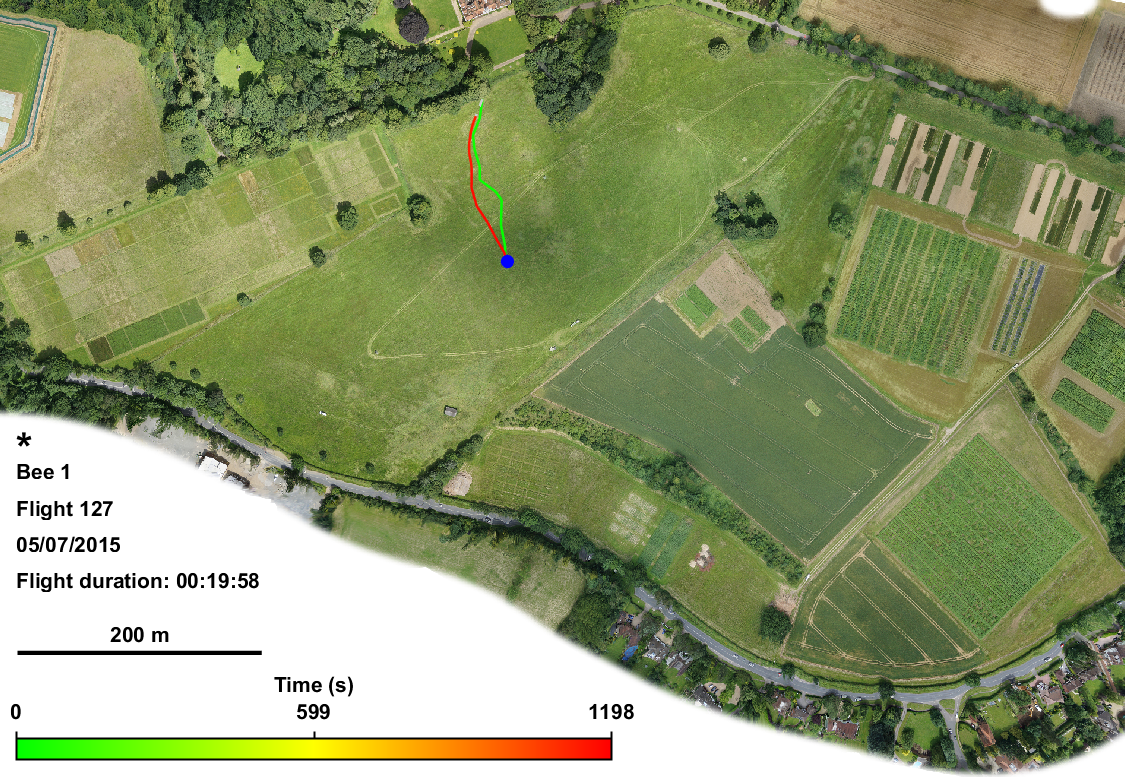

Supplement: S4 File — All flights made by Bee 1 during the period 05/07/15–06/07/15. Each figure represents a single flight. Bee ID, flight number, date of recording and the duration of each flight are shown in the bottom left corner of each figure. The position of the nest is marked by a blue circle. Colours represent the time from the start of each flight: initial portion of each flight is in green, changing smoothly through yellow until the end of the flight is shown in red. Grey dashed lines are used to join radar observations made more than 30 s apart, when the bee’s location was uncertain. (ZIP) [file pone.0160333.s006.zip › S128 Fig.tif]

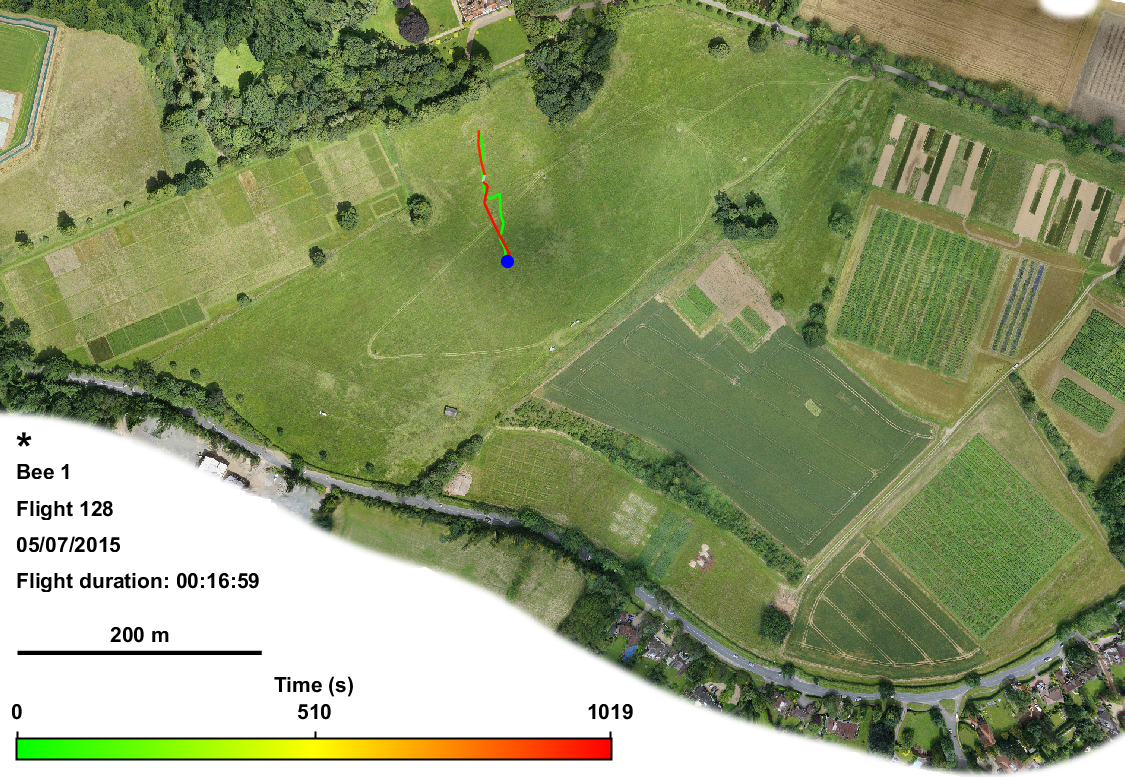

Supplement: S4 File — All flights made by Bee 1 during the period 05/07/15–06/07/15. Each figure represents a single flight. Bee ID, flight number, date of recording and the duration of each flight are shown in the bottom left corner of each figure. The position of the nest is marked by a blue circle. Colours represent the time from the start of each flight: initial portion of each flight is in green, changing smoothly through yellow until the end of the flight is shown in red. Grey dashed lines are used to join radar observations made more than 30 s apart, when the bee’s location was uncertain. (ZIP) [file pone.0160333.s006.zip › S129 Fig.tif]

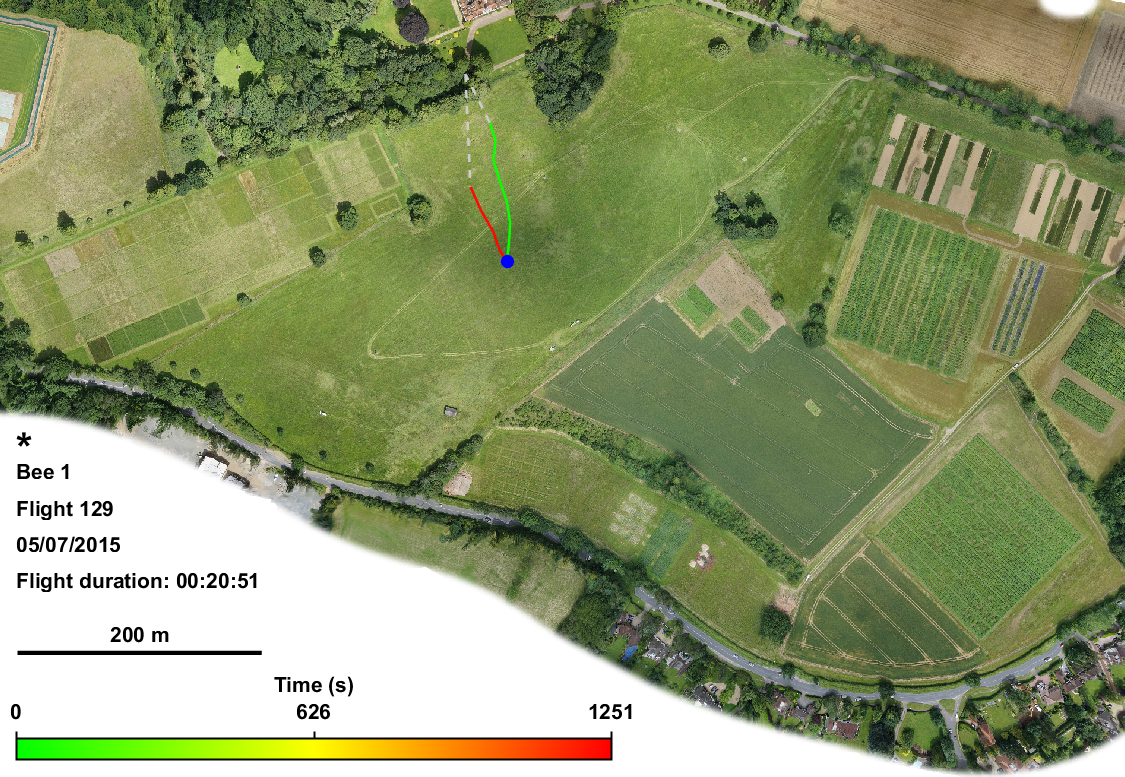

Supplement: S4 File — All flights made by Bee 1 during the period 05/07/15–06/07/15. Each figure represents a single flight. Bee ID, flight number, date of recording and the duration of each flight are shown in the bottom left corner of each figure. The position of the nest is marked by a blue circle. Colours represent the time from the start of each flight: initial portion of each flight is in green, changing smoothly through yellow until the end of the flight is shown in red. Grey dashed lines are used to join radar observations made more than 30 s apart, when the bee’s location was uncertain. (ZIP) [file pone.0160333.s006.zip › S130 Fig.tif]

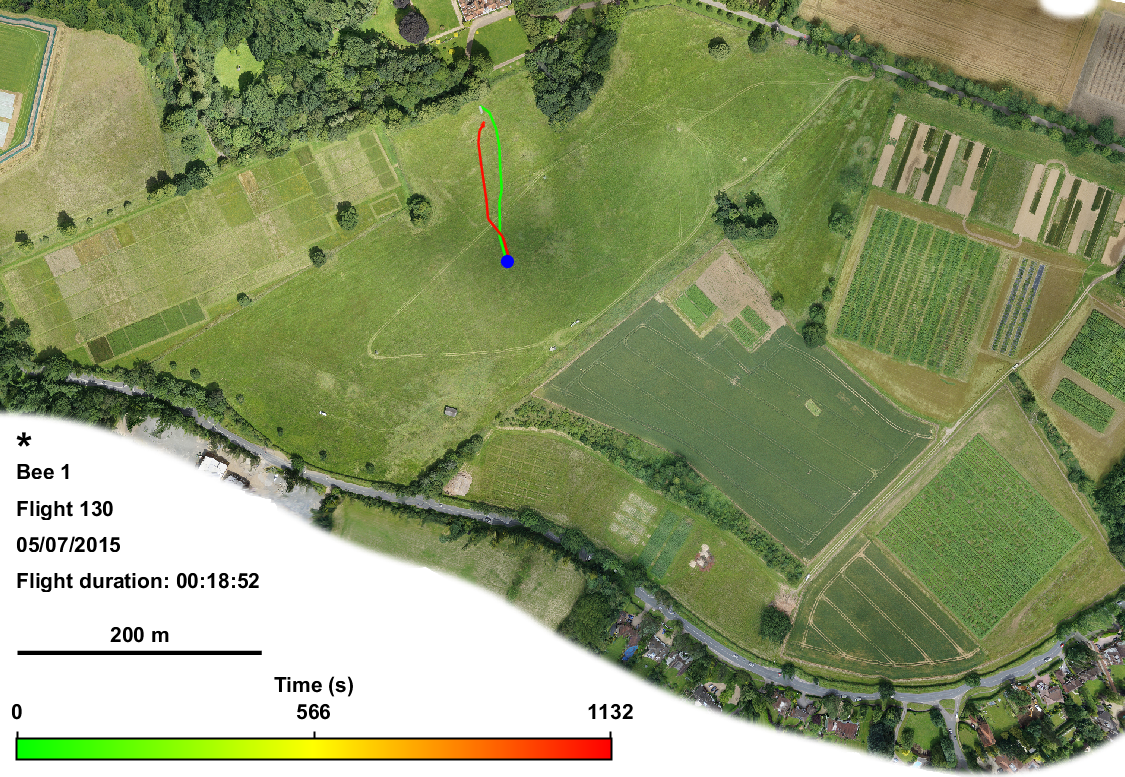

Supplement: S4 File — All flights made by Bee 1 during the period 05/07/15–06/07/15. Each figure represents a single flight. Bee ID, flight number, date of recording and the duration of each flight are shown in the bottom left corner of each figure. The position of the nest is marked by a blue circle. Colours represent the time from the start of each flight: initial portion of each flight is in green, changing smoothly through yellow until the end of the flight is shown in red. Grey dashed lines are used to join radar observations made more than 30 s apart, when the bee’s location was uncertain. (ZIP) [file pone.0160333.s006.zip › S131 Fig.tif]

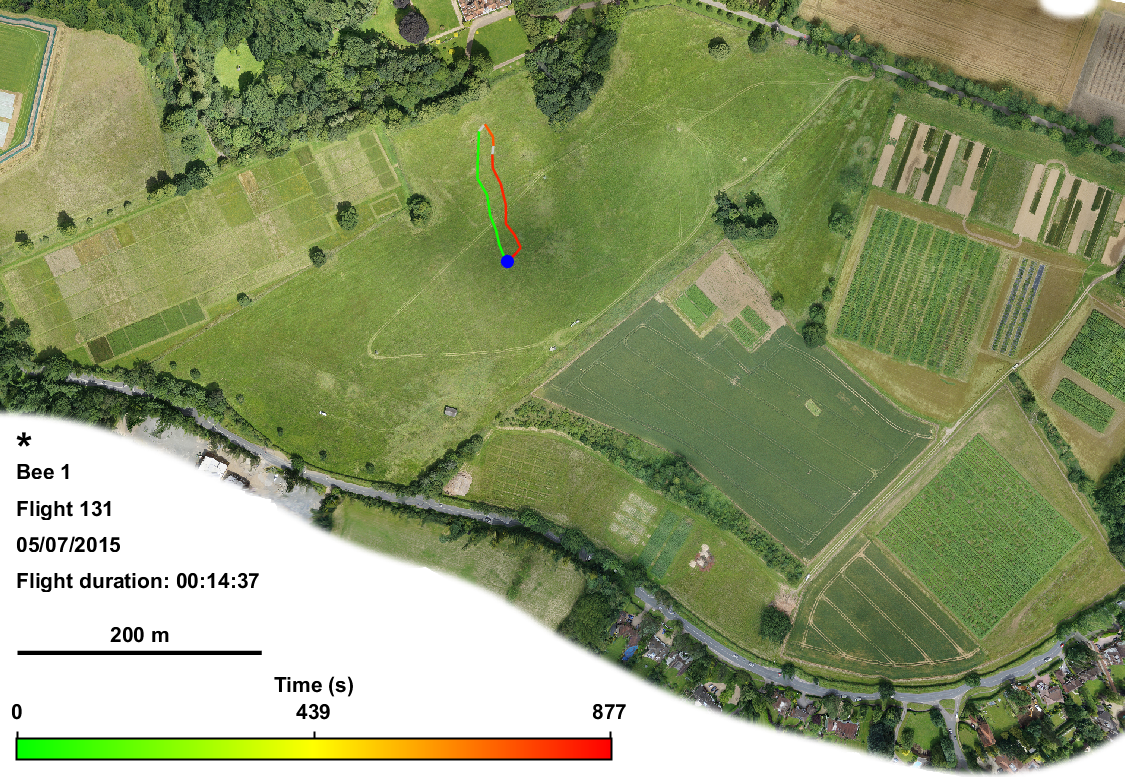

Supplement: S4 File — All flights made by Bee 1 during the period 05/07/15–06/07/15. Each figure represents a single flight. Bee ID, flight number, date of recording and the duration of each flight are shown in the bottom left corner of each figure. The position of the nest is marked by a blue circle. Colours represent the time from the start of each flight: initial portion of each flight is in green, changing smoothly through yellow until the end of the flight is shown in red. Grey dashed lines are used to join radar observations made more than 30 s apart, when the bee’s location was uncertain. (ZIP) [file pone.0160333.s006.zip › S132 Fig.tif]

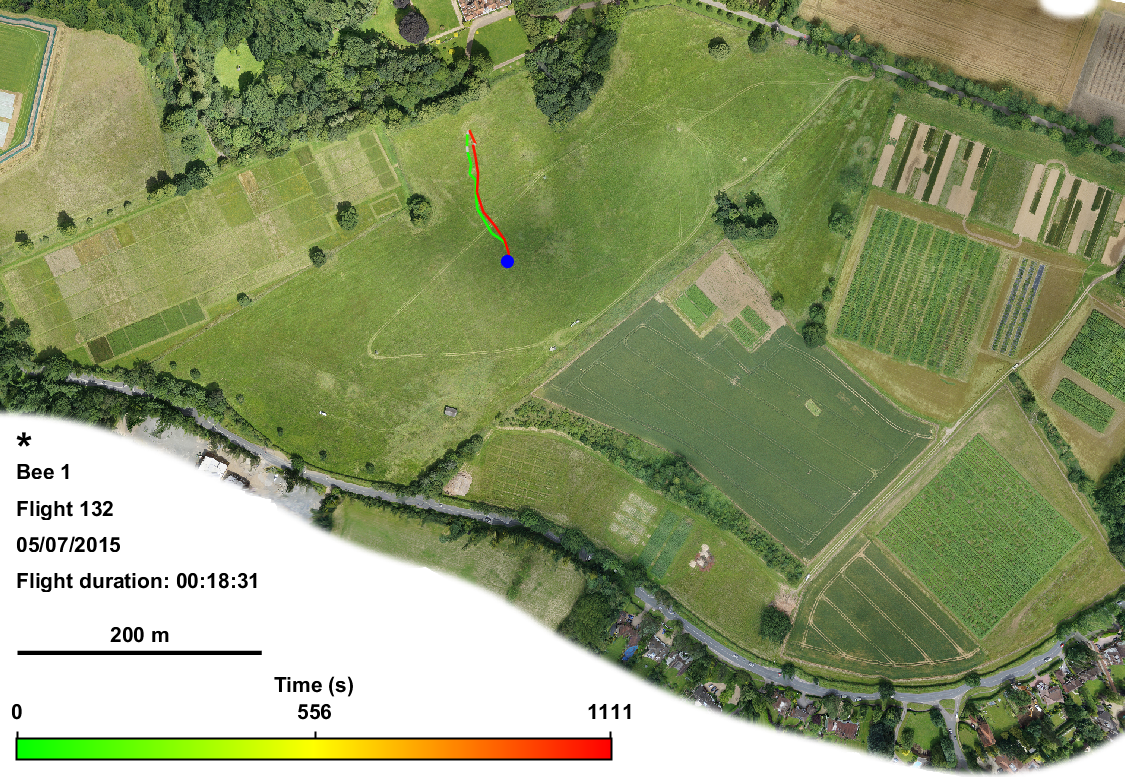

Supplement: S4 File — All flights made by Bee 1 during the period 05/07/15–06/07/15. Each figure represents a single flight. Bee ID, flight number, date of recording and the duration of each flight are shown in the bottom left corner of each figure. The position of the nest is marked by a blue circle. Colours represent the time from the start of each flight: initial portion of each flight is in green, changing smoothly through yellow until the end of the flight is shown in red. Grey dashed lines are used to join radar observations made more than 30 s apart, when the bee’s location was uncertain. (ZIP) [file pone.0160333.s006.zip › S133 Fig.tif]

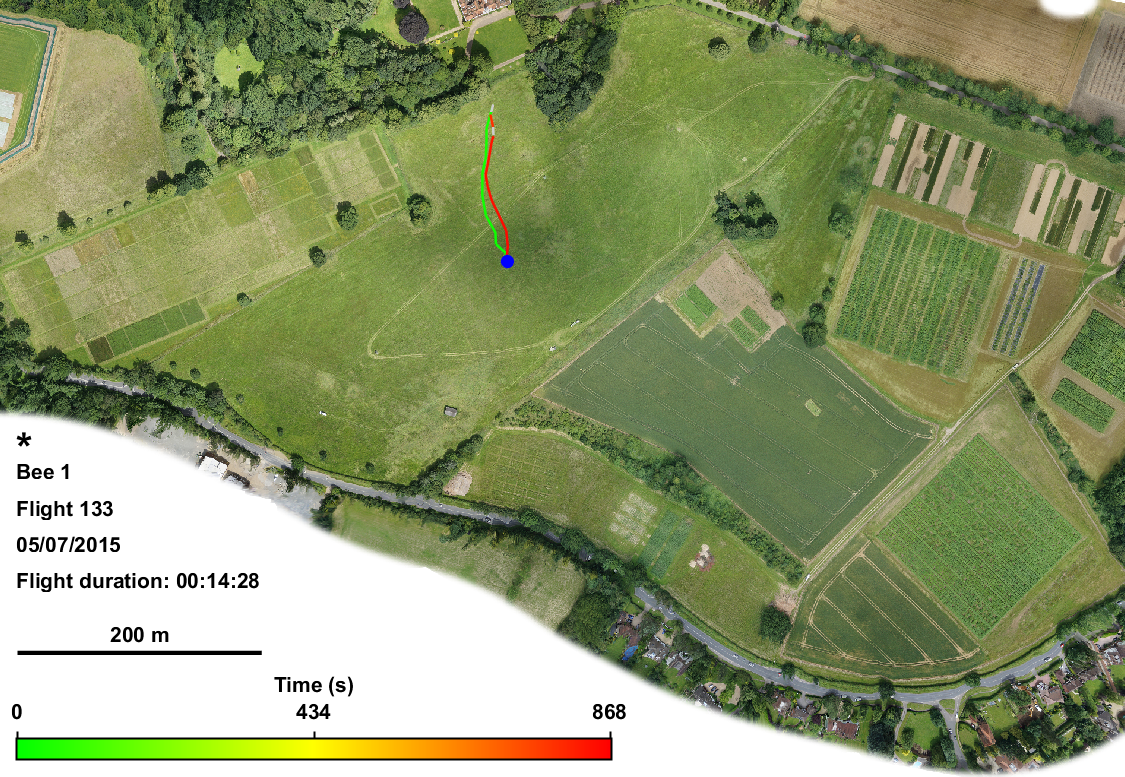

Supplement: S4 File — All flights made by Bee 1 during the period 05/07/15–06/07/15. Each figure represents a single flight. Bee ID, flight number, date of recording and the duration of each flight are shown in the bottom left corner of each figure. The position of the nest is marked by a blue circle. Colours represent the time from the start of each flight: initial portion of each flight is in green, changing smoothly through yellow until the end of the flight is shown in red. Grey dashed lines are used to join radar observations made more than 30 s apart, when the bee’s location was uncertain. (ZIP) [file pone.0160333.s006.zip › S134 Fig.tif]

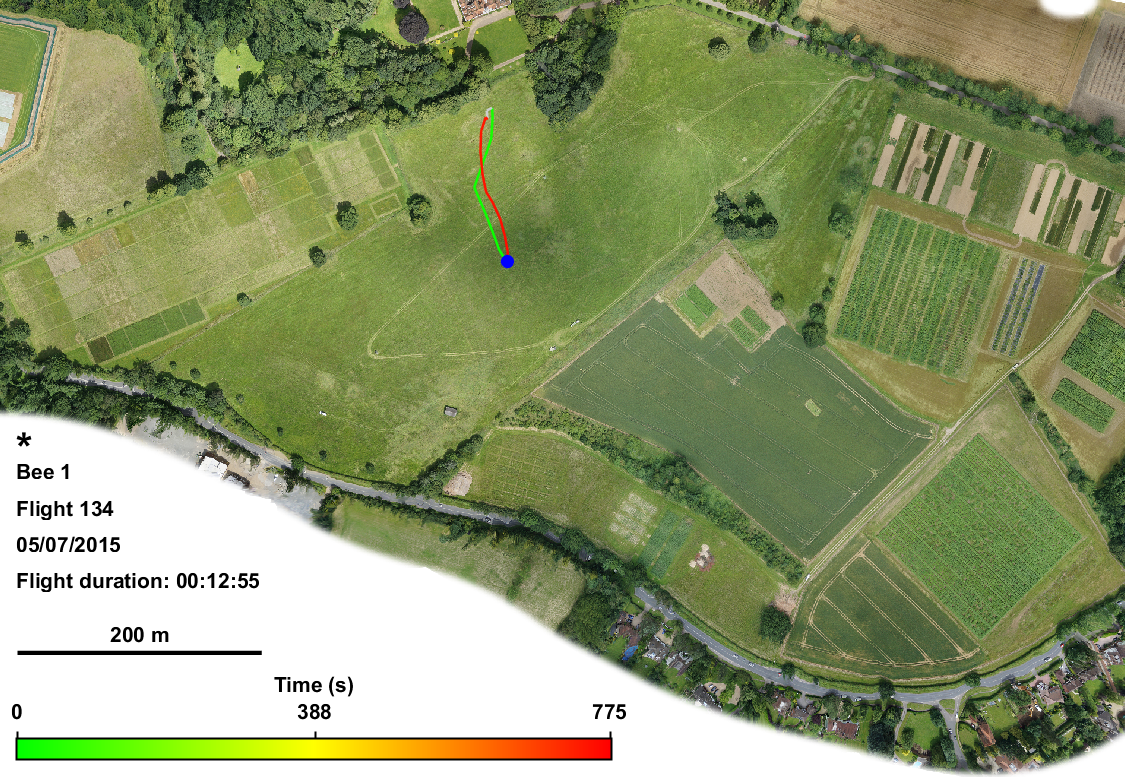

Supplement: S4 File — All flights made by Bee 1 during the period 05/07/15–06/07/15. Each figure represents a single flight. Bee ID, flight number, date of recording and the duration of each flight are shown in the bottom left corner of each figure. The position of the nest is marked by a blue circle. Colours represent the time from the start of each flight: initial portion of each flight is in green, changing smoothly through yellow until the end of the flight is shown in red. Grey dashed lines are used to join radar observations made more than 30 s apart, when the bee’s location was uncertain. (ZIP) [file pone.0160333.s006.zip › S135 Fig.tif]

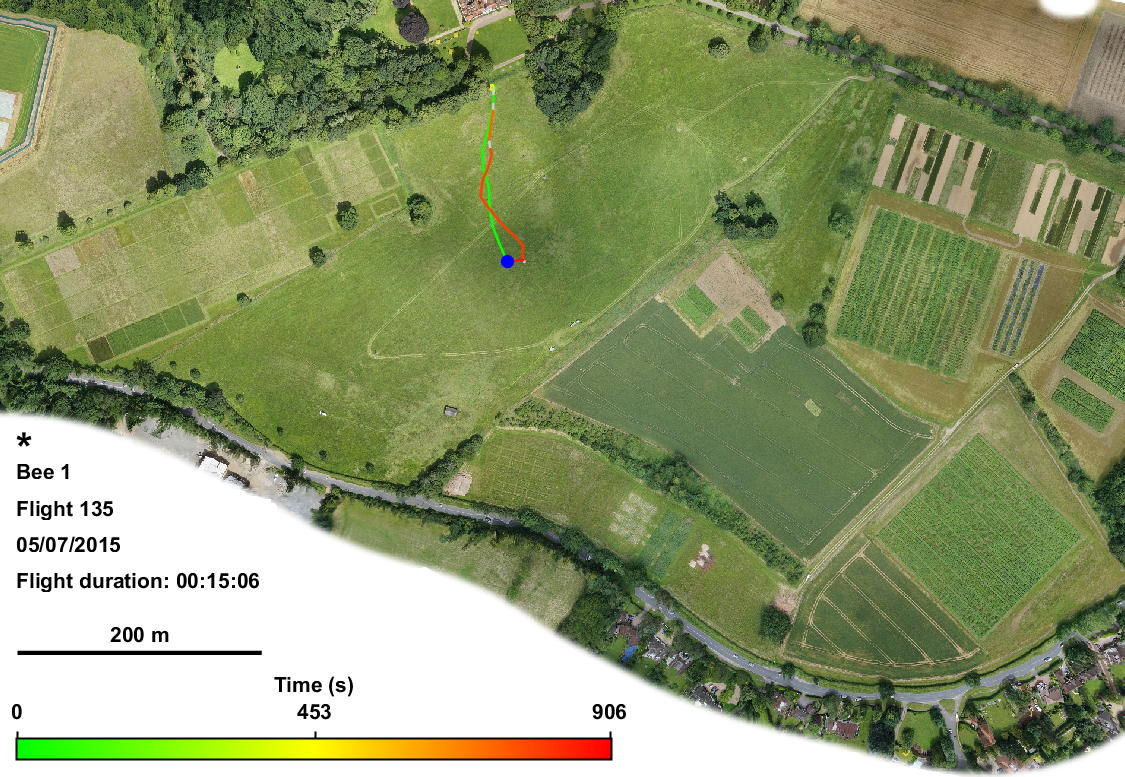

Supplement: S4 File — All flights made by Bee 1 during the period 05/07/15–06/07/15. Each figure represents a single flight. Bee ID, flight number, date of recording and the duration of each flight are shown in the bottom left corner of each figure. The position of the nest is marked by a blue circle. Colours represent the time from the start of each flight: initial portion of each flight is in green, changing smoothly through yellow until the end of the flight is shown in red. Grey dashed lines are used to join radar observations made more than 30 s apart, when the bee’s location was uncertain. (ZIP) [file pone.0160333.s006.zip › S136 Fig.tif]

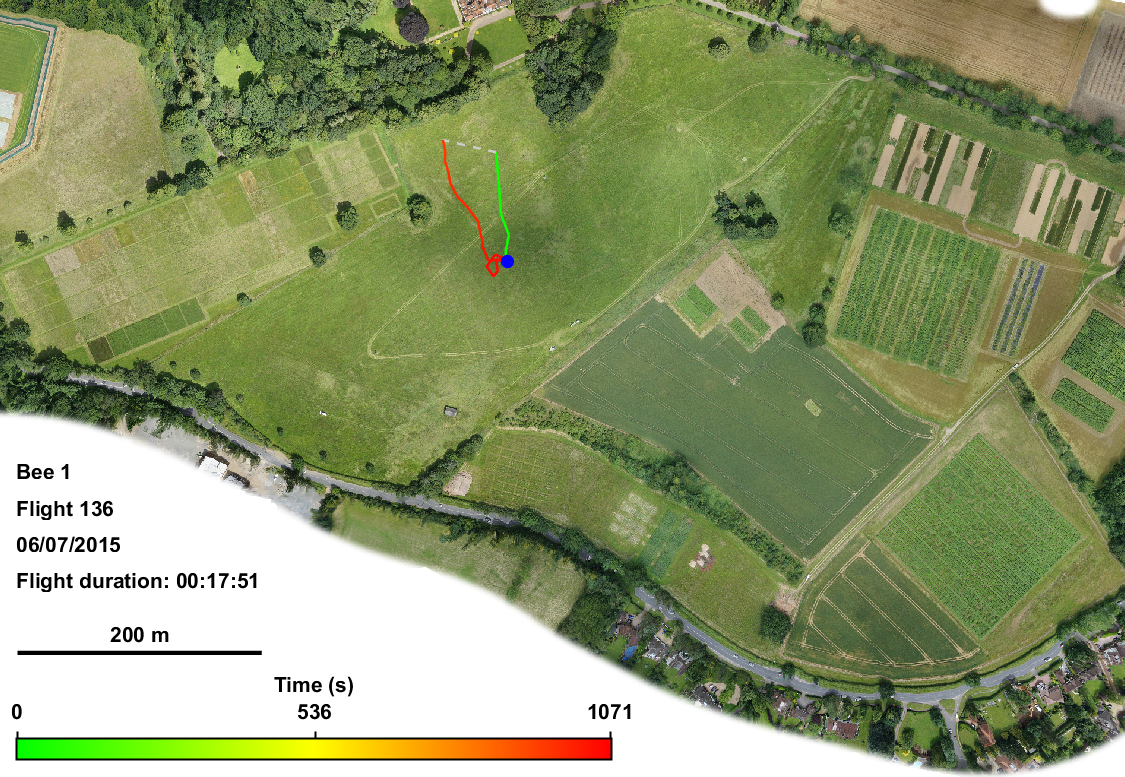

Supplement: S4 File — All flights made by Bee 1 during the period 05/07/15–06/07/15. Each figure represents a single flight. Bee ID, flight number, date of recording and the duration of each flight are shown in the bottom left corner of each figure. The position of the nest is marked by a blue circle. Colours represent the time from the start of each flight: initial portion of each flight is in green, changing smoothly through yellow until the end of the flight is shown in red. Grey dashed lines are used to join radar observations made more than 30 s apart, when the bee’s location was uncertain. (ZIP) [file pone.0160333.s006.zip › S137 Fig.tif]

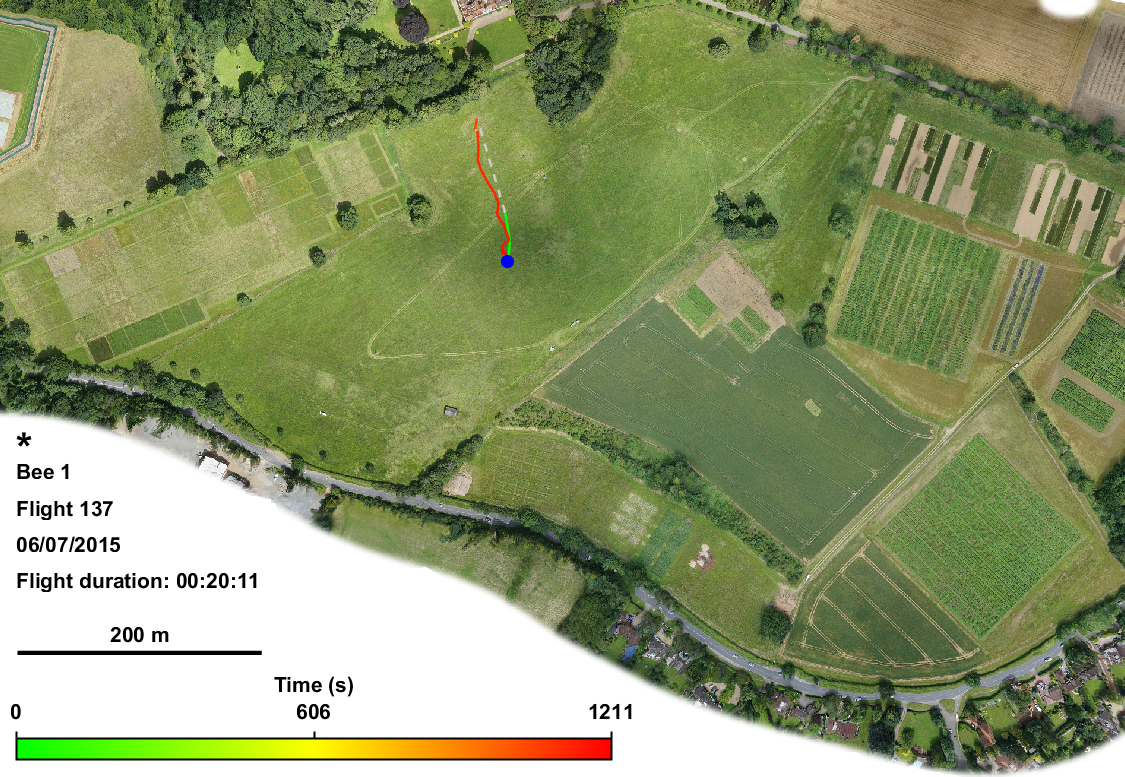

Supplement: S4 File — All flights made by Bee 1 during the period 05/07/15–06/07/15. Each figure represents a single flight. Bee ID, flight number, date of recording and the duration of each flight are shown in the bottom left corner of each figure. The position of the nest is marked by a blue circle. Colours represent the time from the start of each flight: initial portion of each flight is in green, changing smoothly through yellow until the end of the flight is shown in red. Grey dashed lines are used to join radar observations made more than 30 s apart, when the bee’s location was uncertain. (ZIP) [file pone.0160333.s006.zip › S138 Fig.tif]

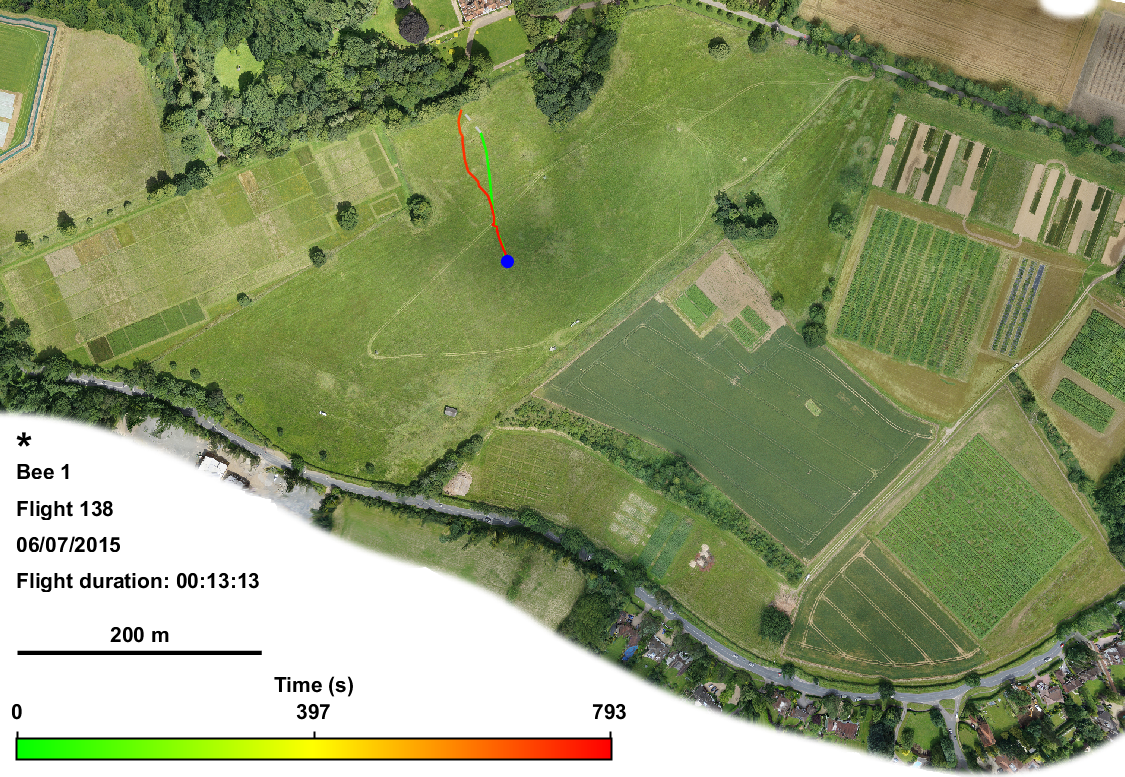

Supplement: S4 File — All flights made by Bee 1 during the period 05/07/15–06/07/15. Each figure represents a single flight. Bee ID, flight number, date of recording and the duration of each flight are shown in the bottom left corner of each figure. The position of the nest is marked by a blue circle. Colours represent the time from the start of each flight: initial portion of each flight is in green, changing smoothly through yellow until the end of the flight is shown in red. Grey dashed lines are used to join radar observations made more than 30 s apart, when the bee’s location was uncertain. (ZIP) [file pone.0160333.s006.zip › S139 Fig.tif]

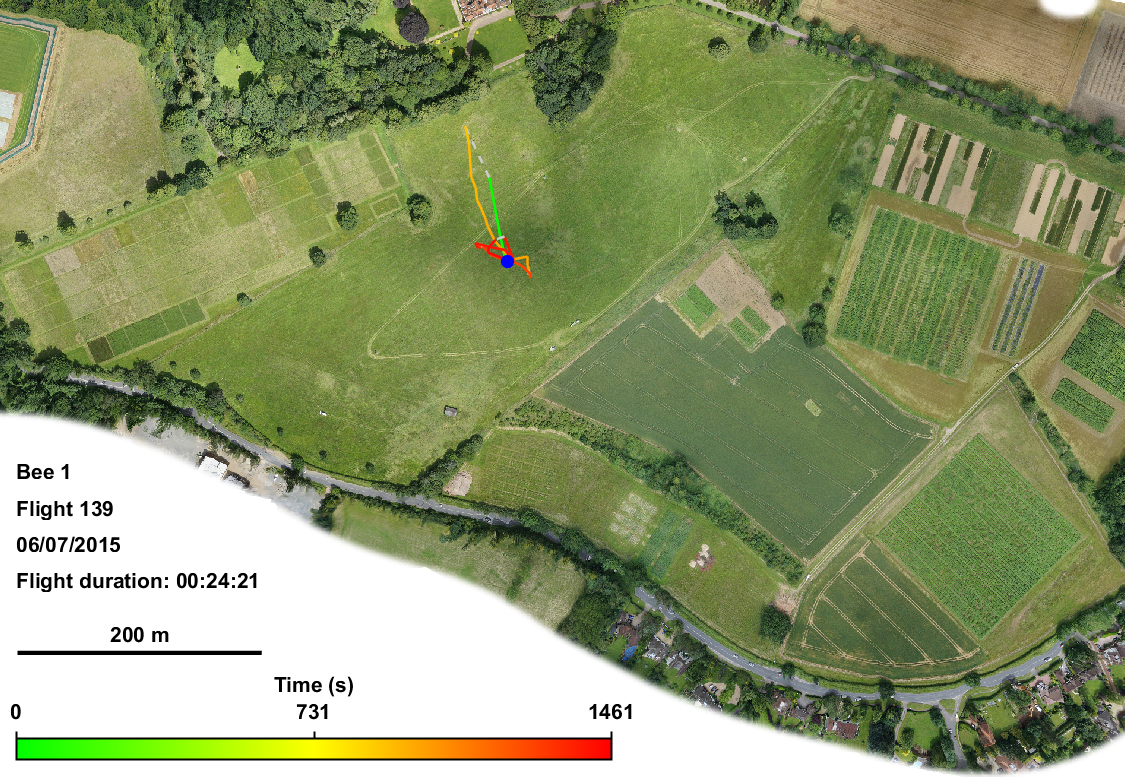

Supplement: S4 File — All flights made by Bee 1 during the period 05/07/15–06/07/15. Each figure represents a single flight. Bee ID, flight number, date of recording and the duration of each flight are shown in the bottom left corner of each figure. The position of the nest is marked by a blue circle. Colours represent the time from the start of each flight: initial portion of each flight is in green, changing smoothly through yellow until the end of the flight is shown in red. Grey dashed lines are used to join radar observations made more than 30 s apart, when the bee’s location was uncertain. (ZIP) [file pone.0160333.s006.zip › S140 Fig.tif]

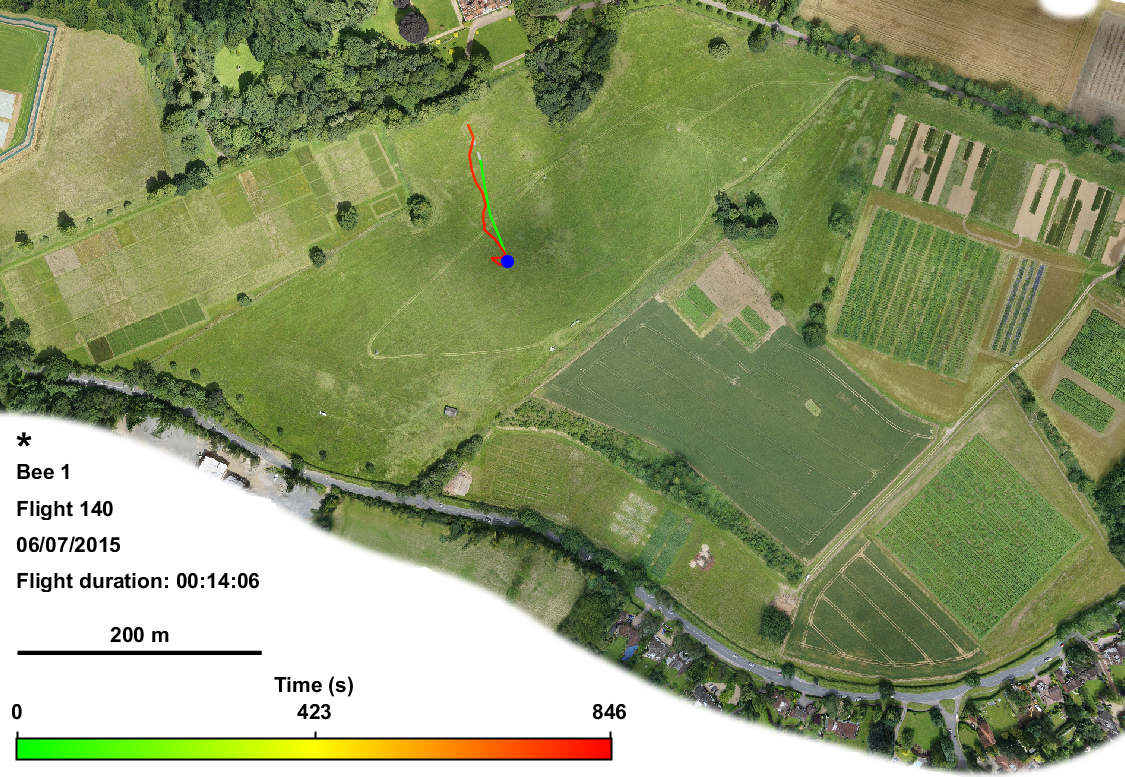

Supplement: S4 File — All flights made by Bee 1 during the period 05/07/15–06/07/15. Each figure represents a single flight. Bee ID, flight number, date of recording and the duration of each flight are shown in the bottom left corner of each figure. The position of the nest is marked by a blue circle. Colours represent the time from the start of each flight: initial portion of each flight is in green, changing smoothly through yellow until the end of the flight is shown in red. Grey dashed lines are used to join radar observations made more than 30 s apart, when the bee’s location was uncertain. (ZIP) [file pone.0160333.s006.zip › S141 Fig.tif]

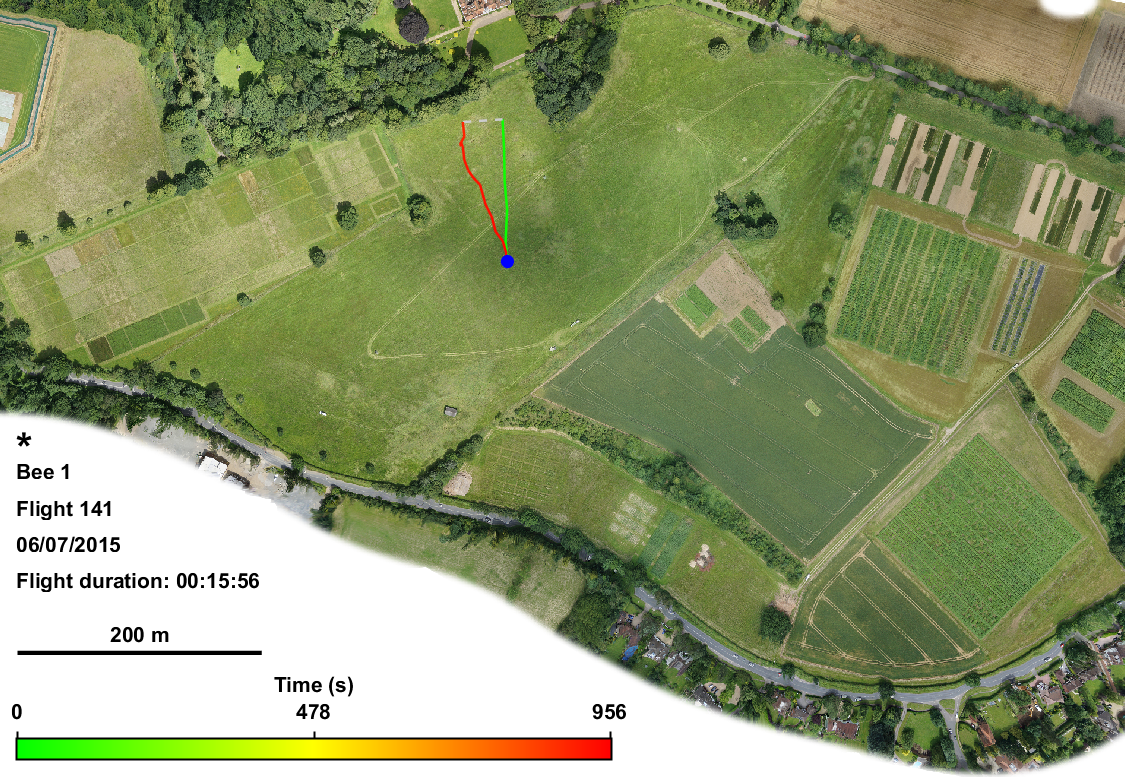

Supplement: S4 File — All flights made by Bee 1 during the period 05/07/15–06/07/15. Each figure represents a single flight. Bee ID, flight number, date of recording and the duration of each flight are shown in the bottom left corner of each figure. The position of the nest is marked by a blue circle. Colours represent the time from the start of each flight: initial portion of each flight is in green, changing smoothly through yellow until the end of the flight is shown in red. Grey dashed lines are used to join radar observations made more than 30 s apart, when the bee’s location was uncertain. (ZIP) [file pone.0160333.s006.zip › S142 Fig.tif]

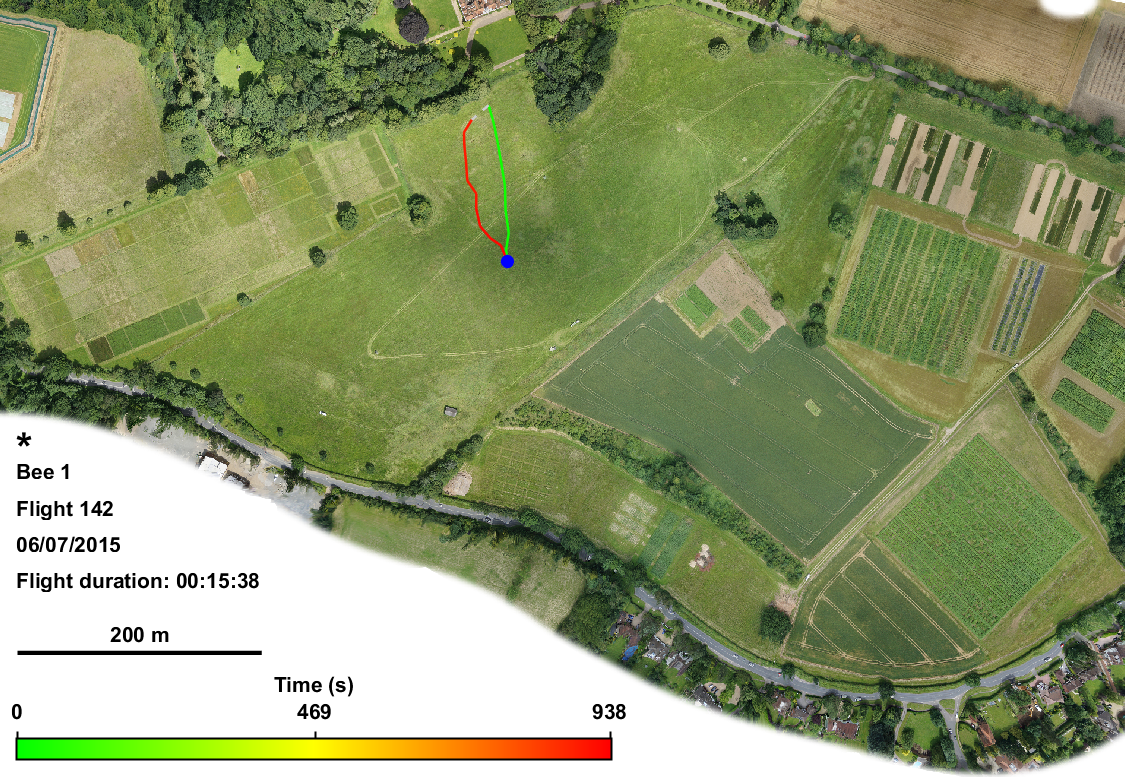

Supplement: S4 File — All flights made by Bee 1 during the period 05/07/15–06/07/15. Each figure represents a single flight. Bee ID, flight number, date of recording and the duration of each flight are shown in the bottom left corner of each figure. The position of the nest is marked by a blue circle. Colours represent the time from the start of each flight: initial portion of each flight is in green, changing smoothly through yellow until the end of the flight is shown in red. Grey dashed lines are used to join radar observations made more than 30 s apart, when the bee’s location was uncertain. (ZIP) [file pone.0160333.s006.zip › S143 Fig.tif]

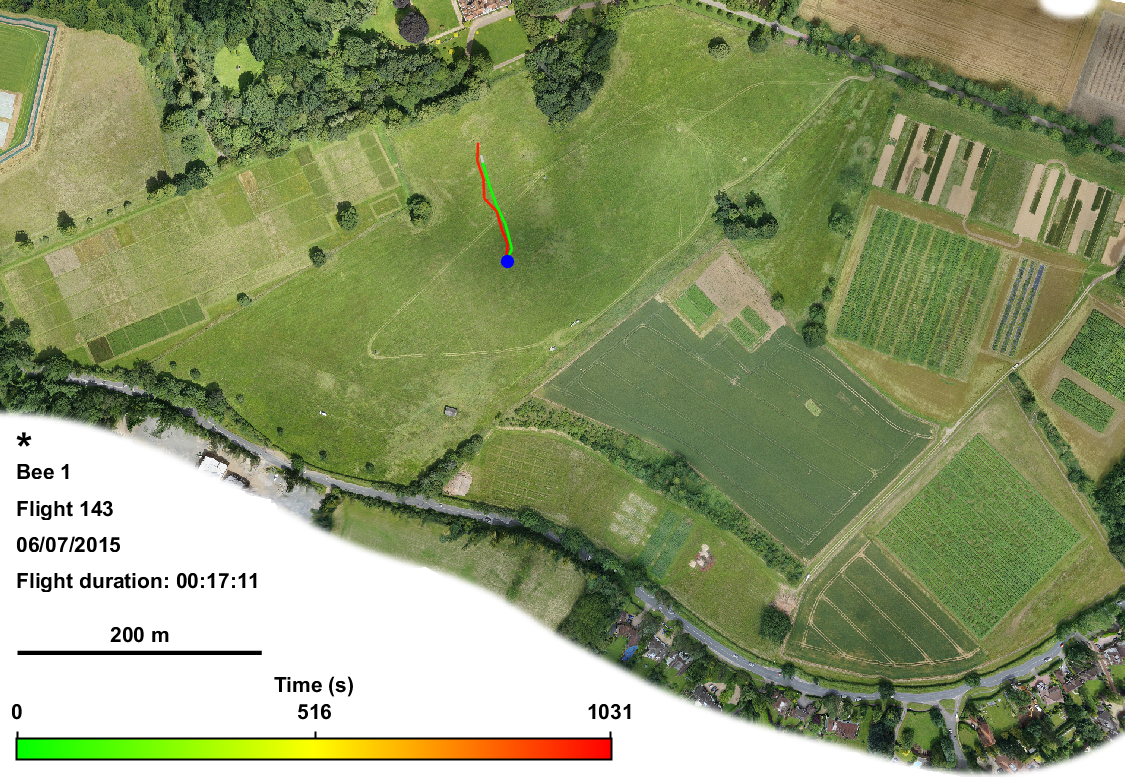

Supplement: S4 File — All flights made by Bee 1 during the period 05/07/15–06/07/15. Each figure represents a single flight. Bee ID, flight number, date of recording and the duration of each flight are shown in the bottom left corner of each figure. The position of the nest is marked by a blue circle. Colours represent the time from the start of each flight: initial portion of each flight is in green, changing smoothly through yellow until the end of the flight is shown in red. Grey dashed lines are used to join radar observations made more than 30 s apart, when the bee’s location was uncertain. (ZIP) [file pone.0160333.s006.zip › S144 Fig.tif]

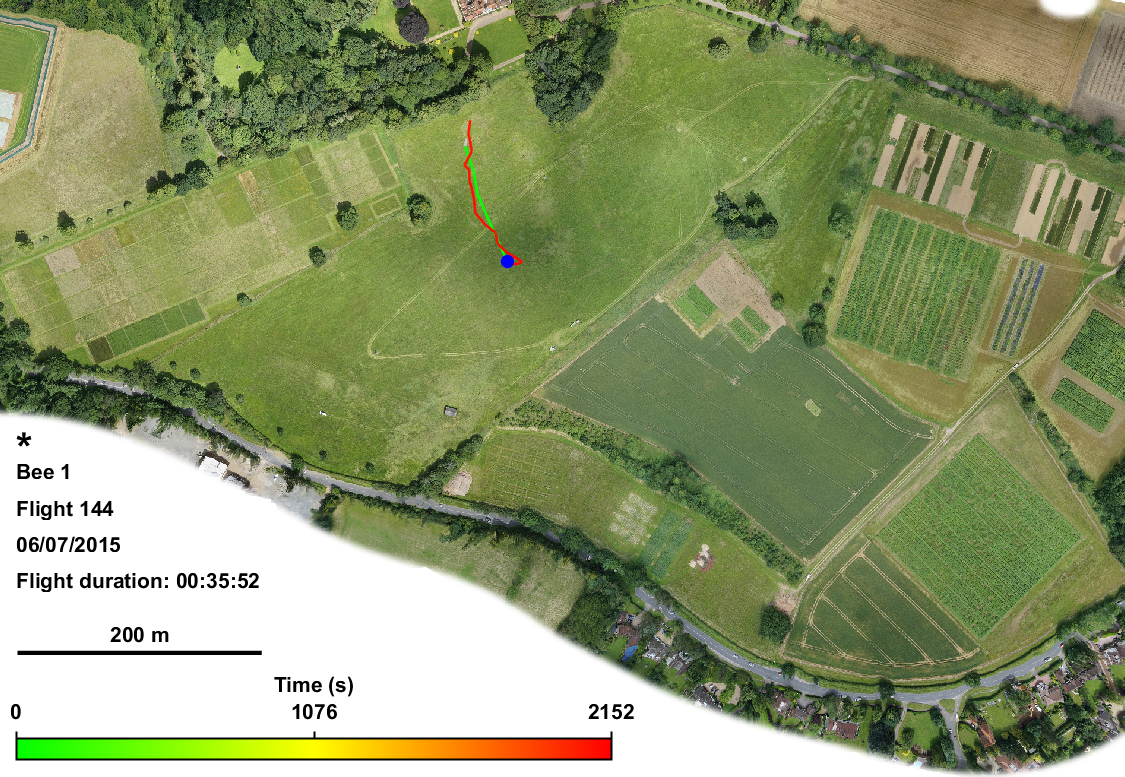

Supplement: S4 File — All flights made by Bee 1 during the period 05/07/15–06/07/15. Each figure represents a single flight. Bee ID, flight number, date of recording and the duration of each flight are shown in the bottom left corner of each figure. The position of the nest is marked by a blue circle. Colours represent the time from the start of each flight: initial portion of each flight is in green, changing smoothly through yellow until the end of the flight is shown in red. Grey dashed lines are used to join radar observations made more than 30 s apart, when the bee’s location was uncertain. (ZIP) [file pone.0160333.s006.zip › S145 Fig.tif]

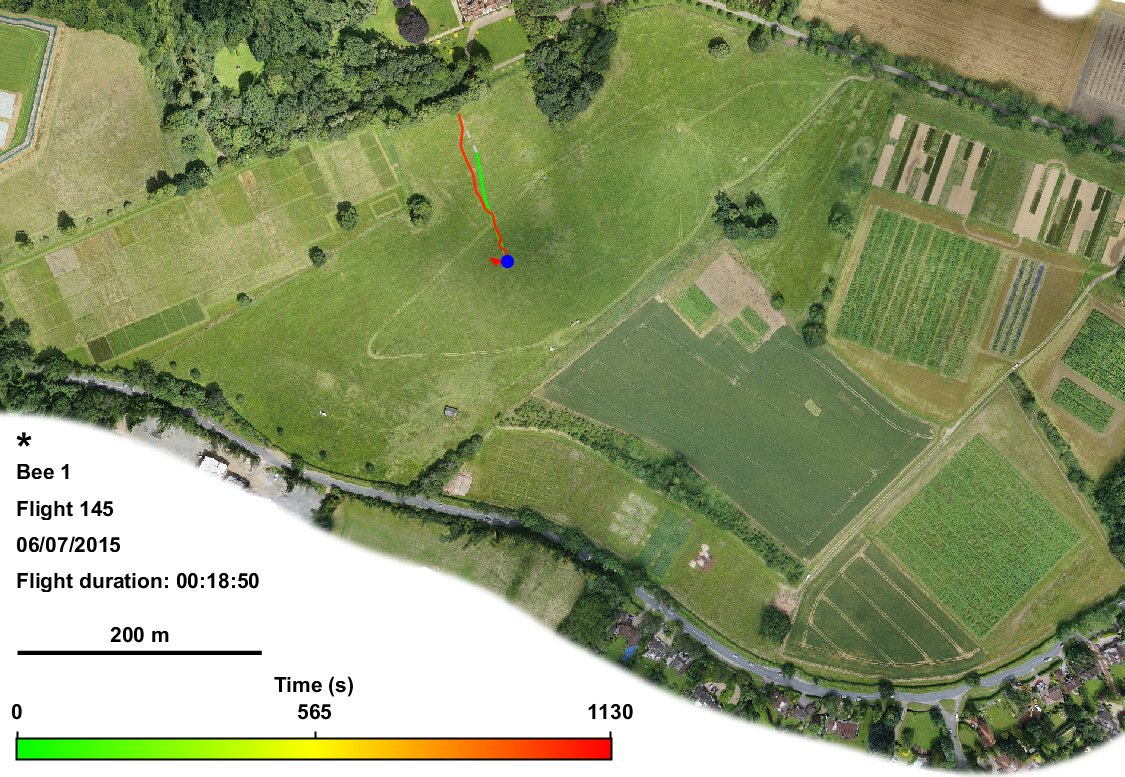

Supplement: S4 File — All flights made by Bee 1 during the period 05/07/15–06/07/15. Each figure represents a single flight. Bee ID, flight number, date of recording and the duration of each flight are shown in the bottom left corner of each figure. The position of the nest is marked by a blue circle. Colours represent the time from the start of each flight: initial portion of each flight is in green, changing smoothly through yellow until the end of the flight is shown in red. Grey dashed lines are used to join radar observations made more than 30 s apart, when the bee’s location was uncertain. (ZIP) [file pone.0160333.s006.zip › S146 Fig.tif]

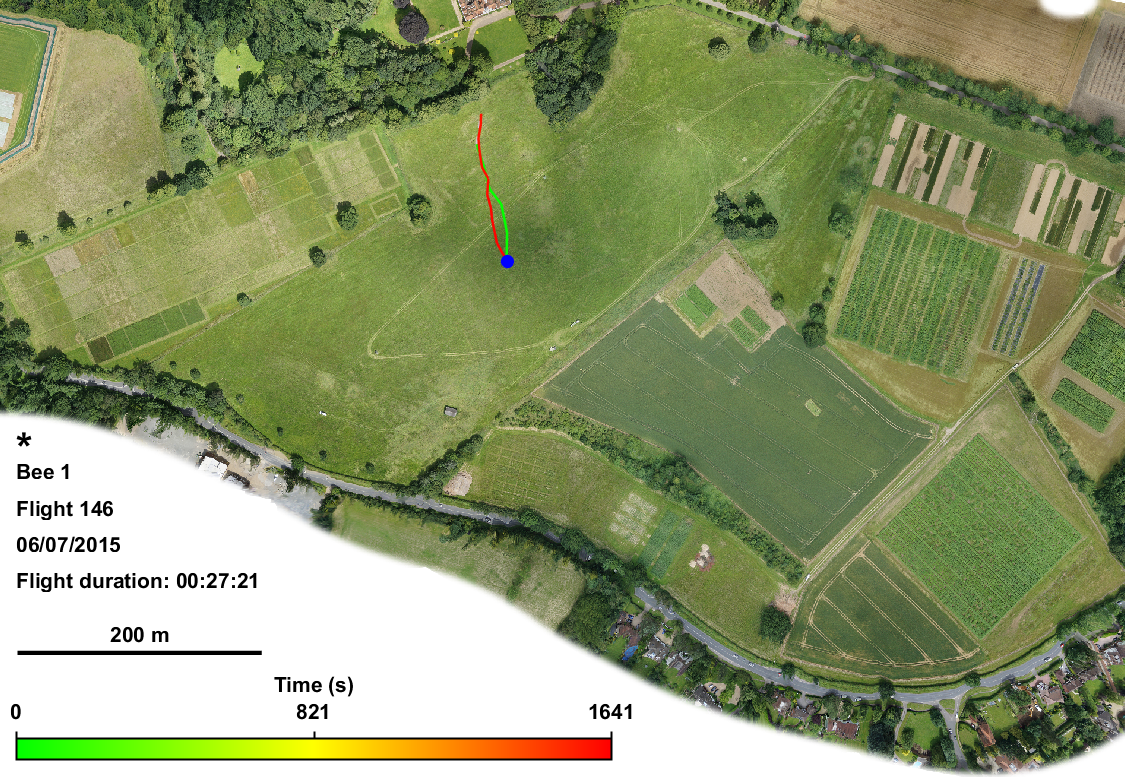

Supplement: S4 File — All flights made by Bee 1 during the period 05/07/15–06/07/15. Each figure represents a single flight. Bee ID, flight number, date of recording and the duration of each flight are shown in the bottom left corner of each figure. The position of the nest is marked by a blue circle. Colours represent the time from the start of each flight: initial portion of each flight is in green, changing smoothly through yellow until the end of the flight is shown in red. Grey dashed lines are used to join radar observations made more than 30 s apart, when the bee’s location was uncertain. (ZIP) [file pone.0160333.s006.zip › S147 Fig.tif]

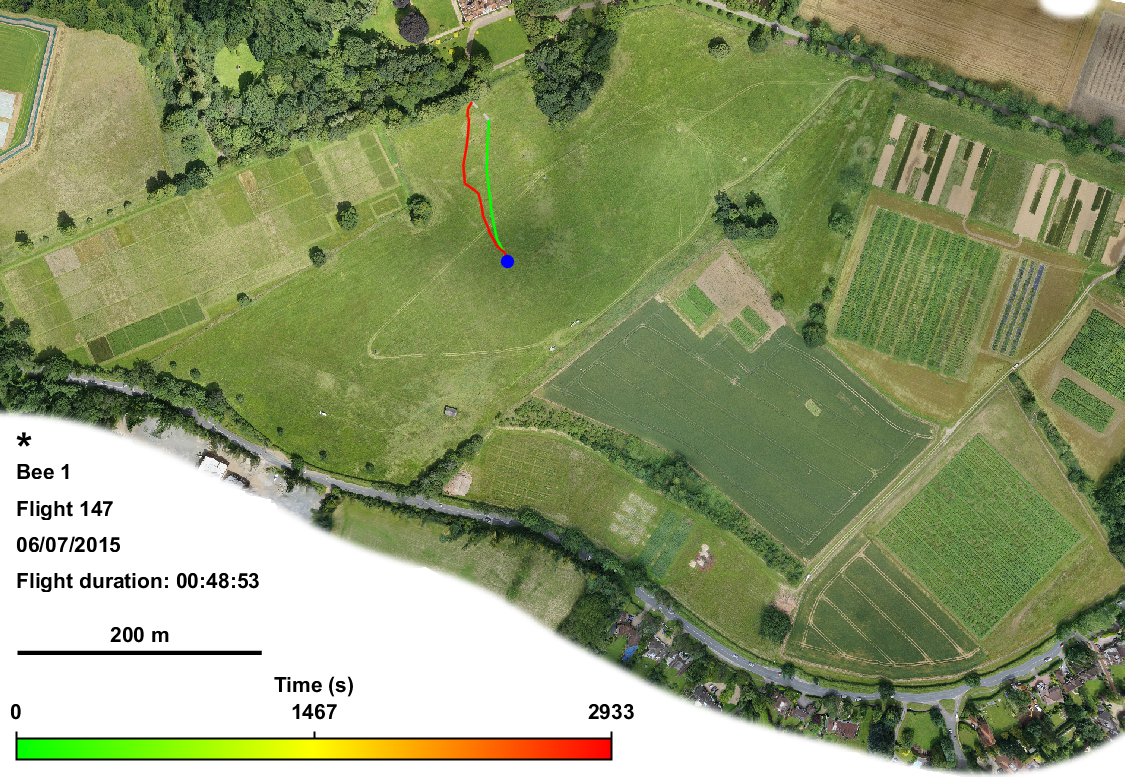

Supplement: S4 File — All flights made by Bee 1 during the period 05/07/15–06/07/15. Each figure represents a single flight. Bee ID, flight number, date of recording and the duration of each flight are shown in the bottom left corner of each figure. The position of the nest is marked by a blue circle. Colours represent the time from the start of each flight: initial portion of each flight is in green, changing smoothly through yellow until the end of the flight is shown in red. Grey dashed lines are used to join radar observations made more than 30 s apart, when the bee’s location was uncertain. (ZIP) [file pone.0160333.s006.zip › S148 Fig.tif]

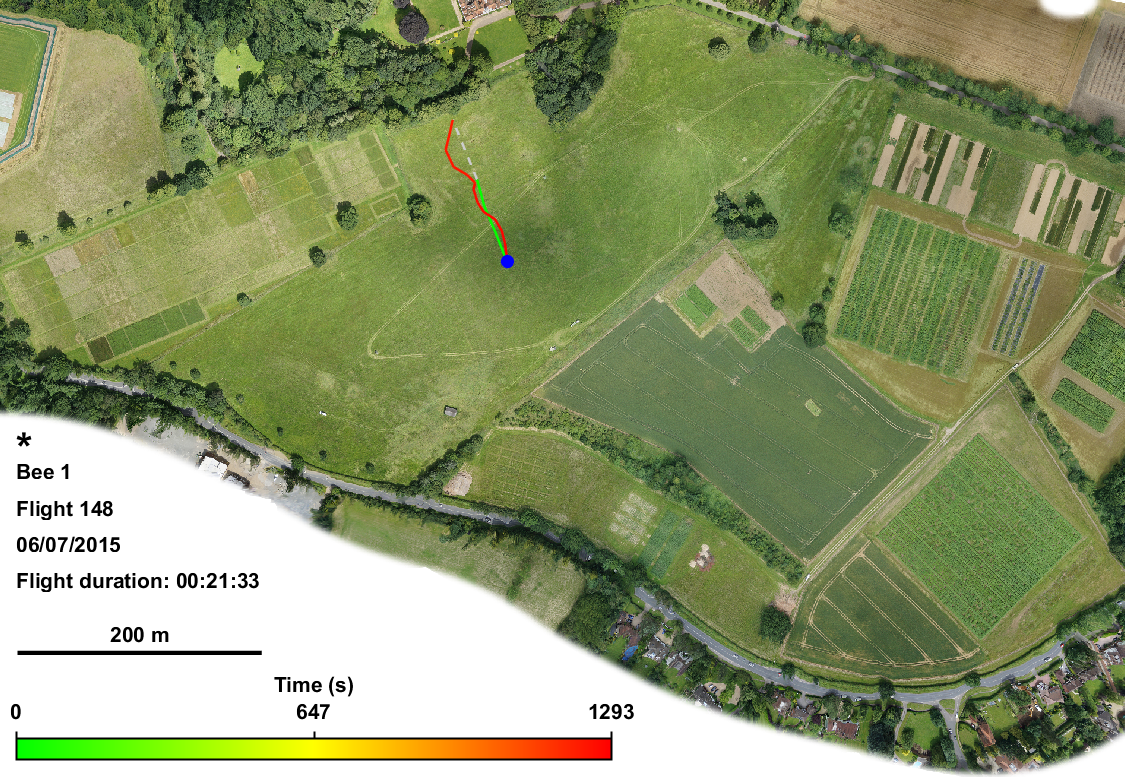

Supplement: S4 File — All flights made by Bee 1 during the period 05/07/15–06/07/15. Each figure represents a single flight. Bee ID, flight number, date of recording and the duration of each flight are shown in the bottom left corner of each figure. The position of the nest is marked by a blue circle. Colours represent the time from the start of each flight: initial portion of each flight is in green, changing smoothly through yellow until the end of the flight is shown in red. Grey dashed lines are used to join radar observations made more than 30 s apart, when the bee’s location was uncertain. (ZIP) [file pone.0160333.s006.zip › S149 Fig.tif]

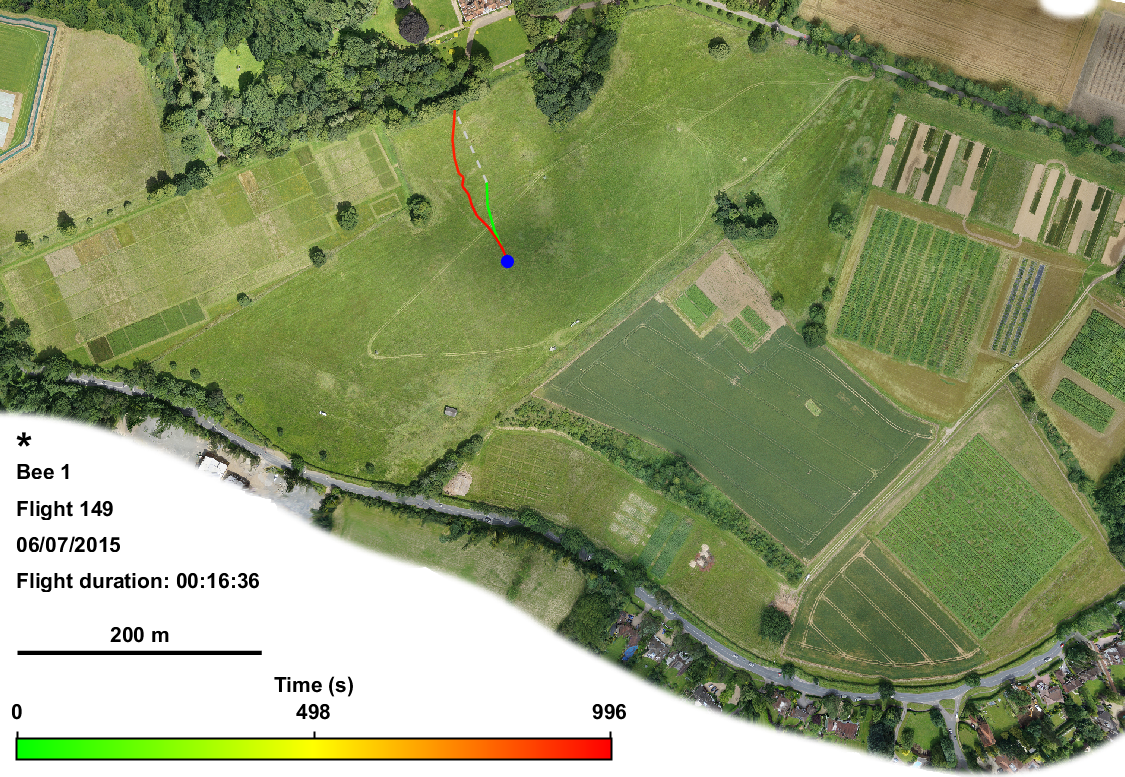

Supplement: S4 File — All flights made by Bee 1 during the period 05/07/15–06/07/15. Each figure represents a single flight. Bee ID, flight number, date of recording and the duration of each flight are shown in the bottom left corner of each figure. The position of the nest is marked by a blue circle. Colours represent the time from the start of each flight: initial portion of each flight is in green, changing smoothly through yellow until the end of the flight is shown in red. Grey dashed lines are used to join radar observations made more than 30 s apart, when the bee’s location was uncertain. (ZIP) [file pone.0160333.s006.zip › S150 Fig.tif]

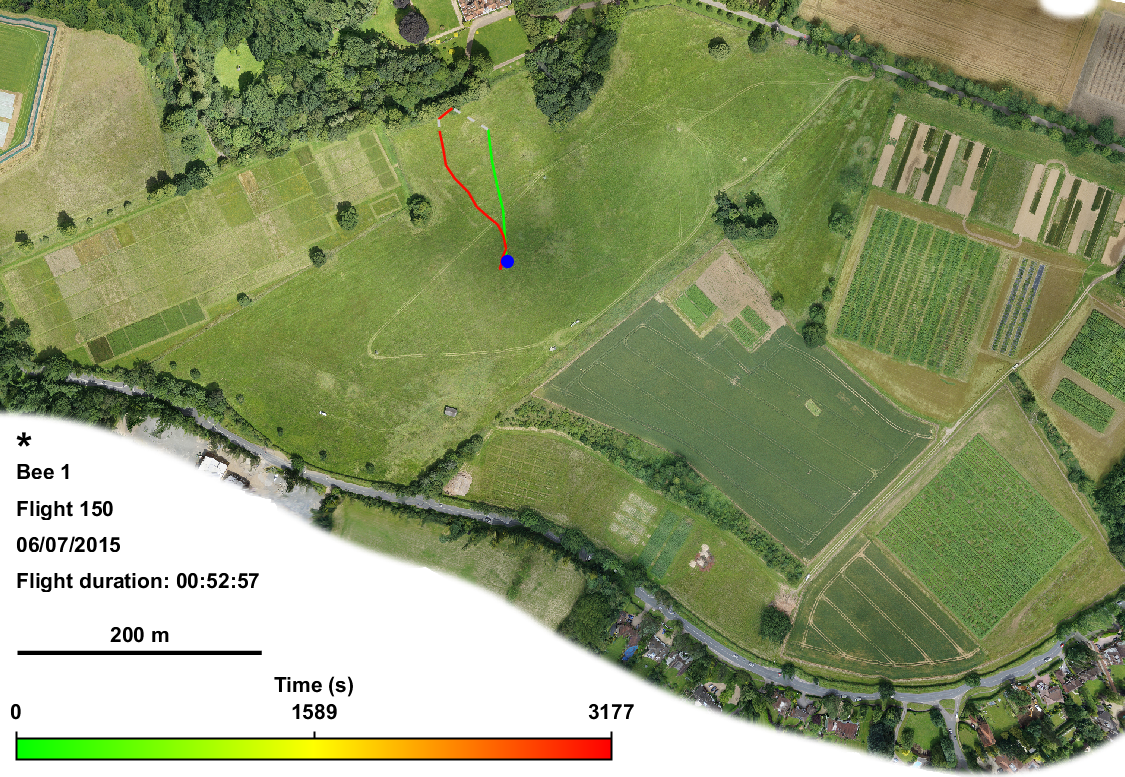

Supplement: S4 File — All flights made by Bee 1 during the period 05/07/15–06/07/15. Each figure represents a single flight. Bee ID, flight number, date of recording and the duration of each flight are shown in the bottom left corner of each figure. The position of the nest is marked by a blue circle. Colours represent the time from the start of each flight: initial portion of each flight is in green, changing smoothly through yellow until the end of the flight is shown in red. Grey dashed lines are used to join radar observations made more than 30 s apart, when the bee’s location was uncertain. (ZIP) [file pone.0160333.s006.zip › S151 Fig.tif]

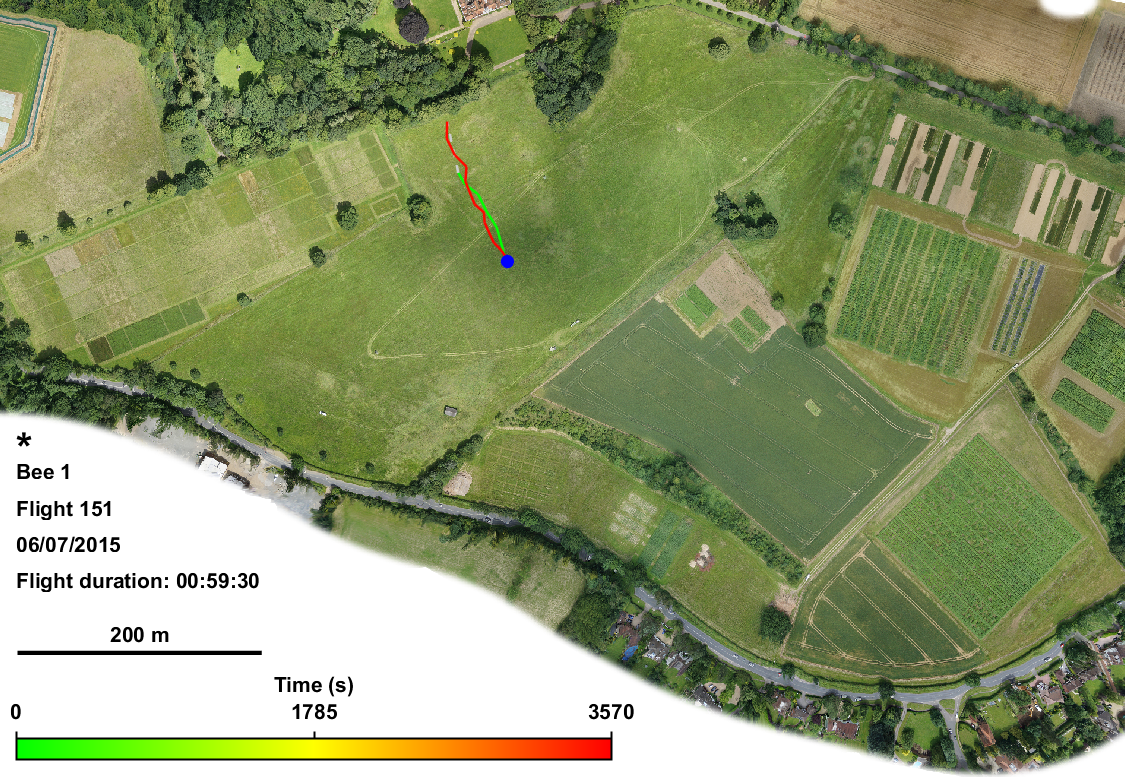

Supplement: S4 File — All flights made by Bee 1 during the period 05/07/15–06/07/15. Each figure represents a single flight. Bee ID, flight number, date of recording and the duration of each flight are shown in the bottom left corner of each figure. The position of the nest is marked by a blue circle. Colours represent the time from the start of each flight: initial portion of each flight is in green, changing smoothly through yellow until the end of the flight is shown in red. Grey dashed lines are used to join radar observations made more than 30 s apart, when the bee’s location was uncertain. (ZIP) [file pone.0160333.s006.zip › S152 Fig.tif]

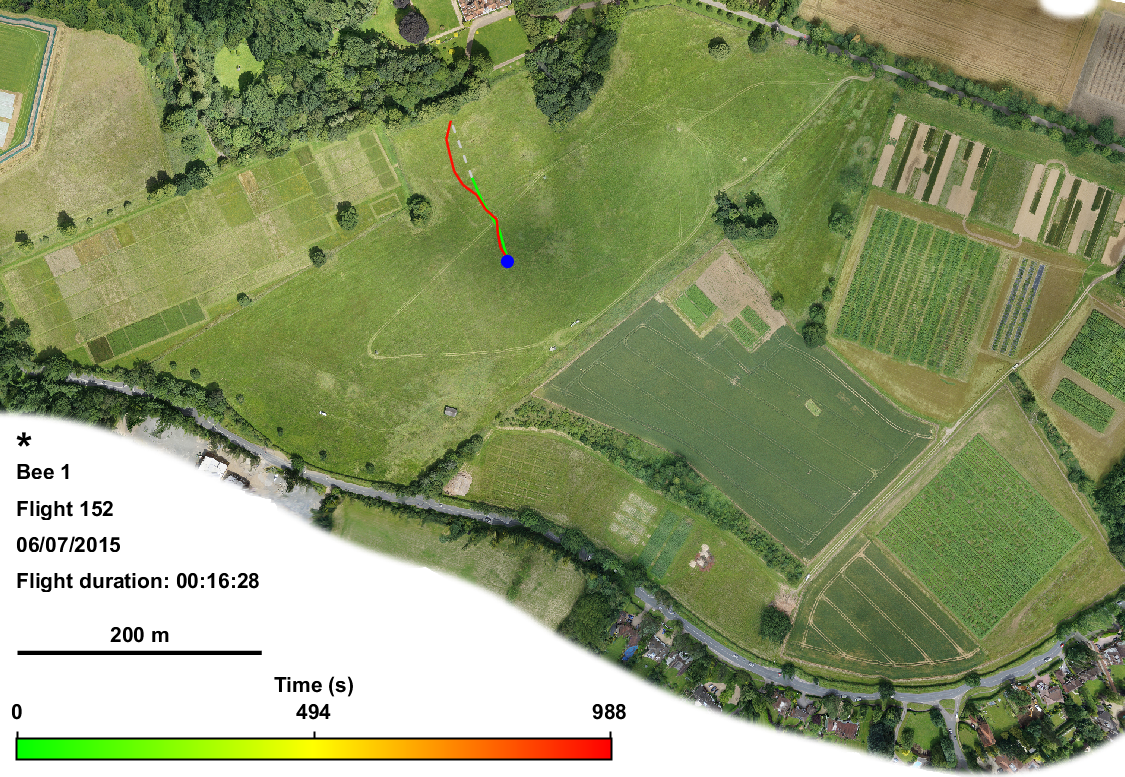

Supplement: S4 File — All flights made by Bee 1 during the period 05/07/15–06/07/15. Each figure represents a single flight. Bee ID, flight number, date of recording and the duration of each flight are shown in the bottom left corner of each figure. The position of the nest is marked by a blue circle. Colours represent the time from the start of each flight: initial portion of each flight is in green, changing smoothly through yellow until the end of the flight is shown in red. Grey dashed lines are used to join radar observations made more than 30 s apart, when the bee’s location was uncertain. (ZIP) [file pone.0160333.s006.zip › S153 Fig.tif]

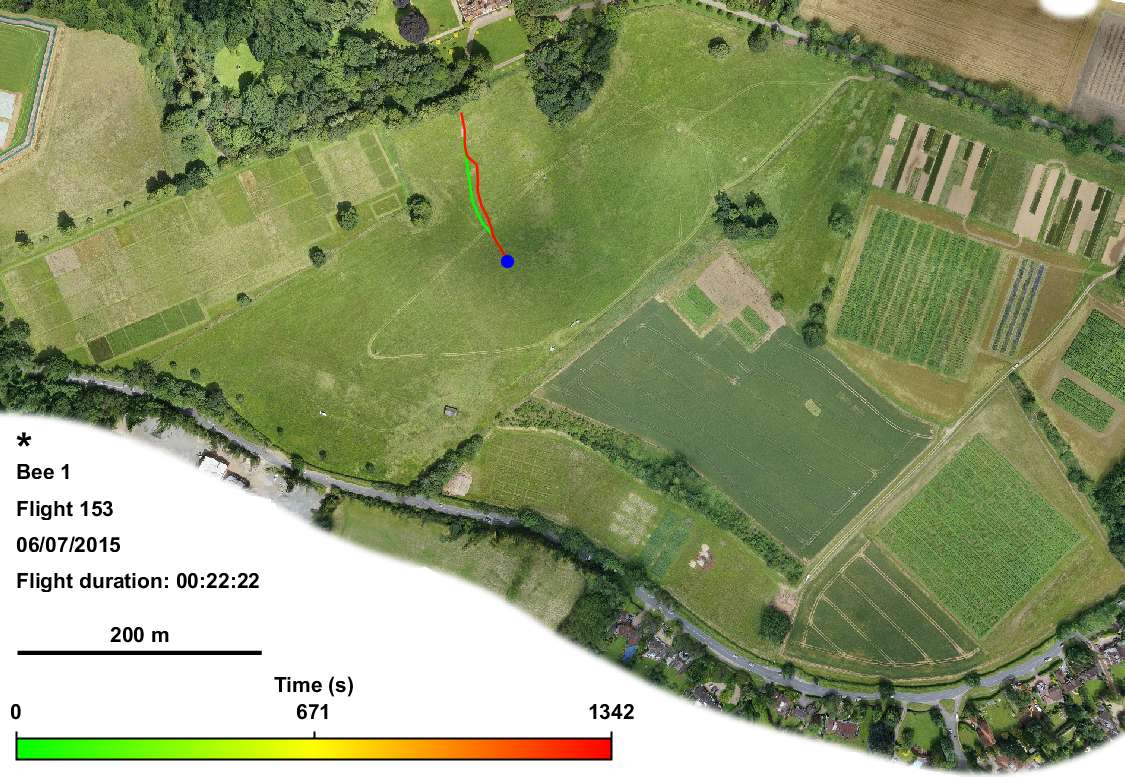

Supplement: S4 File — All flights made by Bee 1 during the period 05/07/15–06/07/15. Each figure represents a single flight. Bee ID, flight number, date of recording and the duration of each flight are shown in the bottom left corner of each figure. The position of the nest is marked by a blue circle. Colours represent the time from the start of each flight: initial portion of each flight is in green, changing smoothly through yellow until the end of the flight is shown in red. Grey dashed lines are used to join radar observations made more than 30 s apart, when the bee’s location was uncertain. (ZIP) [file pone.0160333.s006.zip › S154 Fig.tif]

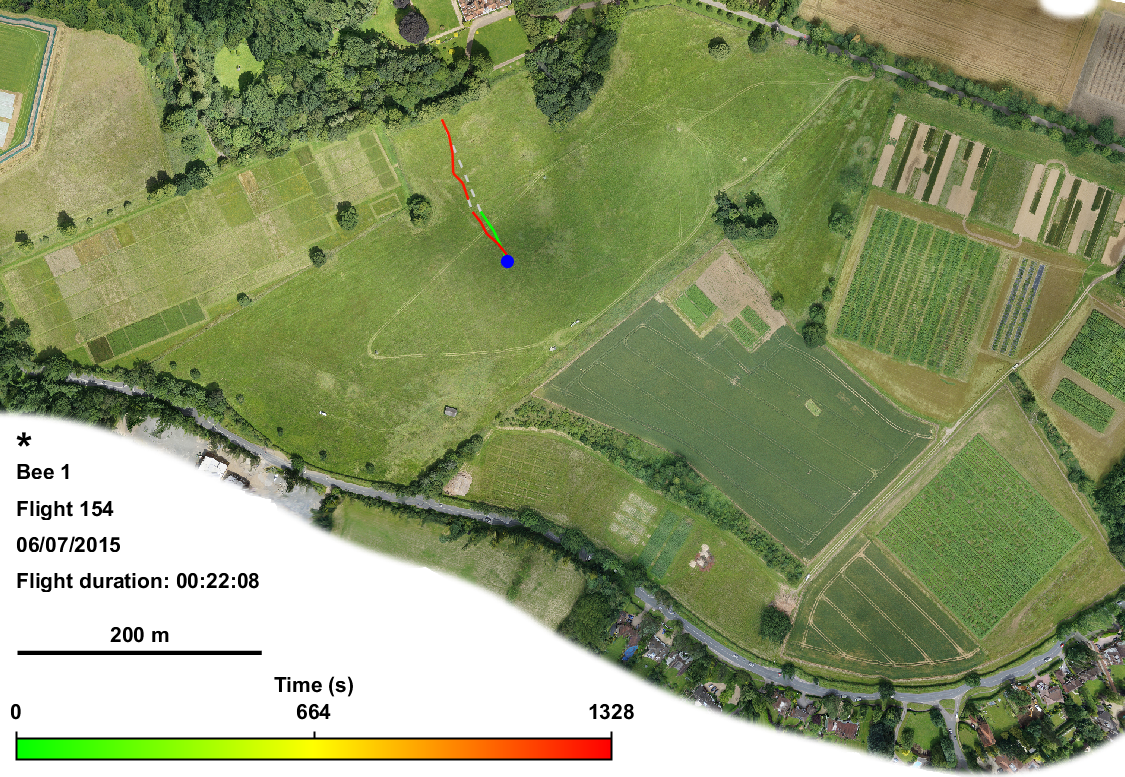

Supplement: S4 File — All flights made by Bee 1 during the period 05/07/15–06/07/15. Each figure represents a single flight. Bee ID, flight number, date of recording and the duration of each flight are shown in the bottom left corner of each figure. The position of the nest is marked by a blue circle. Colours represent the time from the start of each flight: initial portion of each flight is in green, changing smoothly through yellow until the end of the flight is shown in red. Grey dashed lines are used to join radar observations made more than 30 s apart, when the bee’s location was uncertain. (ZIP) [file pone.0160333.s006.zip › S155 Fig.tif]

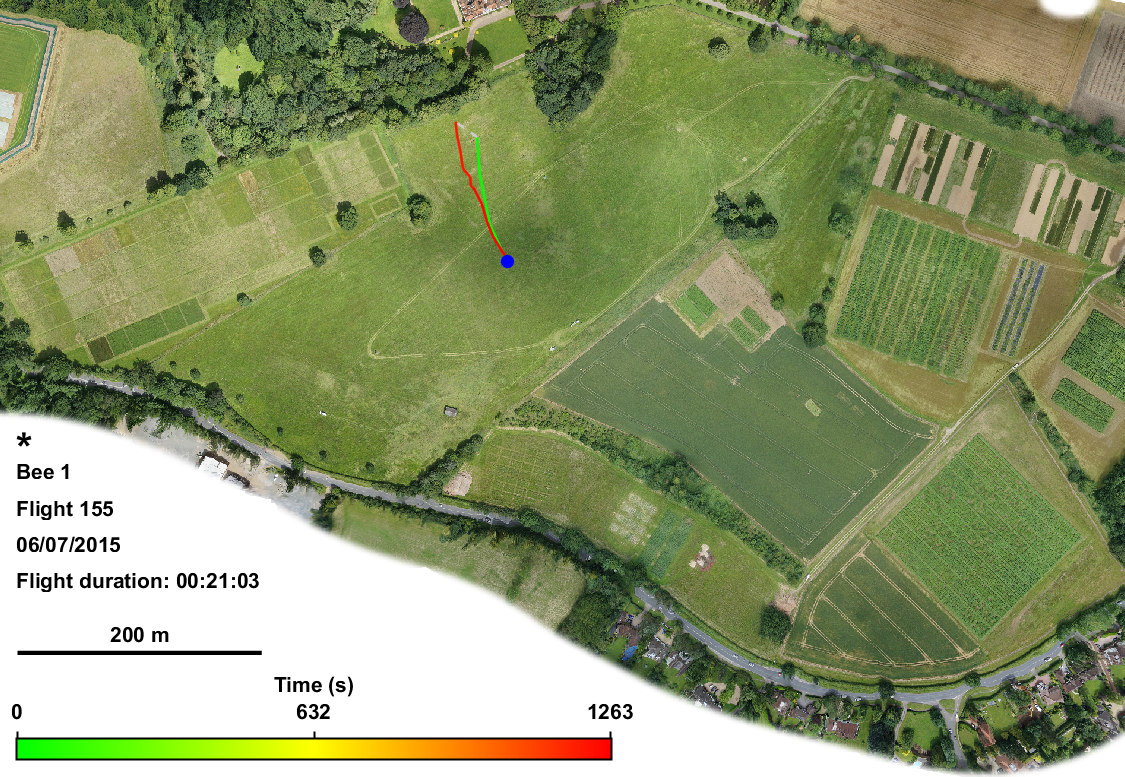

Supplement: S4 File — All flights made by Bee 1 during the period 05/07/15–06/07/15. Each figure represents a single flight. Bee ID, flight number, date of recording and the duration of each flight are shown in the bottom left corner of each figure. The position of the nest is marked by a blue circle. Colours represent the time from the start of each flight: initial portion of each flight is in green, changing smoothly through yellow until the end of the flight is shown in red. Grey dashed lines are used to join radar observations made more than 30 s apart, when the bee’s location was uncertain. (ZIP) [file pone.0160333.s006.zip › S156 Fig.tif]

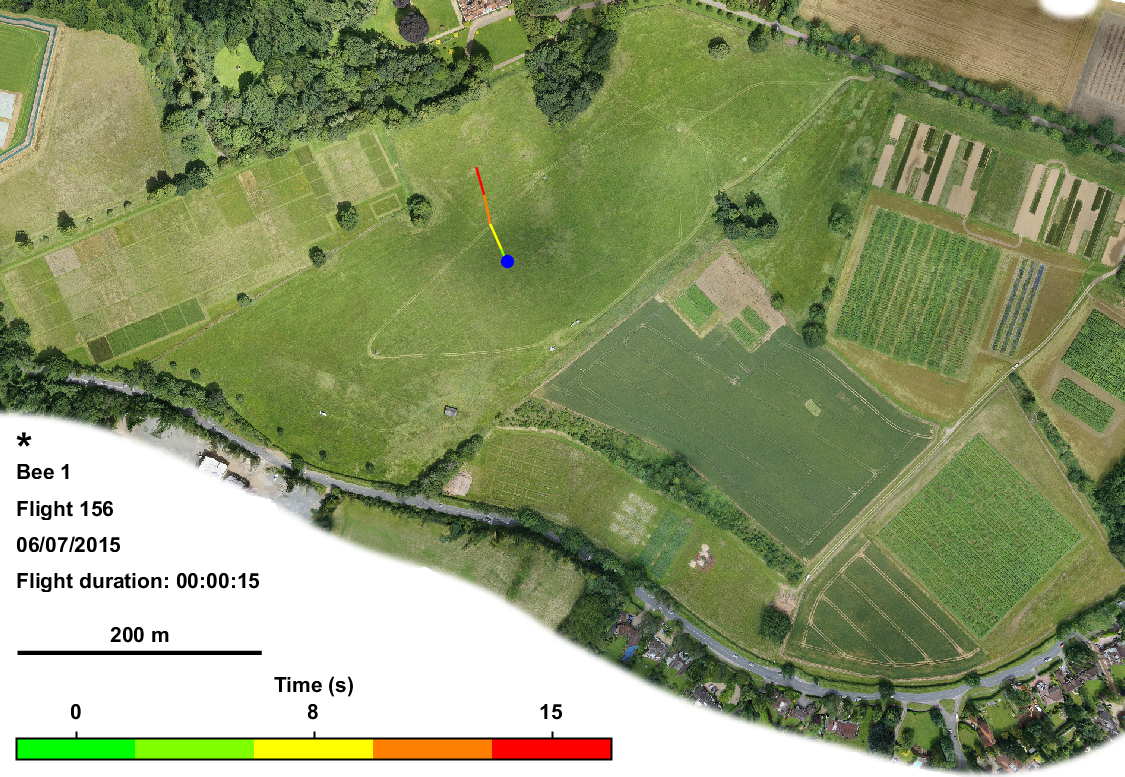

Supplement: S4 File — All flights made by Bee 1 during the period 05/07/15–06/07/15. Each figure represents a single flight. Bee ID, flight number, date of recording and the duration of each flight are shown in the bottom left corner of each figure. The position of the nest is marked by a blue circle. Colours represent the time from the start of each flight: initial portion of each flight is in green, changing smoothly through yellow until the end of the flight is shown in red. Grey dashed lines are used to join radar observations made more than 30 s apart, when the bee’s location was uncertain. (ZIP) [file pone.0160333.s006.zip › S157 Fig.tif]

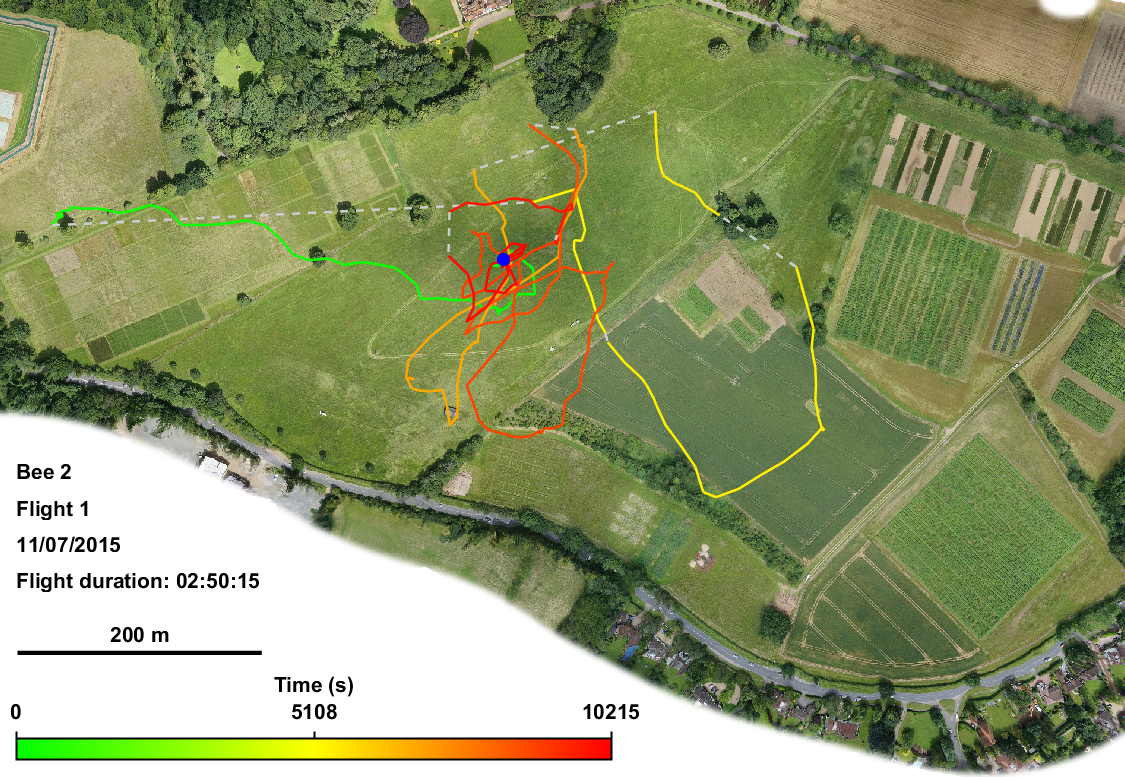

Supplement: S5 File — All flights made by Bee 2 during the period 11/07/15–16/07/15. Each figure represents a single flight. Bee ID, flight number, date of recording and the duration of each flight are shown in the bottom left corner of each figure. The position of the nest is marked by a blue circle. Colours represent the time from the start of each flight: initial portion of each flight is in green, changing smoothly through yellow until the end of the flight is shown in red. Grey dashed lines are used to join radar observations made more than 30 s apart, when the bee’s location was uncertain. (ZIP) [file pone.0160333.s007.zip › S158 Fig.tif]

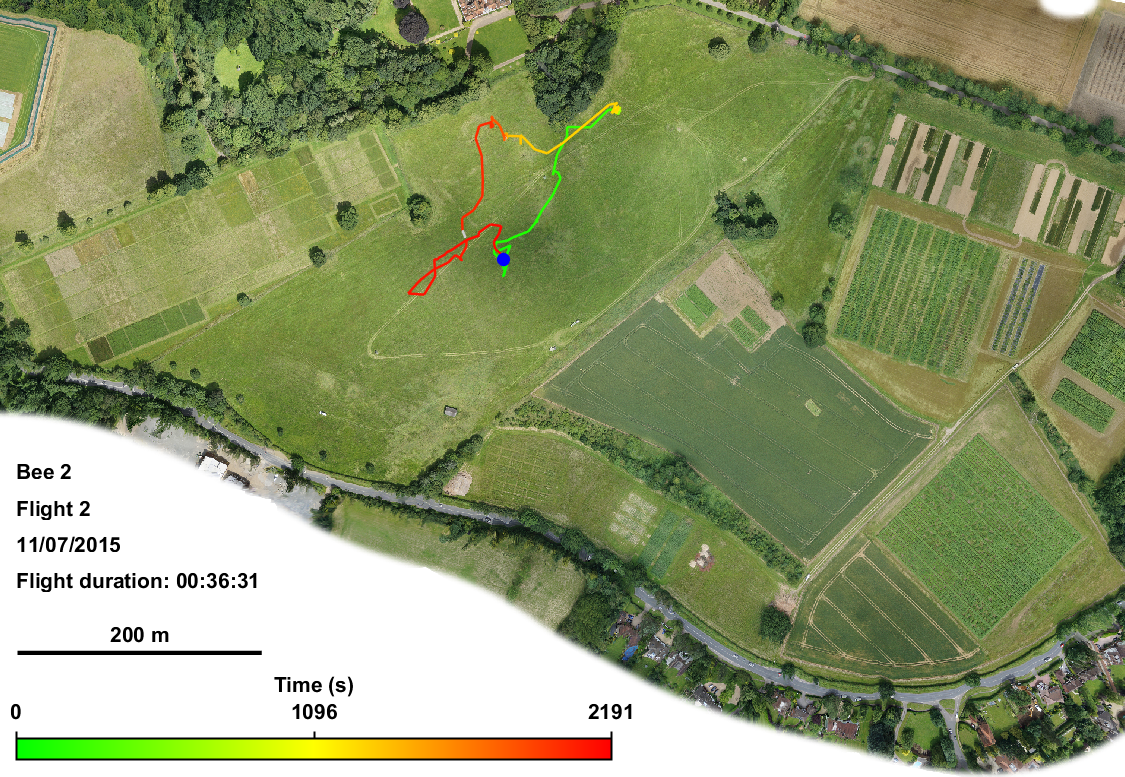

Supplement: S5 File — All flights made by Bee 2 during the period 11/07/15–16/07/15. Each figure represents a single flight. Bee ID, flight number, date of recording and the duration of each flight are shown in the bottom left corner of each figure. The position of the nest is marked by a blue circle. Colours represent the time from the start of each flight: initial portion of each flight is in green, changing smoothly through yellow until the end of the flight is shown in red. Grey dashed lines are used to join radar observations made more than 30 s apart, when the bee’s location was uncertain. (ZIP) [file pone.0160333.s007.zip › S159 Fig.tif]

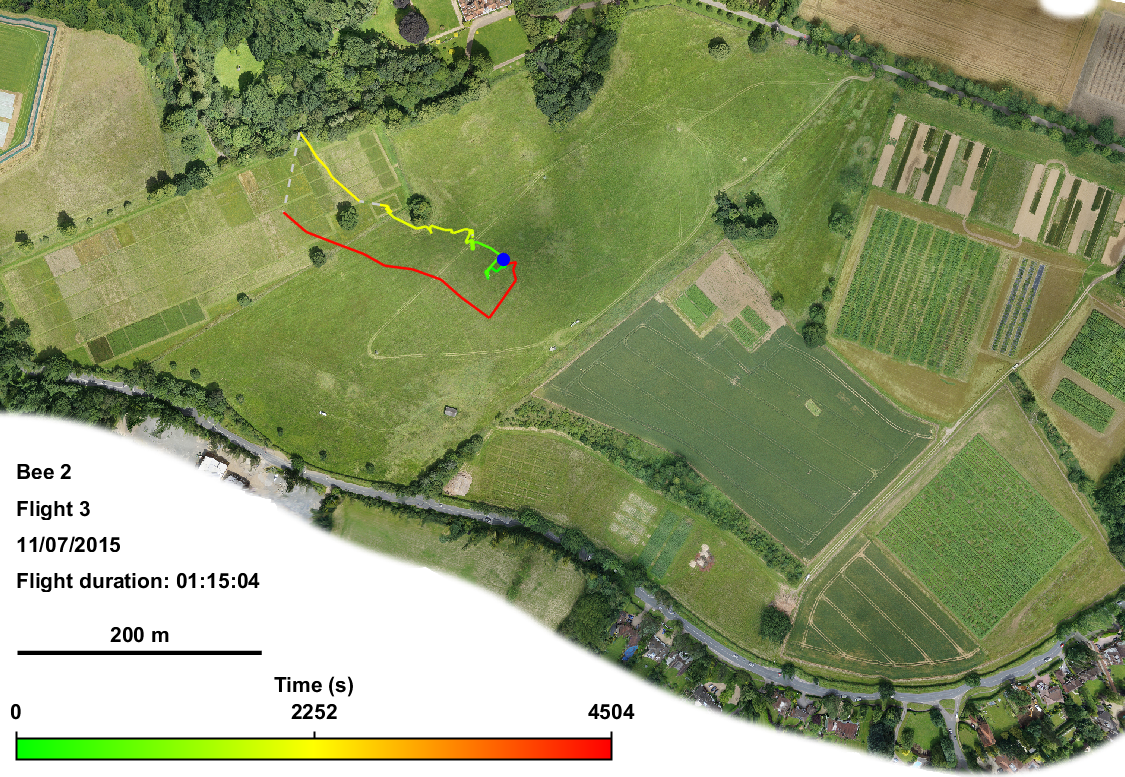

Supplement: S5 File — All flights made by Bee 2 during the period 11/07/15–16/07/15. Each figure represents a single flight. Bee ID, flight number, date of recording and the duration of each flight are shown in the bottom left corner of each figure. The position of the nest is marked by a blue circle. Colours represent the time from the start of each flight: initial portion of each flight is in green, changing smoothly through yellow until the end of the flight is shown in red. Grey dashed lines are used to join radar observations made more than 30 s apart, when the bee’s location was uncertain. (ZIP) [file pone.0160333.s007.zip › S160 Fig.tif]

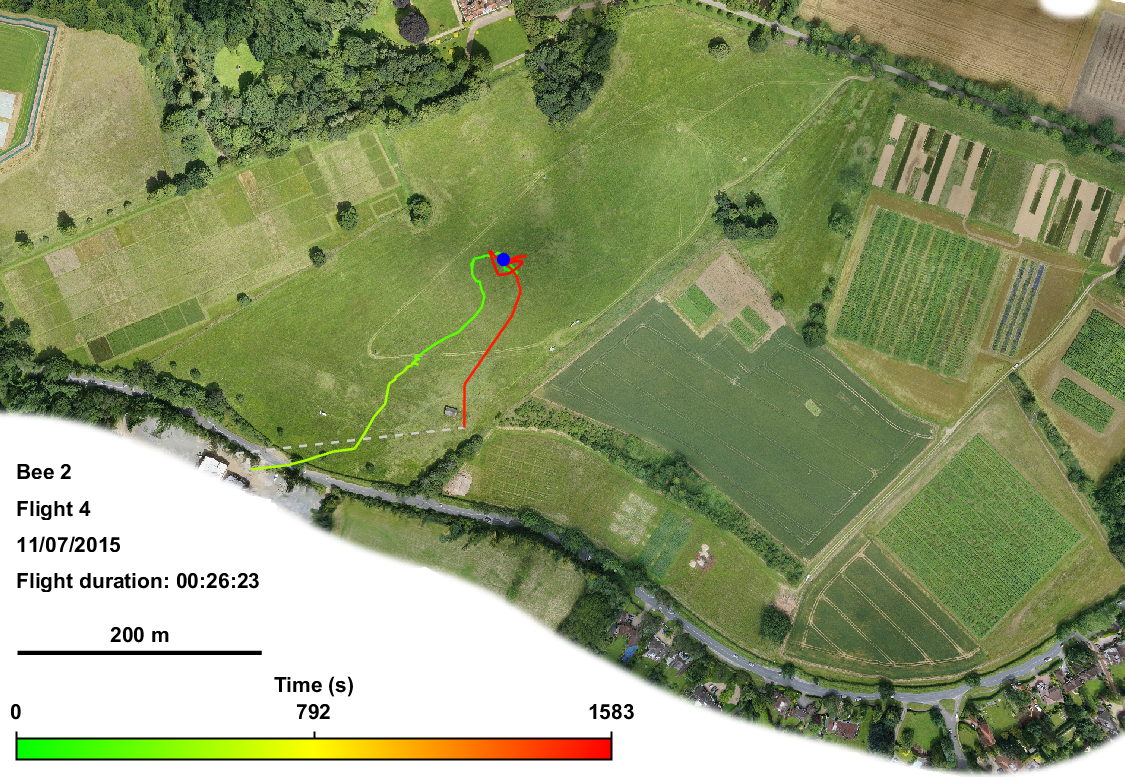

Supplement: S5 File — All flights made by Bee 2 during the period 11/07/15–16/07/15. Each figure represents a single flight. Bee ID, flight number, date of recording and the duration of each flight are shown in the bottom left corner of each figure. The position of the nest is marked by a blue circle. Colours represent the time from the start of each flight: initial portion of each flight is in green, changing smoothly through yellow until the end of the flight is shown in red. Grey dashed lines are used to join radar observations made more than 30 s apart, when the bee’s location was uncertain. (ZIP) [file pone.0160333.s007.zip › S161 Fig.tif]

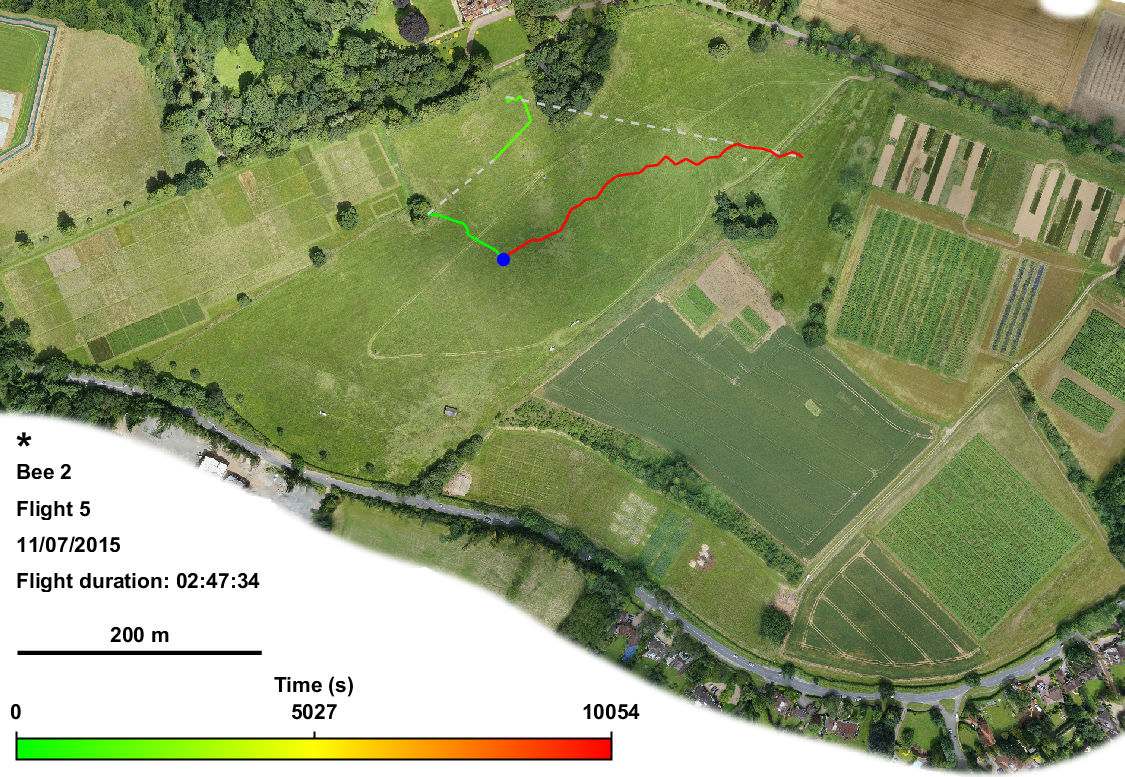

Supplement: S5 File — All flights made by Bee 2 during the period 11/07/15–16/07/15. Each figure represents a single flight. Bee ID, flight number, date of recording and the duration of each flight are shown in the bottom left corner of each figure. The position of the nest is marked by a blue circle. Colours represent the time from the start of each flight: initial portion of each flight is in green, changing smoothly through yellow until the end of the flight is shown in red. Grey dashed lines are used to join radar observations made more than 30 s apart, when the bee’s location was uncertain. (ZIP) [file pone.0160333.s007.zip › S162 Fig.tif]

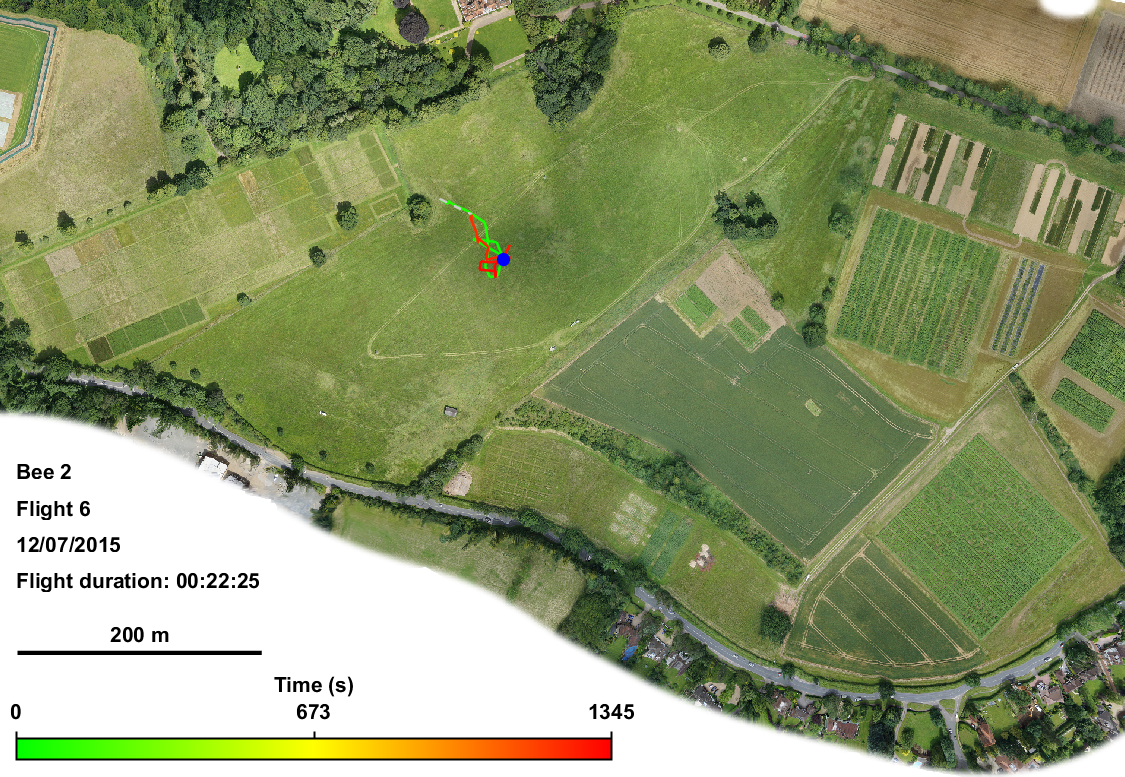

Supplement: S5 File — All flights made by Bee 2 during the period 11/07/15–16/07/15. Each figure represents a single flight. Bee ID, flight number, date of recording and the duration of each flight are shown in the bottom left corner of each figure. The position of the nest is marked by a blue circle. Colours represent the time from the start of each flight: initial portion of each flight is in green, changing smoothly through yellow until the end of the flight is shown in red. Grey dashed lines are used to join radar observations made more than 30 s apart, when the bee’s location was uncertain. (ZIP) [file pone.0160333.s007.zip › S163 Fig.tif]

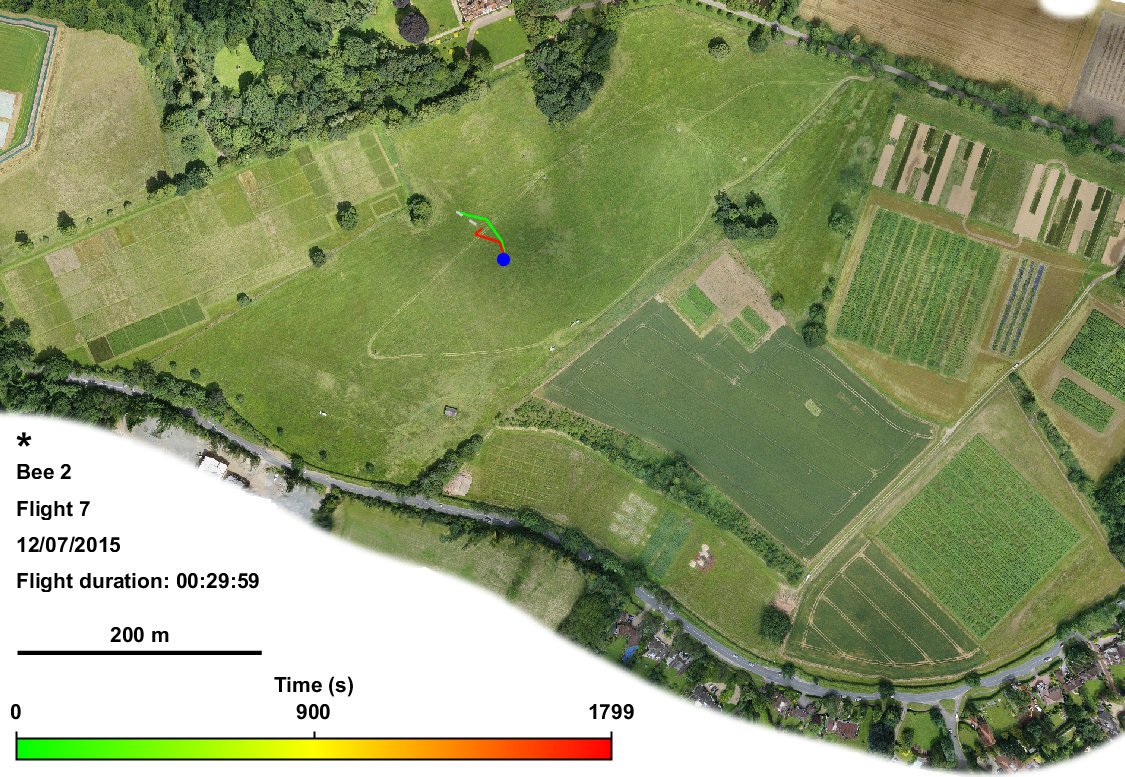

Supplement: S5 File — All flights made by Bee 2 during the period 11/07/15–16/07/15. Each figure represents a single flight. Bee ID, flight number, date of recording and the duration of each flight are shown in the bottom left corner of each figure. The position of the nest is marked by a blue circle. Colours represent the time from the start of each flight: initial portion of each flight is in green, changing smoothly through yellow until the end of the flight is shown in red. Grey dashed lines are used to join radar observations made more than 30 s apart, when the bee’s location was uncertain. (ZIP) [file pone.0160333.s007.zip › S164 Fig.tif]

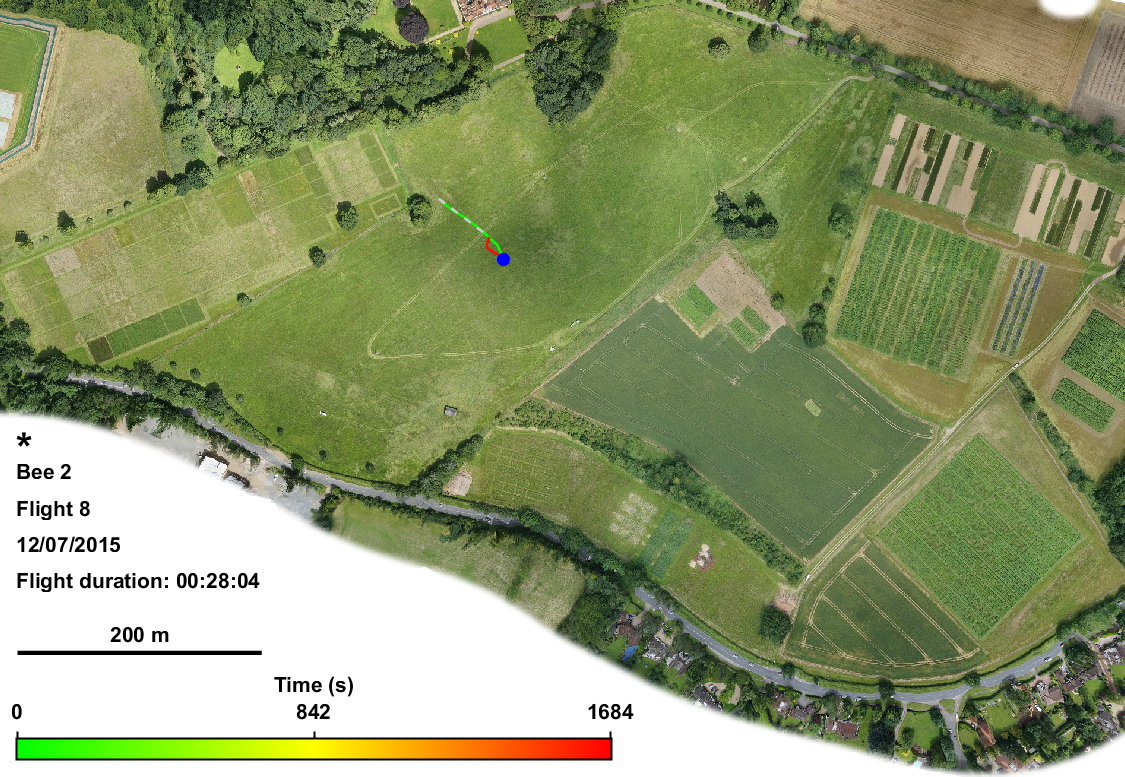

Supplement: S5 File — All flights made by Bee 2 during the period 11/07/15–16/07/15. Each figure represents a single flight. Bee ID, flight number, date of recording and the duration of each flight are shown in the bottom left corner of each figure. The position of the nest is marked by a blue circle. Colours represent the time from the start of each flight: initial portion of each flight is in green, changing smoothly through yellow until the end of the flight is shown in red. Grey dashed lines are used to join radar observations made more than 30 s apart, when the bee’s location was uncertain. (ZIP) [file pone.0160333.s007.zip › S165 Fig.tif]

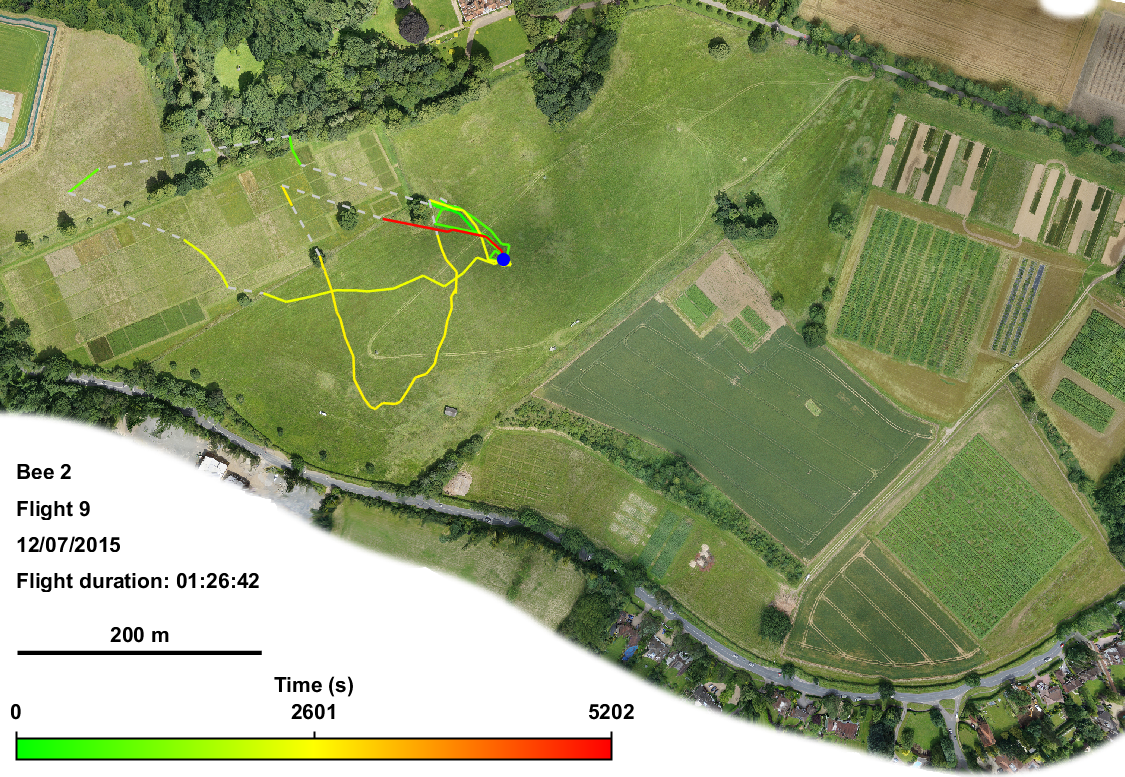

Supplement: S5 File — All flights made by Bee 2 during the period 11/07/15–16/07/15. Each figure represents a single flight. Bee ID, flight number, date of recording and the duration of each flight are shown in the bottom left corner of each figure. The position of the nest is marked by a blue circle. Colours represent the time from the start of each flight: initial portion of each flight is in green, changing smoothly through yellow until the end of the flight is shown in red. Grey dashed lines are used to join radar observations made more than 30 s apart, when the bee’s location was uncertain. (ZIP) [file pone.0160333.s007.zip › S166 Fig.tif]

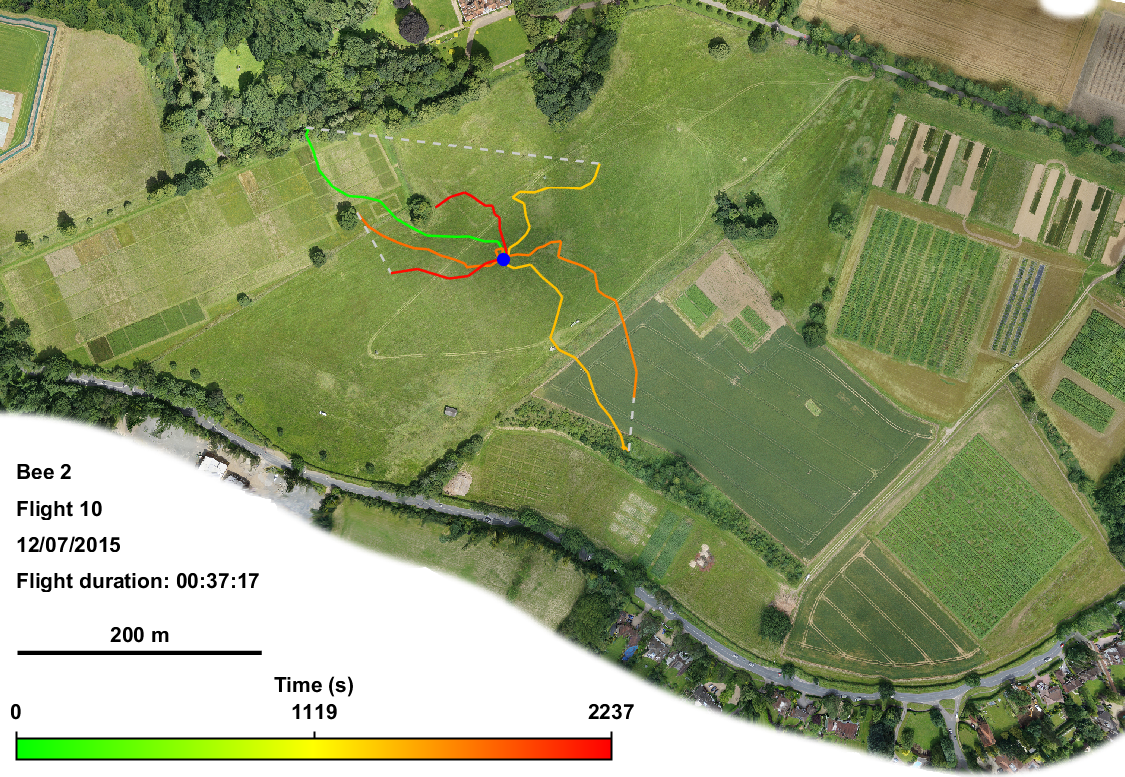

Supplement: S5 File — All flights made by Bee 2 during the period 11/07/15–16/07/15. Each figure represents a single flight. Bee ID, flight number, date of recording and the duration of each flight are shown in the bottom left corner of each figure. The position of the nest is marked by a blue circle. Colours represent the time from the start of each flight: initial portion of each flight is in green, changing smoothly through yellow until the end of the flight is shown in red. Grey dashed lines are used to join radar observations made more than 30 s apart, when the bee’s location was uncertain. (ZIP) [file pone.0160333.s007.zip › S167 Fig.tif]

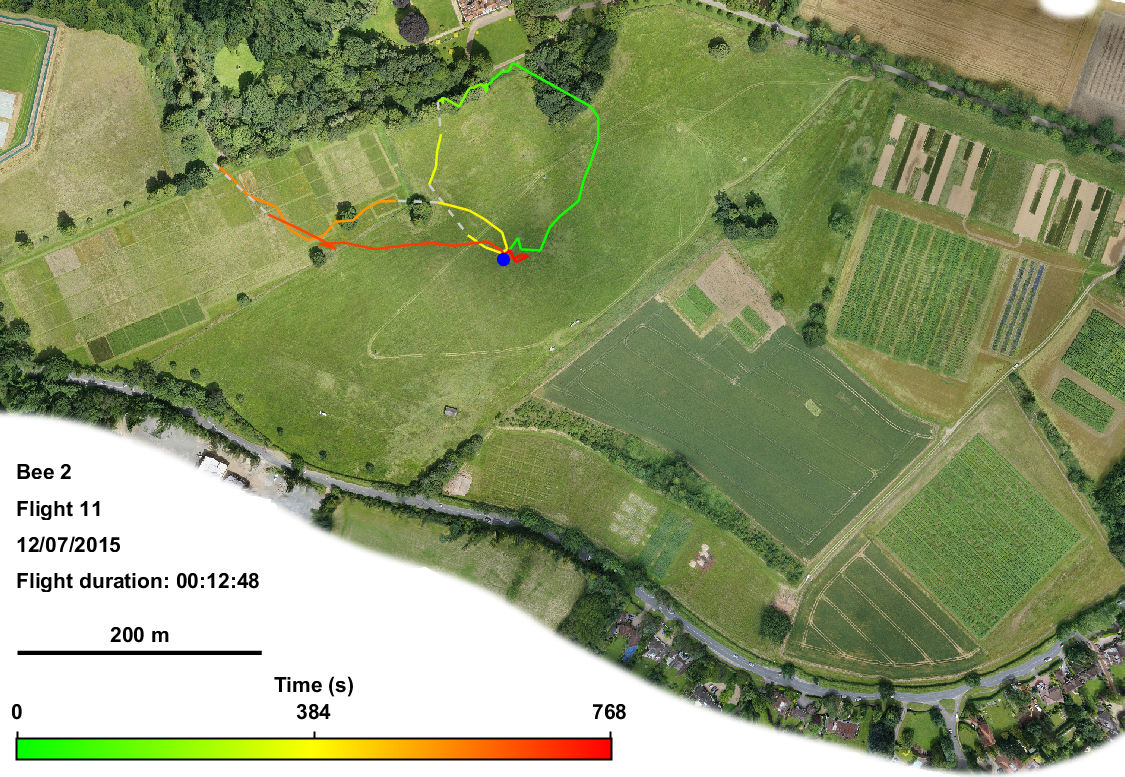

Supplement: S5 File — All flights made by Bee 2 during the period 11/07/15–16/07/15. Each figure represents a single flight. Bee ID, flight number, date of recording and the duration of each flight are shown in the bottom left corner of each figure. The position of the nest is marked by a blue circle. Colours represent the time from the start of each flight: initial portion of each flight is in green, changing smoothly through yellow until the end of the flight is shown in red. Grey dashed lines are used to join radar observations made more than 30 s apart, when the bee’s location was uncertain. (ZIP) [file pone.0160333.s007.zip › S168 Fig.tif]

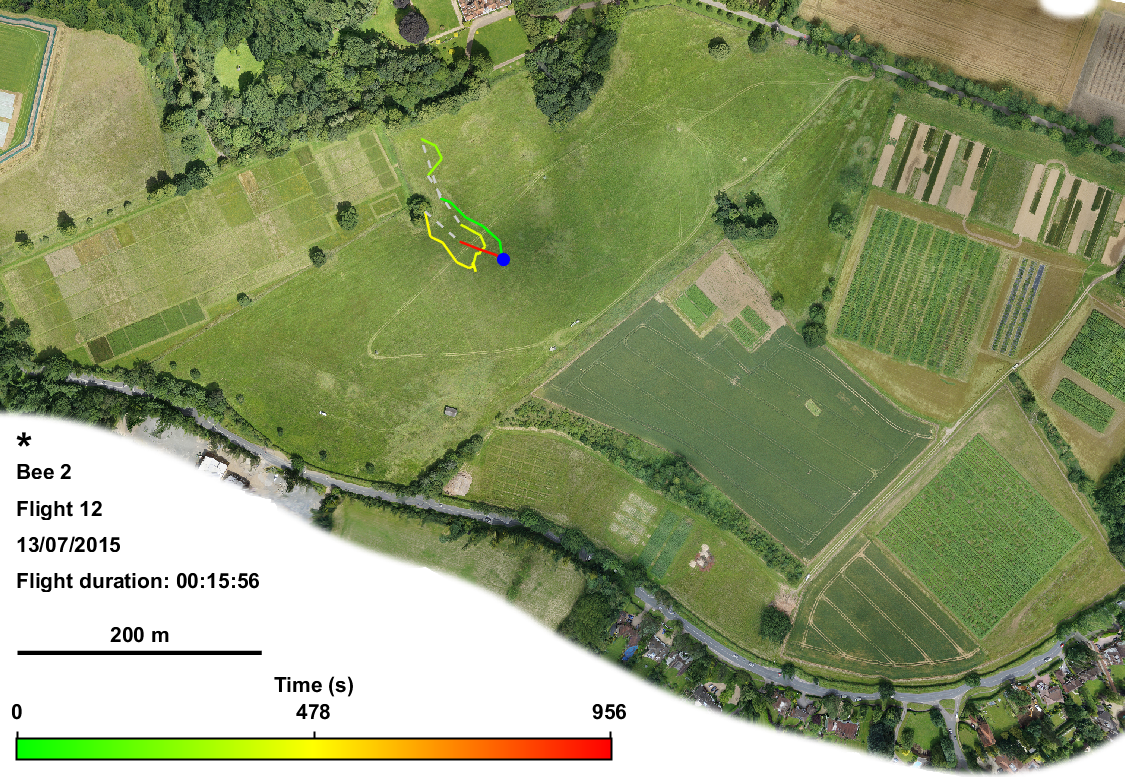

Supplement: S5 File — All flights made by Bee 2 during the period 11/07/15–16/07/15. Each figure represents a single flight. Bee ID, flight number, date of recording and the duration of each flight are shown in the bottom left corner of each figure. The position of the nest is marked by a blue circle. Colours represent the time from the start of each flight: initial portion of each flight is in green, changing smoothly through yellow until the end of the flight is shown in red. Grey dashed lines are used to join radar observations made more than 30 s apart, when the bee’s location was uncertain. (ZIP) [file pone.0160333.s007.zip › S169 Fig.tif]

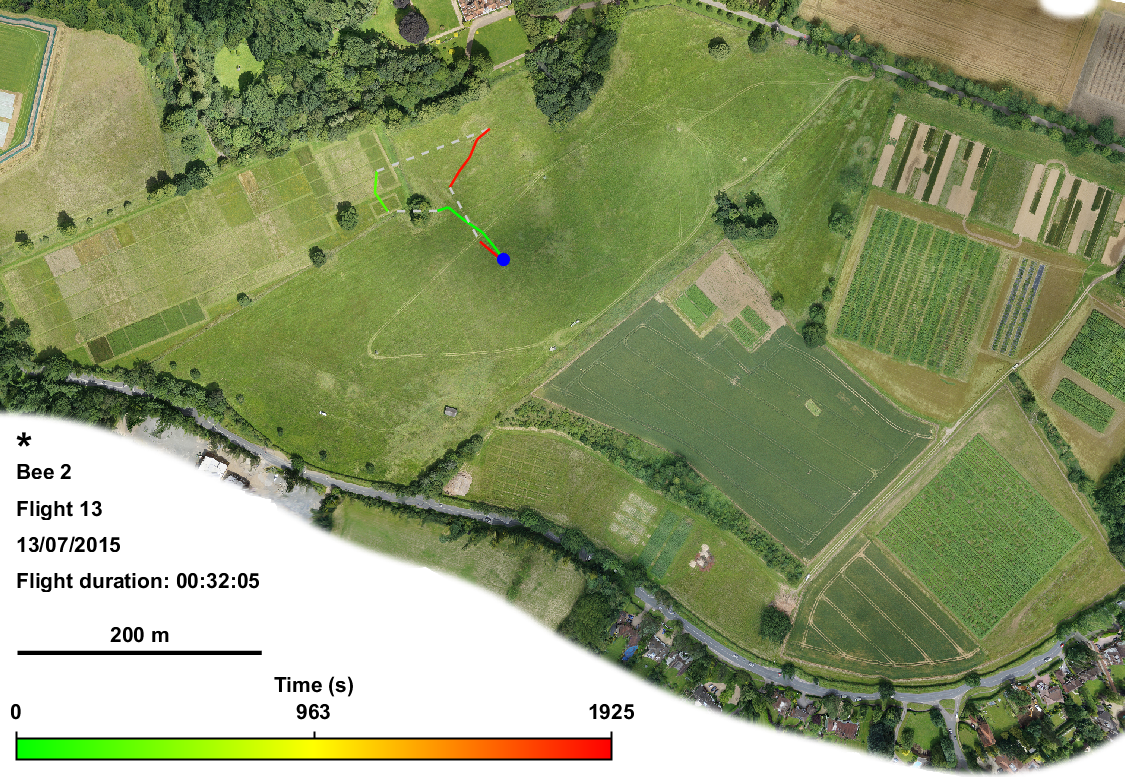

Supplement: S5 File — All flights made by Bee 2 during the period 11/07/15–16/07/15. Each figure represents a single flight. Bee ID, flight number, date of recording and the duration of each flight are shown in the bottom left corner of each figure. The position of the nest is marked by a blue circle. Colours represent the time from the start of each flight: initial portion of each flight is in green, changing smoothly through yellow until the end of the flight is shown in red. Grey dashed lines are used to join radar observations made more than 30 s apart, when the bee’s location was uncertain. (ZIP) [file pone.0160333.s007.zip › S170 Fig.tif]

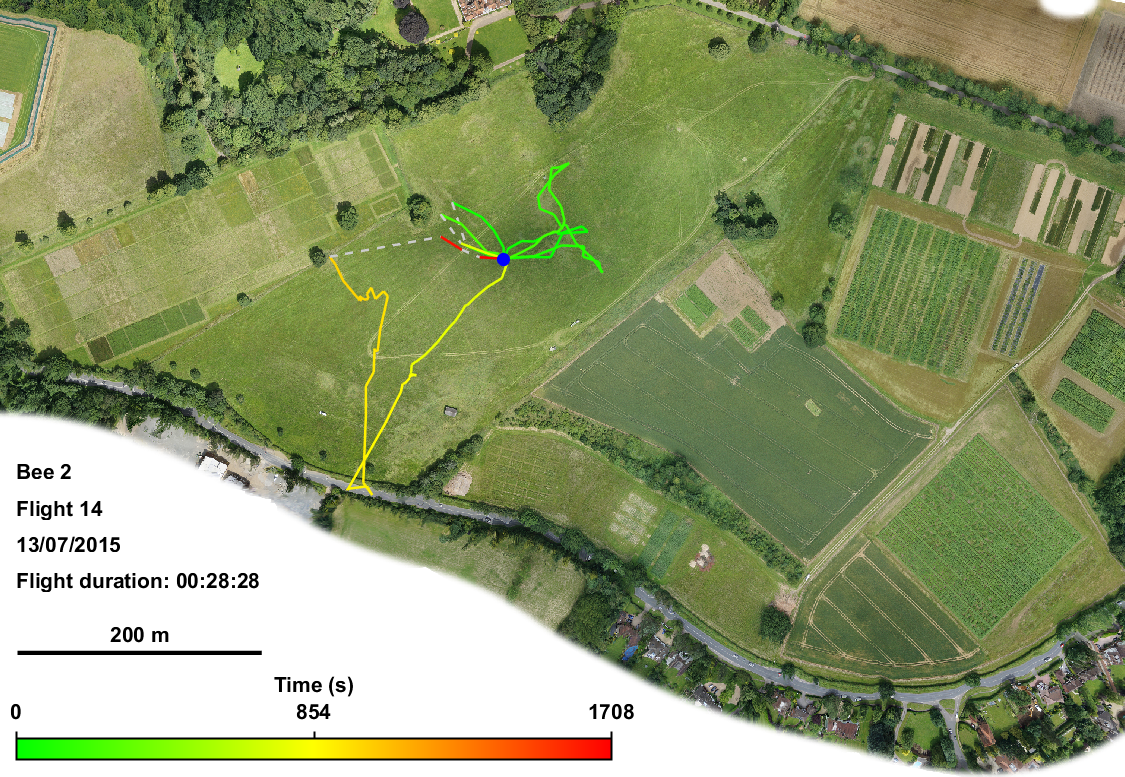

Supplement: S5 File — All flights made by Bee 2 during the period 11/07/15–16/07/15. Each figure represents a single flight. Bee ID, flight number, date of recording and the duration of each flight are shown in the bottom left corner of each figure. The position of the nest is marked by a blue circle. Colours represent the time from the start of each flight: initial portion of each flight is in green, changing smoothly through yellow until the end of the flight is shown in red. Grey dashed lines are used to join radar observations made more than 30 s apart, when the bee’s location was uncertain. (ZIP) [file pone.0160333.s007.zip › S171 Fig.tif]

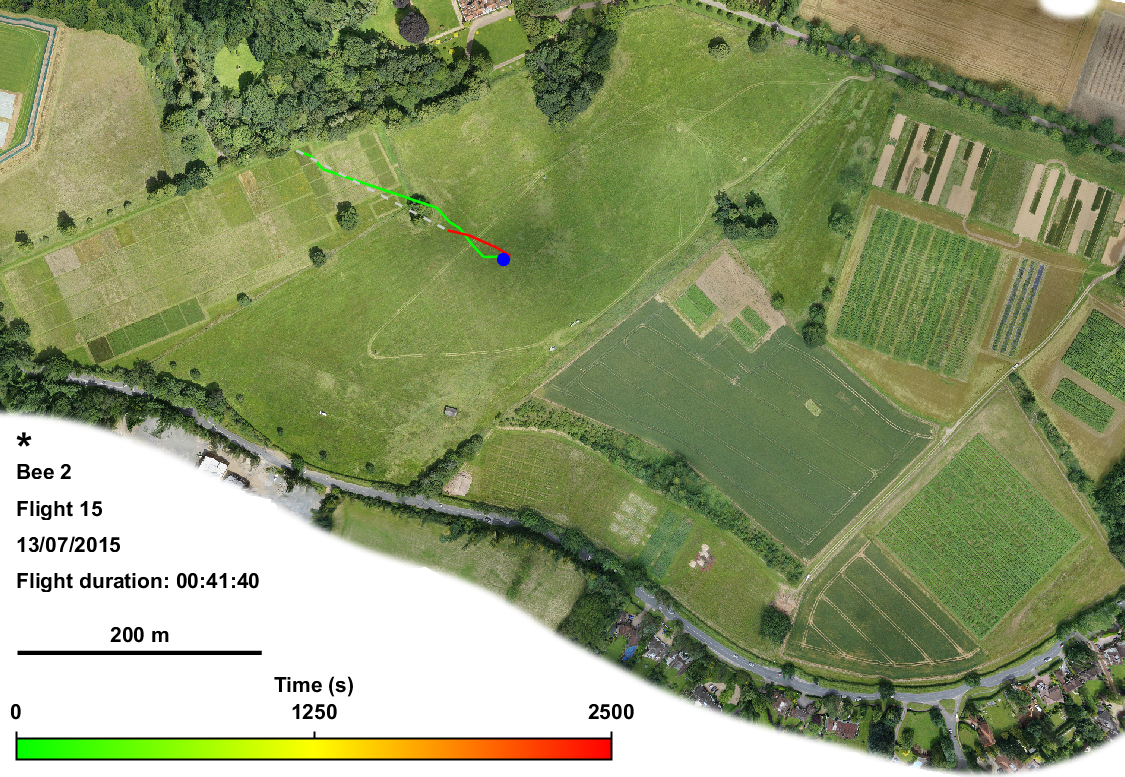

Supplement: S5 File — All flights made by Bee 2 during the period 11/07/15–16/07/15. Each figure represents a single flight. Bee ID, flight number, date of recording and the duration of each flight are shown in the bottom left corner of each figure. The position of the nest is marked by a blue circle. Colours represent the time from the start of each flight: initial portion of each flight is in green, changing smoothly through yellow until the end of the flight is shown in red. Grey dashed lines are used to join radar observations made more than 30 s apart, when the bee’s location was uncertain. (ZIP) [file pone.0160333.s007.zip › S172 Fig.tif]

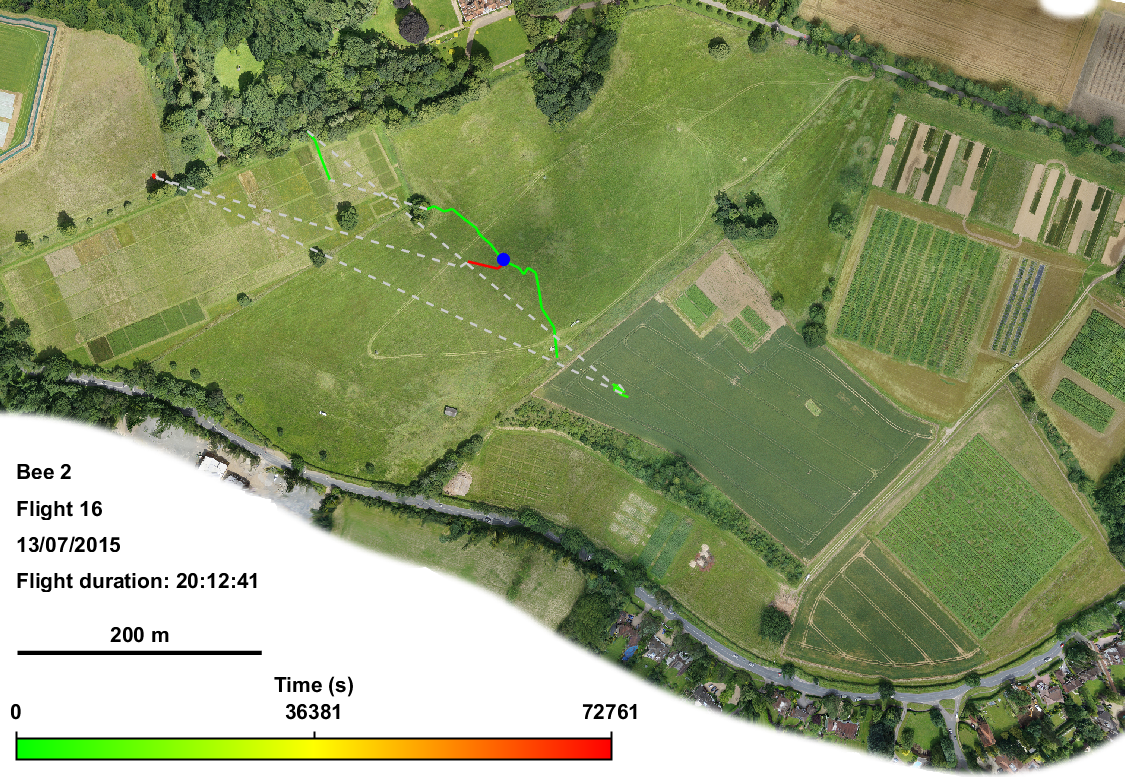

Supplement: S5 File — All flights made by Bee 2 during the period 11/07/15–16/07/15. Each figure represents a single flight. Bee ID, flight number, date of recording and the duration of each flight are shown in the bottom left corner of each figure. The position of the nest is marked by a blue circle. Colours represent the time from the start of each flight: initial portion of each flight is in green, changing smoothly through yellow until the end of the flight is shown in red. Grey dashed lines are used to join radar observations made more than 30 s apart, when the bee’s location was uncertain. (ZIP) [file pone.0160333.s007.zip › S173 Fig.tif]

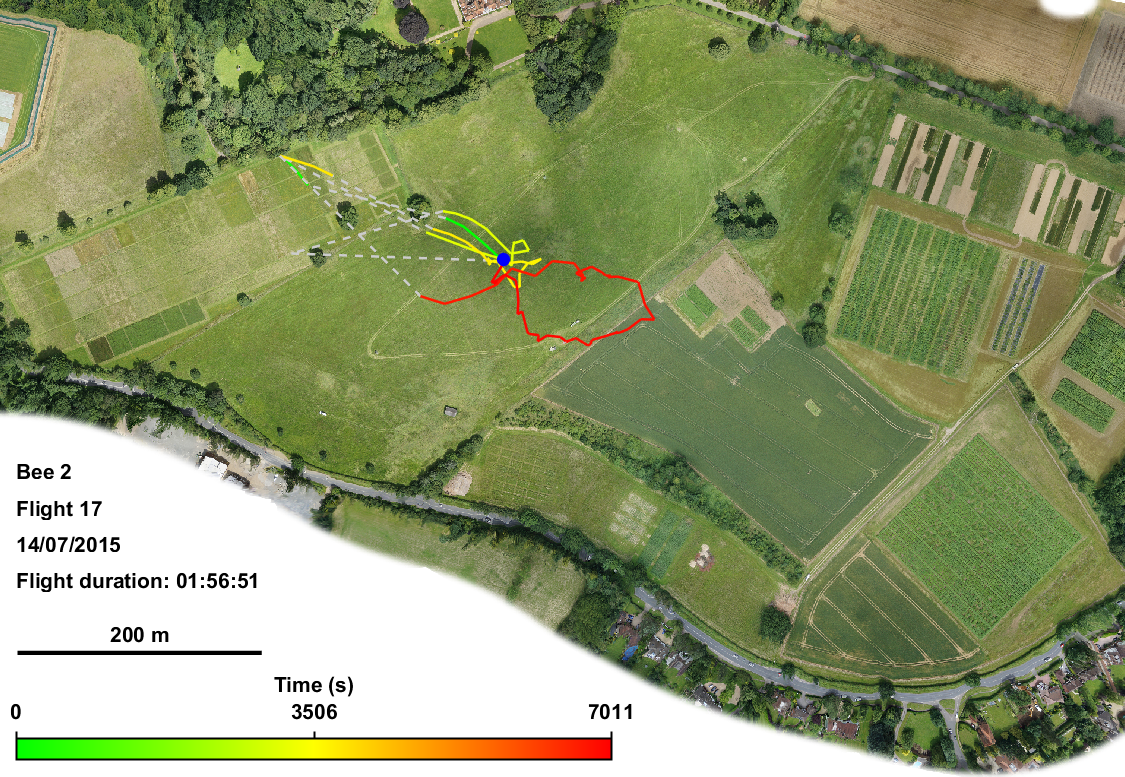

Supplement: S5 File — All flights made by Bee 2 during the period 11/07/15–16/07/15. Each figure represents a single flight. Bee ID, flight number, date of recording and the duration of each flight are shown in the bottom left corner of each figure. The position of the nest is marked by a blue circle. Colours represent the time from the start of each flight: initial portion of each flight is in green, changing smoothly through yellow until the end of the flight is shown in red. Grey dashed lines are used to join radar observations made more than 30 s apart, when the bee’s location was uncertain. (ZIP) [file pone.0160333.s007.zip › S174 Fig.tif]

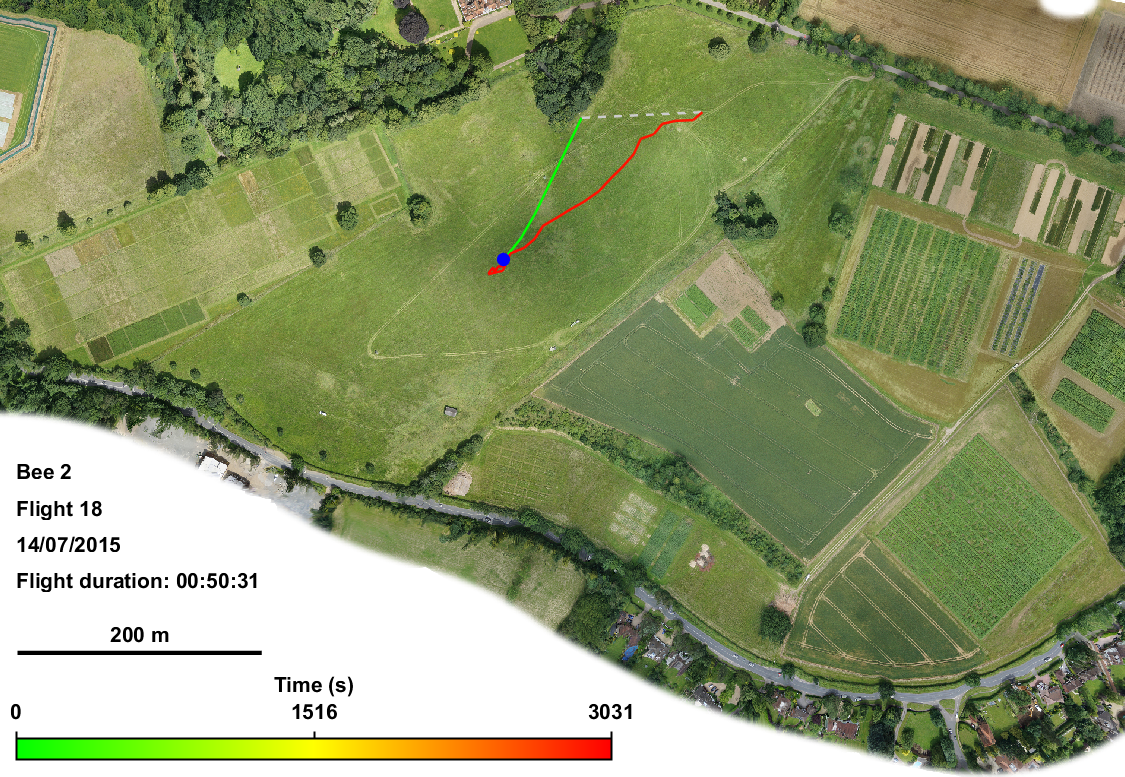

Supplement: S5 File — All flights made by Bee 2 during the period 11/07/15–16/07/15. Each figure represents a single flight. Bee ID, flight number, date of recording and the duration of each flight are shown in the bottom left corner of each figure. The position of the nest is marked by a blue circle. Colours represent the time from the start of each flight: initial portion of each flight is in green, changing smoothly through yellow until the end of the flight is shown in red. Grey dashed lines are used to join radar observations made more than 30 s apart, when the bee’s location was uncertain. (ZIP) [file pone.0160333.s007.zip › S175 Fig.tif]

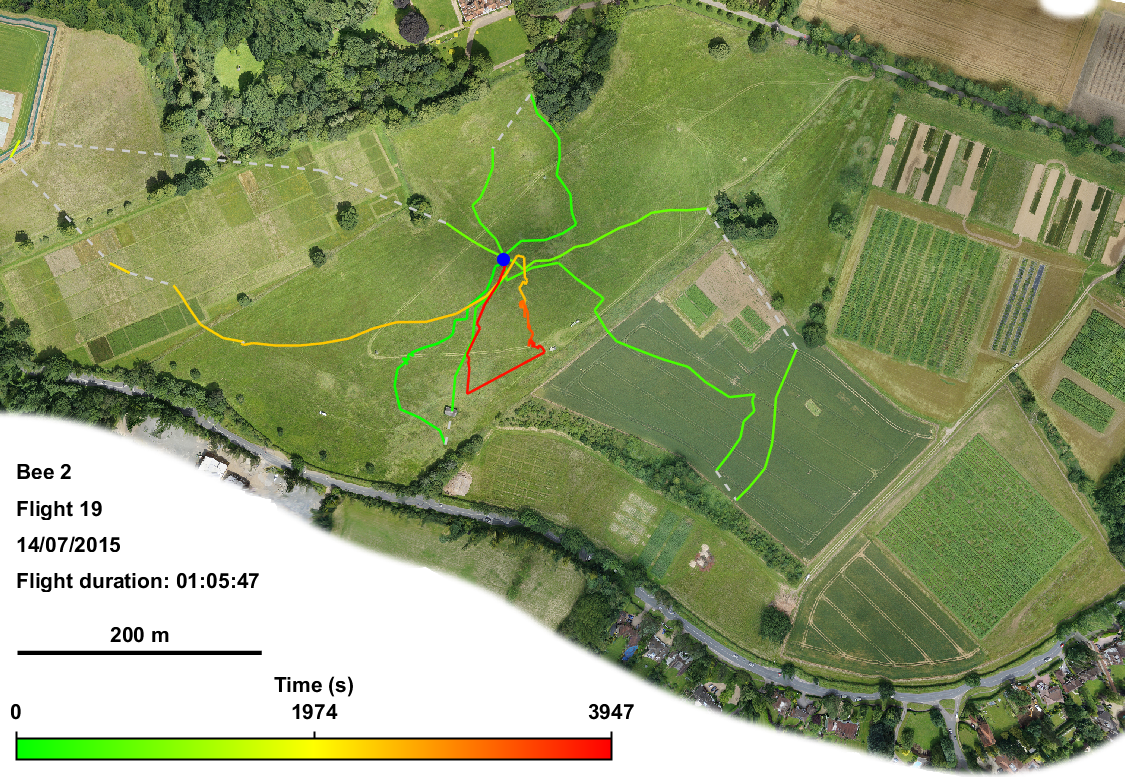

Supplement: S5 File — All flights made by Bee 2 during the period 11/07/15–16/07/15. Each figure represents a single flight. Bee ID, flight number, date of recording and the duration of each flight are shown in the bottom left corner of each figure. The position of the nest is marked by a blue circle. Colours represent the time from the start of each flight: initial portion of each flight is in green, changing smoothly through yellow until the end of the flight is shown in red. Grey dashed lines are used to join radar observations made more than 30 s apart, when the bee’s location was uncertain. (ZIP) [file pone.0160333.s007.zip › S176 Fig.tif]

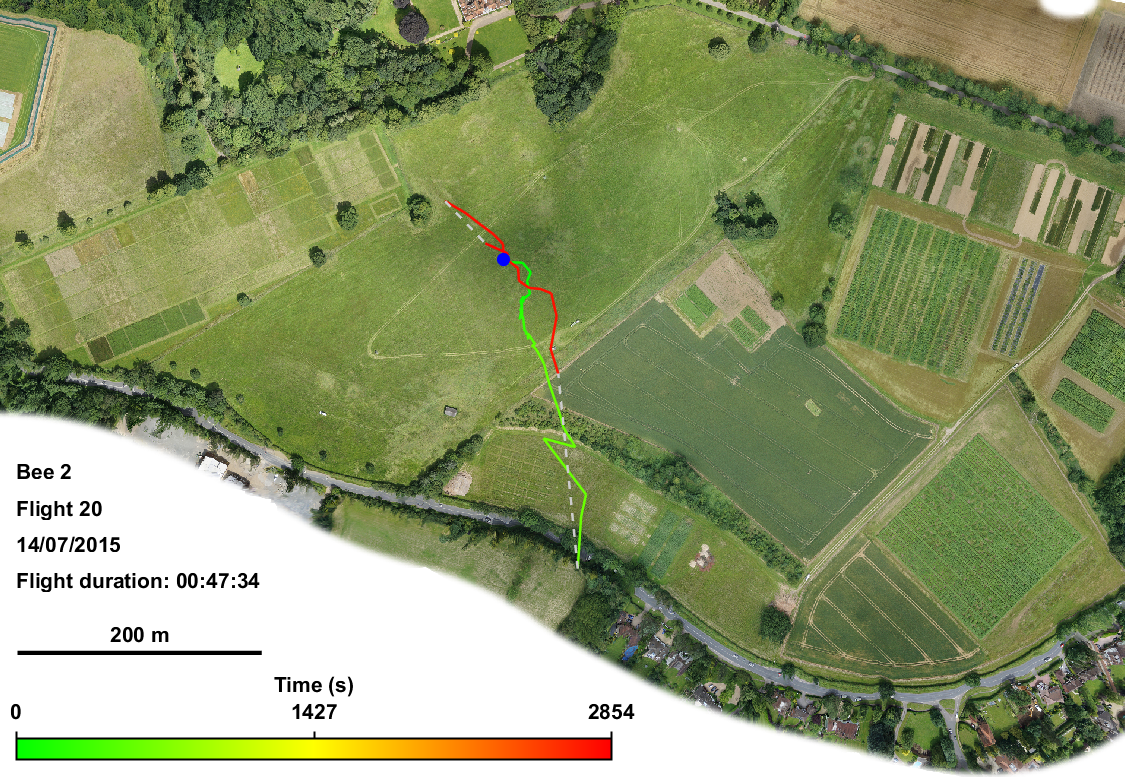

Supplement: S5 File — All flights made by Bee 2 during the period 11/07/15–16/07/15. Each figure represents a single flight. Bee ID, flight number, date of recording and the duration of each flight are shown in the bottom left corner of each figure. The position of the nest is marked by a blue circle. Colours represent the time from the start of each flight: initial portion of each flight is in green, changing smoothly through yellow until the end of the flight is shown in red. Grey dashed lines are used to join radar observations made more than 30 s apart, when the bee’s location was uncertain. (ZIP) [file pone.0160333.s007.zip › S177 Fig.tif]

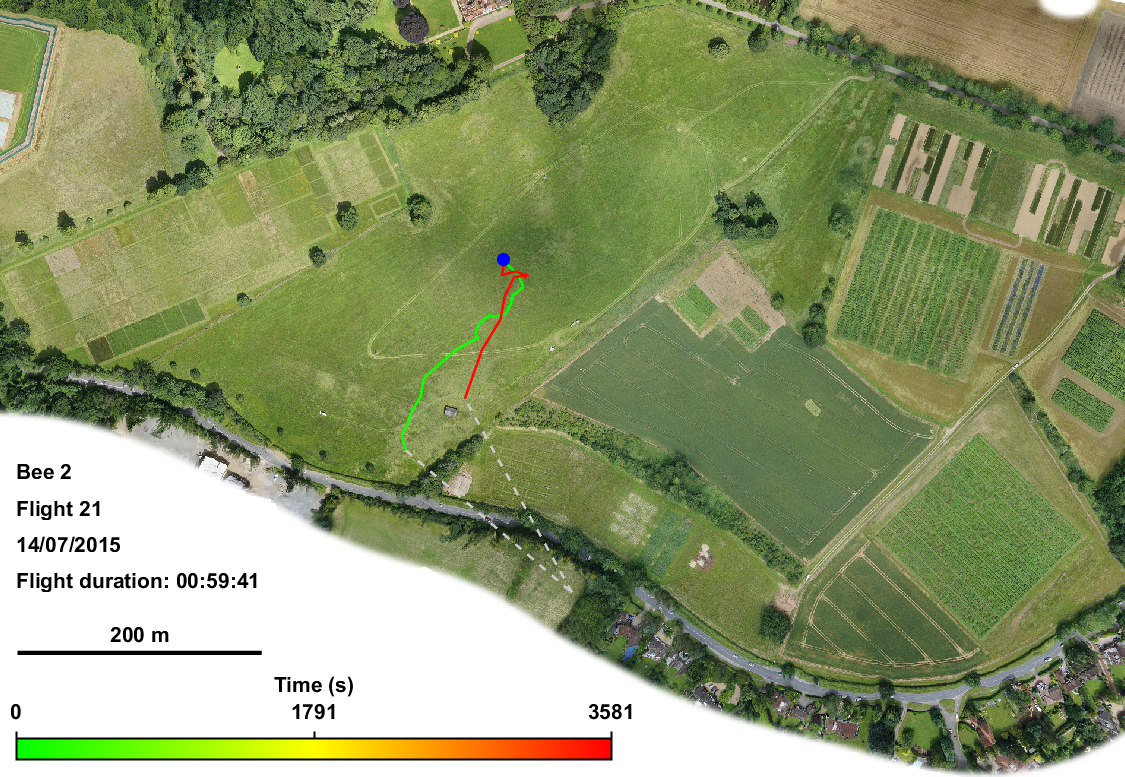

Supplement: S5 File — All flights made by Bee 2 during the period 11/07/15–16/07/15. Each figure represents a single flight. Bee ID, flight number, date of recording and the duration of each flight are shown in the bottom left corner of each figure. The position of the nest is marked by a blue circle. Colours represent the time from the start of each flight: initial portion of each flight is in green, changing smoothly through yellow until the end of the flight is shown in red. Grey dashed lines are used to join radar observations made more than 30 s apart, when the bee’s location was uncertain. (ZIP) [file pone.0160333.s007.zip › S178 Fig.tif]

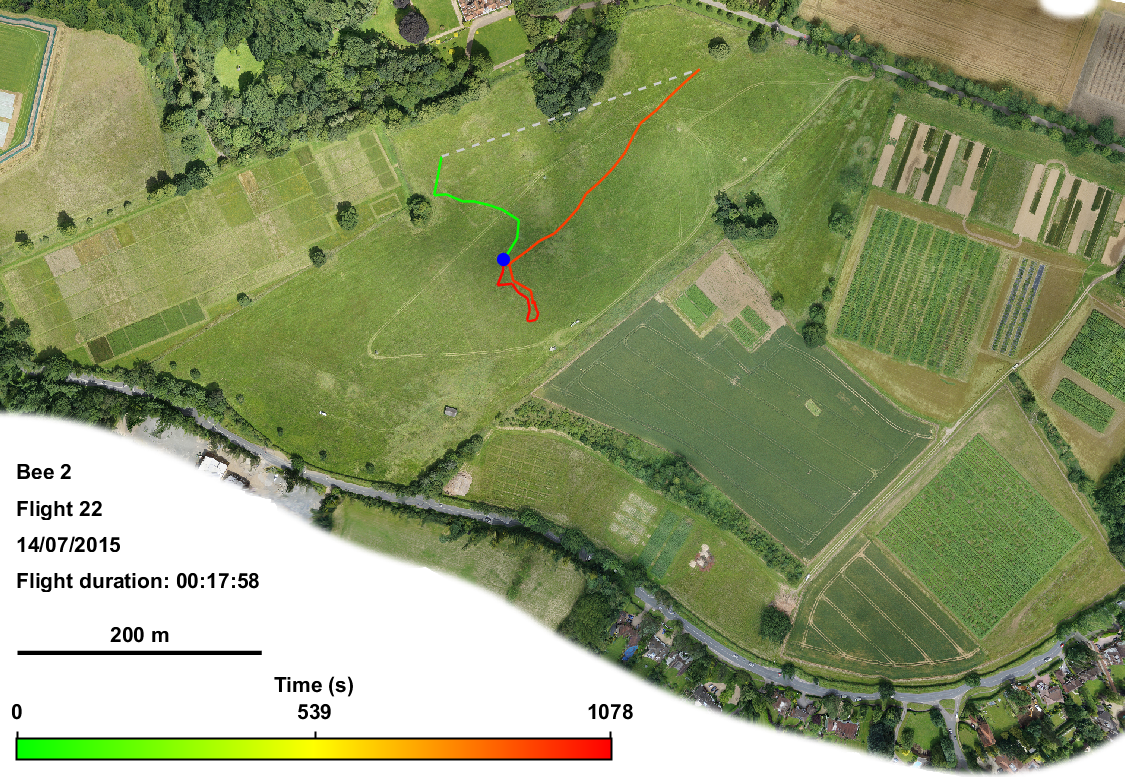

Supplement: S5 File — All flights made by Bee 2 during the period 11/07/15–16/07/15. Each figure represents a single flight. Bee ID, flight number, date of recording and the duration of each flight are shown in the bottom left corner of each figure. The position of the nest is marked by a blue circle. Colours represent the time from the start of each flight: initial portion of each flight is in green, changing smoothly through yellow until the end of the flight is shown in red. Grey dashed lines are used to join radar observations made more than 30 s apart, when the bee’s location was uncertain. (ZIP) [file pone.0160333.s007.zip › S179 Fig.tif]

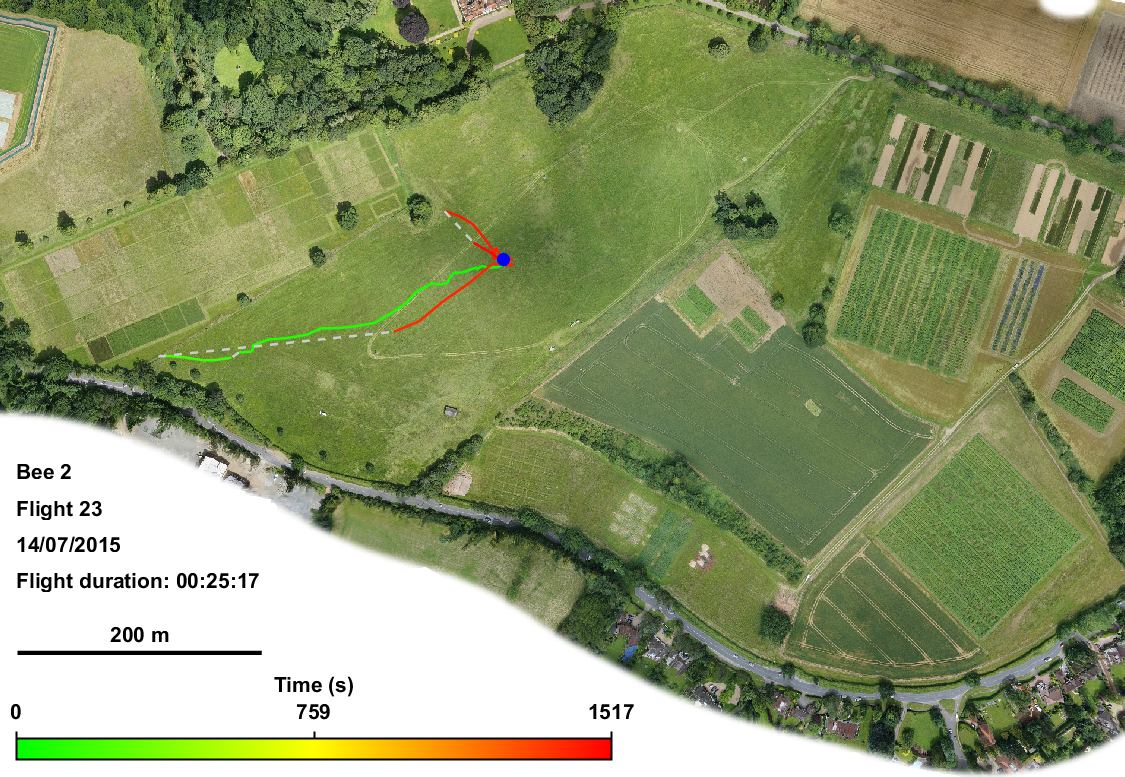

Supplement: S5 File — All flights made by Bee 2 during the period 11/07/15–16/07/15. Each figure represents a single flight. Bee ID, flight number, date of recording and the duration of each flight are shown in the bottom left corner of each figure. The position of the nest is marked by a blue circle. Colours represent the time from the start of each flight: initial portion of each flight is in green, changing smoothly through yellow until the end of the flight is shown in red. Grey dashed lines are used to join radar observations made more than 30 s apart, when the bee’s location was uncertain. (ZIP) [file pone.0160333.s007.zip › S180 Fig.tif]

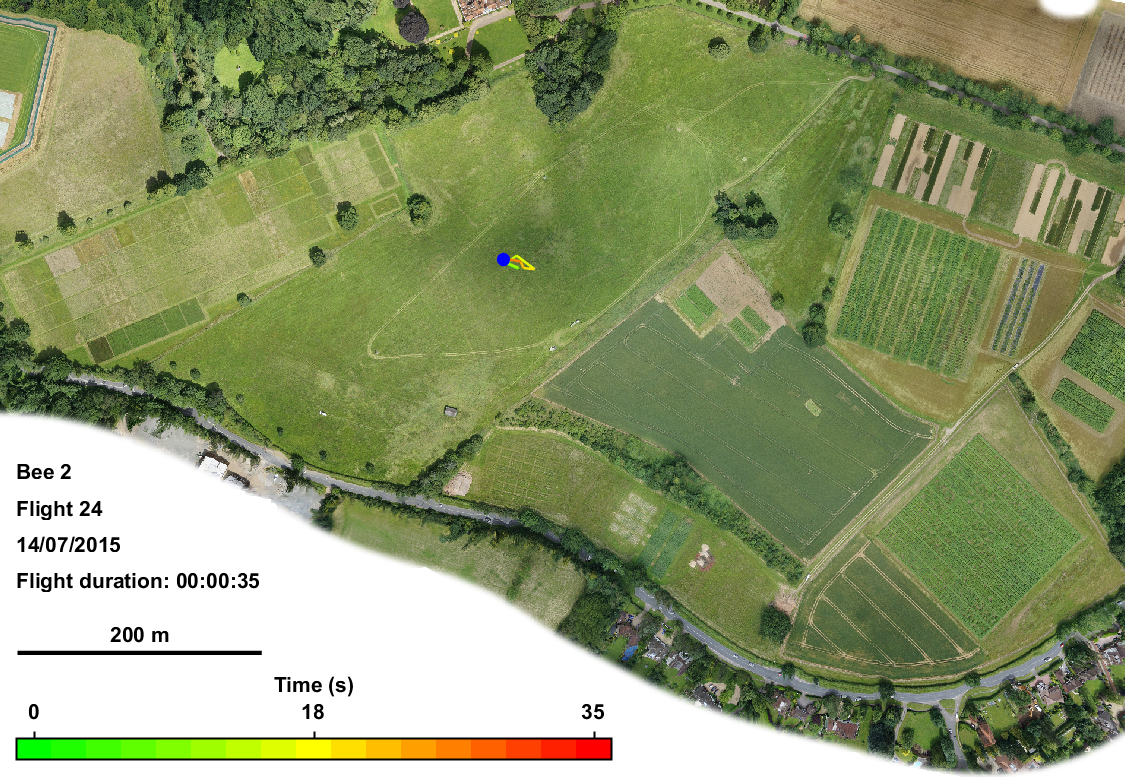

Supplement: S5 File — All flights made by Bee 2 during the period 11/07/15–16/07/15. Each figure represents a single flight. Bee ID, flight number, date of recording and the duration of each flight are shown in the bottom left corner of each figure. The position of the nest is marked by a blue circle. Colours represent the time from the start of each flight: initial portion of each flight is in green, changing smoothly through yellow until the end of the flight is shown in red. Grey dashed lines are used to join radar observations made more than 30 s apart, when the bee’s location was uncertain. (ZIP) [file pone.0160333.s007.zip › S181 Fig.tif]

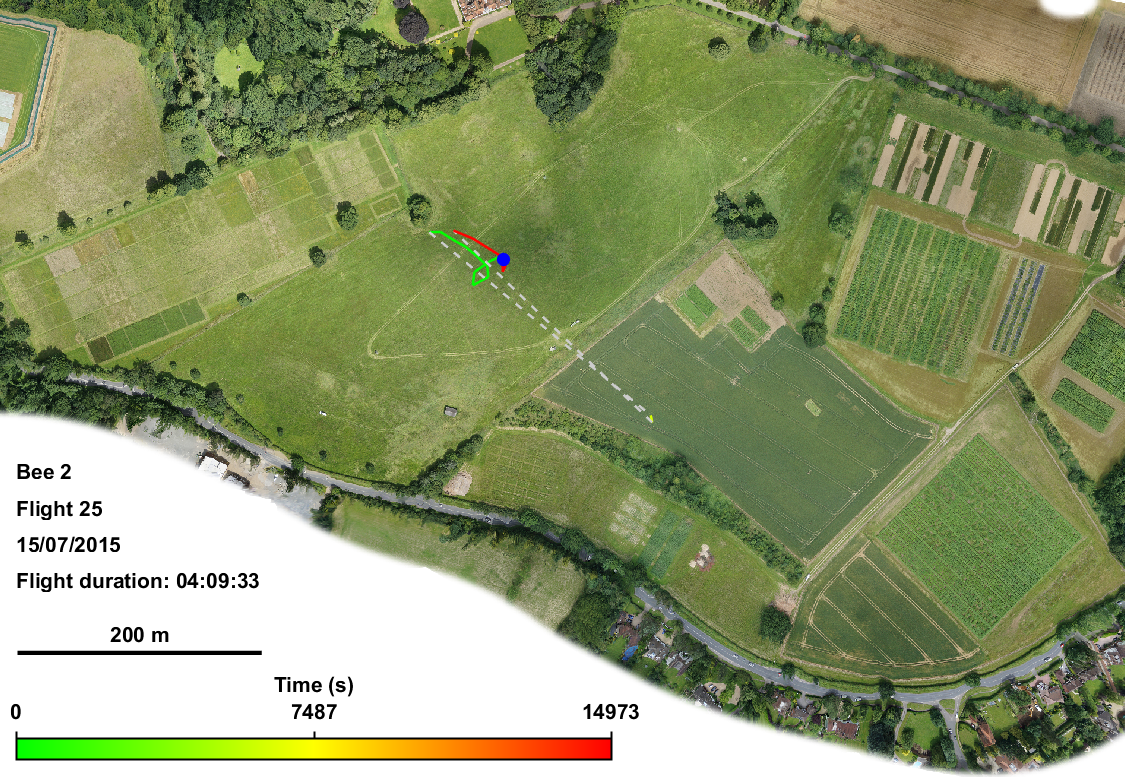

Supplement: S5 File — All flights made by Bee 2 during the period 11/07/15–16/07/15. Each figure represents a single flight. Bee ID, flight number, date of recording and the duration of each flight are shown in the bottom left corner of each figure. The position of the nest is marked by a blue circle. Colours represent the time from the start of each flight: initial portion of each flight is in green, changing smoothly through yellow until the end of the flight is shown in red. Grey dashed lines are used to join radar observations made more than 30 s apart, when the bee’s location was uncertain. (ZIP) [file pone.0160333.s007.zip › S182 Fig.tif]

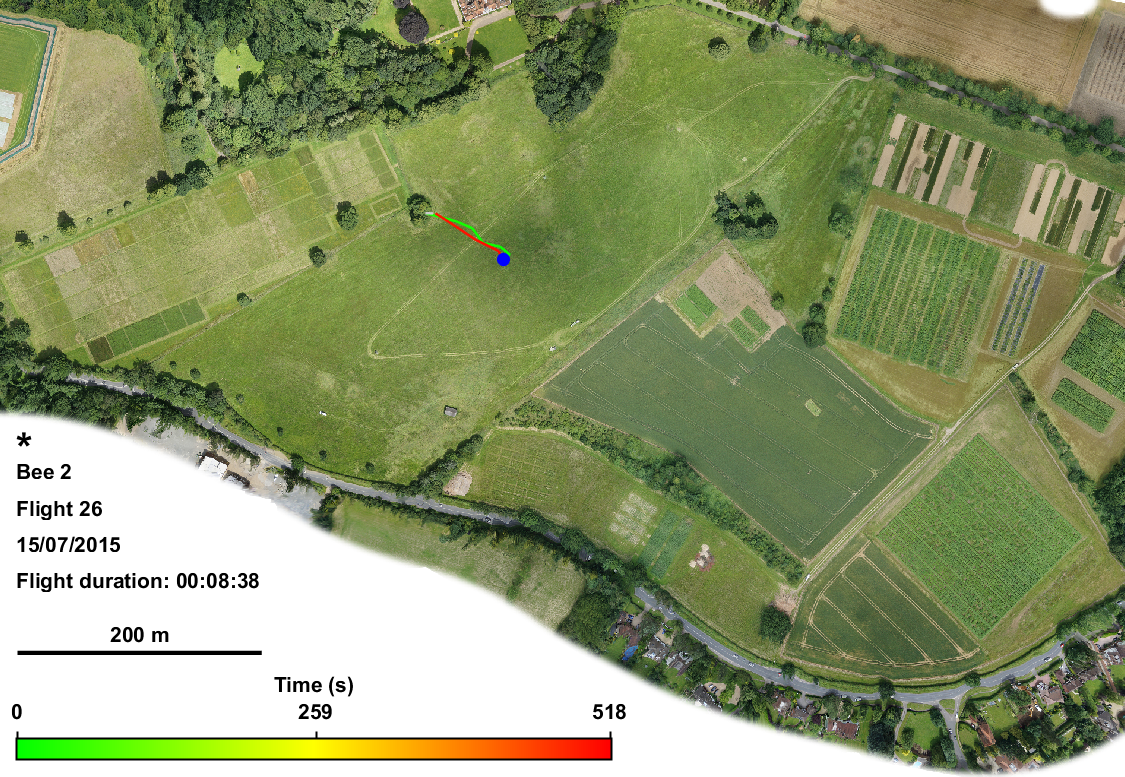

Supplement: S5 File — All flights made by Bee 2 during the period 11/07/15–16/07/15. Each figure represents a single flight. Bee ID, flight number, date of recording and the duration of each flight are shown in the bottom left corner of each figure. The position of the nest is marked by a blue circle. Colours represent the time from the start of each flight: initial portion of each flight is in green, changing smoothly through yellow until the end of the flight is shown in red. Grey dashed lines are used to join radar observations made more than 30 s apart, when the bee’s location was uncertain. (ZIP) [file pone.0160333.s007.zip › S183 Fig.tif]

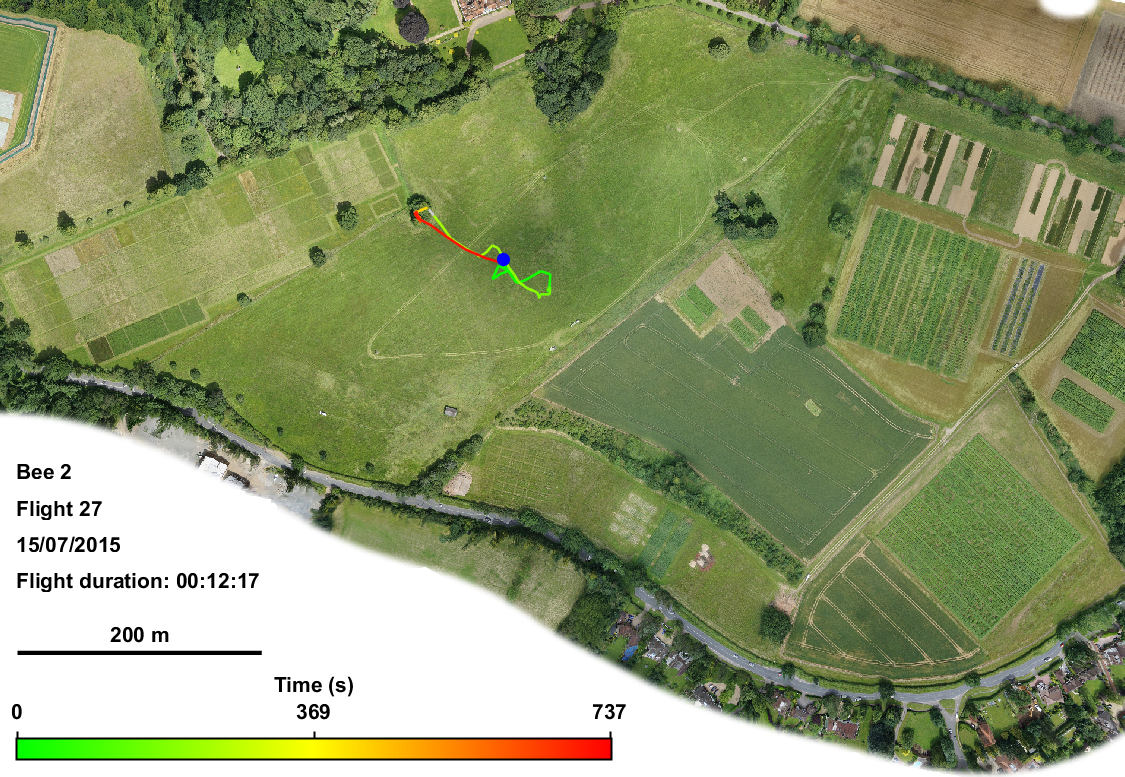

Supplement: S5 File — All flights made by Bee 2 during the period 11/07/15–16/07/15. Each figure represents a single flight. Bee ID, flight number, date of recording and the duration of each flight are shown in the bottom left corner of each figure. The position of the nest is marked by a blue circle. Colours represent the time from the start of each flight: initial portion of each flight is in green, changing smoothly through yellow until the end of the flight is shown in red. Grey dashed lines are used to join radar observations made more than 30 s apart, when the bee’s location was uncertain. (ZIP) [file pone.0160333.s007.zip › S184 Fig.tif]

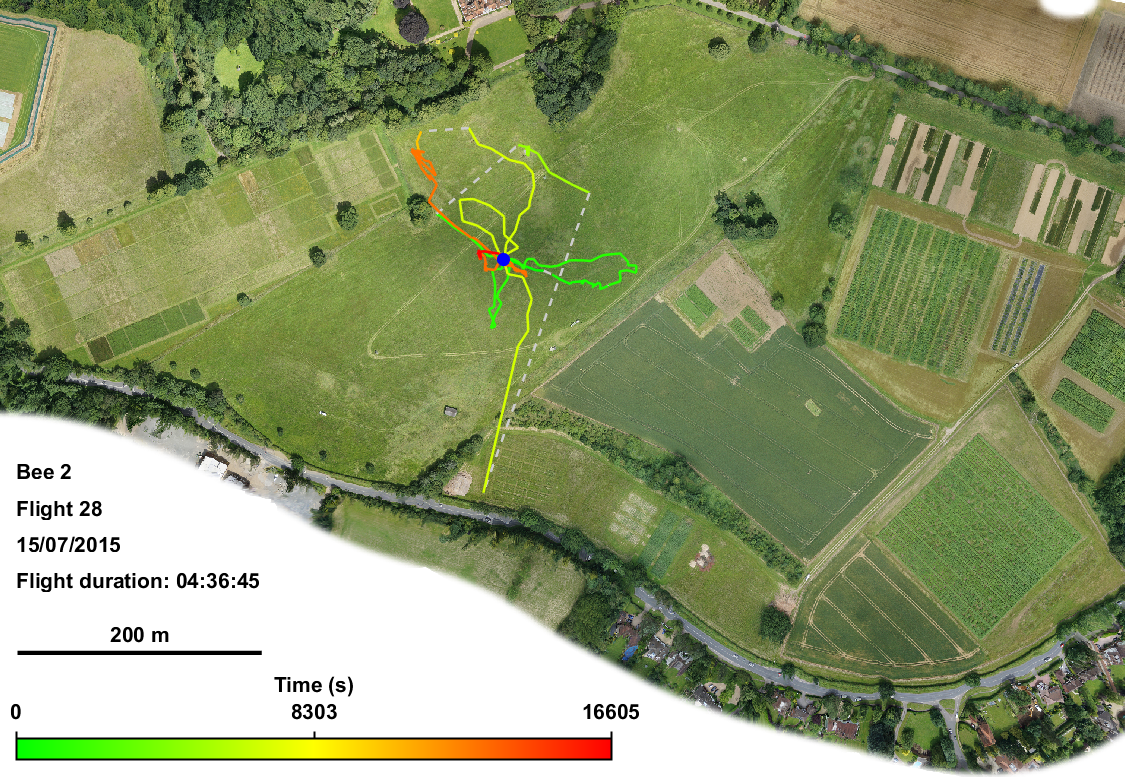

Supplement: S5 File — All flights made by Bee 2 during the period 11/07/15–16/07/15. Each figure represents a single flight. Bee ID, flight number, date of recording and the duration of each flight are shown in the bottom left corner of each figure. The position of the nest is marked by a blue circle. Colours represent the time from the start of each flight: initial portion of each flight is in green, changing smoothly through yellow until the end of the flight is shown in red. Grey dashed lines are used to join radar observations made more than 30 s apart, when the bee’s location was uncertain. (ZIP) [file pone.0160333.s007.zip › S185 Fig.tif]

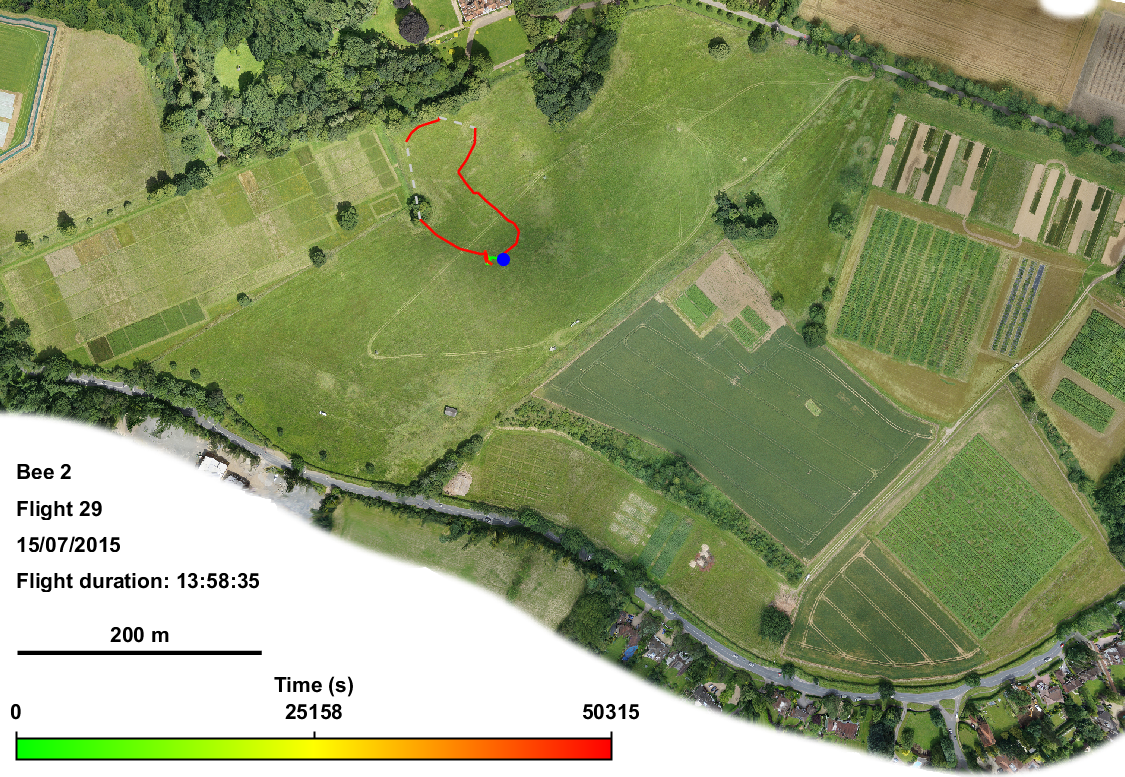

Supplement: S5 File — All flights made by Bee 2 during the period 11/07/15–16/07/15. Each figure represents a single flight. Bee ID, flight number, date of recording and the duration of each flight are shown in the bottom left corner of each figure. The position of the nest is marked by a blue circle. Colours represent the time from the start of each flight: initial portion of each flight is in green, changing smoothly through yellow until the end of the flight is shown in red. Grey dashed lines are used to join radar observations made more than 30 s apart, when the bee’s location was uncertain. (ZIP) [file pone.0160333.s007.zip › S186 Fig.tif]

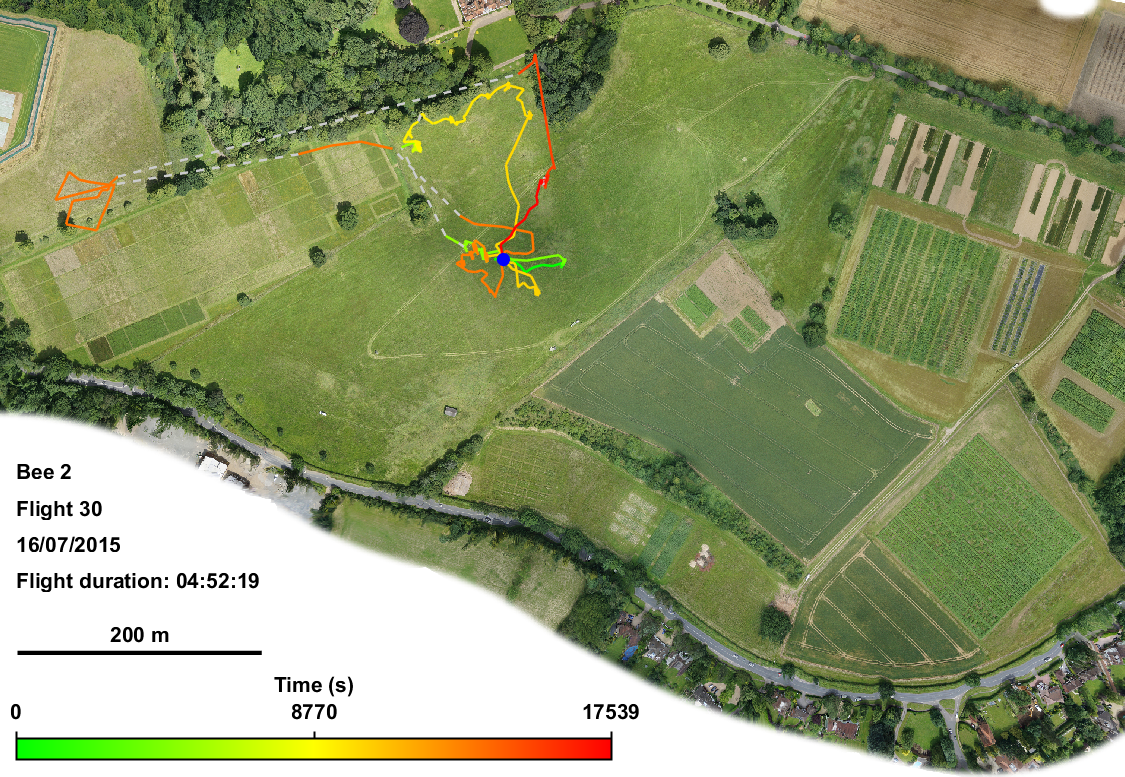

Supplement: S5 File — All flights made by Bee 2 during the period 11/07/15–16/07/15. Each figure represents a single flight. Bee ID, flight number, date of recording and the duration of each flight are shown in the bottom left corner of each figure. The position of the nest is marked by a blue circle. Colours represent the time from the start of each flight: initial portion of each flight is in green, changing smoothly through yellow until the end of the flight is shown in red. Grey dashed lines are used to join radar observations made more than 30 s apart, when the bee’s location was uncertain. (ZIP) [file pone.0160333.s007.zip › S187 Fig.tif]

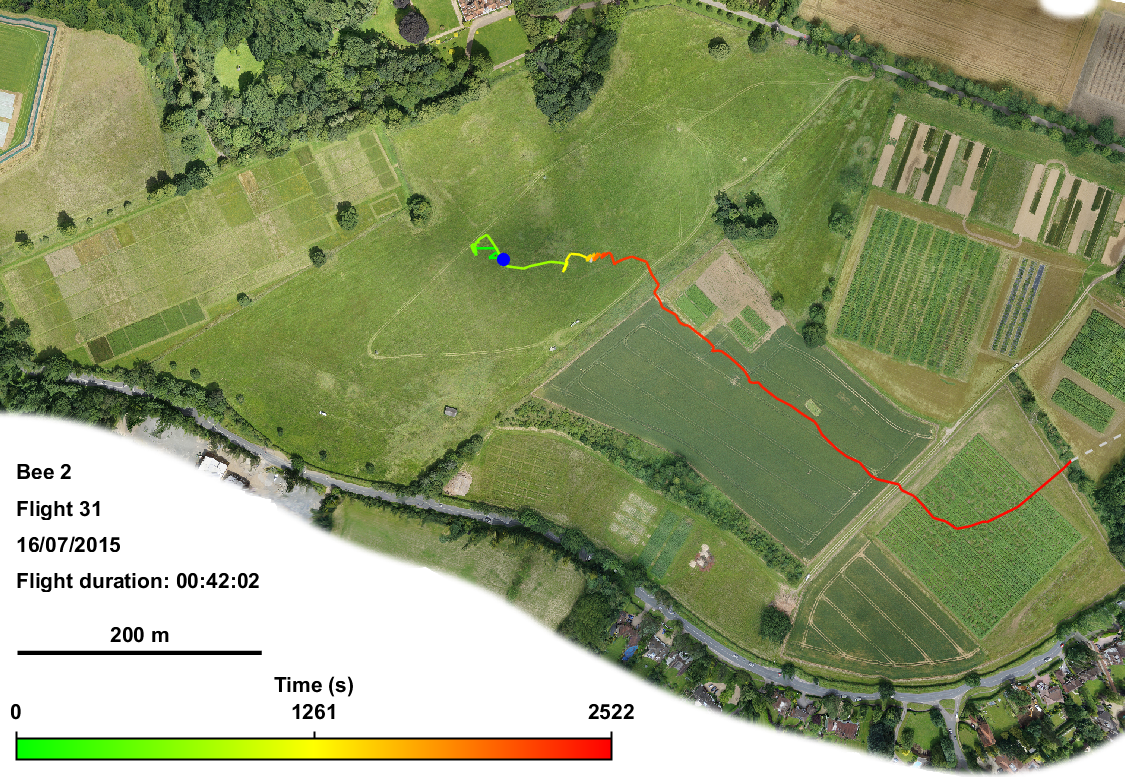

Supplement: S5 File — All flights made by Bee 2 during the period 11/07/15–16/07/15. Each figure represents a single flight. Bee ID, flight number, date of recording and the duration of each flight are shown in the bottom left corner of each figure. The position of the nest is marked by a blue circle. Colours represent the time from the start of each flight: initial portion of each flight is in green, changing smoothly through yellow until the end of the flight is shown in red. Grey dashed lines are used to join radar observations made more than 30 s apart, when the bee’s location was uncertain. (ZIP) [file pone.0160333.s007.zip › S188 Fig.tif]

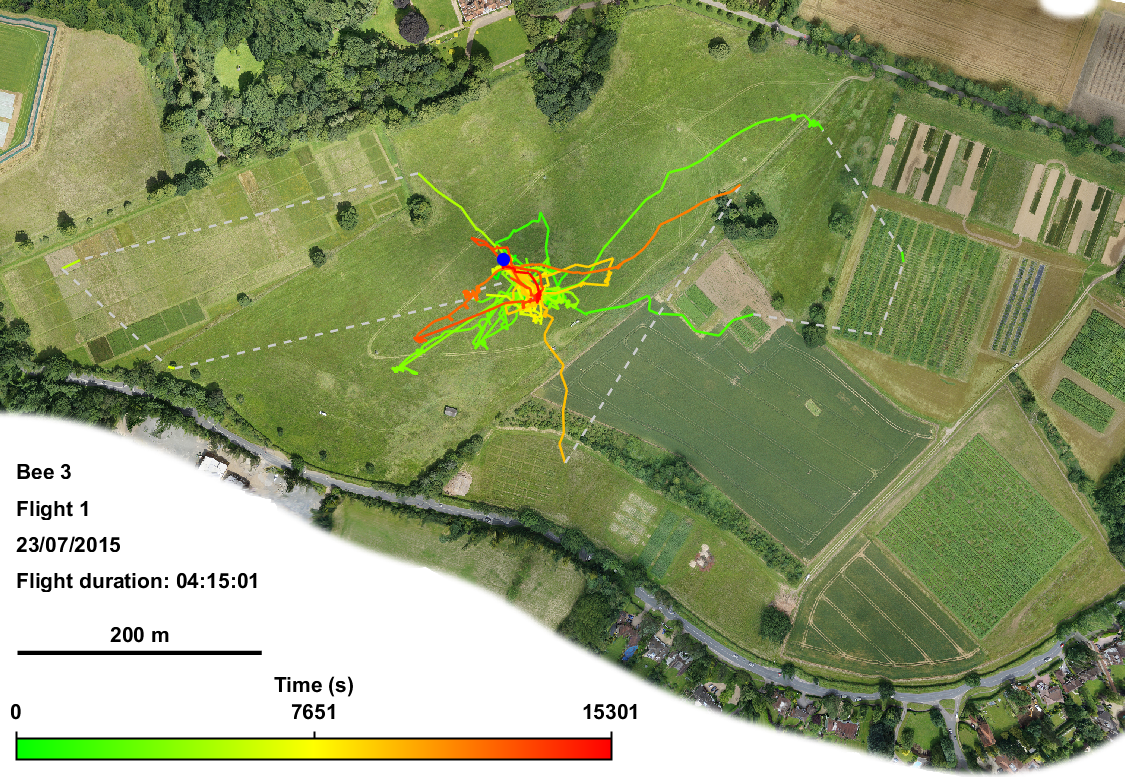

Supplement: S6 File — All flights made by Bee 3 during the period 23/07/15–06/08/15. Each figure represents a single flight. Bee ID, flight number, date of recording and the duration of each flight are shown in the bottom left corner of each figure. The position of the nest is marked by a blue circle. Colours represent the time from the start of each flight: initial portion of each flight is in green, changing smoothly through yellow until the end of the flight is shown in red. Grey dashed lines are used to join radar observations made more than 30 s apart, when the bee’s location was uncertain. (ZIP) [file pone.0160333.s008.zip › S189 Fig.tif]

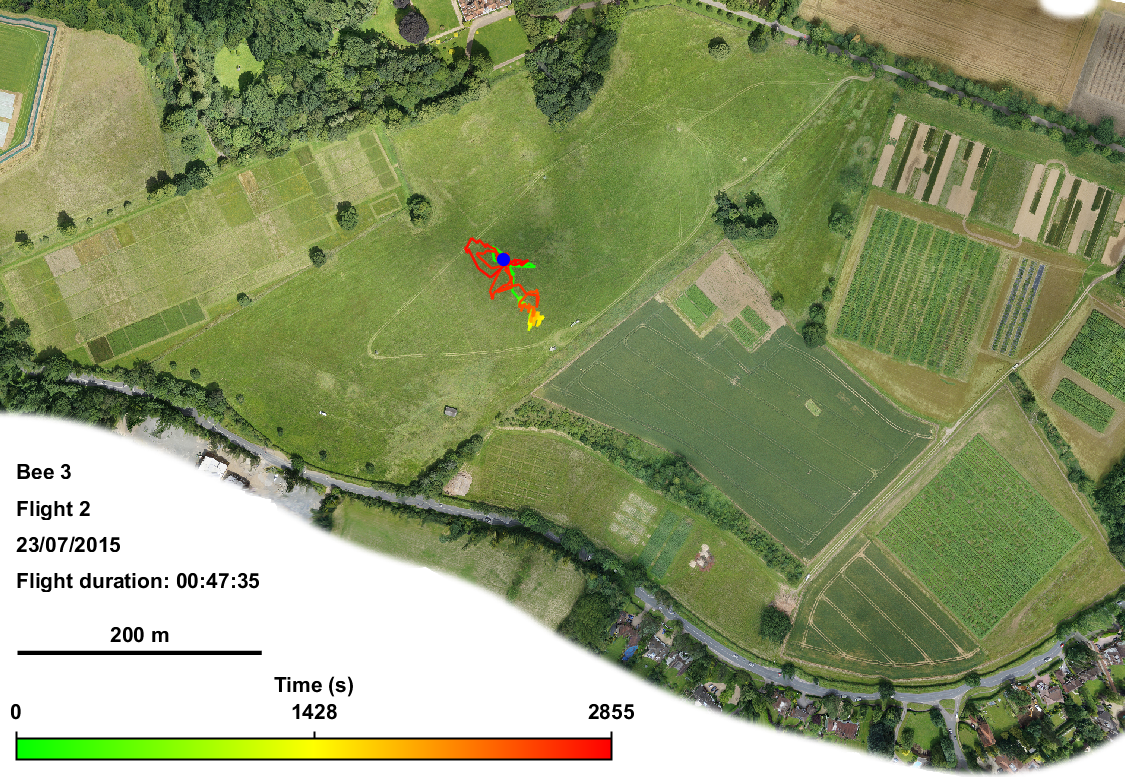

Supplement: S6 File — All flights made by Bee 3 during the period 23/07/15–06/08/15. Each figure represents a single flight. Bee ID, flight number, date of recording and the duration of each flight are shown in the bottom left corner of each figure. The position of the nest is marked by a blue circle. Colours represent the time from the start of each flight: initial portion of each flight is in green, changing smoothly through yellow until the end of the flight is shown in red. Grey dashed lines are used to join radar observations made more than 30 s apart, when the bee’s location was uncertain. (ZIP) [file pone.0160333.s008.zip › S190 Fig.tif]

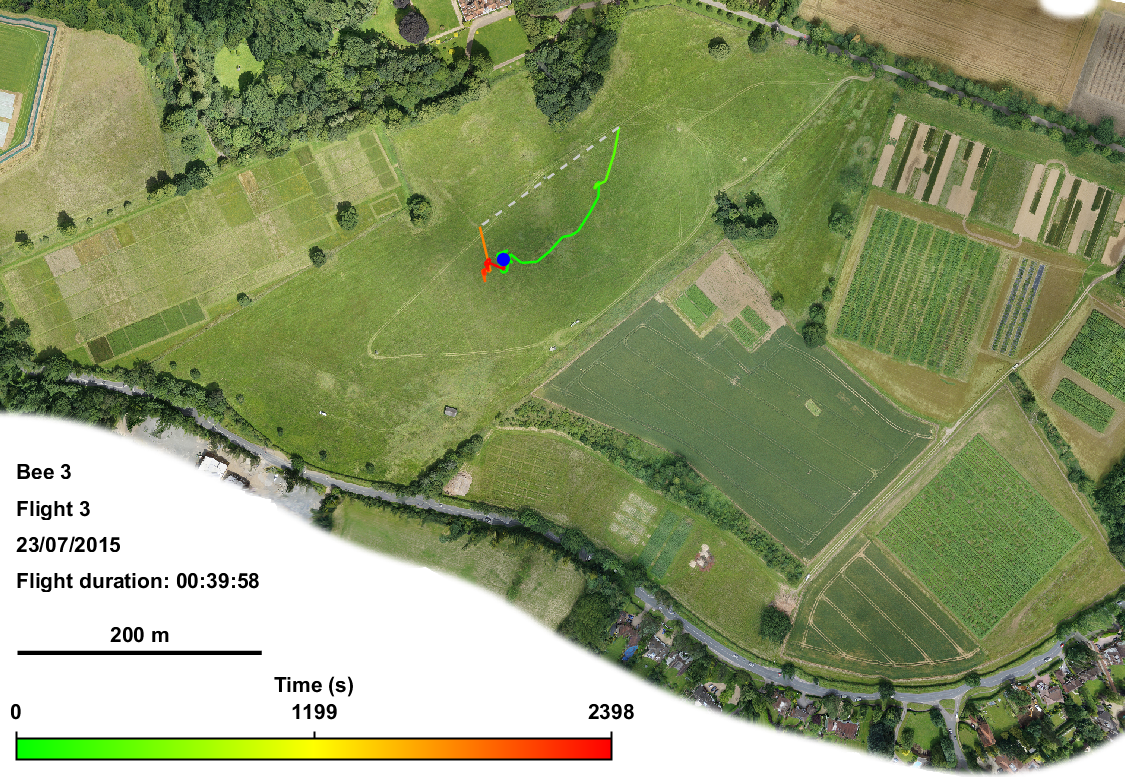

Supplement: S6 File — All flights made by Bee 3 during the period 23/07/15–06/08/15. Each figure represents a single flight. Bee ID, flight number, date of recording and the duration of each flight are shown in the bottom left corner of each figure. The position of the nest is marked by a blue circle. Colours represent the time from the start of each flight: initial portion of each flight is in green, changing smoothly through yellow until the end of the flight is shown in red. Grey dashed lines are used to join radar observations made more than 30 s apart, when the bee’s location was uncertain. (ZIP) [file pone.0160333.s008.zip › S191 Fig.tif]

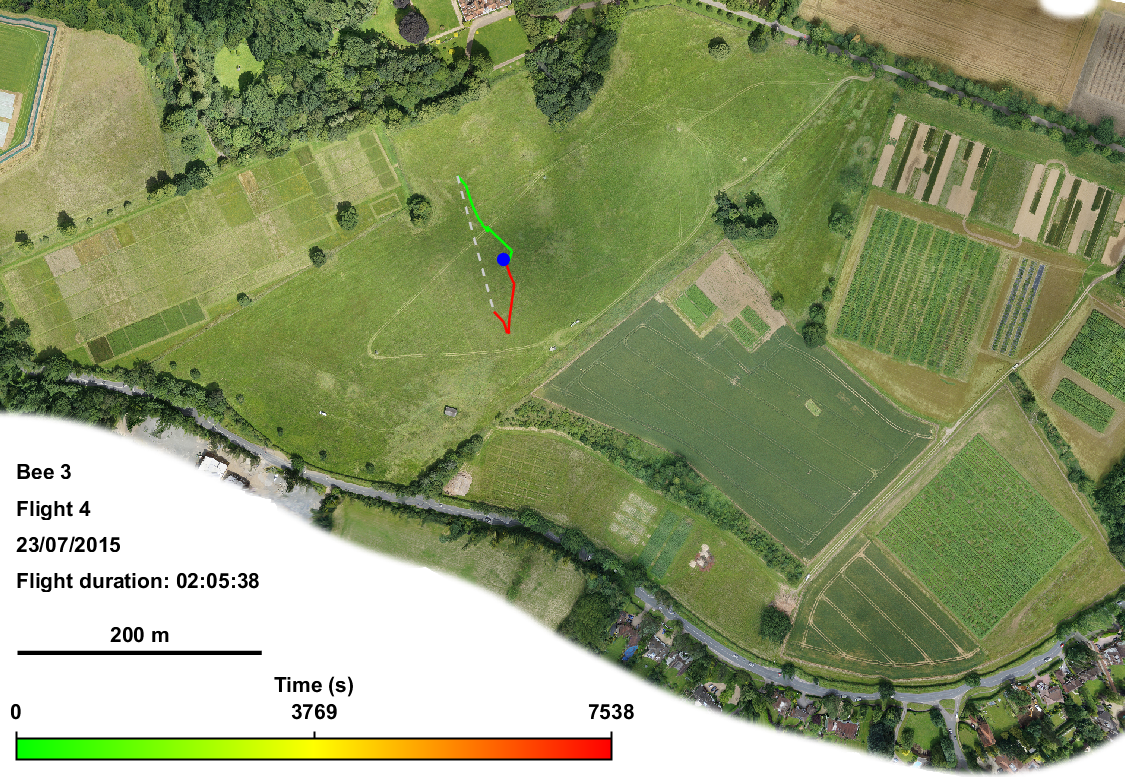

Supplement: S6 File — All flights made by Bee 3 during the period 23/07/15–06/08/15. Each figure represents a single flight. Bee ID, flight number, date of recording and the duration of each flight are shown in the bottom left corner of each figure. The position of the nest is marked by a blue circle. Colours represent the time from the start of each flight: initial portion of each flight is in green, changing smoothly through yellow until the end of the flight is shown in red. Grey dashed lines are used to join radar observations made more than 30 s apart, when the bee’s location was uncertain. (ZIP) [file pone.0160333.s008.zip › S192 Fig.tif]

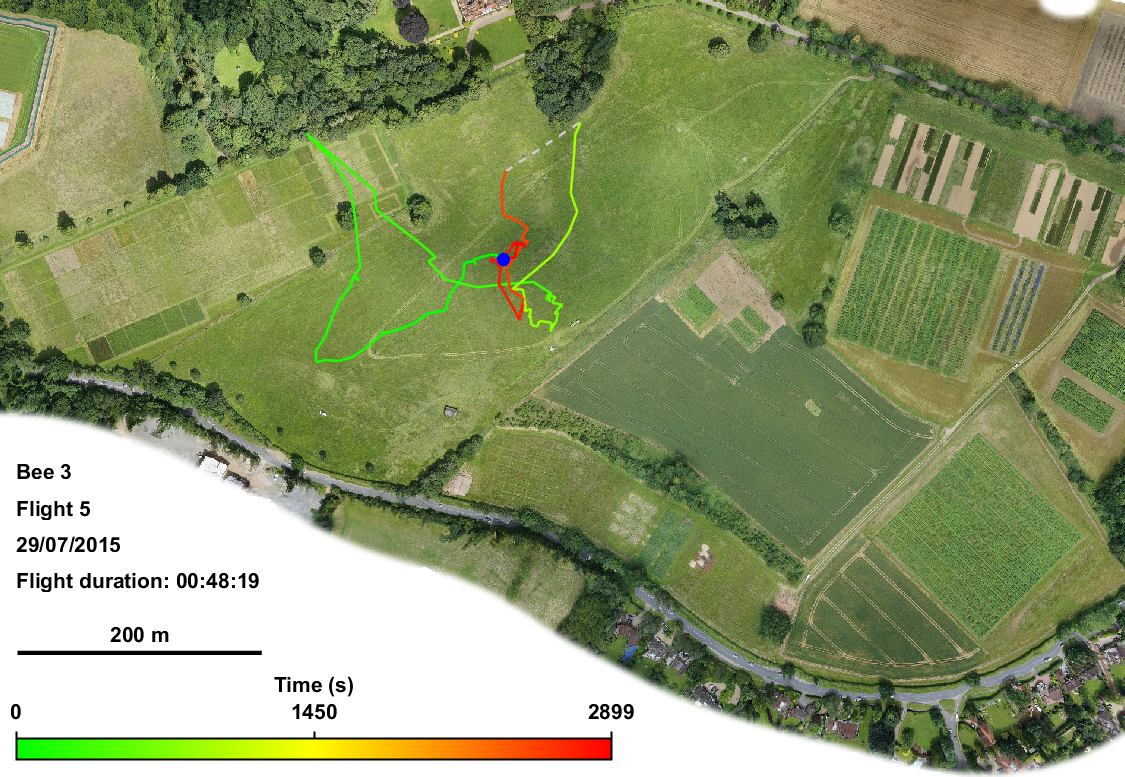

Supplement: S6 File — All flights made by Bee 3 during the period 23/07/15–06/08/15. Each figure represents a single flight. Bee ID, flight number, date of recording and the duration of each flight are shown in the bottom left corner of each figure. The position of the nest is marked by a blue circle. Colours represent the time from the start of each flight: initial portion of each flight is in green, changing smoothly through yellow until the end of the flight is shown in red. Grey dashed lines are used to join radar observations made more than 30 s apart, when the bee’s location was uncertain. (ZIP) [file pone.0160333.s008.zip › S193 Fig.tif]

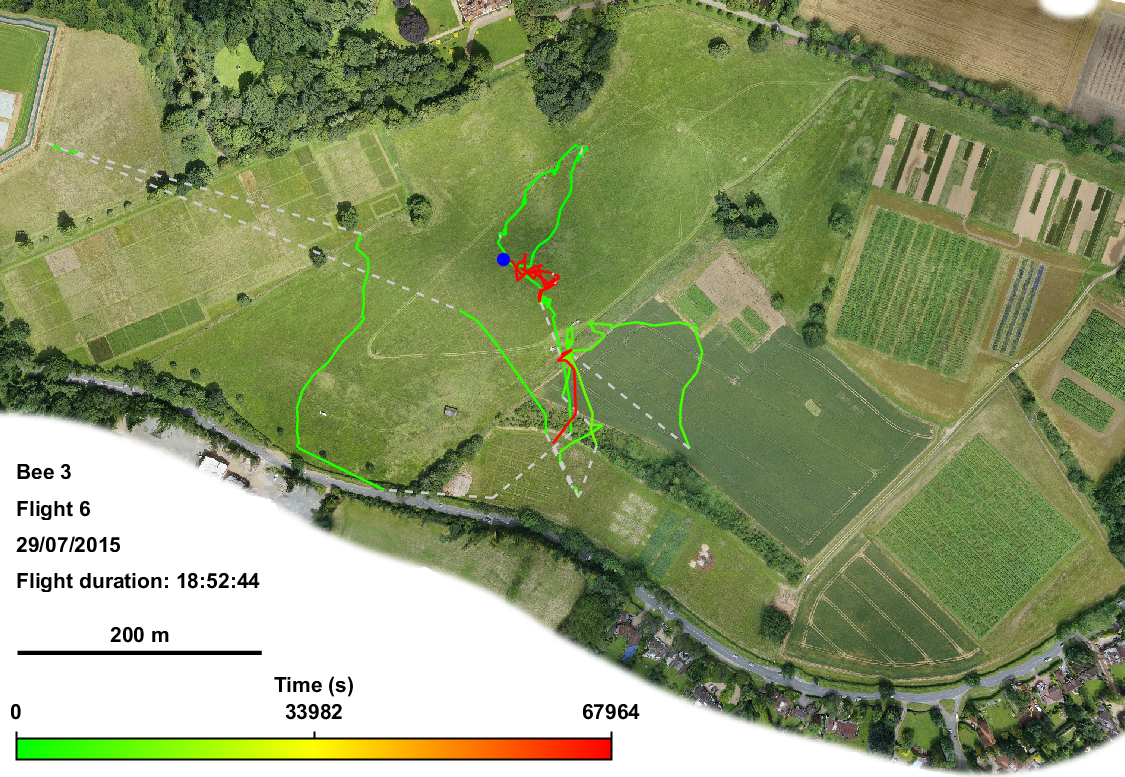

Supplement: S6 File — All flights made by Bee 3 during the period 23/07/15–06/08/15. Each figure represents a single flight. Bee ID, flight number, date of recording and the duration of each flight are shown in the bottom left corner of each figure. The position of the nest is marked by a blue circle. Colours represent the time from the start of each flight: initial portion of each flight is in green, changing smoothly through yellow until the end of the flight is shown in red. Grey dashed lines are used to join radar observations made more than 30 s apart, when the bee’s location was uncertain. (ZIP) [file pone.0160333.s008.zip › S194 Fig.tif]

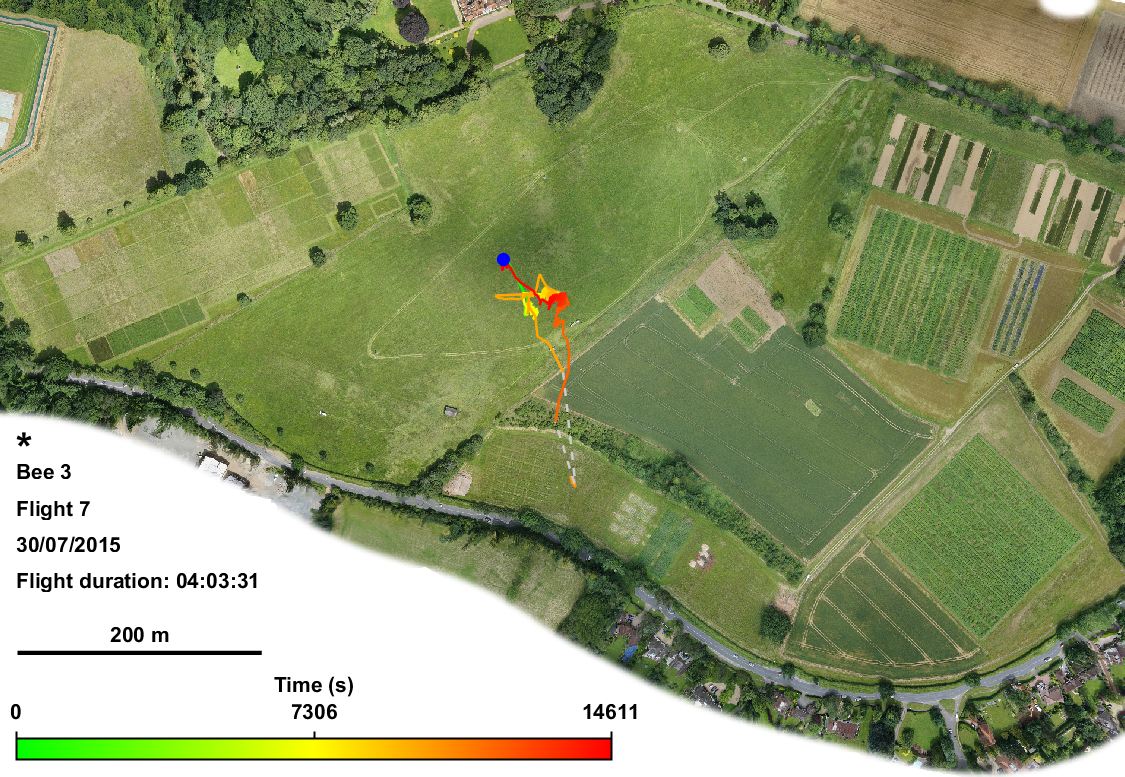

Supplement: S6 File — All flights made by Bee 3 during the period 23/07/15–06/08/15. Each figure represents a single flight. Bee ID, flight number, date of recording and the duration of each flight are shown in the bottom left corner of each figure. The position of the nest is marked by a blue circle. Colours represent the time from the start of each flight: initial portion of each flight is in green, changing smoothly through yellow until the end of the flight is shown in red. Grey dashed lines are used to join radar observations made more than 30 s apart, when the bee’s location was uncertain. (ZIP) [file pone.0160333.s008.zip › S195 Fig.tif]

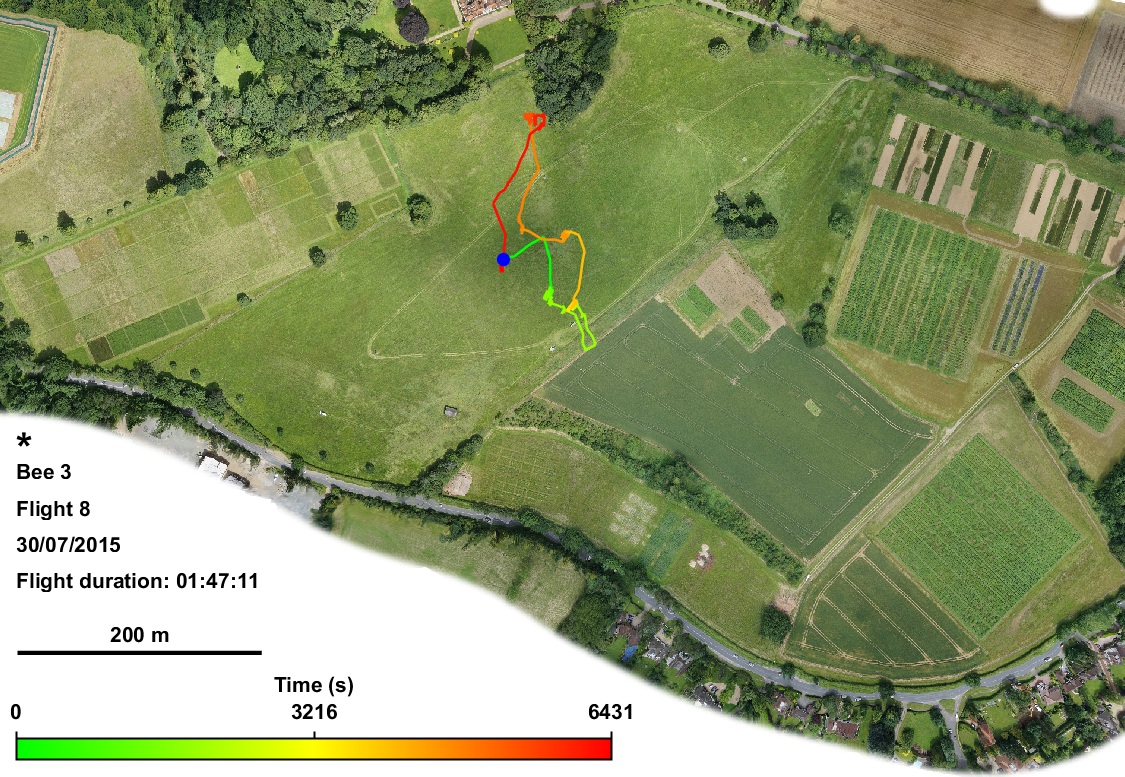

Supplement: S6 File — All flights made by Bee 3 during the period 23/07/15–06/08/15. Each figure represents a single flight. Bee ID, flight number, date of recording and the duration of each flight are shown in the bottom left corner of each figure. The position of the nest is marked by a blue circle. Colours represent the time from the start of each flight: initial portion of each flight is in green, changing smoothly through yellow until the end of the flight is shown in red. Grey dashed lines are used to join radar observations made more than 30 s apart, when the bee’s location was uncertain. (ZIP) [file pone.0160333.s008.zip › S196 Fig.tif]

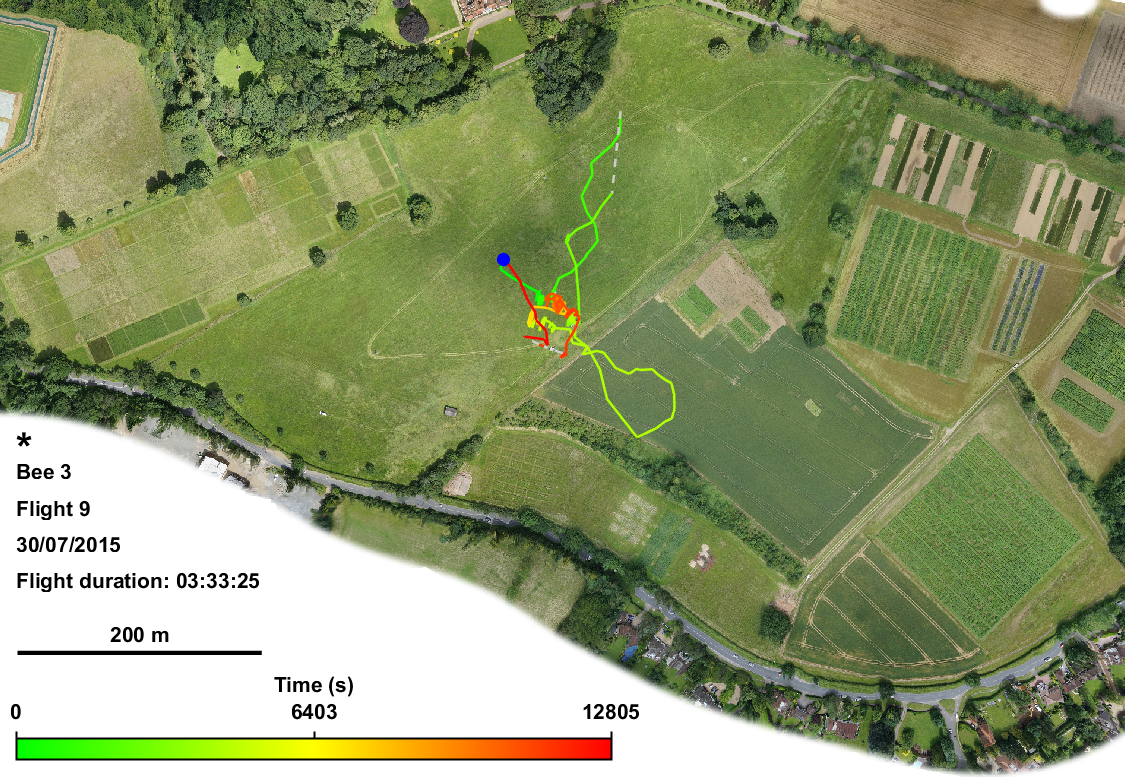

Supplement: S6 File — All flights made by Bee 3 during the period 23/07/15–06/08/15. Each figure represents a single flight. Bee ID, flight number, date of recording and the duration of each flight are shown in the bottom left corner of each figure. The position of the nest is marked by a blue circle. Colours represent the time from the start of each flight: initial portion of each flight is in green, changing smoothly through yellow until the end of the flight is shown in red. Grey dashed lines are used to join radar observations made more than 30 s apart, when the bee’s location was uncertain. (ZIP) [file pone.0160333.s008.zip › S197 Fig.tif]

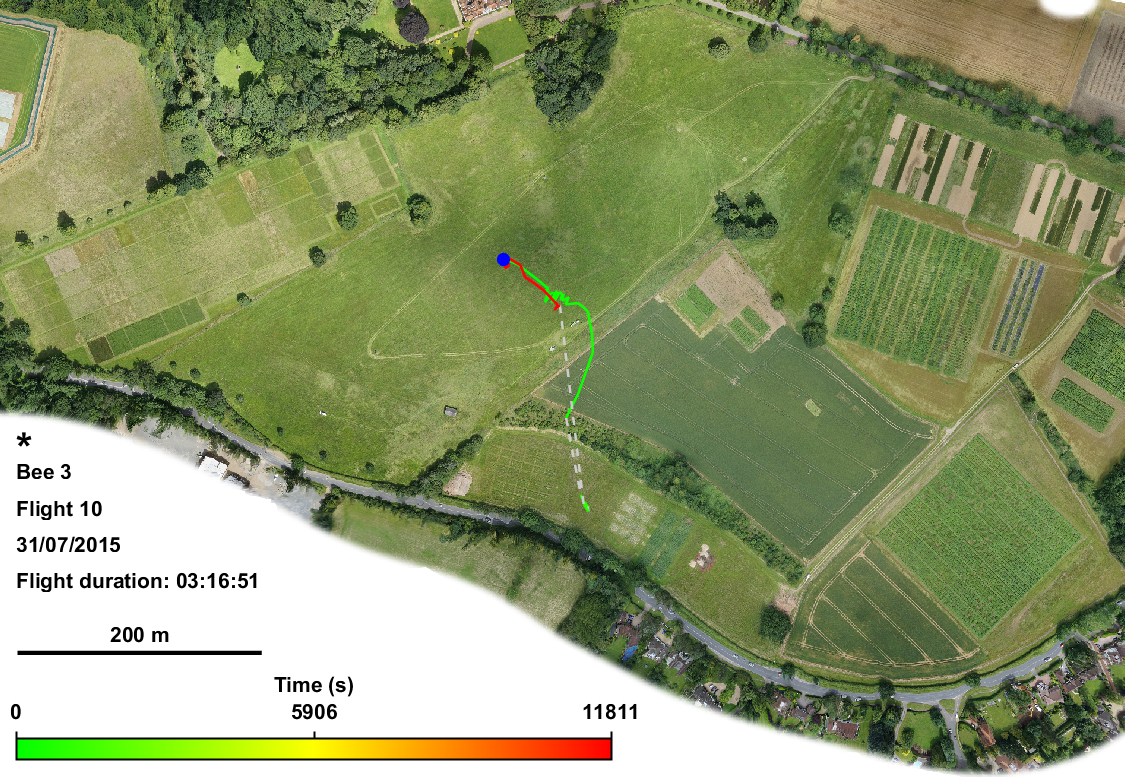

Supplement: S6 File — All flights made by Bee 3 during the period 23/07/15–06/08/15. Each figure represents a single flight. Bee ID, flight number, date of recording and the duration of each flight are shown in the bottom left corner of each figure. The position of the nest is marked by a blue circle. Colours represent the time from the start of each flight: initial portion of each flight is in green, changing smoothly through yellow until the end of the flight is shown in red. Grey dashed lines are used to join radar observations made more than 30 s apart, when the bee’s location was uncertain. (ZIP) [file pone.0160333.s008.zip › S198 Fig.tif]

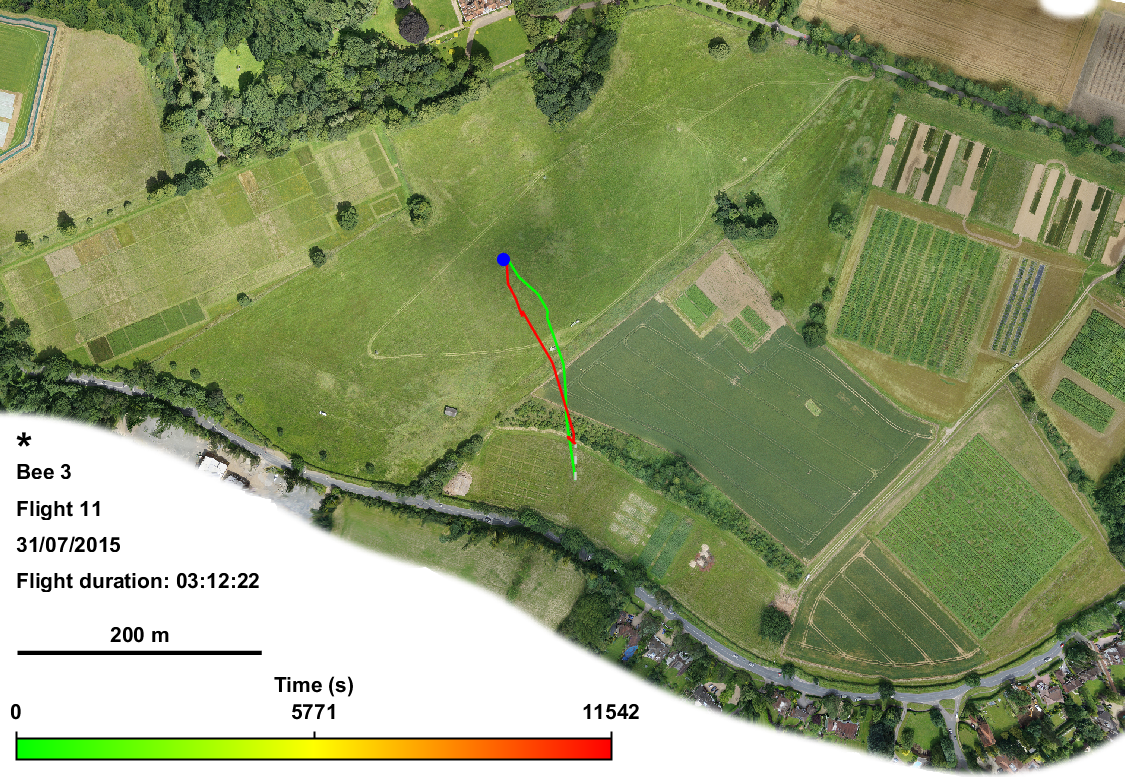

Supplement: S6 File — All flights made by Bee 3 during the period 23/07/15–06/08/15. Each figure represents a single flight. Bee ID, flight number, date of recording and the duration of each flight are shown in the bottom left corner of each figure. The position of the nest is marked by a blue circle. Colours represent the time from the start of each flight: initial portion of each flight is in green, changing smoothly through yellow until the end of the flight is shown in red. Grey dashed lines are used to join radar observations made more than 30 s apart, when the bee’s location was uncertain. (ZIP) [file pone.0160333.s008.zip › S199 Fig.tif]

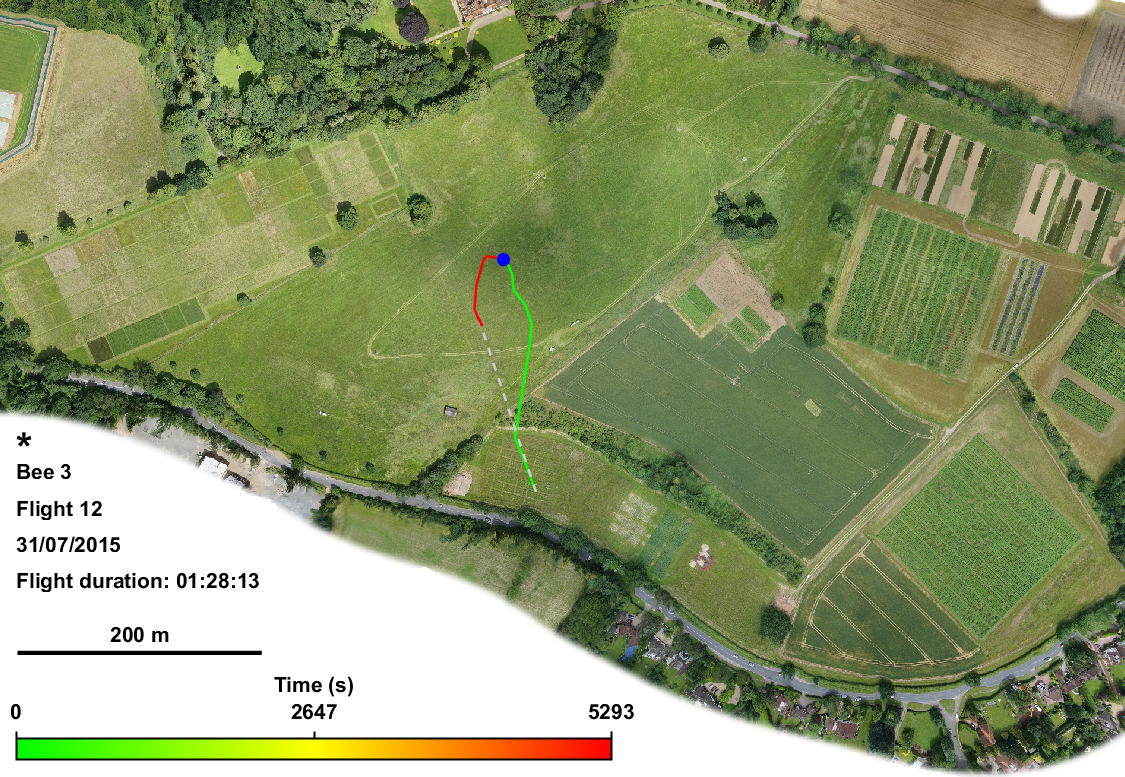

Supplement: S6 File — All flights made by Bee 3 during the period 23/07/15–06/08/15. Each figure represents a single flight. Bee ID, flight number, date of recording and the duration of each flight are shown in the bottom left corner of each figure. The position of the nest is marked by a blue circle. Colours represent the time from the start of each flight: initial portion of each flight is in green, changing smoothly through yellow until the end of the flight is shown in red. Grey dashed lines are used to join radar observations made more than 30 s apart, when the bee’s location was uncertain. (ZIP) [file pone.0160333.s008.zip › S200 Fig.tif]
