# Supplementary figures and images for: Life-Long Radar Tracking of Bumblebees (part 3 of 3)
Source: PLoS One. 2016 Aug 4;11(8):e0160333. doi: 10.1371/journal.pone.0160333 (PMC4973990; doi:10.1371/journal.pone.0160333)

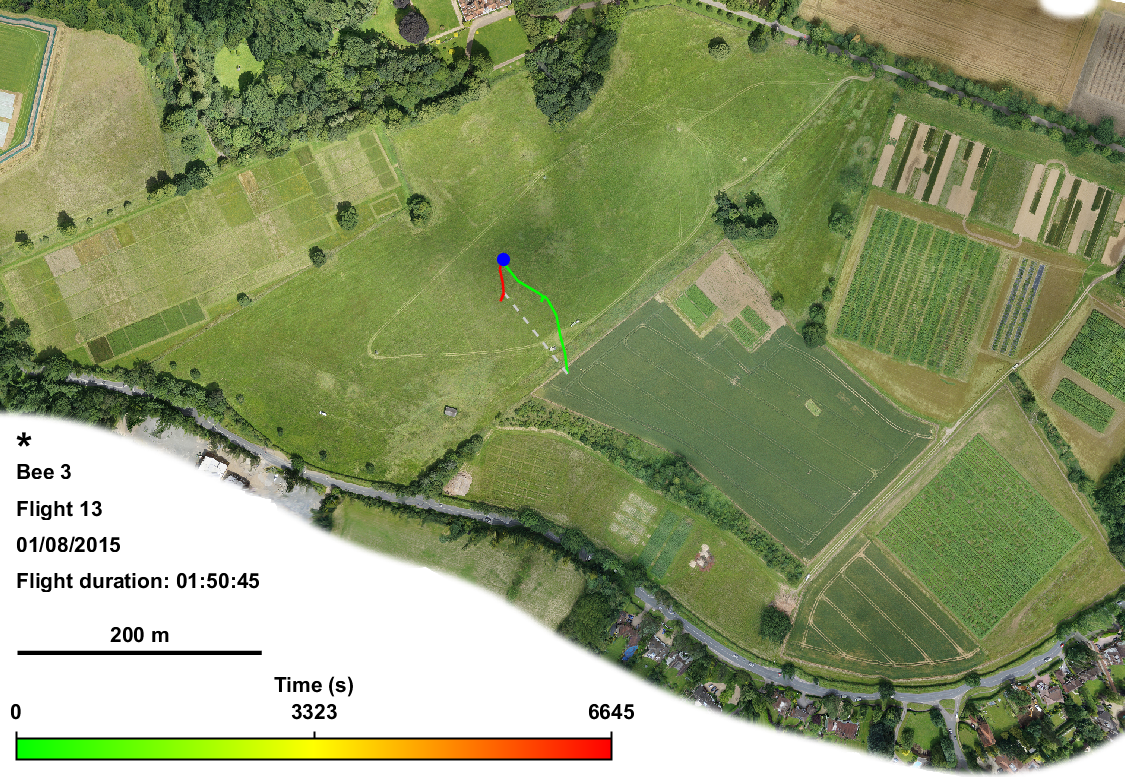

Supplement: S6 File — All flights made by Bee 3 during the period 23/07/15–06/08/15. Each figure represents a single flight. Bee ID, flight number, date of recording and the duration of each flight are shown in the bottom left corner of each figure. The position of the nest is marked by a blue circle. Colours represent the time from the start of each flight: initial portion of each flight is in green, changing smoothly through yellow until the end of the flight is shown in red. Grey dashed lines are used to join radar observations made more than 30 s apart, when the bee’s location was uncertain. (ZIP) [file pone.0160333.s008.zip › S201 Fig.tif]

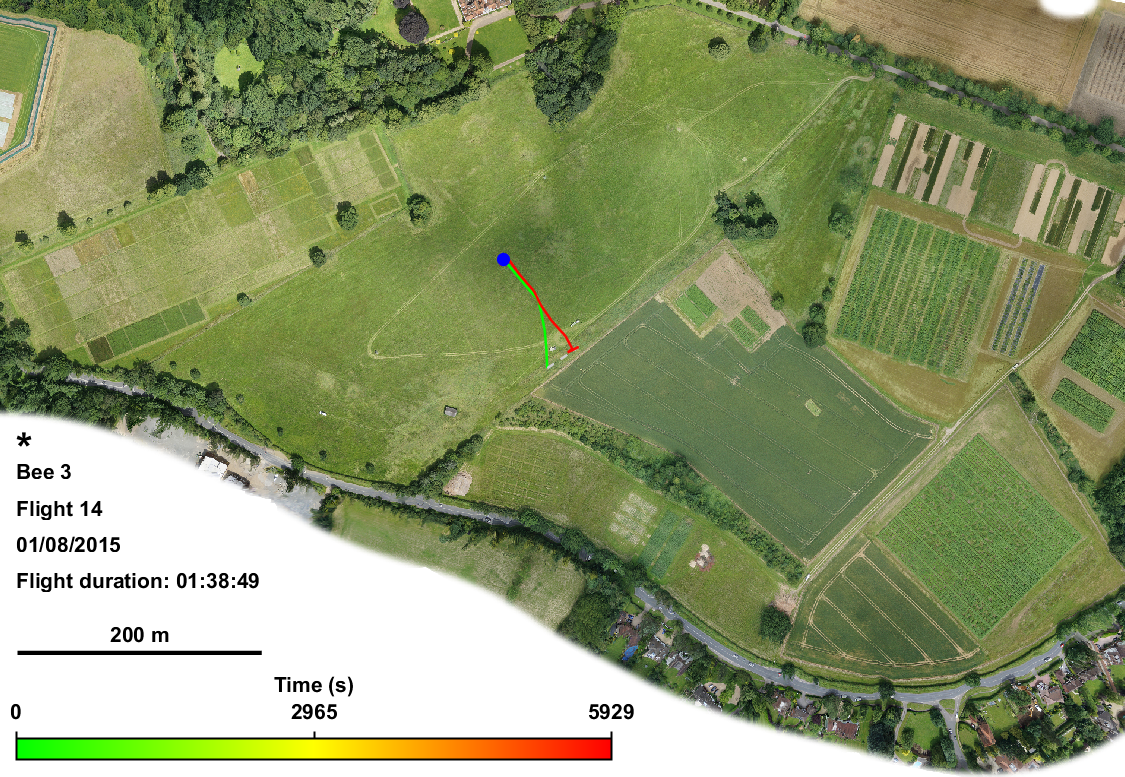

Supplement: S6 File — All flights made by Bee 3 during the period 23/07/15–06/08/15. Each figure represents a single flight. Bee ID, flight number, date of recording and the duration of each flight are shown in the bottom left corner of each figure. The position of the nest is marked by a blue circle. Colours represent the time from the start of each flight: initial portion of each flight is in green, changing smoothly through yellow until the end of the flight is shown in red. Grey dashed lines are used to join radar observations made more than 30 s apart, when the bee’s location was uncertain. (ZIP) [file pone.0160333.s008.zip › S202 Fig.tif]

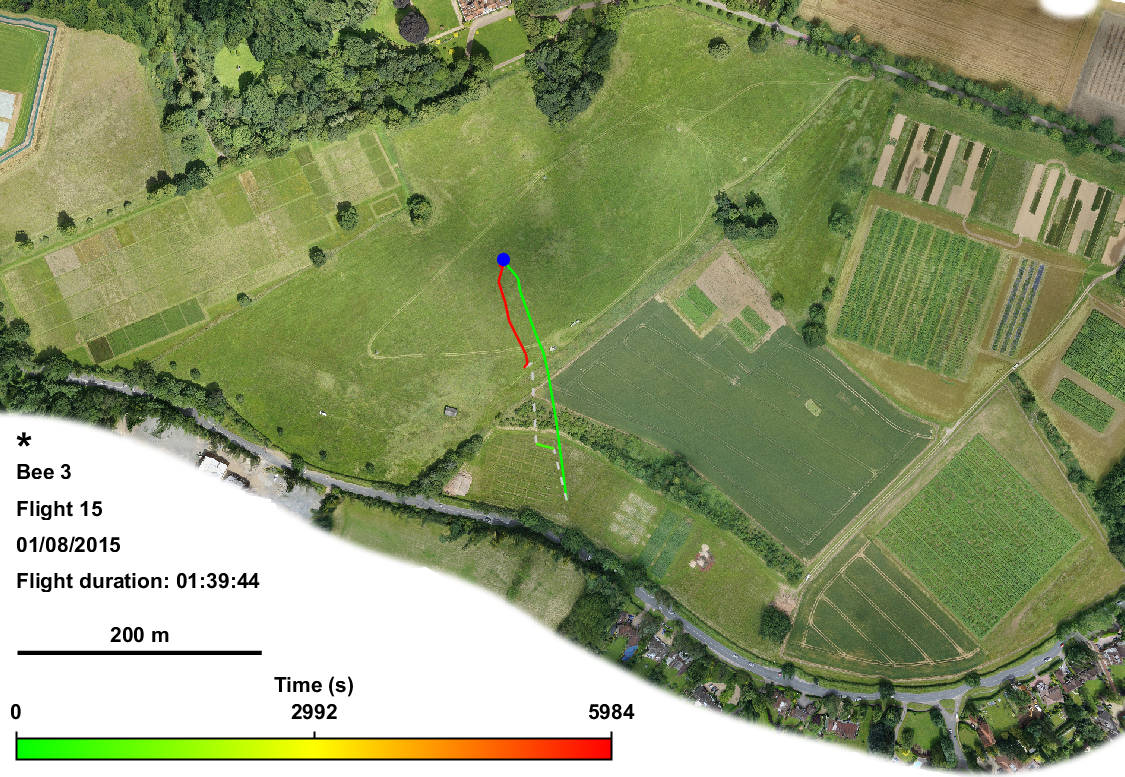

Supplement: S6 File — All flights made by Bee 3 during the period 23/07/15–06/08/15. Each figure represents a single flight. Bee ID, flight number, date of recording and the duration of each flight are shown in the bottom left corner of each figure. The position of the nest is marked by a blue circle. Colours represent the time from the start of each flight: initial portion of each flight is in green, changing smoothly through yellow until the end of the flight is shown in red. Grey dashed lines are used to join radar observations made more than 30 s apart, when the bee’s location was uncertain. (ZIP) [file pone.0160333.s008.zip › S203 Fig.tif]

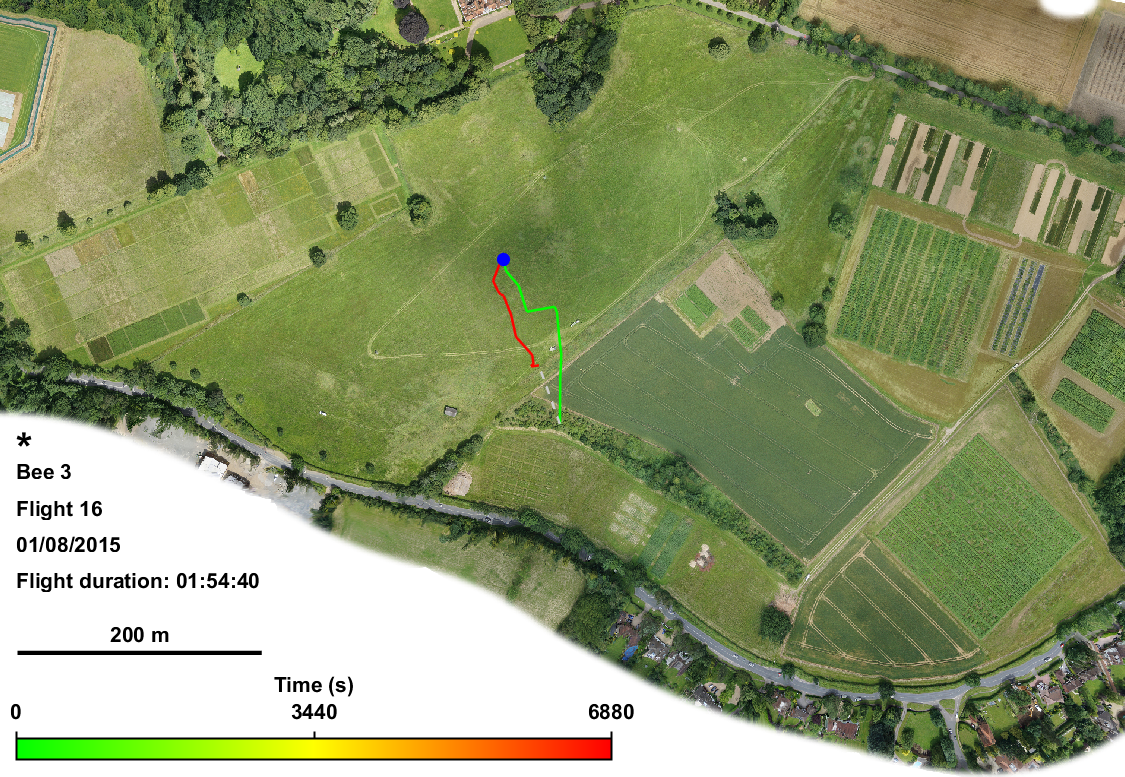

Supplement: S6 File — All flights made by Bee 3 during the period 23/07/15–06/08/15. Each figure represents a single flight. Bee ID, flight number, date of recording and the duration of each flight are shown in the bottom left corner of each figure. The position of the nest is marked by a blue circle. Colours represent the time from the start of each flight: initial portion of each flight is in green, changing smoothly through yellow until the end of the flight is shown in red. Grey dashed lines are used to join radar observations made more than 30 s apart, when the bee’s location was uncertain. (ZIP) [file pone.0160333.s008.zip › S204 Fig.tif]

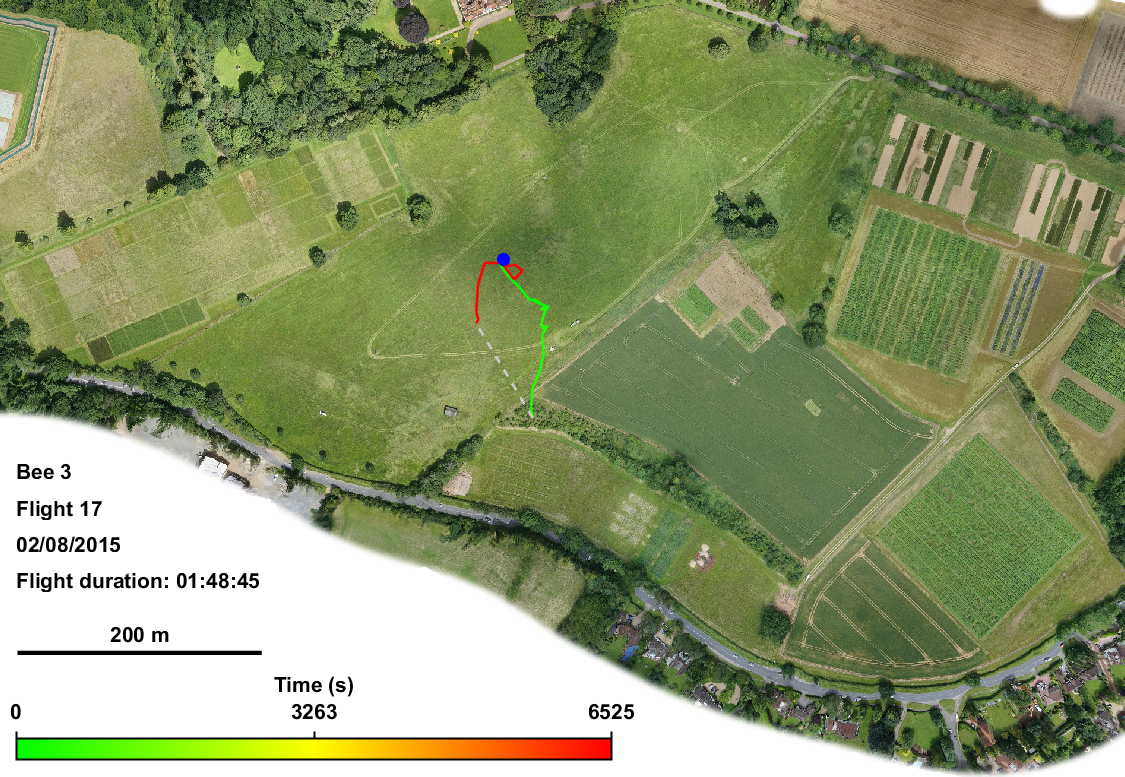

Supplement: S6 File — All flights made by Bee 3 during the period 23/07/15–06/08/15. Each figure represents a single flight. Bee ID, flight number, date of recording and the duration of each flight are shown in the bottom left corner of each figure. The position of the nest is marked by a blue circle. Colours represent the time from the start of each flight: initial portion of each flight is in green, changing smoothly through yellow until the end of the flight is shown in red. Grey dashed lines are used to join radar observations made more than 30 s apart, when the bee’s location was uncertain. (ZIP) [file pone.0160333.s008.zip › S205 Fig.tif]

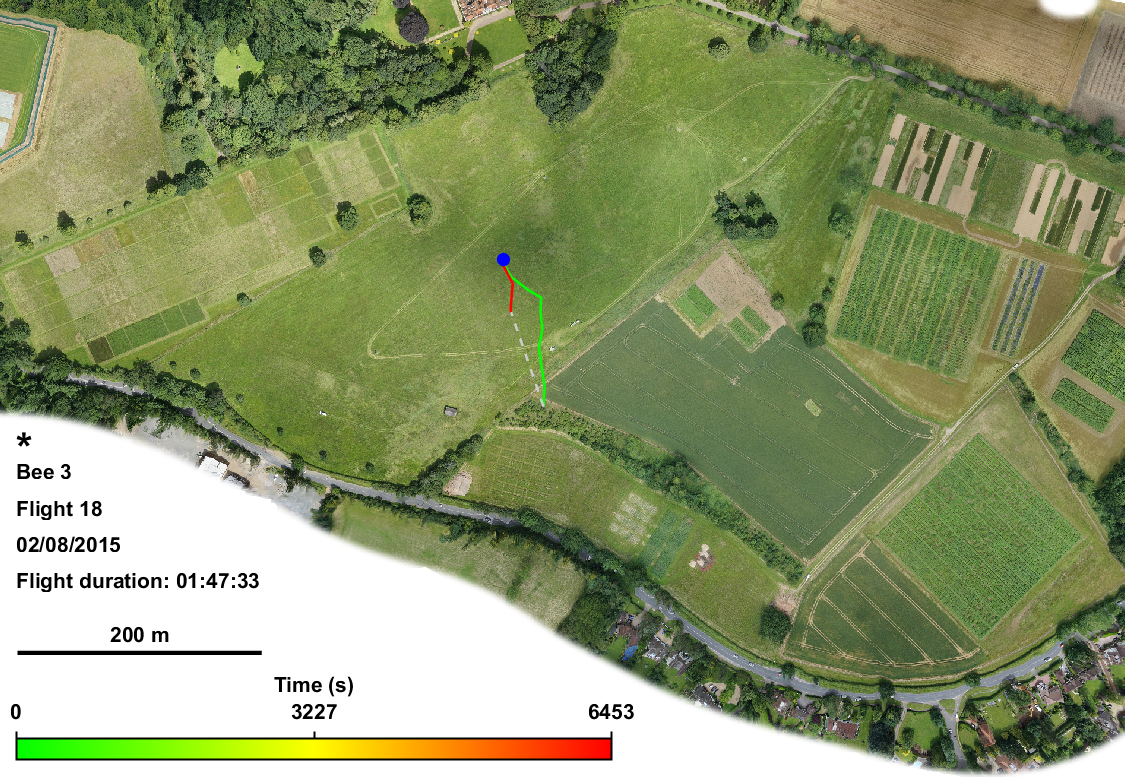

Supplement: S6 File — All flights made by Bee 3 during the period 23/07/15–06/08/15. Each figure represents a single flight. Bee ID, flight number, date of recording and the duration of each flight are shown in the bottom left corner of each figure. The position of the nest is marked by a blue circle. Colours represent the time from the start of each flight: initial portion of each flight is in green, changing smoothly through yellow until the end of the flight is shown in red. Grey dashed lines are used to join radar observations made more than 30 s apart, when the bee’s location was uncertain. (ZIP) [file pone.0160333.s008.zip › S206 Fig.tif]

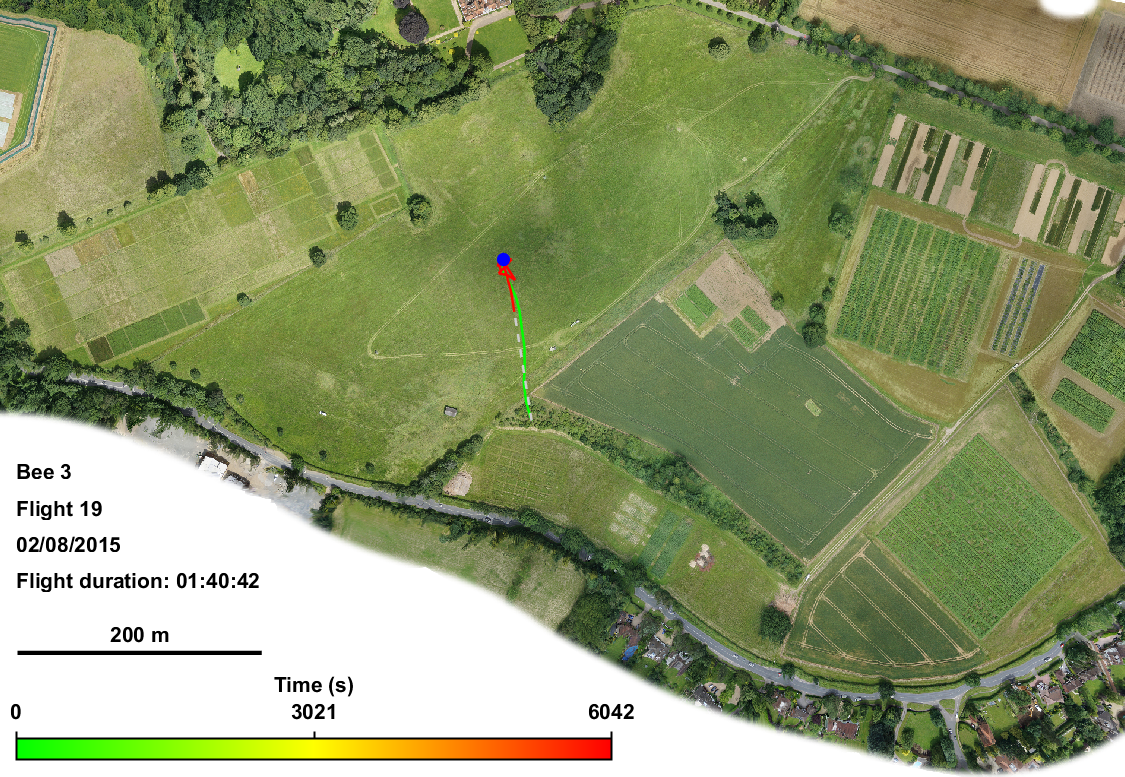

Supplement: S6 File — All flights made by Bee 3 during the period 23/07/15–06/08/15. Each figure represents a single flight. Bee ID, flight number, date of recording and the duration of each flight are shown in the bottom left corner of each figure. The position of the nest is marked by a blue circle. Colours represent the time from the start of each flight: initial portion of each flight is in green, changing smoothly through yellow until the end of the flight is shown in red. Grey dashed lines are used to join radar observations made more than 30 s apart, when the bee’s location was uncertain. (ZIP) [file pone.0160333.s008.zip › S207 Fig.tif]

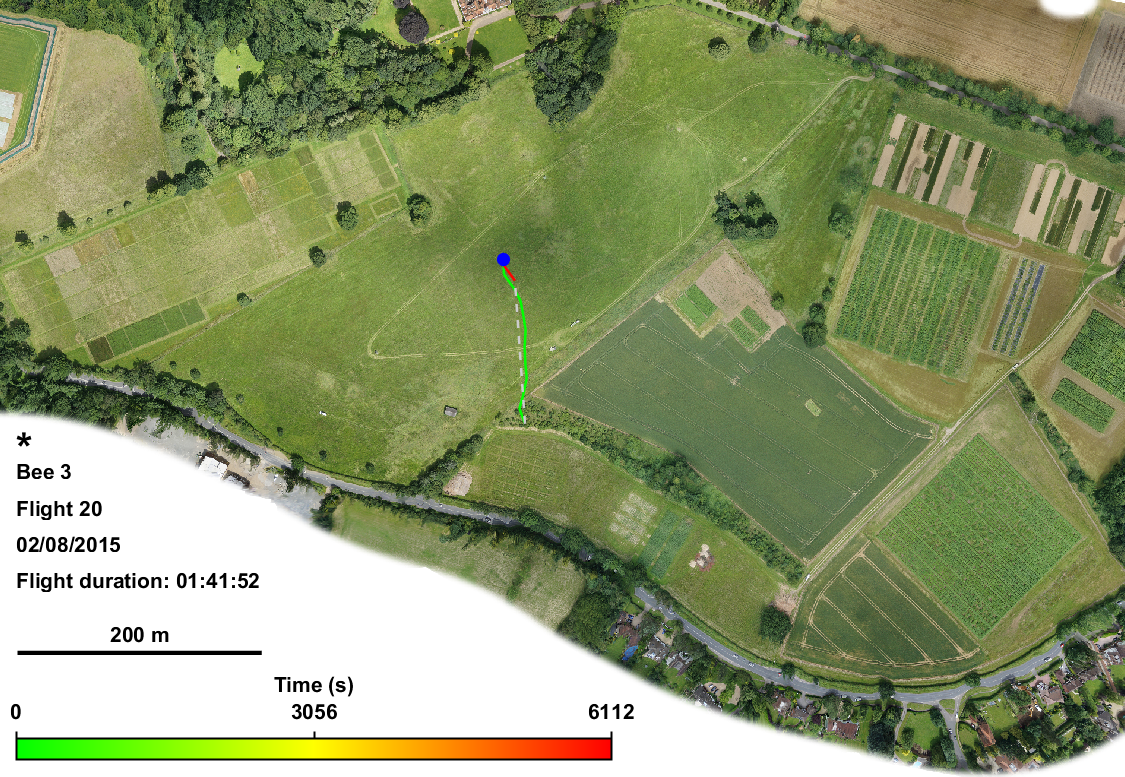

Supplement: S6 File — All flights made by Bee 3 during the period 23/07/15–06/08/15. Each figure represents a single flight. Bee ID, flight number, date of recording and the duration of each flight are shown in the bottom left corner of each figure. The position of the nest is marked by a blue circle. Colours represent the time from the start of each flight: initial portion of each flight is in green, changing smoothly through yellow until the end of the flight is shown in red. Grey dashed lines are used to join radar observations made more than 30 s apart, when the bee’s location was uncertain. (ZIP) [file pone.0160333.s008.zip › S208 Fig.tif]

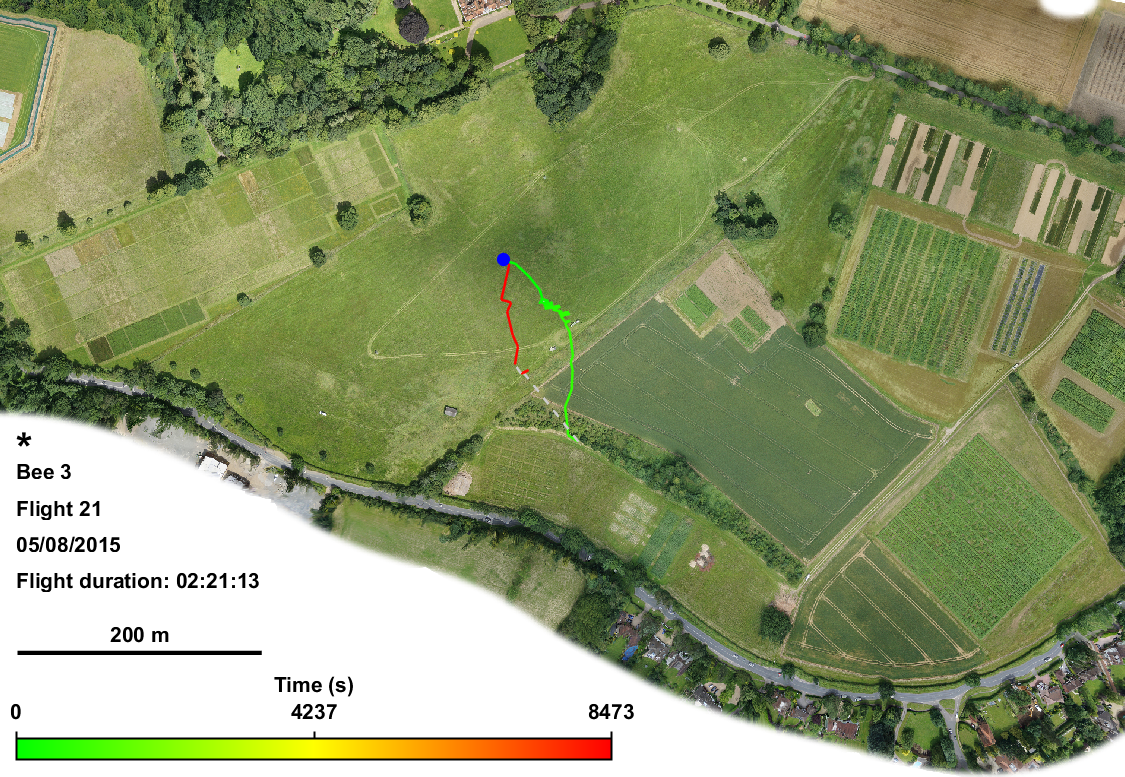

Supplement: S6 File — All flights made by Bee 3 during the period 23/07/15–06/08/15. Each figure represents a single flight. Bee ID, flight number, date of recording and the duration of each flight are shown in the bottom left corner of each figure. The position of the nest is marked by a blue circle. Colours represent the time from the start of each flight: initial portion of each flight is in green, changing smoothly through yellow until the end of the flight is shown in red. Grey dashed lines are used to join radar observations made more than 30 s apart, when the bee’s location was uncertain. (ZIP) [file pone.0160333.s008.zip › S209 Fig.tif]

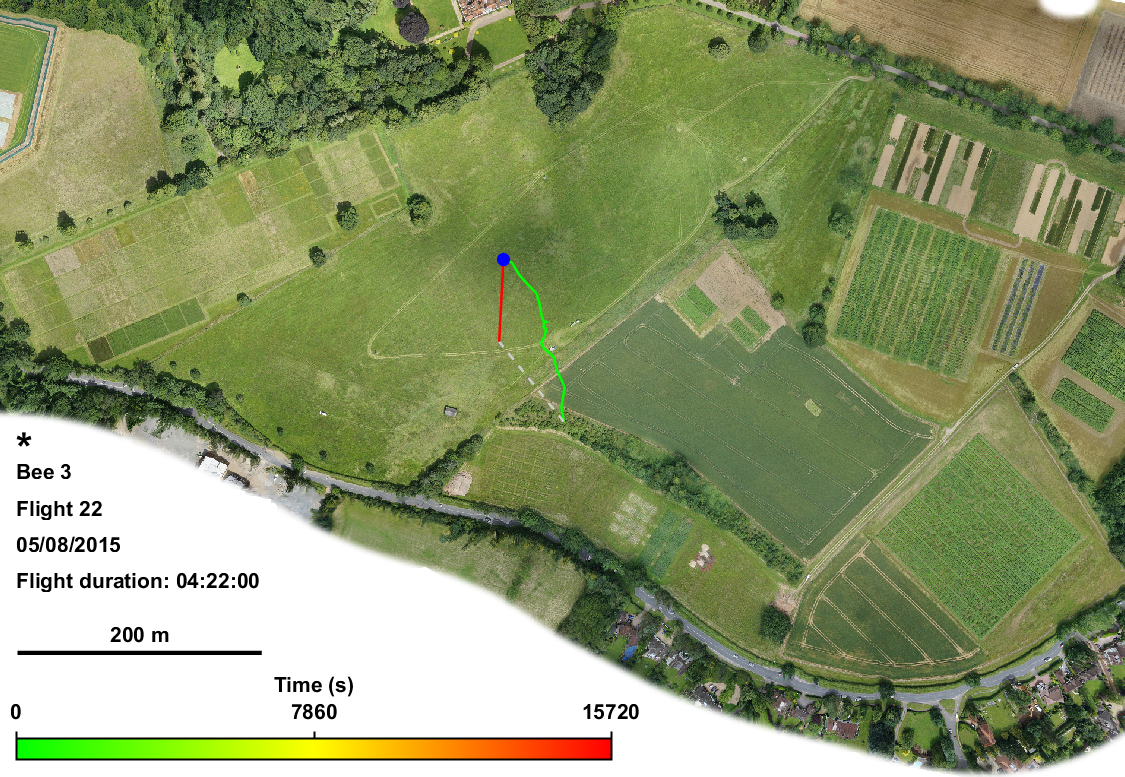

Supplement: S6 File — All flights made by Bee 3 during the period 23/07/15–06/08/15. Each figure represents a single flight. Bee ID, flight number, date of recording and the duration of each flight are shown in the bottom left corner of each figure. The position of the nest is marked by a blue circle. Colours represent the time from the start of each flight: initial portion of each flight is in green, changing smoothly through yellow until the end of the flight is shown in red. Grey dashed lines are used to join radar observations made more than 30 s apart, when the bee’s location was uncertain. (ZIP) [file pone.0160333.s008.zip › S210 Fig.tif]

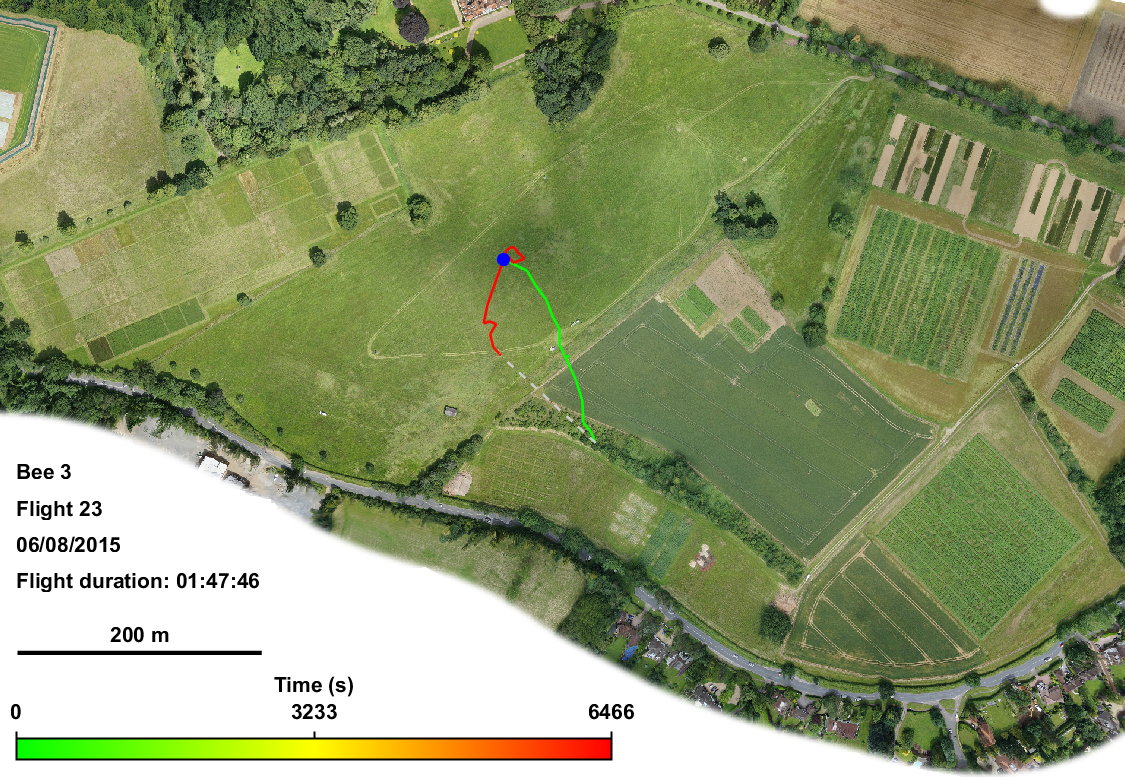

Supplement: S6 File — All flights made by Bee 3 during the period 23/07/15–06/08/15. Each figure represents a single flight. Bee ID, flight number, date of recording and the duration of each flight are shown in the bottom left corner of each figure. The position of the nest is marked by a blue circle. Colours represent the time from the start of each flight: initial portion of each flight is in green, changing smoothly through yellow until the end of the flight is shown in red. Grey dashed lines are used to join radar observations made more than 30 s apart, when the bee’s location was uncertain. (ZIP) [file pone.0160333.s008.zip › S211 Fig.tif]

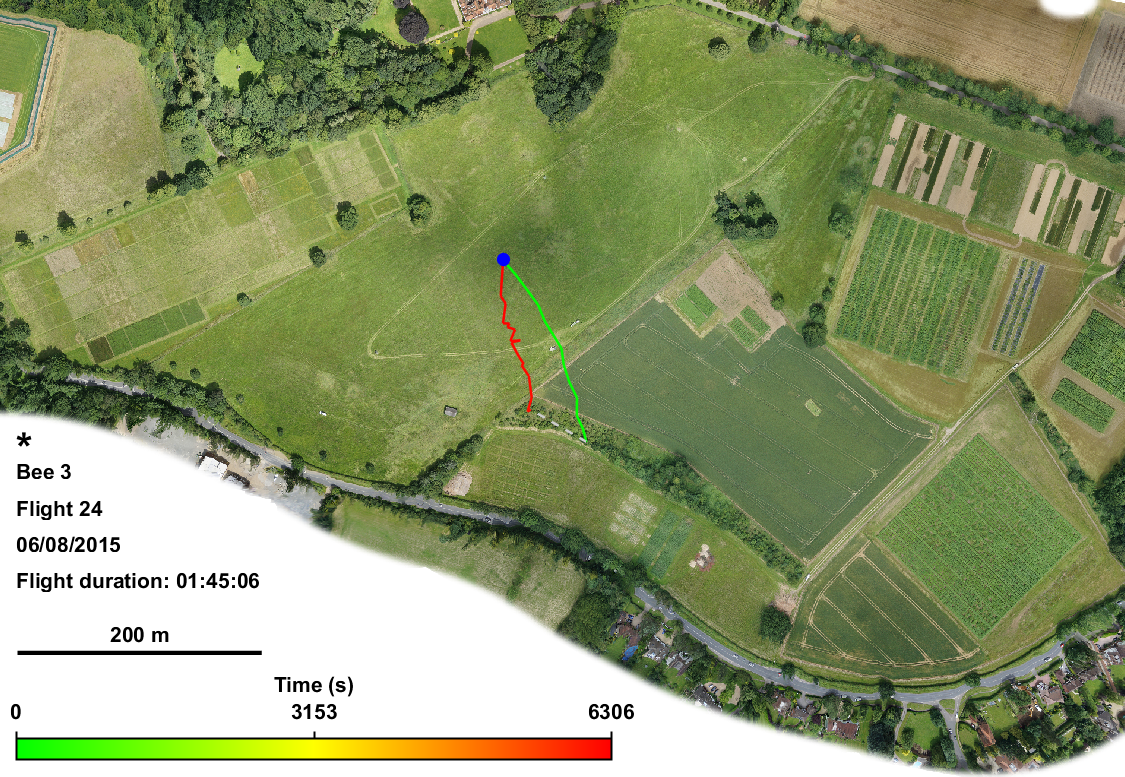

Supplement: S6 File — All flights made by Bee 3 during the period 23/07/15–06/08/15. Each figure represents a single flight. Bee ID, flight number, date of recording and the duration of each flight are shown in the bottom left corner of each figure. The position of the nest is marked by a blue circle. Colours represent the time from the start of each flight: initial portion of each flight is in green, changing smoothly through yellow until the end of the flight is shown in red. Grey dashed lines are used to join radar observations made more than 30 s apart, when the bee’s location was uncertain. (ZIP) [file pone.0160333.s008.zip › S212 Fig.tif]

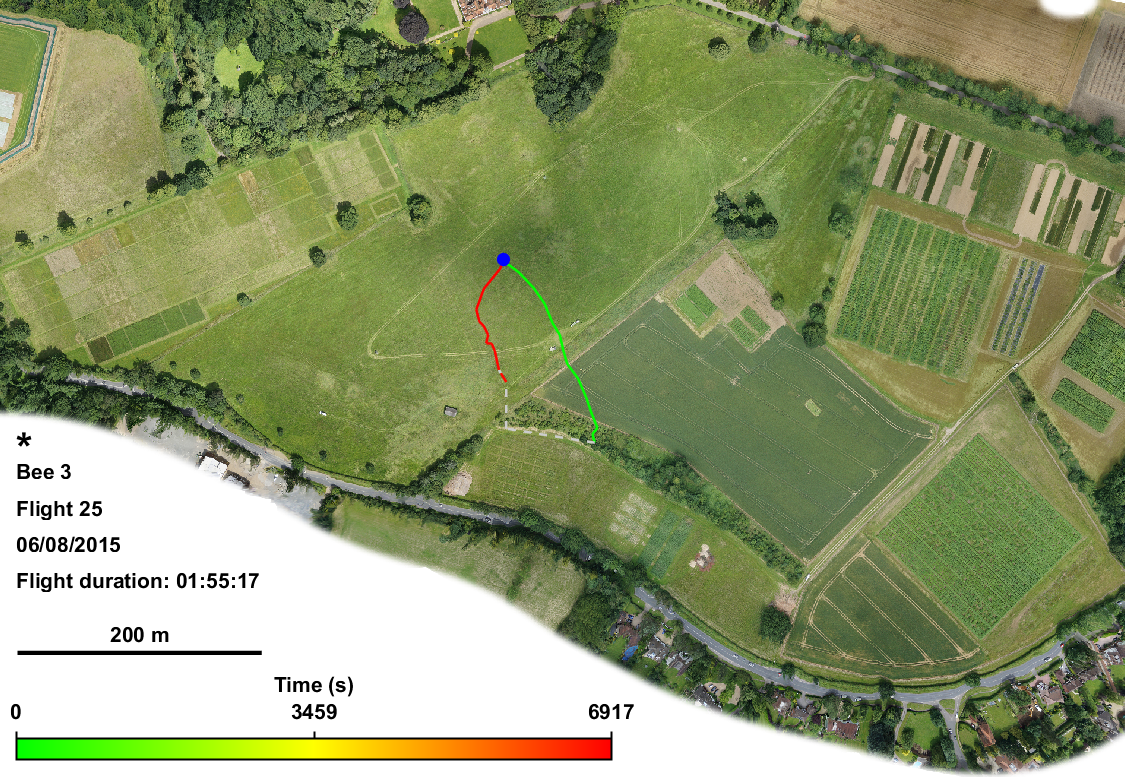

Supplement: S6 File — All flights made by Bee 3 during the period 23/07/15–06/08/15. Each figure represents a single flight. Bee ID, flight number, date of recording and the duration of each flight are shown in the bottom left corner of each figure. The position of the nest is marked by a blue circle. Colours represent the time from the start of each flight: initial portion of each flight is in green, changing smoothly through yellow until the end of the flight is shown in red. Grey dashed lines are used to join radar observations made more than 30 s apart, when the bee’s location was uncertain. (ZIP) [file pone.0160333.s008.zip › S213 Fig.tif]

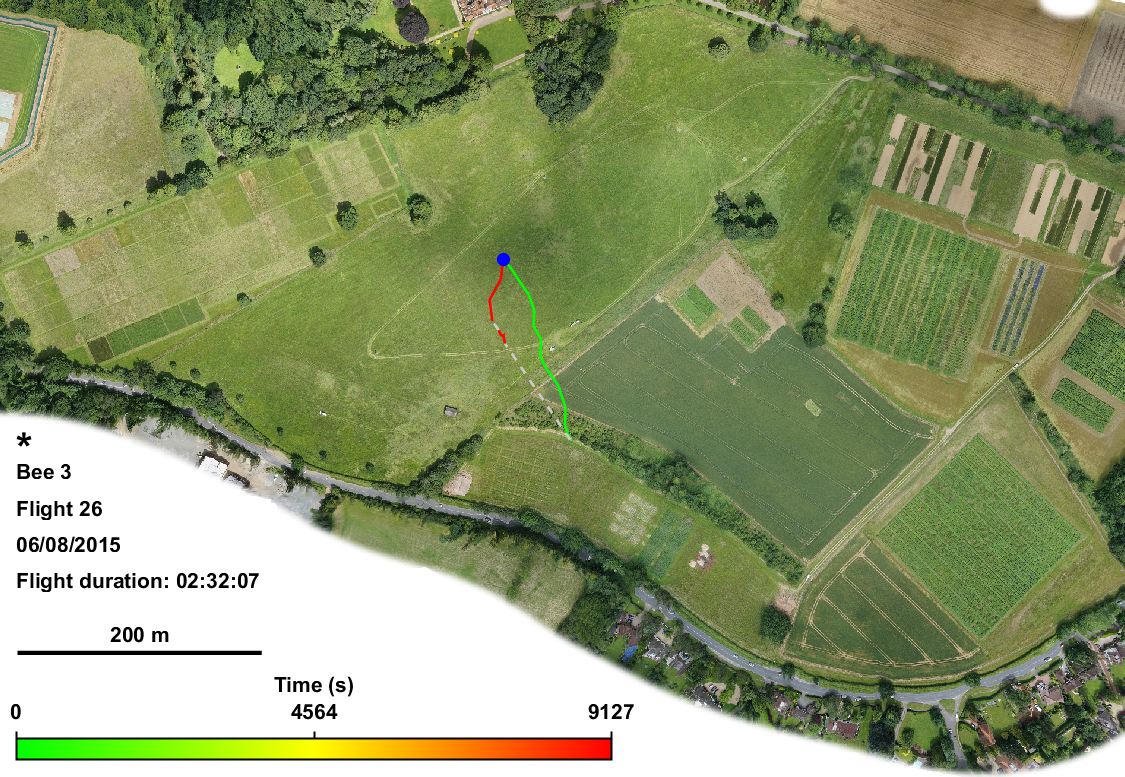

Supplement: S6 File — All flights made by Bee 3 during the period 23/07/15–06/08/15. Each figure represents a single flight. Bee ID, flight number, date of recording and the duration of each flight are shown in the bottom left corner of each figure. The position of the nest is marked by a blue circle. Colours represent the time from the start of each flight: initial portion of each flight is in green, changing smoothly through yellow until the end of the flight is shown in red. Grey dashed lines are used to join radar observations made more than 30 s apart, when the bee’s location was uncertain. (ZIP) [file pone.0160333.s008.zip › S214 Fig.tif]

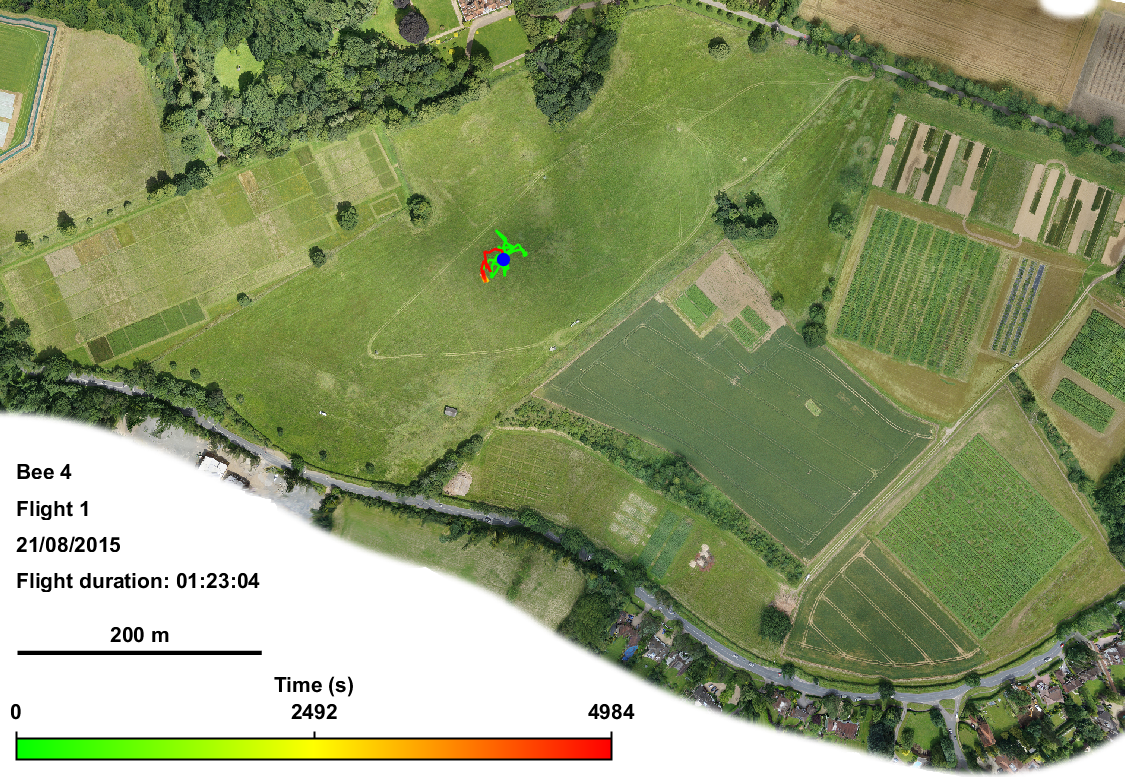

Supplement: S7 File — All flights made by Bee 4 during the period 21/08/15–03/09/15. Each figure represents a single flight. Bee ID, flight number, date of recording and the duration of each flight are shown in the bottom left corner of each figure. The position of the nest is marked by a blue circle. Colours represent the time from the start of each flight: initial portion of each flight is in green, changing smoothly through yellow until the end of the flight is shown in red. Grey dashed lines are used to join radar observations made more than 30 s apart, when the bee’s location was uncertain. (ZIP) [file pone.0160333.s009.zip › S215 Fig.tif]

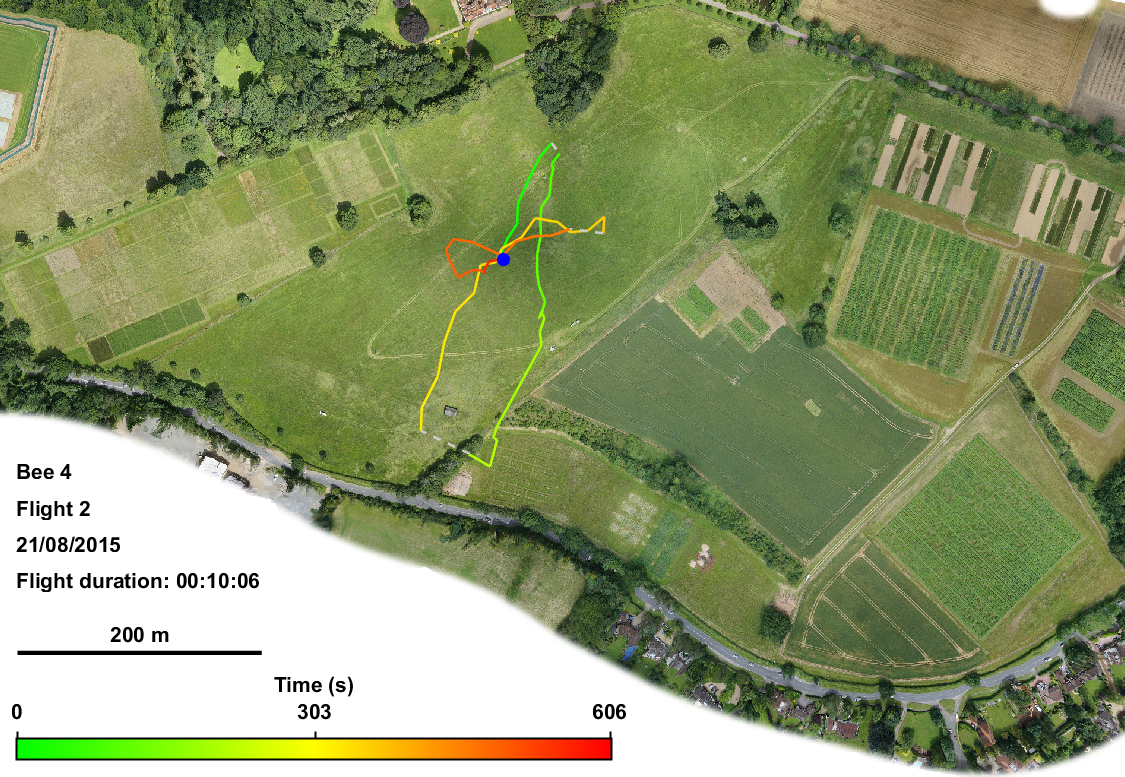

Supplement: S7 File — All flights made by Bee 4 during the period 21/08/15–03/09/15. Each figure represents a single flight. Bee ID, flight number, date of recording and the duration of each flight are shown in the bottom left corner of each figure. The position of the nest is marked by a blue circle. Colours represent the time from the start of each flight: initial portion of each flight is in green, changing smoothly through yellow until the end of the flight is shown in red. Grey dashed lines are used to join radar observations made more than 30 s apart, when the bee’s location was uncertain. (ZIP) [file pone.0160333.s009.zip › S216 Fig.tif]

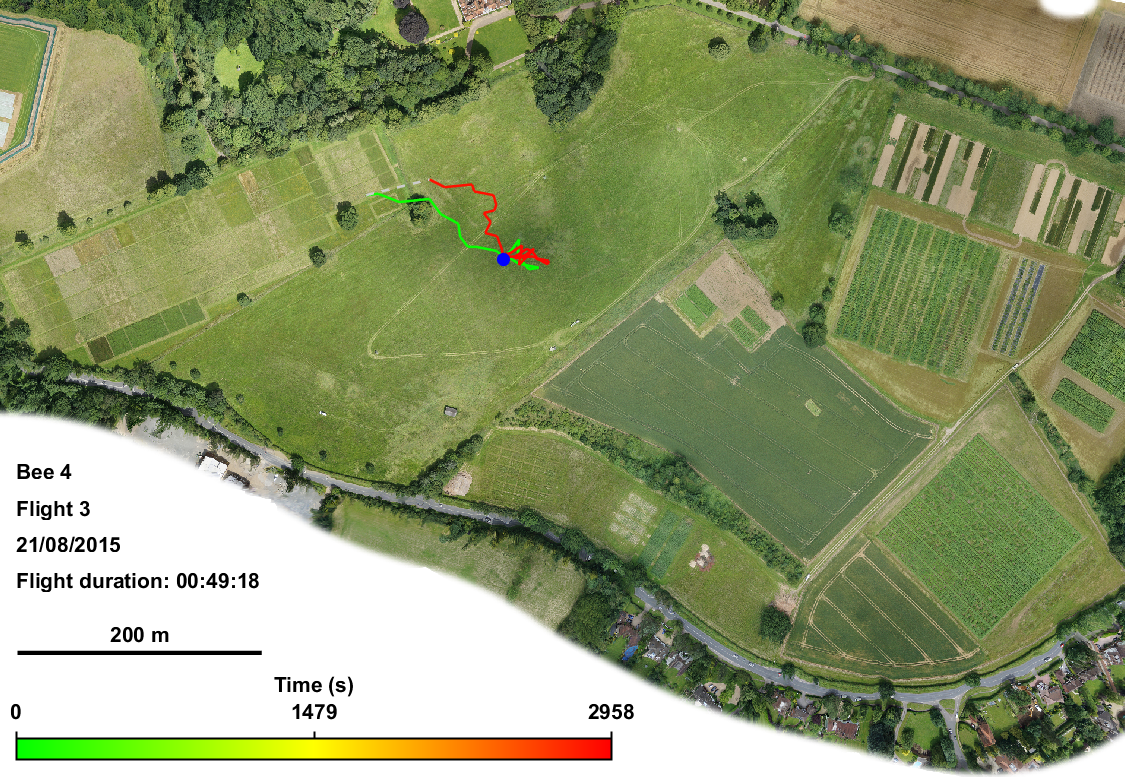

Supplement: S7 File — All flights made by Bee 4 during the period 21/08/15–03/09/15. Each figure represents a single flight. Bee ID, flight number, date of recording and the duration of each flight are shown in the bottom left corner of each figure. The position of the nest is marked by a blue circle. Colours represent the time from the start of each flight: initial portion of each flight is in green, changing smoothly through yellow until the end of the flight is shown in red. Grey dashed lines are used to join radar observations made more than 30 s apart, when the bee’s location was uncertain. (ZIP) [file pone.0160333.s009.zip › S217 Fig.tif]

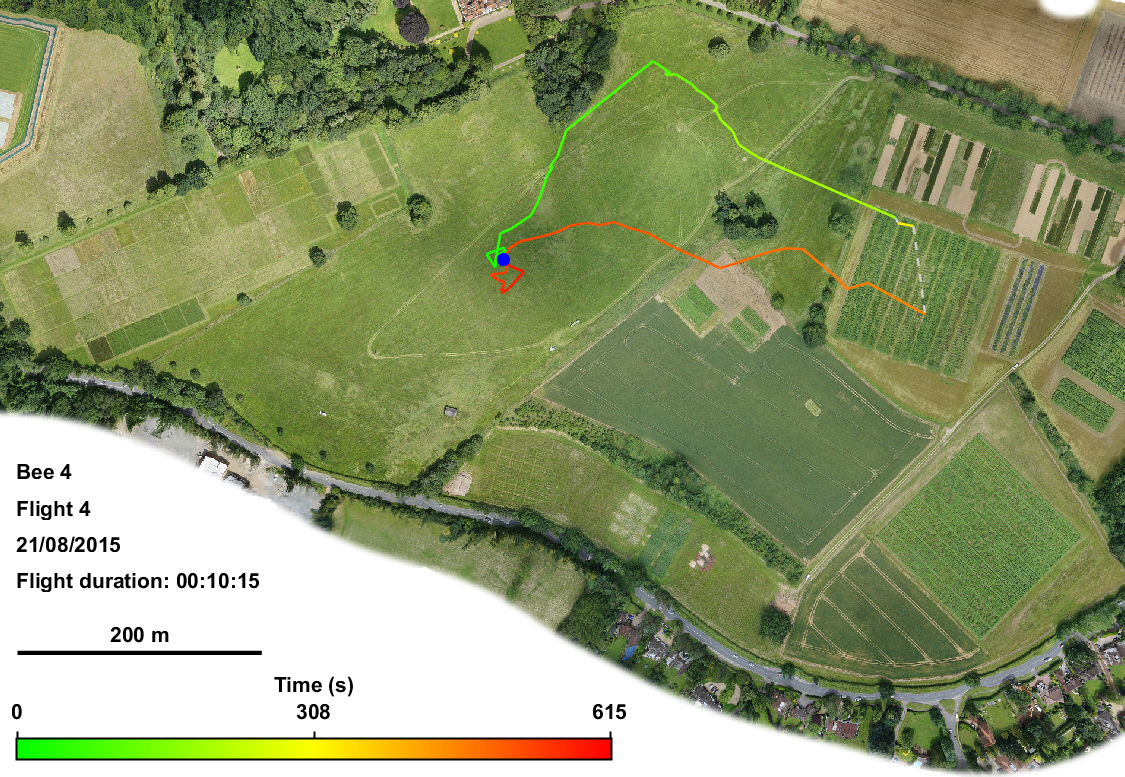

Supplement: S7 File — All flights made by Bee 4 during the period 21/08/15–03/09/15. Each figure represents a single flight. Bee ID, flight number, date of recording and the duration of each flight are shown in the bottom left corner of each figure. The position of the nest is marked by a blue circle. Colours represent the time from the start of each flight: initial portion of each flight is in green, changing smoothly through yellow until the end of the flight is shown in red. Grey dashed lines are used to join radar observations made more than 30 s apart, when the bee’s location was uncertain. (ZIP) [file pone.0160333.s009.zip › S218 Fig.tif]

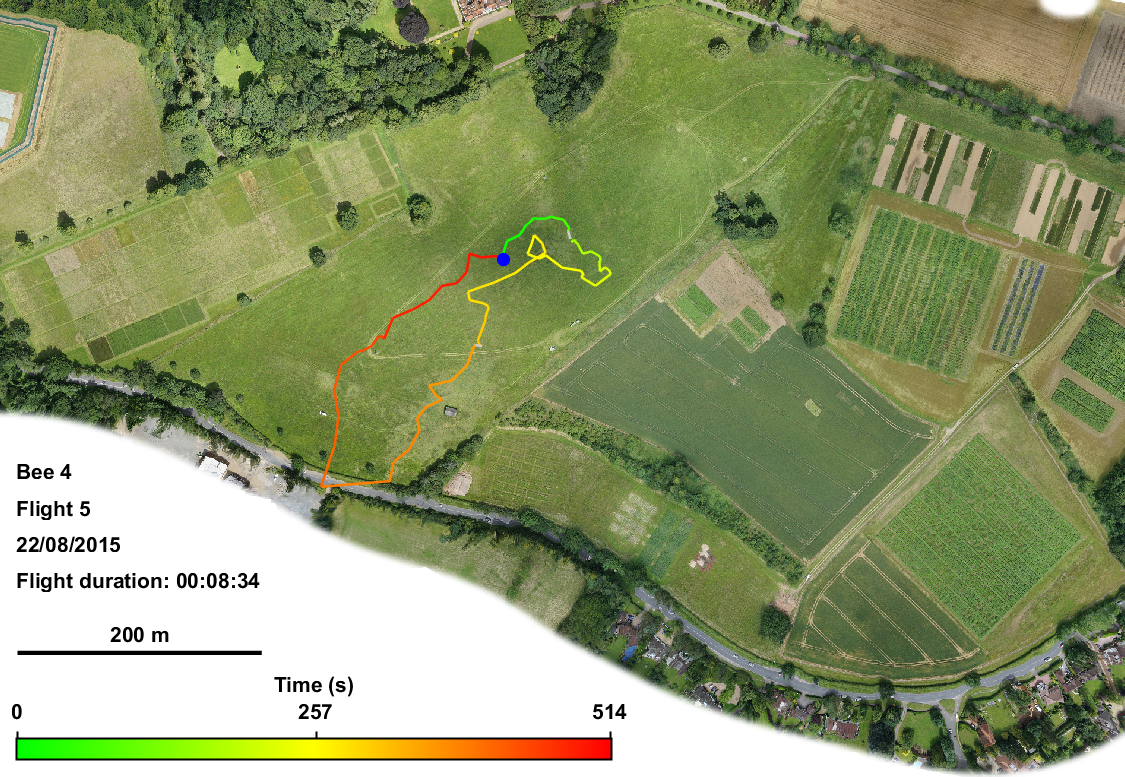

Supplement: S7 File — All flights made by Bee 4 during the period 21/08/15–03/09/15. Each figure represents a single flight. Bee ID, flight number, date of recording and the duration of each flight are shown in the bottom left corner of each figure. The position of the nest is marked by a blue circle. Colours represent the time from the start of each flight: initial portion of each flight is in green, changing smoothly through yellow until the end of the flight is shown in red. Grey dashed lines are used to join radar observations made more than 30 s apart, when the bee’s location was uncertain. (ZIP) [file pone.0160333.s009.zip › S219 Fig.tif]

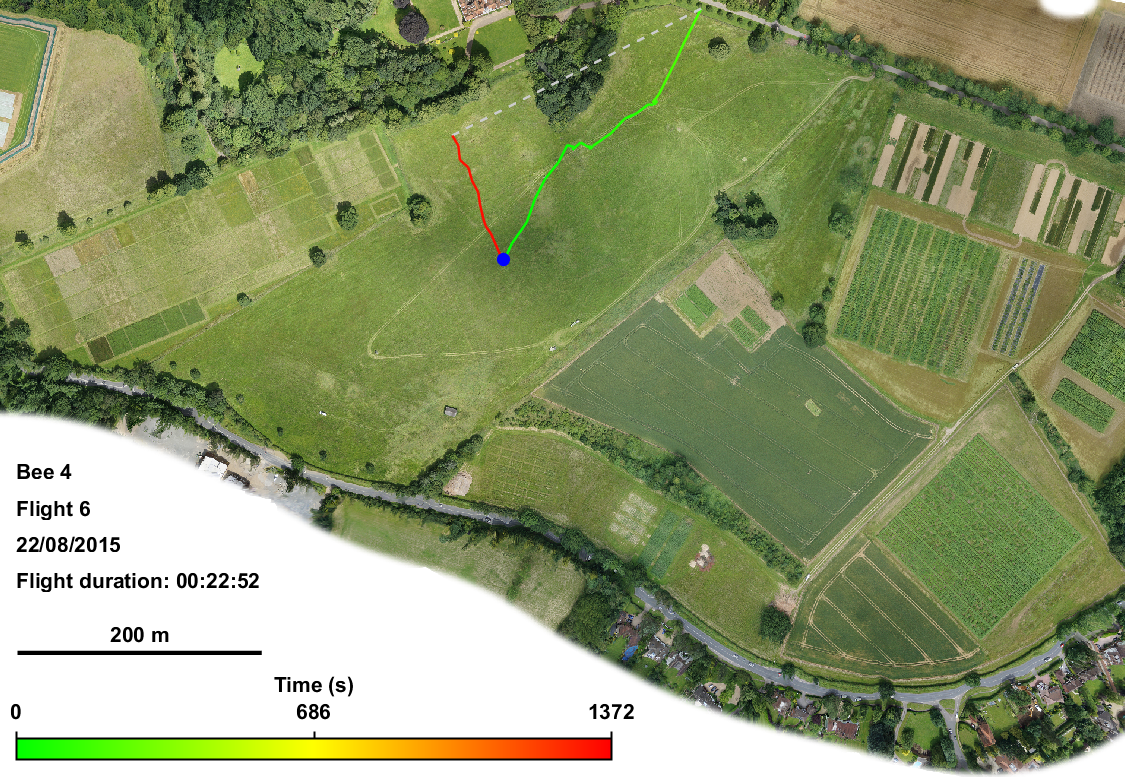

Supplement: S7 File — All flights made by Bee 4 during the period 21/08/15–03/09/15. Each figure represents a single flight. Bee ID, flight number, date of recording and the duration of each flight are shown in the bottom left corner of each figure. The position of the nest is marked by a blue circle. Colours represent the time from the start of each flight: initial portion of each flight is in green, changing smoothly through yellow until the end of the flight is shown in red. Grey dashed lines are used to join radar observations made more than 30 s apart, when the bee’s location was uncertain. (ZIP) [file pone.0160333.s009.zip › S220 Fig.tif]

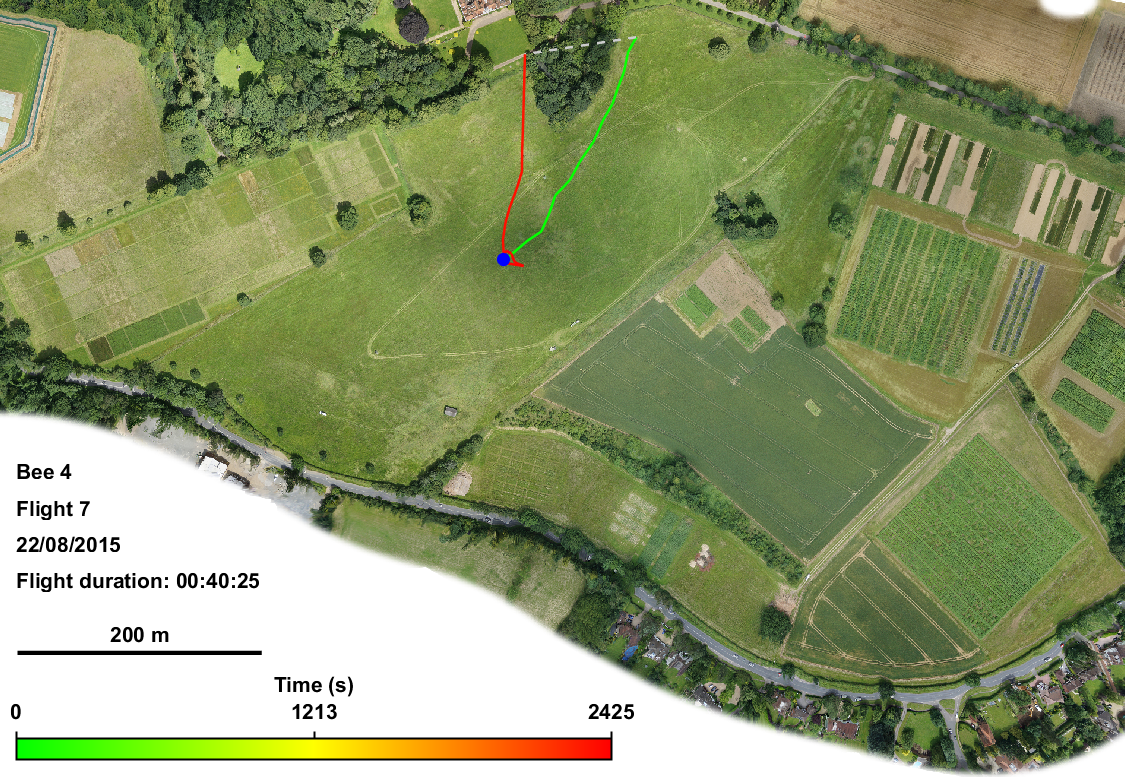

Supplement: S7 File — All flights made by Bee 4 during the period 21/08/15–03/09/15. Each figure represents a single flight. Bee ID, flight number, date of recording and the duration of each flight are shown in the bottom left corner of each figure. The position of the nest is marked by a blue circle. Colours represent the time from the start of each flight: initial portion of each flight is in green, changing smoothly through yellow until the end of the flight is shown in red. Grey dashed lines are used to join radar observations made more than 30 s apart, when the bee’s location was uncertain. (ZIP) [file pone.0160333.s009.zip › S221 Fig.tif]

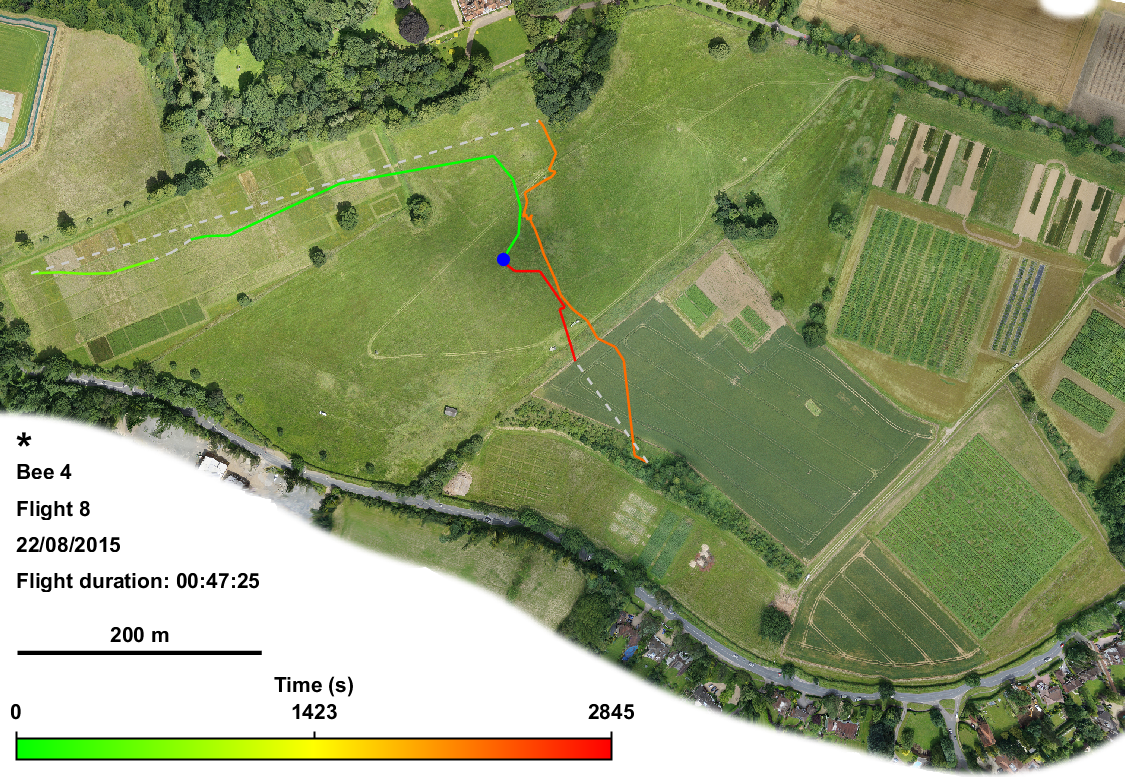

Supplement: S7 File — All flights made by Bee 4 during the period 21/08/15–03/09/15. Each figure represents a single flight. Bee ID, flight number, date of recording and the duration of each flight are shown in the bottom left corner of each figure. The position of the nest is marked by a blue circle. Colours represent the time from the start of each flight: initial portion of each flight is in green, changing smoothly through yellow until the end of the flight is shown in red. Grey dashed lines are used to join radar observations made more than 30 s apart, when the bee’s location was uncertain. (ZIP) [file pone.0160333.s009.zip › S222 Fig.tif]

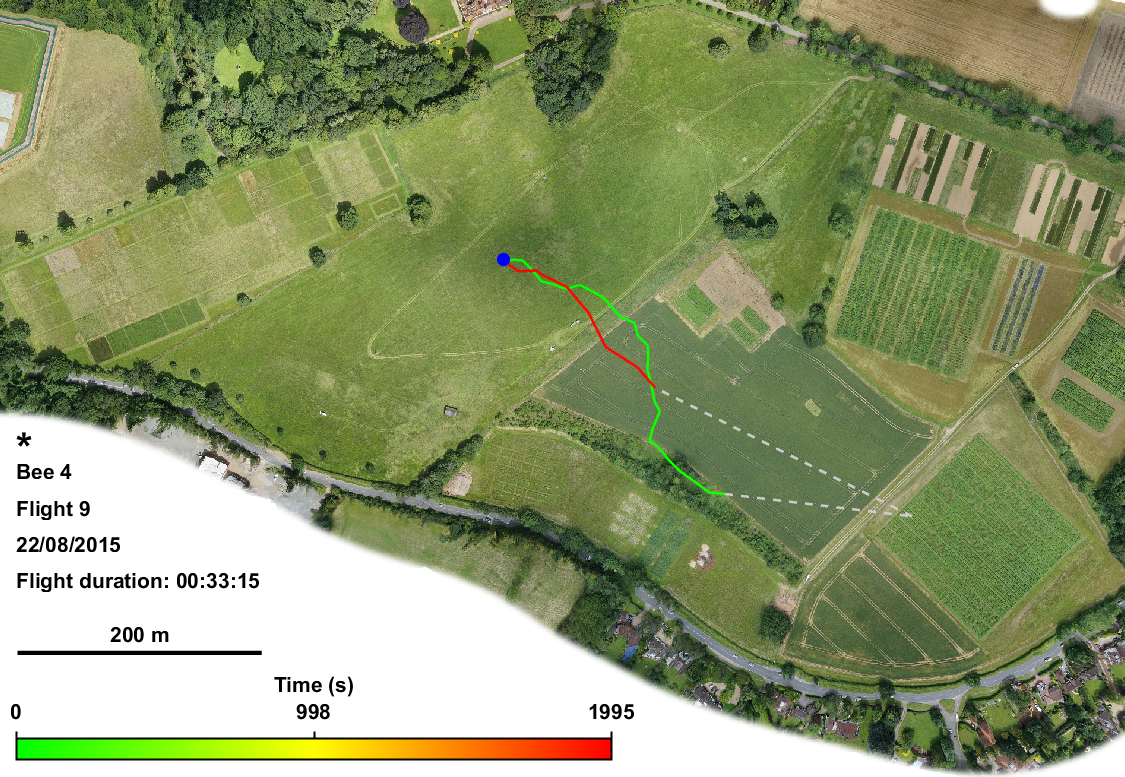

Supplement: S7 File — All flights made by Bee 4 during the period 21/08/15–03/09/15. Each figure represents a single flight. Bee ID, flight number, date of recording and the duration of each flight are shown in the bottom left corner of each figure. The position of the nest is marked by a blue circle. Colours represent the time from the start of each flight: initial portion of each flight is in green, changing smoothly through yellow until the end of the flight is shown in red. Grey dashed lines are used to join radar observations made more than 30 s apart, when the bee’s location was uncertain. (ZIP) [file pone.0160333.s009.zip › S223 Fig.tif]

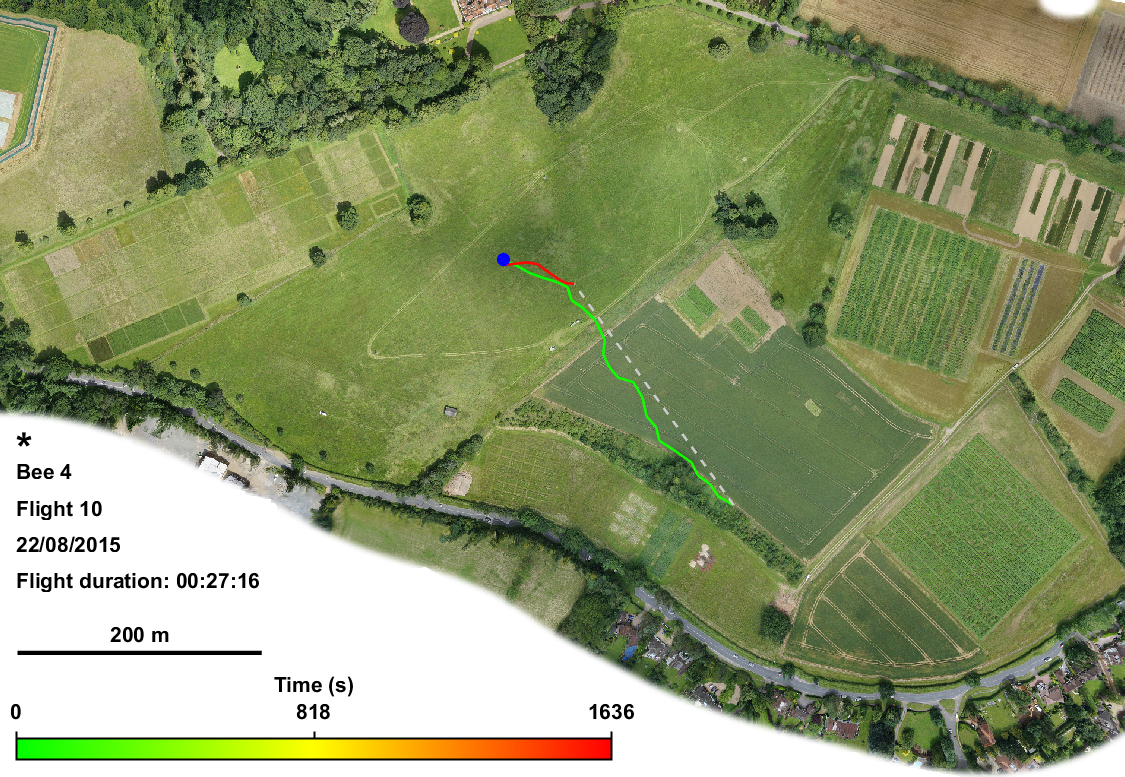

Supplement: S7 File — All flights made by Bee 4 during the period 21/08/15–03/09/15. Each figure represents a single flight. Bee ID, flight number, date of recording and the duration of each flight are shown in the bottom left corner of each figure. The position of the nest is marked by a blue circle. Colours represent the time from the start of each flight: initial portion of each flight is in green, changing smoothly through yellow until the end of the flight is shown in red. Grey dashed lines are used to join radar observations made more than 30 s apart, when the bee’s location was uncertain. (ZIP) [file pone.0160333.s009.zip › S224 Fig.tif]

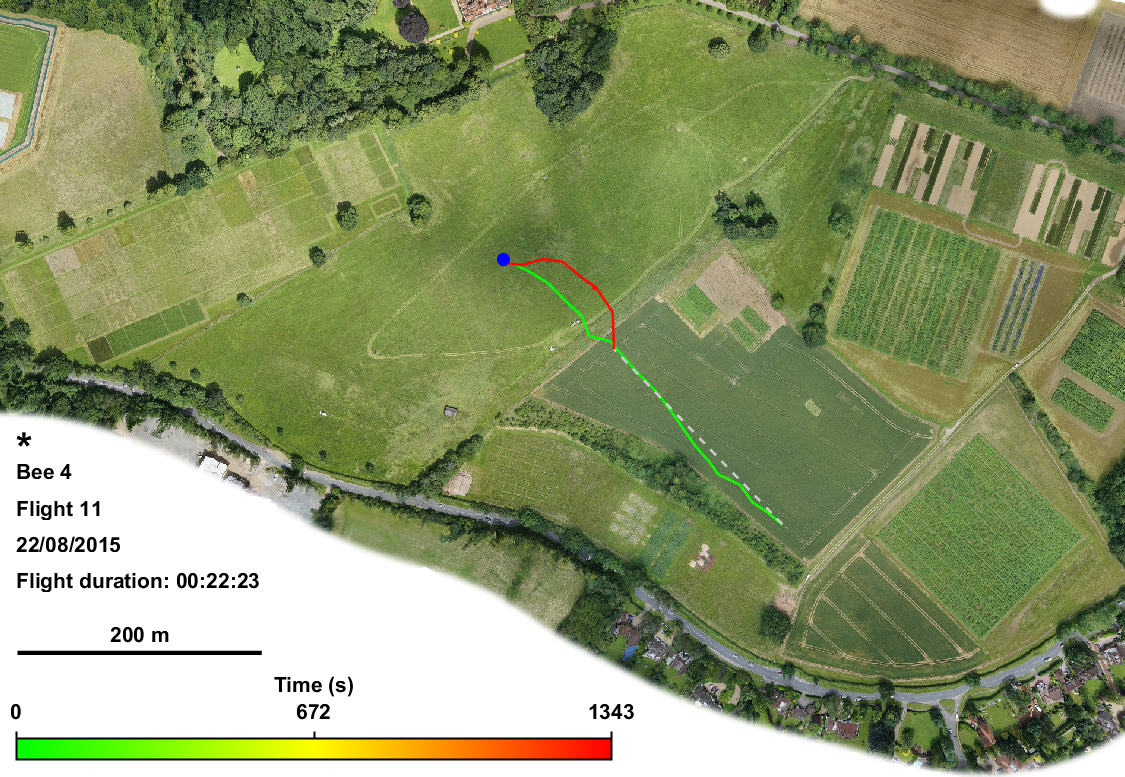

Supplement: S7 File — All flights made by Bee 4 during the period 21/08/15–03/09/15. Each figure represents a single flight. Bee ID, flight number, date of recording and the duration of each flight are shown in the bottom left corner of each figure. The position of the nest is marked by a blue circle. Colours represent the time from the start of each flight: initial portion of each flight is in green, changing smoothly through yellow until the end of the flight is shown in red. Grey dashed lines are used to join radar observations made more than 30 s apart, when the bee’s location was uncertain. (ZIP) [file pone.0160333.s009.zip › S225 Fig.tif]

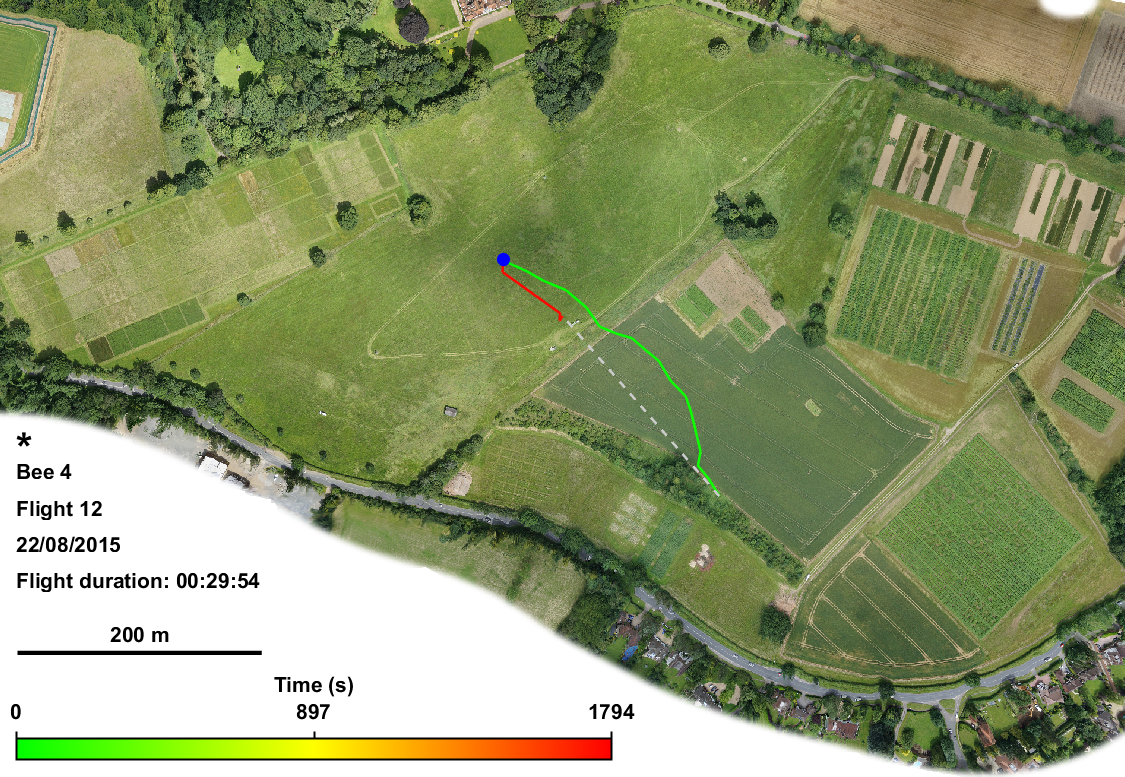

Supplement: S7 File — All flights made by Bee 4 during the period 21/08/15–03/09/15. Each figure represents a single flight. Bee ID, flight number, date of recording and the duration of each flight are shown in the bottom left corner of each figure. The position of the nest is marked by a blue circle. Colours represent the time from the start of each flight: initial portion of each flight is in green, changing smoothly through yellow until the end of the flight is shown in red. Grey dashed lines are used to join radar observations made more than 30 s apart, when the bee’s location was uncertain. (ZIP) [file pone.0160333.s009.zip › S226 Fig.tif]

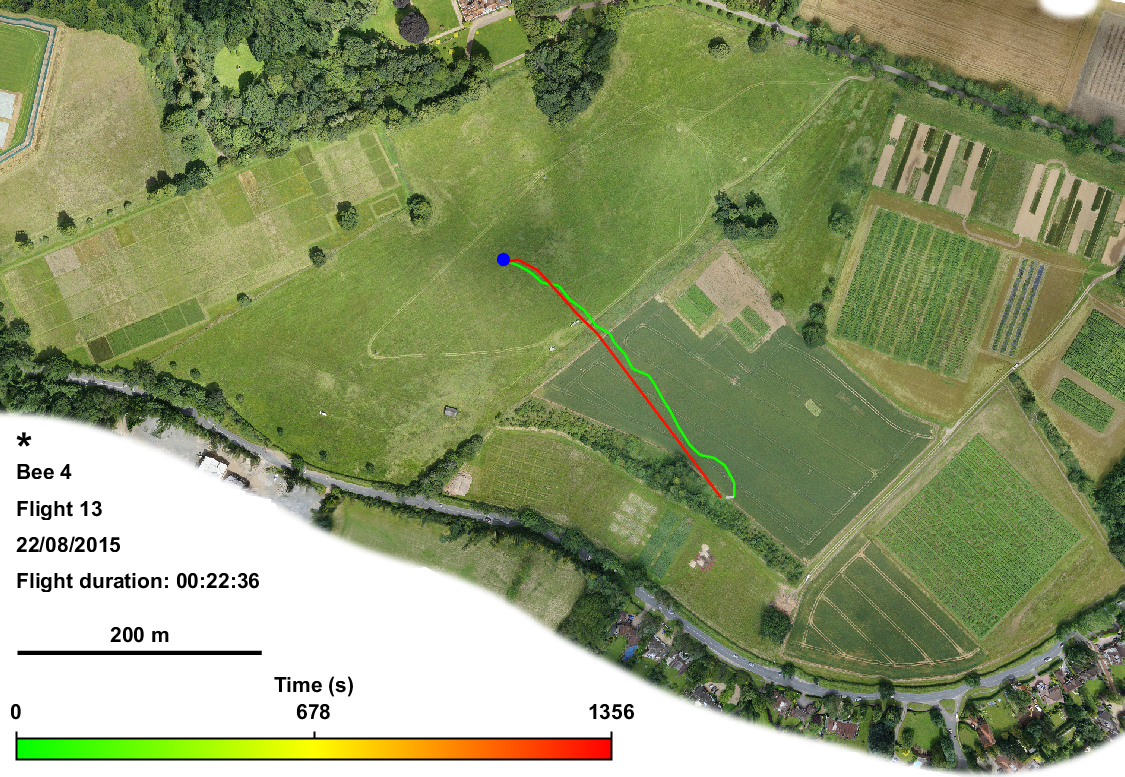

Supplement: S7 File — All flights made by Bee 4 during the period 21/08/15–03/09/15. Each figure represents a single flight. Bee ID, flight number, date of recording and the duration of each flight are shown in the bottom left corner of each figure. The position of the nest is marked by a blue circle. Colours represent the time from the start of each flight: initial portion of each flight is in green, changing smoothly through yellow until the end of the flight is shown in red. Grey dashed lines are used to join radar observations made more than 30 s apart, when the bee’s location was uncertain. (ZIP) [file pone.0160333.s009.zip › S227 Fig.tif]

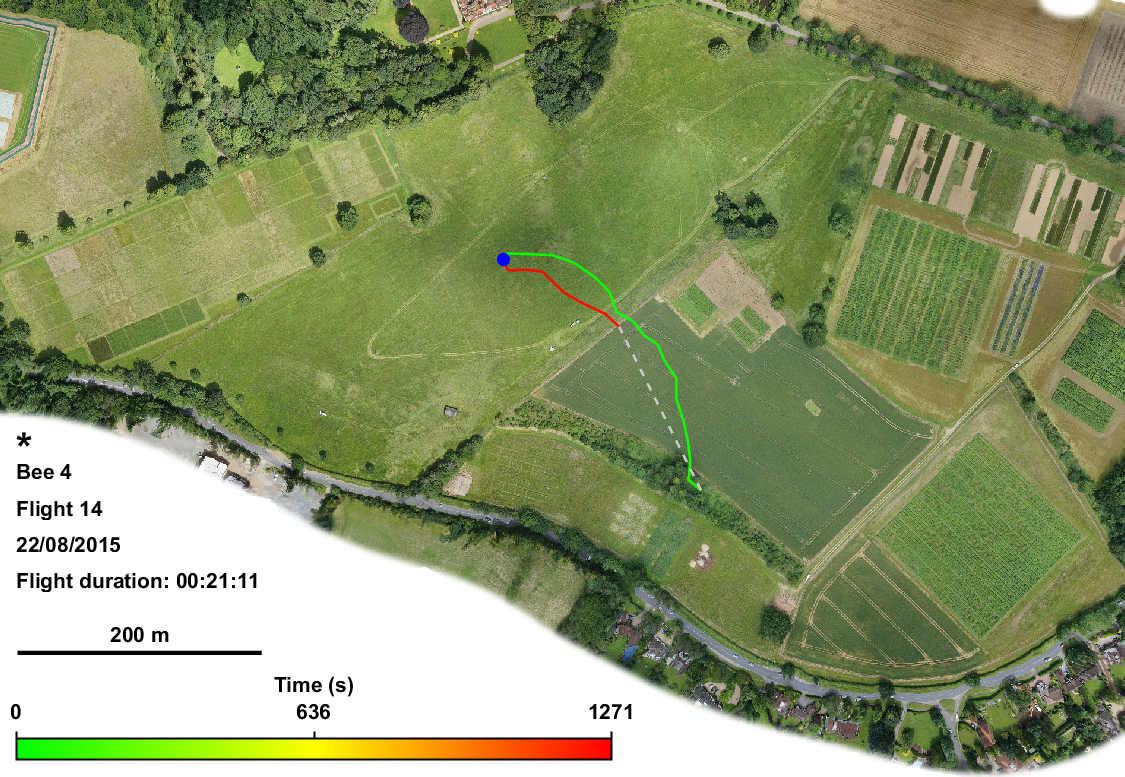

Supplement: S7 File — All flights made by Bee 4 during the period 21/08/15–03/09/15. Each figure represents a single flight. Bee ID, flight number, date of recording and the duration of each flight are shown in the bottom left corner of each figure. The position of the nest is marked by a blue circle. Colours represent the time from the start of each flight: initial portion of each flight is in green, changing smoothly through yellow until the end of the flight is shown in red. Grey dashed lines are used to join radar observations made more than 30 s apart, when the bee’s location was uncertain. (ZIP) [file pone.0160333.s009.zip › S228 Fig.tif]

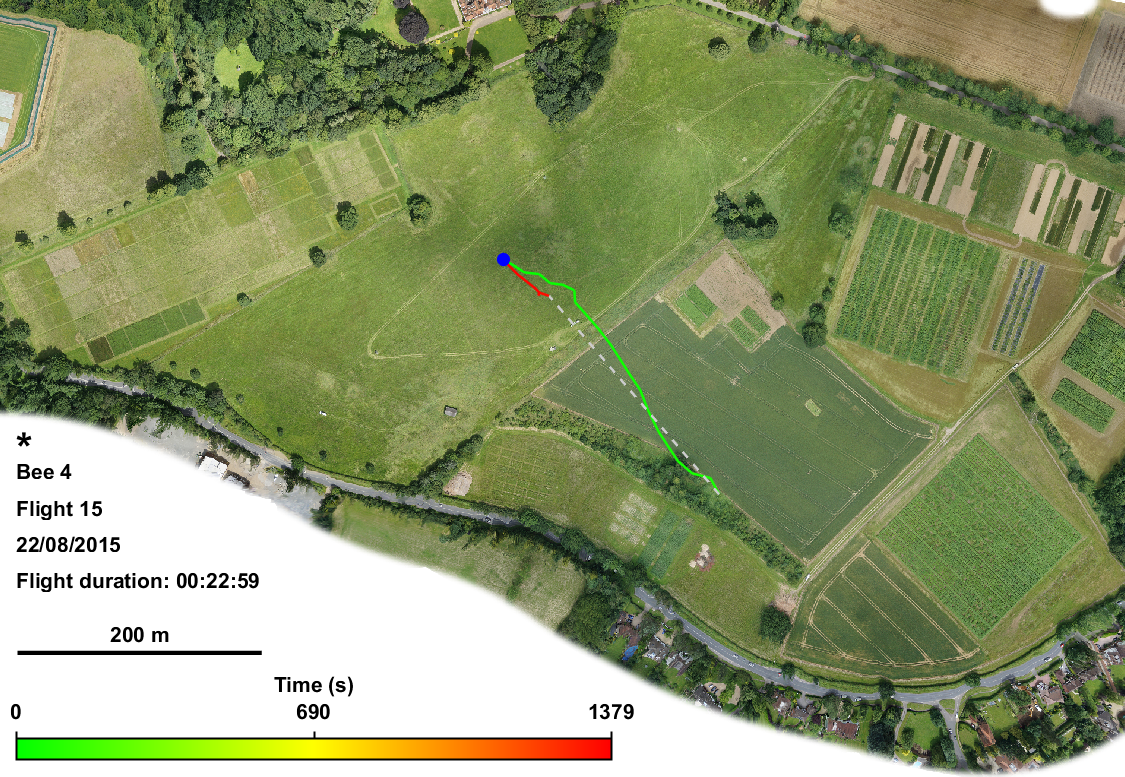

Supplement: S7 File — All flights made by Bee 4 during the period 21/08/15–03/09/15. Each figure represents a single flight. Bee ID, flight number, date of recording and the duration of each flight are shown in the bottom left corner of each figure. The position of the nest is marked by a blue circle. Colours represent the time from the start of each flight: initial portion of each flight is in green, changing smoothly through yellow until the end of the flight is shown in red. Grey dashed lines are used to join radar observations made more than 30 s apart, when the bee’s location was uncertain. (ZIP) [file pone.0160333.s009.zip › S229 Fig.tif]

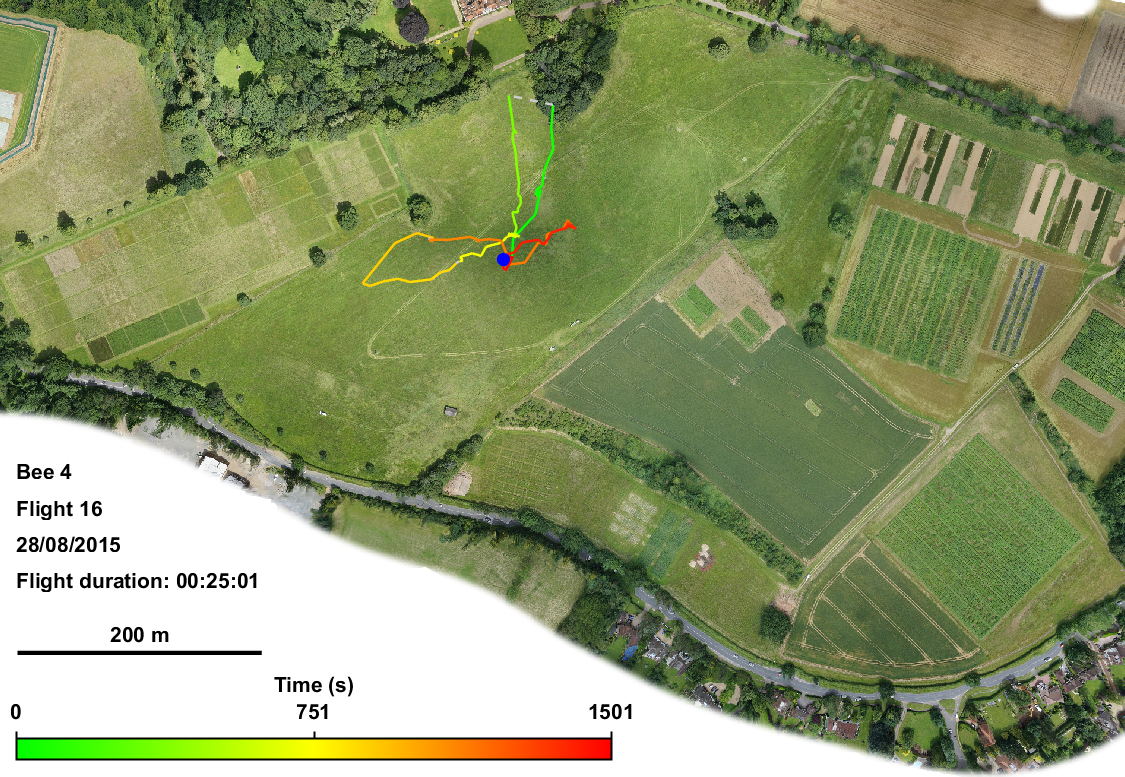

Supplement: S7 File — All flights made by Bee 4 during the period 21/08/15–03/09/15. Each figure represents a single flight. Bee ID, flight number, date of recording and the duration of each flight are shown in the bottom left corner of each figure. The position of the nest is marked by a blue circle. Colours represent the time from the start of each flight: initial portion of each flight is in green, changing smoothly through yellow until the end of the flight is shown in red. Grey dashed lines are used to join radar observations made more than 30 s apart, when the bee’s location was uncertain. (ZIP) [file pone.0160333.s009.zip › S230 Fig.tif]

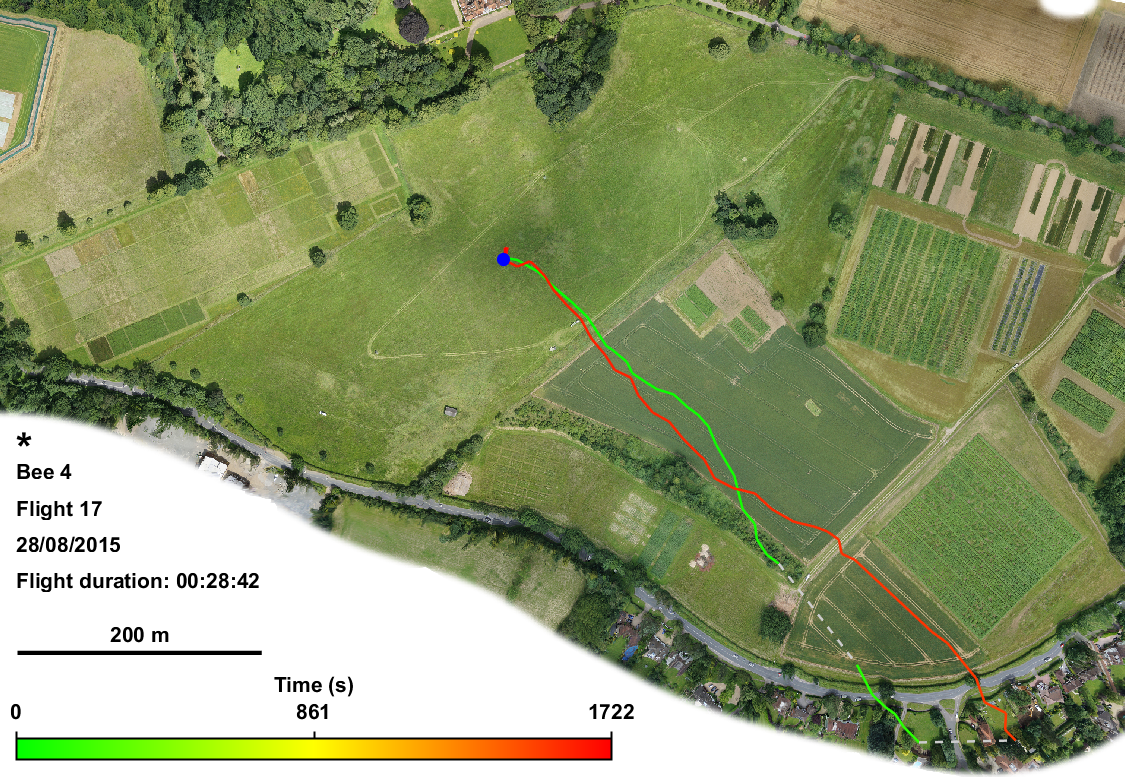

Supplement: S7 File — All flights made by Bee 4 during the period 21/08/15–03/09/15. Each figure represents a single flight. Bee ID, flight number, date of recording and the duration of each flight are shown in the bottom left corner of each figure. The position of the nest is marked by a blue circle. Colours represent the time from the start of each flight: initial portion of each flight is in green, changing smoothly through yellow until the end of the flight is shown in red. Grey dashed lines are used to join radar observations made more than 30 s apart, when the bee’s location was uncertain. (ZIP) [file pone.0160333.s009.zip › S231 Fig.tif]

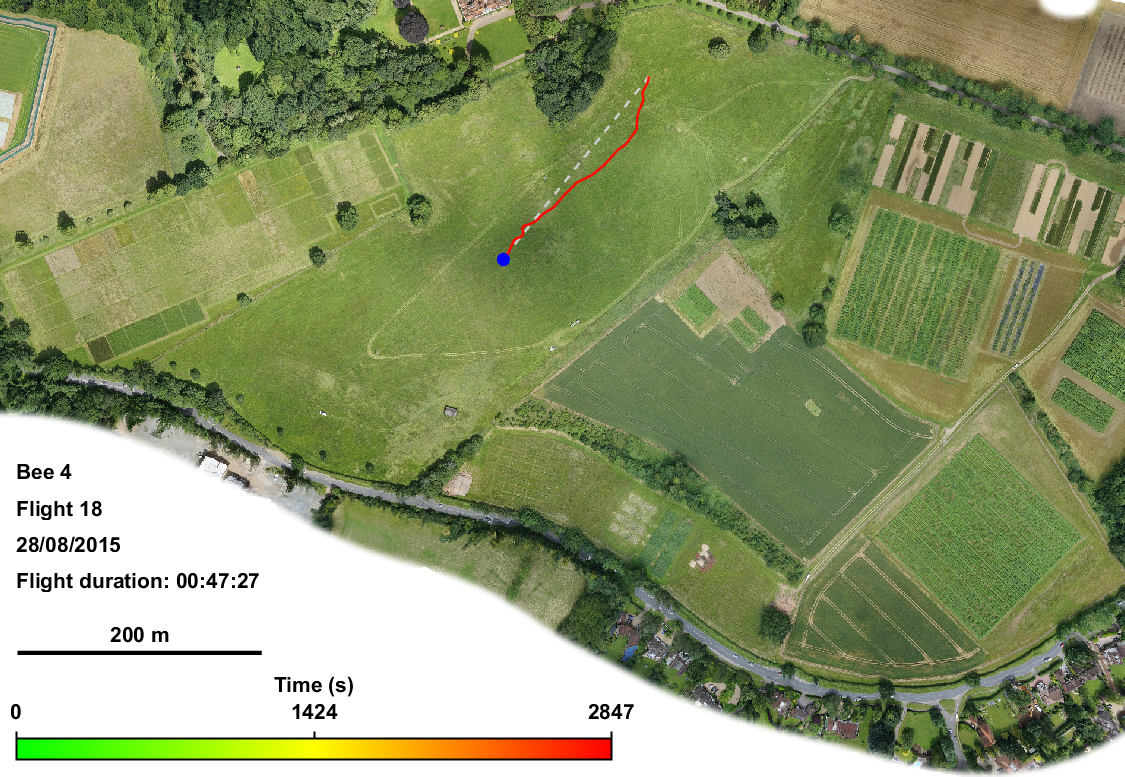

Supplement: S7 File — All flights made by Bee 4 during the period 21/08/15–03/09/15. Each figure represents a single flight. Bee ID, flight number, date of recording and the duration of each flight are shown in the bottom left corner of each figure. The position of the nest is marked by a blue circle. Colours represent the time from the start of each flight: initial portion of each flight is in green, changing smoothly through yellow until the end of the flight is shown in red. Grey dashed lines are used to join radar observations made more than 30 s apart, when the bee’s location was uncertain. (ZIP) [file pone.0160333.s009.zip › S232 Fig.tif]

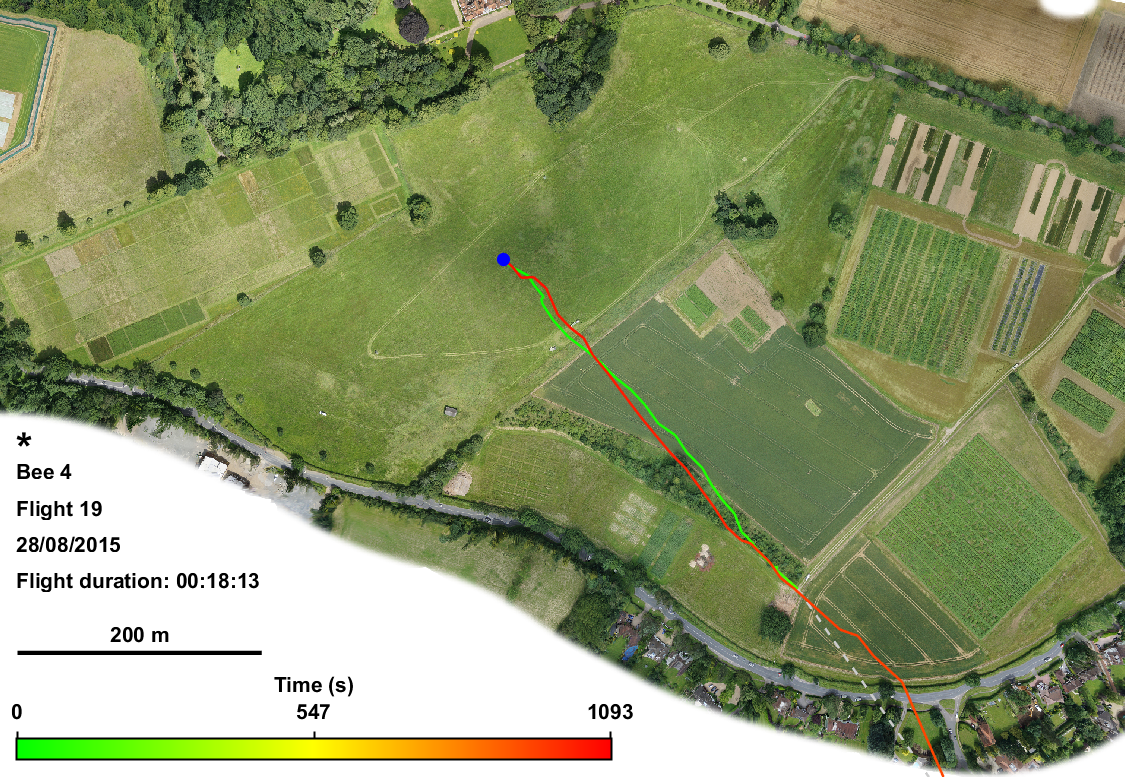

Supplement: S7 File — All flights made by Bee 4 during the period 21/08/15–03/09/15. Each figure represents a single flight. Bee ID, flight number, date of recording and the duration of each flight are shown in the bottom left corner of each figure. The position of the nest is marked by a blue circle. Colours represent the time from the start of each flight: initial portion of each flight is in green, changing smoothly through yellow until the end of the flight is shown in red. Grey dashed lines are used to join radar observations made more than 30 s apart, when the bee’s location was uncertain. (ZIP) [file pone.0160333.s009.zip › S233 Fig.tif]

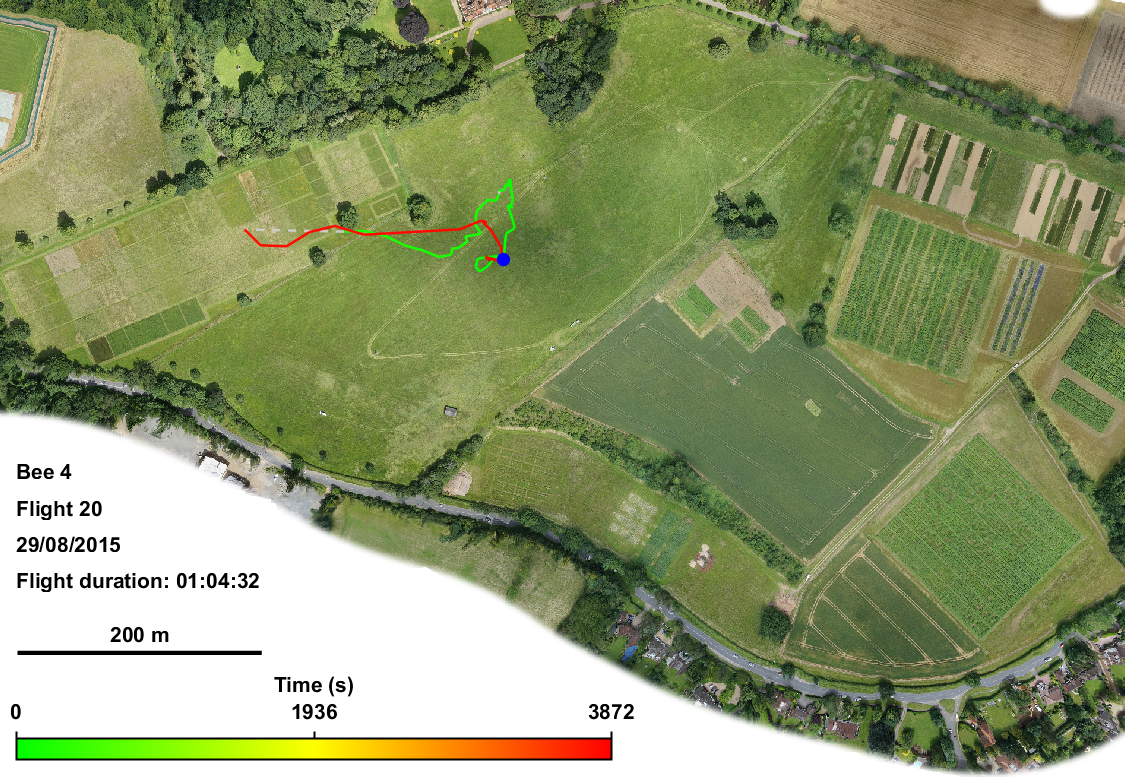

Supplement: S7 File — All flights made by Bee 4 during the period 21/08/15–03/09/15. Each figure represents a single flight. Bee ID, flight number, date of recording and the duration of each flight are shown in the bottom left corner of each figure. The position of the nest is marked by a blue circle. Colours represent the time from the start of each flight: initial portion of each flight is in green, changing smoothly through yellow until the end of the flight is shown in red. Grey dashed lines are used to join radar observations made more than 30 s apart, when the bee’s location was uncertain. (ZIP) [file pone.0160333.s009.zip › S234 Fig.tif]

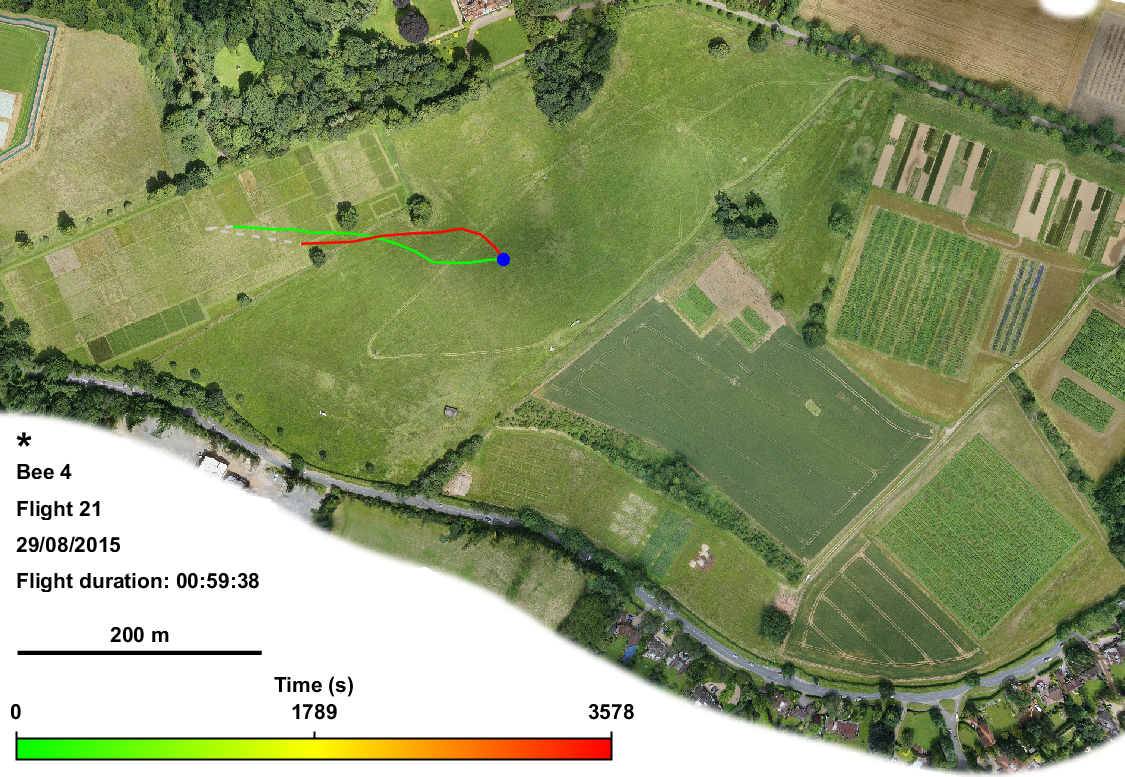

Supplement: S7 File — All flights made by Bee 4 during the period 21/08/15–03/09/15. Each figure represents a single flight. Bee ID, flight number, date of recording and the duration of each flight are shown in the bottom left corner of each figure. The position of the nest is marked by a blue circle. Colours represent the time from the start of each flight: initial portion of each flight is in green, changing smoothly through yellow until the end of the flight is shown in red. Grey dashed lines are used to join radar observations made more than 30 s apart, when the bee’s location was uncertain. (ZIP) [file pone.0160333.s009.zip › S235 Fig.tif]

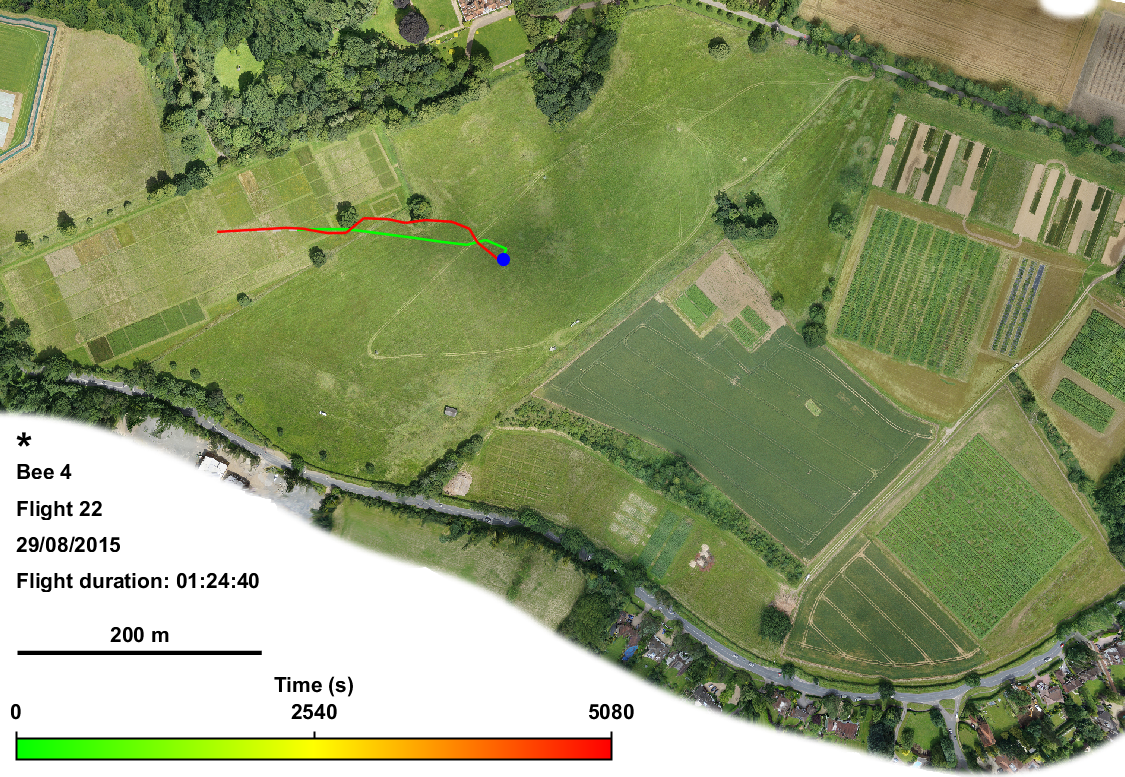

Supplement: S7 File — All flights made by Bee 4 during the period 21/08/15–03/09/15. Each figure represents a single flight. Bee ID, flight number, date of recording and the duration of each flight are shown in the bottom left corner of each figure. The position of the nest is marked by a blue circle. Colours represent the time from the start of each flight: initial portion of each flight is in green, changing smoothly through yellow until the end of the flight is shown in red. Grey dashed lines are used to join radar observations made more than 30 s apart, when the bee’s location was uncertain. (ZIP) [file pone.0160333.s009.zip › S236 Fig.tif]

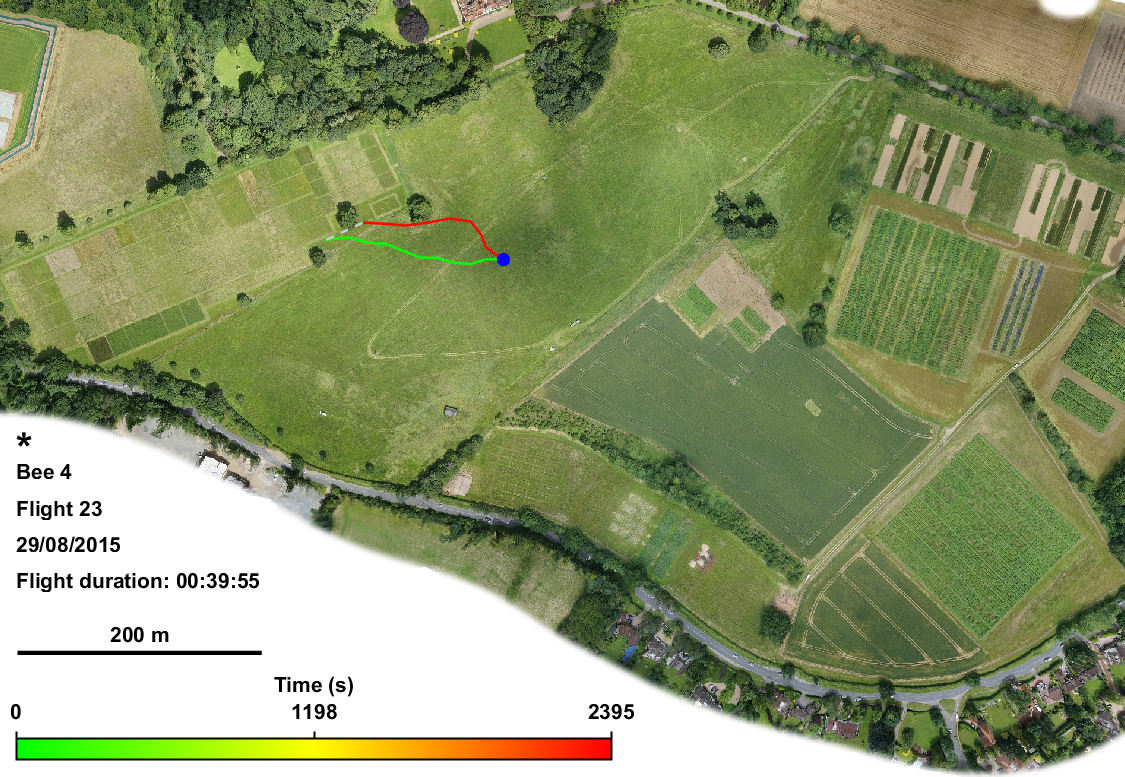

Supplement: S7 File — All flights made by Bee 4 during the period 21/08/15–03/09/15. Each figure represents a single flight. Bee ID, flight number, date of recording and the duration of each flight are shown in the bottom left corner of each figure. The position of the nest is marked by a blue circle. Colours represent the time from the start of each flight: initial portion of each flight is in green, changing smoothly through yellow until the end of the flight is shown in red. Grey dashed lines are used to join radar observations made more than 30 s apart, when the bee’s location was uncertain. (ZIP) [file pone.0160333.s009.zip › S237 Fig.tif]

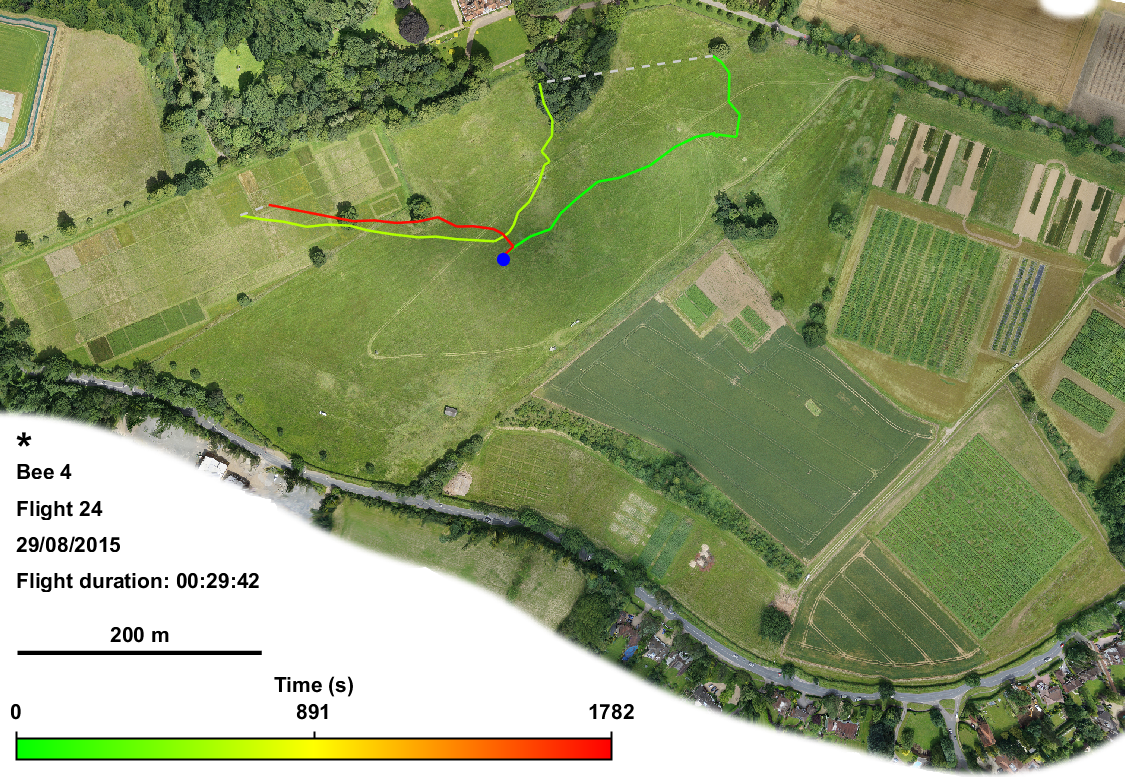

Supplement: S7 File — All flights made by Bee 4 during the period 21/08/15–03/09/15. Each figure represents a single flight. Bee ID, flight number, date of recording and the duration of each flight are shown in the bottom left corner of each figure. The position of the nest is marked by a blue circle. Colours represent the time from the start of each flight: initial portion of each flight is in green, changing smoothly through yellow until the end of the flight is shown in red. Grey dashed lines are used to join radar observations made more than 30 s apart, when the bee’s location was uncertain. (ZIP) [file pone.0160333.s009.zip › S238 Fig.tif]

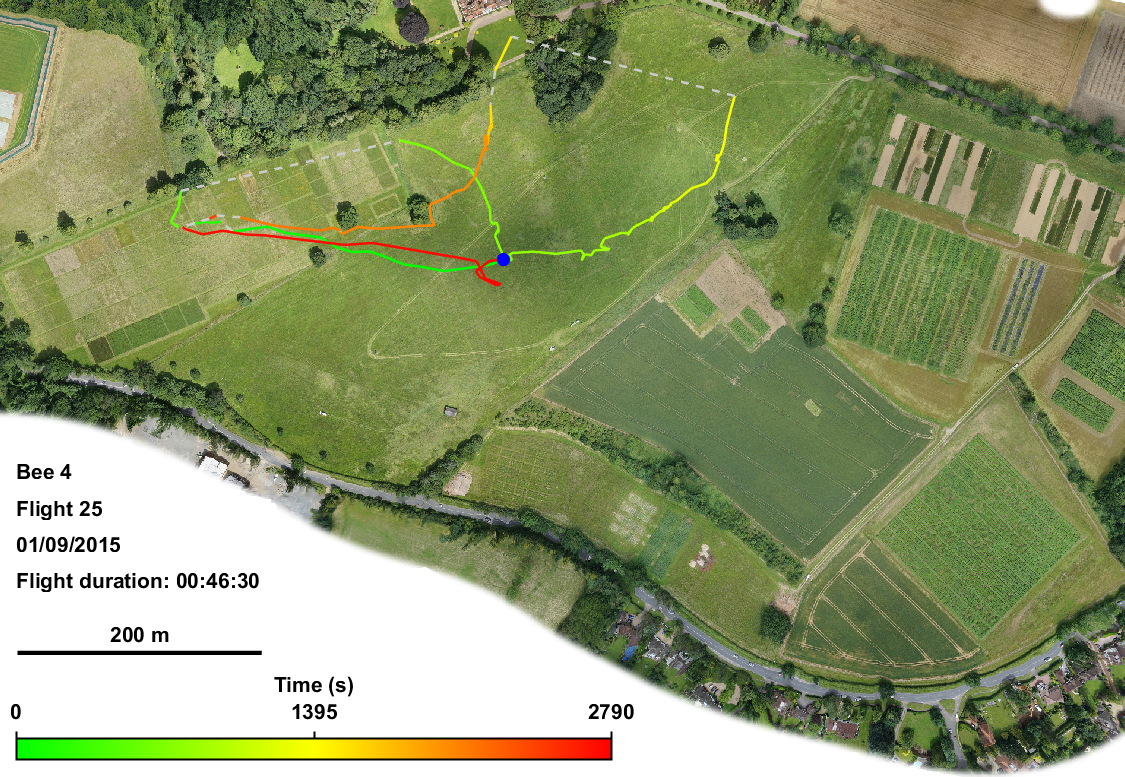

Supplement: S7 File — All flights made by Bee 4 during the period 21/08/15–03/09/15. Each figure represents a single flight. Bee ID, flight number, date of recording and the duration of each flight are shown in the bottom left corner of each figure. The position of the nest is marked by a blue circle. Colours represent the time from the start of each flight: initial portion of each flight is in green, changing smoothly through yellow until the end of the flight is shown in red. Grey dashed lines are used to join radar observations made more than 30 s apart, when the bee’s location was uncertain. (ZIP) [file pone.0160333.s009.zip › S239 Fig.tif]

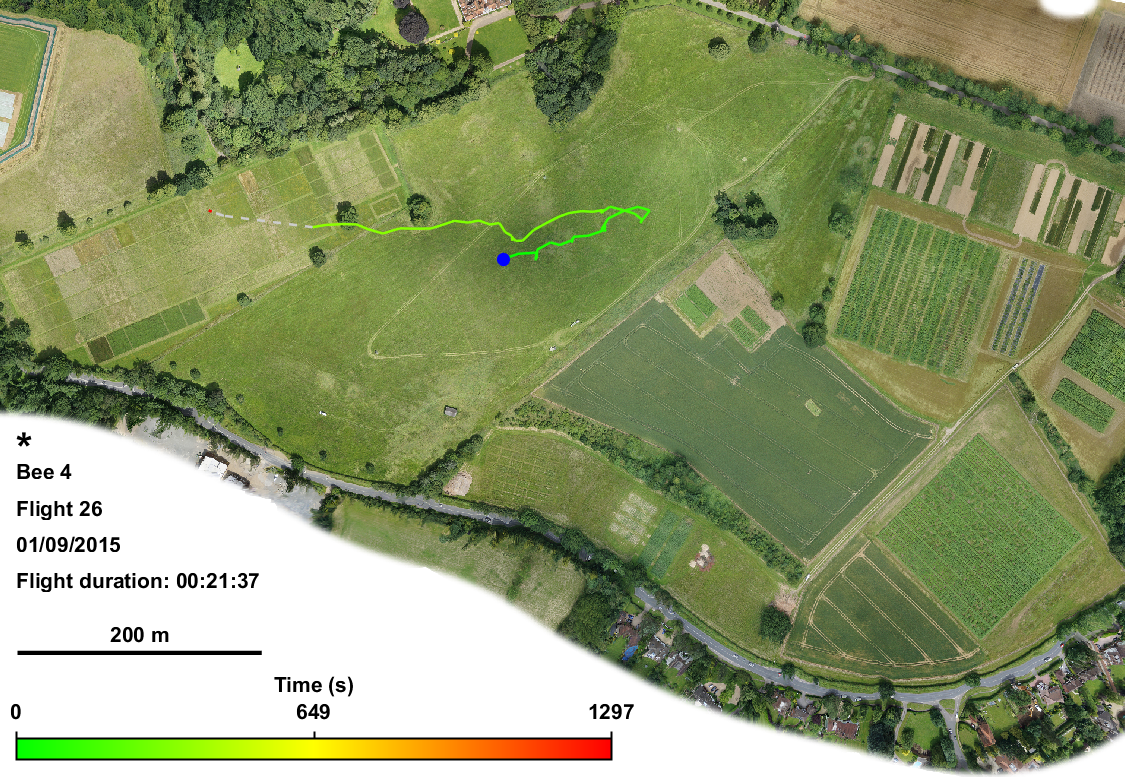

Supplement: S7 File — All flights made by Bee 4 during the period 21/08/15–03/09/15. Each figure represents a single flight. Bee ID, flight number, date of recording and the duration of each flight are shown in the bottom left corner of each figure. The position of the nest is marked by a blue circle. Colours represent the time from the start of each flight: initial portion of each flight is in green, changing smoothly through yellow until the end of the flight is shown in red. Grey dashed lines are used to join radar observations made more than 30 s apart, when the bee’s location was uncertain. (ZIP) [file pone.0160333.s009.zip › S240 Fig.tif]

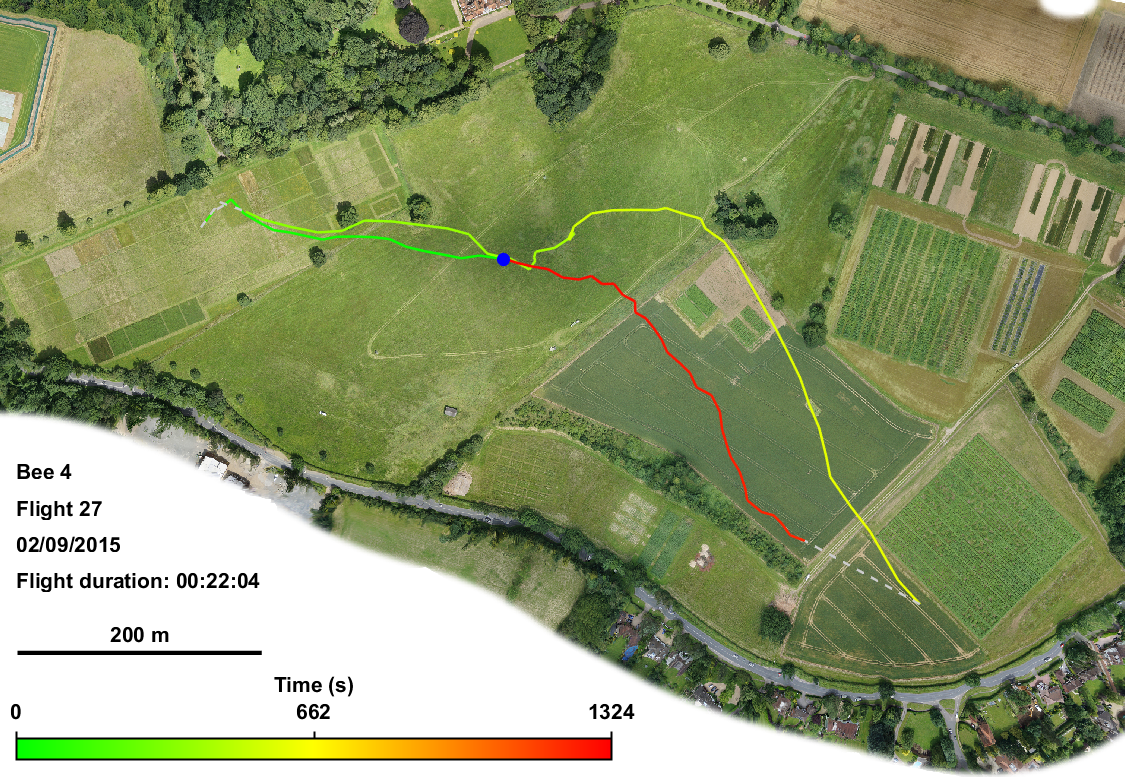

Supplement: S7 File — All flights made by Bee 4 during the period 21/08/15–03/09/15. Each figure represents a single flight. Bee ID, flight number, date of recording and the duration of each flight are shown in the bottom left corner of each figure. The position of the nest is marked by a blue circle. Colours represent the time from the start of each flight: initial portion of each flight is in green, changing smoothly through yellow until the end of the flight is shown in red. Grey dashed lines are used to join radar observations made more than 30 s apart, when the bee’s location was uncertain. (ZIP) [file pone.0160333.s009.zip › S241 Fig.tif]

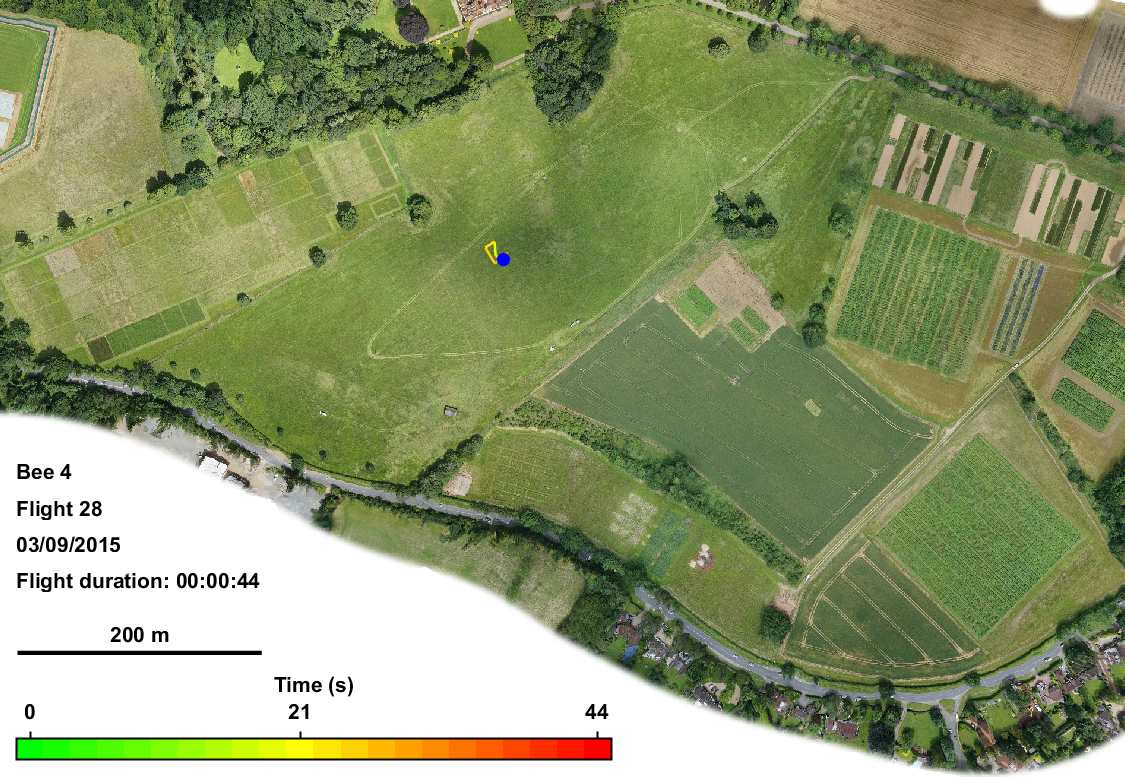

Supplement: S7 File — All flights made by Bee 4 during the period 21/08/15–03/09/15. Each figure represents a single flight. Bee ID, flight number, date of recording and the duration of each flight are shown in the bottom left corner of each figure. The position of the nest is marked by a blue circle. Colours represent the time from the start of each flight: initial portion of each flight is in green, changing smoothly through yellow until the end of the flight is shown in red. Grey dashed lines are used to join radar observations made more than 30 s apart, when the bee’s location was uncertain. (ZIP) [file pone.0160333.s009.zip › S242 Fig.tif]

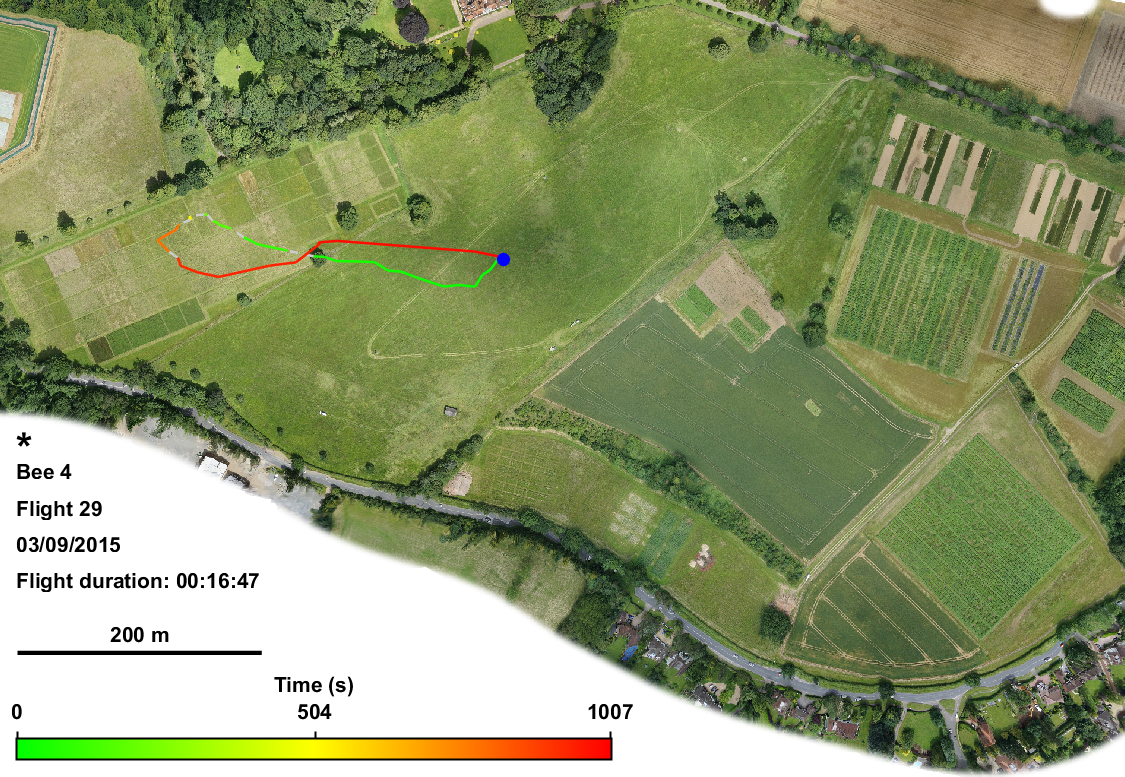

Supplement: S7 File — All flights made by Bee 4 during the period 21/08/15–03/09/15. Each figure represents a single flight. Bee ID, flight number, date of recording and the duration of each flight are shown in the bottom left corner of each figure. The position of the nest is marked by a blue circle. Colours represent the time from the start of each flight: initial portion of each flight is in green, changing smoothly through yellow until the end of the flight is shown in red. Grey dashed lines are used to join radar observations made more than 30 s apart, when the bee’s location was uncertain. (ZIP) [file pone.0160333.s009.zip › S243 Fig.tif]

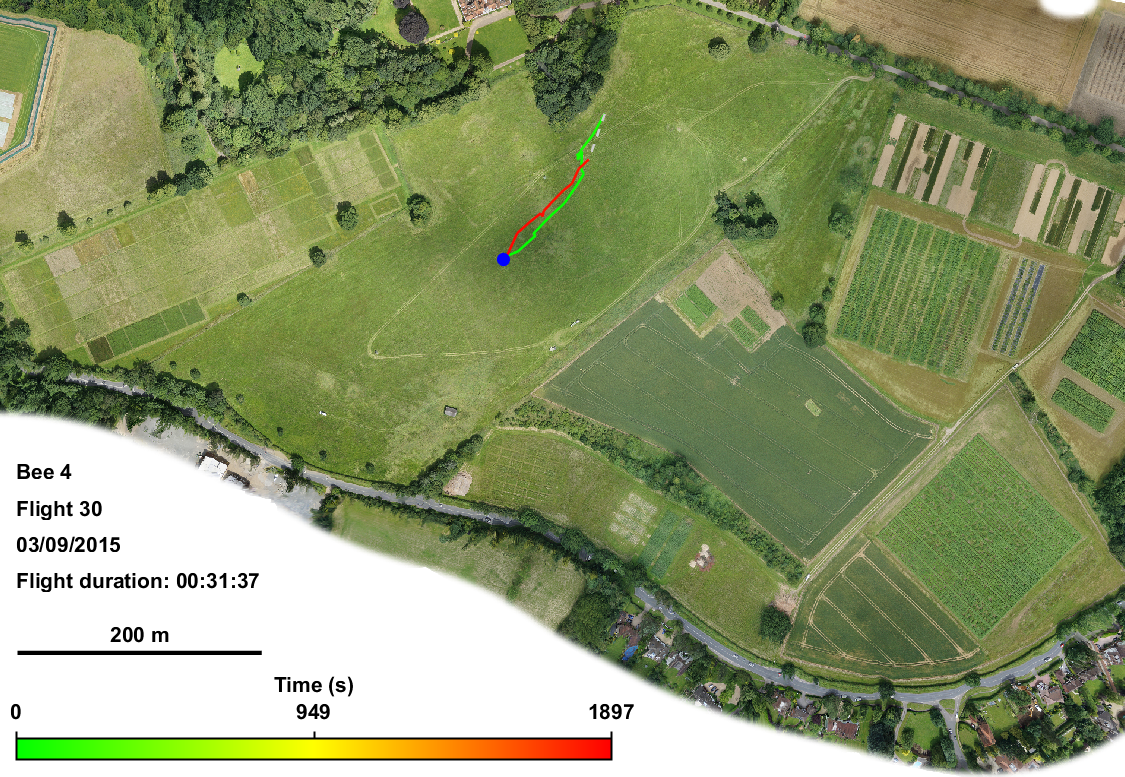

Supplement: S7 File — All flights made by Bee 4 during the period 21/08/15–03/09/15. Each figure represents a single flight. Bee ID, flight number, date of recording and the duration of each flight are shown in the bottom left corner of each figure. The position of the nest is marked by a blue circle. Colours represent the time from the start of each flight: initial portion of each flight is in green, changing smoothly through yellow until the end of the flight is shown in red. Grey dashed lines are used to join radar observations made more than 30 s apart, when the bee’s location was uncertain. (ZIP) [file pone.0160333.s009.zip › S244 Fig.tif]

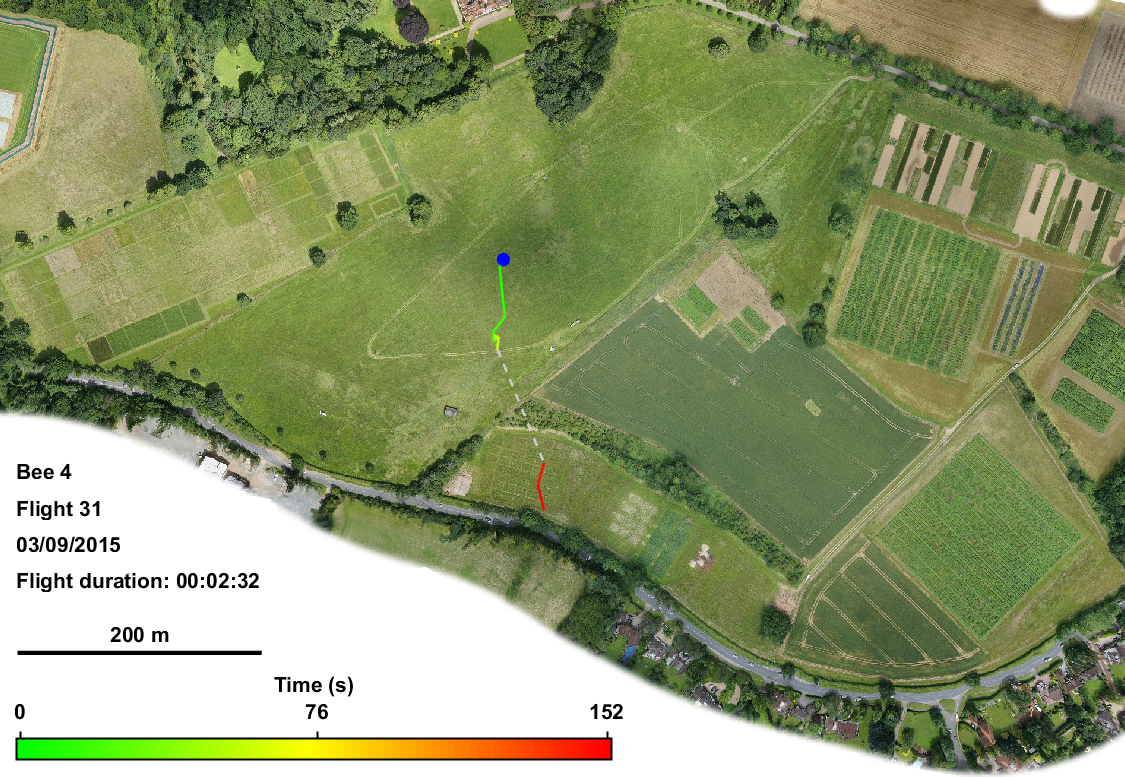

Supplement: S7 File — All flights made by Bee 4 during the period 21/08/15–03/09/15. Each figure represents a single flight. Bee ID, flight number, date of recording and the duration of each flight are shown in the bottom left corner of each figure. The position of the nest is marked by a blue circle. Colours represent the time from the start of each flight: initial portion of each flight is in green, changing smoothly through yellow until the end of the flight is shown in red. Grey dashed lines are used to join radar observations made more than 30 s apart, when the bee’s location was uncertain. (ZIP) [file pone.0160333.s009.zip › S245 Fig.tif]
